# Supplementary material for: On the importance of spatial scales on beta diversity of coral assemblages: a study from Venezuelan coral reefs
Source: PeerJ. 2020 May 4;8:e9082. doi: 10.7717/peerj.9082 (PMC7204821; doi:10.7717/peerj.9082)
Supplement: Supplemental Information 3 [file peerj-08-9082-s003.pdf]

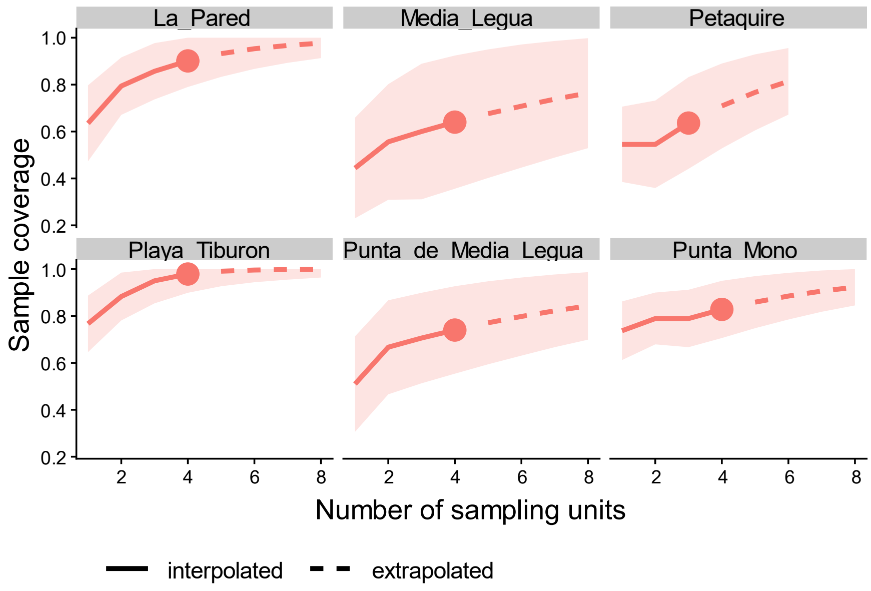

Figure 1. Sample coverage curves for sites from Chichiriviche de la Costa.

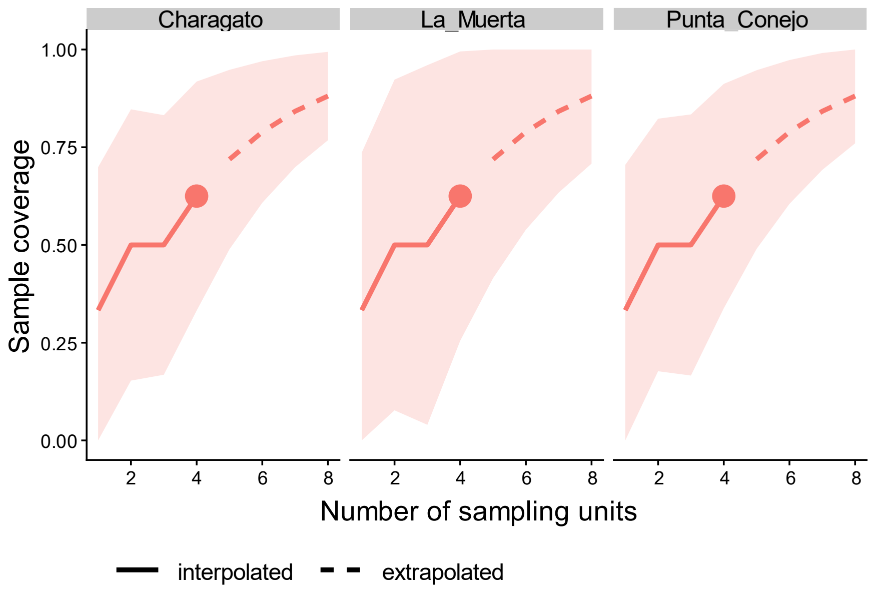

Figure 2. Sample coverage curves for sites from Cubagua.

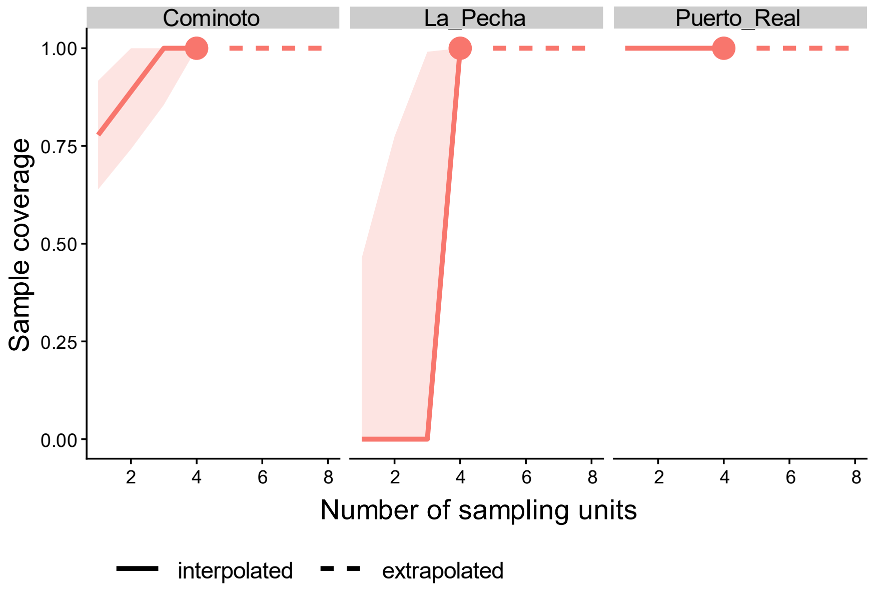

Figure 3. Sample coverage curves for sites from Los Frailes.

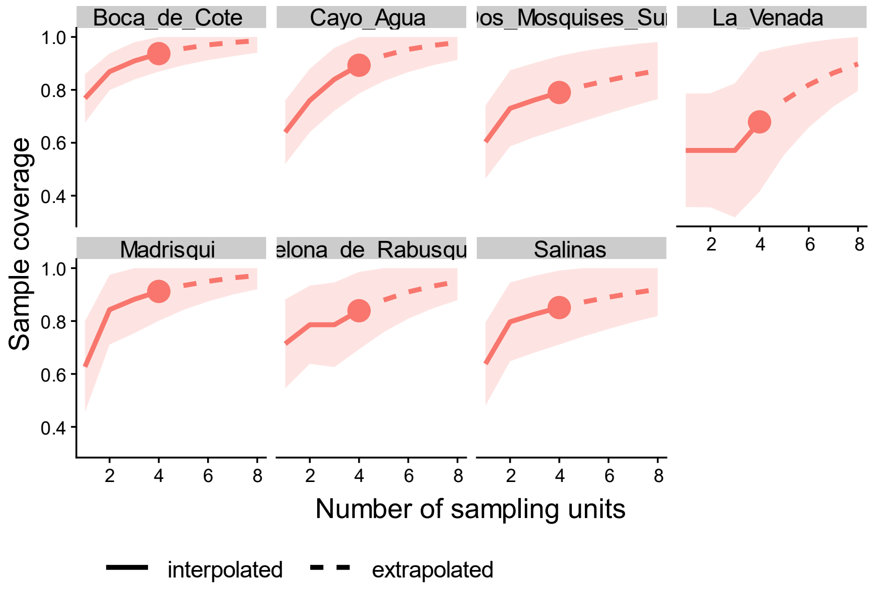

Figure 4. Sample coverage curves for sites from Los Roques N.P.

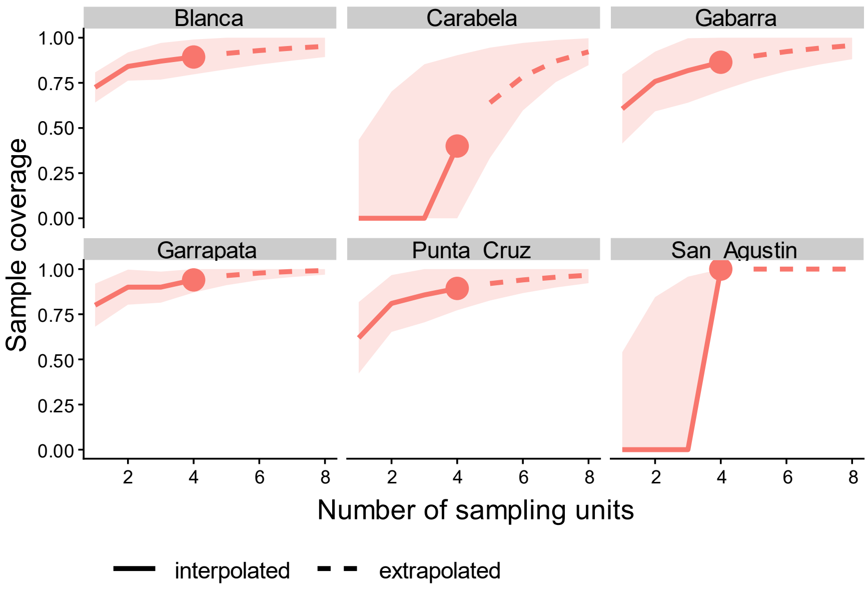

Figure 5. Sample coverage curves for sites from Mochima N.P.

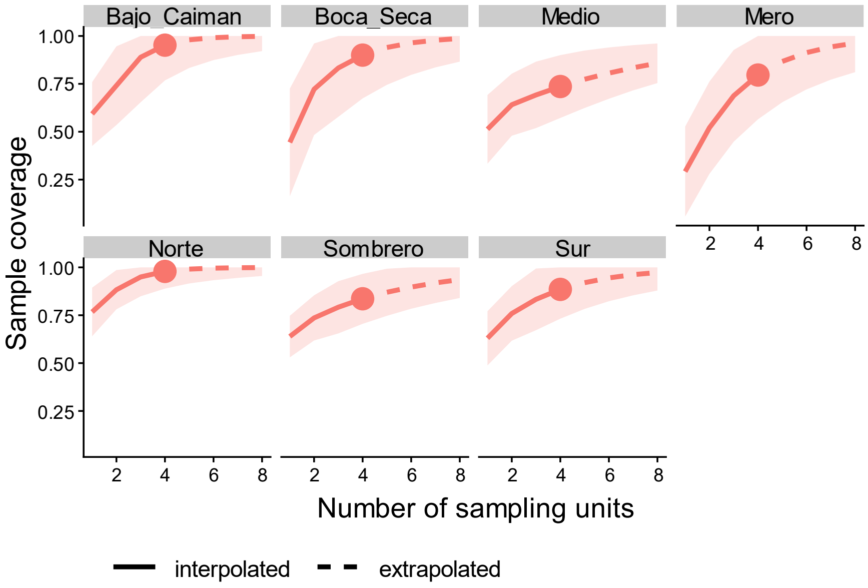

Figure 6. Sample coverage curves for sites from Morrocoy N.P.

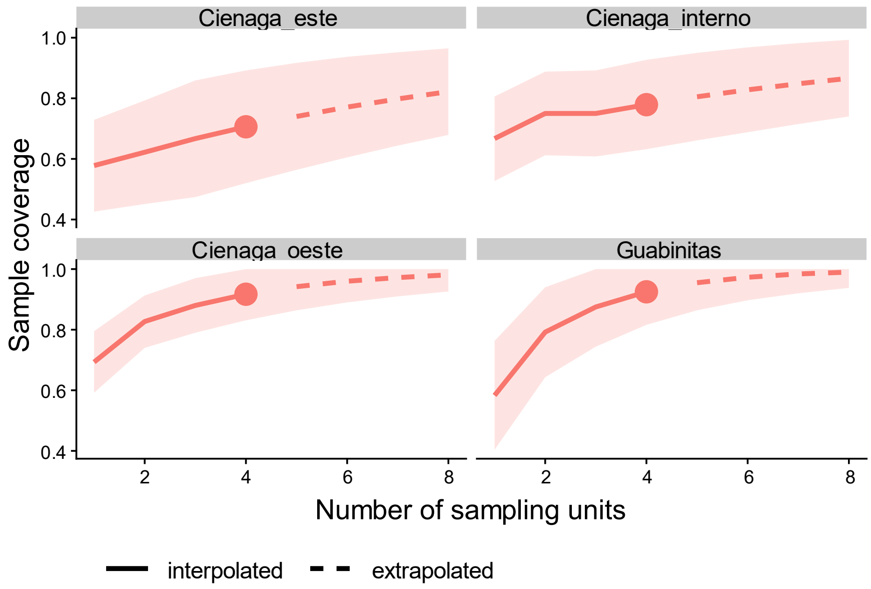

Figure 7. Sample coverage curves for sites from Ocumare de la Costa.
