# Supplementary material for: On the importance of spatial scales on beta diversity of coral assemblages: a study from Venezuelan coral reefs
Source: PeerJ. 2020 May 4;8:e9082. doi: 10.7717/peerj.9082 (PMC7204821; doi:10.7717/peerj.9082)
Supplement: Supplementary file 4 [file peerj-08-9082-s004.odt]

Groups Center  &  East

Average dissimilarity = 76,87

	Group Center	Group East	       	       	        	     

Species	    Av.Abund	  Av.Abund	Av.Diss	Diss/SD	Contrib%	Cum.%

Pseudodiploria strigosa	        1,73	      1,58	  14,77	   1,09	   19,22	19,22

Siderastrea siderea	        1,09	      0,23	   9,81	   1,06	   12,76	31,98

Colpophyllia natans	        0,70	      1,05	   9,75	   0,86	   12,68	44,65

Montastrea cavernosa	        0,92	      0,53	   8,47	   1,02	   11,02	55,67

Orbicella faveolata	        1,02	      0,07	   8,07	   0,69	   10,49	66,16

Porites astreoides	        0,62	      0,32	   5,50	   0,89	    7,15	73,32

Meandrina meandrites	        0,51	      0,03	   4,35	   0,54	    5,66	78,97

Groups Center  &  West

Average dissimilarity = 79,21

	Group Center	Group West	       	       	        	     

Species	    Av.Abund	  Av.Abund	Av.Diss	Diss/SD	Contrib%	Cum.%

Orbicella faveolata	        1,02	      2,84	  13,73	   1,37	   17,34	17,34

Pseudodiploria strigosa	        1,73	      0,39	   9,55	   1,16	   12,06	29,40

Colpophyllia natans	        0,70	      1,29	   7,76	   1,03	    9,79	39,19

Siderastrea siderea	        1,09	      0,45	   6,63	   1,11	    8,37	47,55

Montastrea cavernosa	        0,92	      0,35	   5,47	   1,02	    6,91	54,46

Porites astreoides	        0,62	      0,71	   4,98	   0,96	    6,28	60,74

Orbicella annularis	        0,14	      0,79	   4,21	   0,67	    5,31	66,06

Diploria labyrinthiformis	        0,47	      0,20	   3,13	   0,75	    3,96	70,01

Meandrina meandrites	        0,51	      0,01	   2,91	   0,53	    3,67	73,68

Acropora cervicornis	        0,00	      0,35	   2,63	   0,28	    3,32	77,00

Groups East  &  West

Average dissimilarity = 89,07

	Group East	Group West	       	       	        	     

Species	  Av.Abund	  Av.Abund	Av.Diss	Diss/SD	Contrib%	Cum.%

Orbicella faveolata	      0,07	      2,84	  20,67	   1,37	   23,20	23,20

Pseudodiploria strigosa	      1,58	      0,39	  11,17	   0,94	   12,54	35,74

Colpophyllia natans	      1,05	      1,29	  11,11	   1,00	   12,47	48,21

Porites astreoides	      0,32	      0,71	   6,41	   0,72	    7,20	55,41

Orbicella annularis	      0,00	      0,79	   4,81	   0,63	    5,40	60,81

Montastrea cavernosa	      0,53	      0,35	   4,65	   0,65	    5,22	66,03

Acropora cervicornis	      0,00	      0,35	   4,04	   0,27	    4,54	70,57

Siderastrea siderea	      0,23	      0,45	   3,86	   0,61	    4,34	74,90

Madracis mirabilis	      0,00	      0,52	   2,84	   0,42	    3,19	78,09
