# Supplementary material for: On the importance of spatial scales on beta diversity of coral assemblages: a study from Venezuelan coral reefs
Source: PeerJ. 2020 May 4;8:e9082. doi: 10.7717/peerj.9082 (PMC7204821; doi:10.7717/peerj.9082)
Supplement: Supplementary file 5 [file peerj-08-9082-s005.odt]

Groups Chichiriviche  &  Cubagua

Average dissimilarity = 76,62

	Group Chichiriviche	Group Cubagua	       	       	        	     

Species	           Av.Abund	     Av.Abund	Av.Diss	Diss/SD	Contrib%	Cum.%

Pseudodiploria strigosa	               2,36	         1,46	  18,16	   1,20	   23,69	23,69

Siderastrea siderea	               1,30	         0,15	  12,25	   1,74	   15,99	39,69

Colpophyllia natans	               0,09	         0,83	   8,00	   0,70	   10,44	50,12

Meandrina meandrites	               0,83	         0,00	   7,41	   0,75	    9,68	59,80

Montastrea cavernosa	               0,76	         0,00	   7,05	   0,92	    9,20	69,00

Diploria labyrinthiformis	               0,75	         0,00	   6,58	   1,05	    8,59	77,60

Groups Chichiriviche  &  Los Frailes

Average dissimilarity = 71,08

	Group Chichiriviche	Group Los Frailes	       	       	        	     

Species	           Av.Abund	         Av.Abund	Av.Diss	Diss/SD	Contrib%	Cum.%

Pseudodiploria strigosa	               2,36	             1,91	  16,83	   1,06	   23,67	23,67

Siderastrea siderea	               1,30	             0,00	  13,38	   2,09	   18,82	42,49

Meandrina meandrites	               0,83	             0,00	   7,51	   0,75	   10,56	53,06

Montastrea cavernosa	               0,76	             0,00	   7,15	   0,92	   10,06	63,11

Diploria labyrinthiformis	               0,75	             0,00	   6,66	   1,05	    9,38	72,49

Porites astreoides	               0,26	             0,56	   5,56	   0,96	    7,82	80,31

Groups Cubagua  &  Los Frailes

Average dissimilarity = 75,15

	Group Cubagua	Group Los Frailes	       	       	        	     

Species	     Av.Abund	         Av.Abund	Av.Diss	Diss/SD	Contrib%	Cum.%

Pseudodiploria strigosa	         1,46	             1,91	  36,74	   1,19	   48,88	48,88

Colpophyllia natans	         0,83	             0,00	  14,75	   0,63	   19,63	68,51

Porites astreoides	         0,23	             0,56	  10,41	   0,98	   13,85	82,36

Groups Chichiriviche  &  Los Roques

Average dissimilarity = 85,27

	Group Chichiriviche	Group Los Roques	       	       	        	     

Species	           Av.Abund	        Av.Abund	Av.Diss	Diss/SD	Contrib%	Cum.%

Orbicella faveolata	               0,06	            2,80	  14,17	   1,67	   16,62	16,62

Pseudodiploria strigosa	               2,36	            0,41	  11,04	   1,84	   12,94	29,57

Siderastrea siderea	               1,30	            0,79	   6,47	   1,65	    7,59	37,16

Orbicella annularis	               0,00	            1,31	   6,12	   0,87	    7,17	44,33

Colpophyllia natans	               0,09	            1,14	   5,87	   0,97	    6,89	51,22

Acropora cervicornis	               0,00	            0,70	   5,49	   0,41	    6,44	57,65

Montastrea cavernosa	               0,76	            0,59	   4,80	   1,08	    5,63	63,28

Madracis mirabilis	               0,00	            1,00	   4,50	   0,63	    5,28	68,56

Diploria labyrinthiformis	               0,75	            0,40	   4,23	   1,08	    4,96	73,52

Meandrina meandrites	               0,83	            0,02	   4,17	   0,78	    4,89	78,41

Groups Cubagua  &  Los Roques

Average dissimilarity = 90,62

	Group Cubagua	Group Los Roques	       	       	        	     

Species	     Av.Abund	        Av.Abund	Av.Diss	Diss/SD	Contrib%	Cum.%

Orbicella faveolata	         0,12	            2,80	  18,46	   1,56	   20,37	20,37

Pseudodiploria strigosa	         1,46	            0,41	   9,59	   1,07	   10,59	30,95

Colpophyllia natans	         0,83	            1,14	   9,13	   1,04	   10,07	41,02

Acropora cervicornis	         0,00	            0,70	   8,61	   0,40	    9,50	50,52

Orbicella annularis	         0,00	            1,31	   7,76	   0,87	    8,56	59,08

Madracis mirabilis	         0,00	            1,00	   5,64	   0,63	    6,22	65,30

Siderastrea siderea	         0,15	            0,79	   5,41	   0,75	    5,96	71,27

Porites astreoides	         0,23	            0,70	   5,06	   0,84	    5,58	76,85

Groups Los Frailes  &  Los Roques

Average dissimilarity = 93,53

	Group Los Frailes	Group Los Roques	       	       	        	     

Species	         Av.Abund	        Av.Abund	Av.Diss	Diss/SD	Contrib%	Cum.%

Orbicella faveolata	             0,00	            2,80	  19,02	   1,61	   20,34	20,34

Pseudodiploria strigosa	             1,91	            0,41	  12,29	   1,26	   13,14	33,48

Acropora cervicornis	             0,00	            0,70	   8,75	   0,40	    9,35	42,83

Orbicella annularis	             0,00	            1,31	   7,83	   0,87	    8,37	51,20

Colpophyllia natans	             0,00	            1,14	   7,81	   0,93	    8,35	59,55

Porites astreoides	             0,56	            0,70	   5,71	   1,05	    6,10	65,65

Madracis mirabilis	             0,00	            1,00	   5,69	   0,63	    6,08	71,73

Siderastrea siderea	             0,00	            0,79	   5,18	   0,69	    5,54	77,27

Groups Chichiriviche  &  Mochima

Average dissimilarity = 75,42

	Group Chichiriviche	Group Mochima	       	       	        	     

Species	           Av.Abund	     Av.Abund	Av.Diss	Diss/SD	Contrib%	Cum.%

Pseudodiploria strigosa	               2,36	         1,47	  16,66	   1,05	   22,09	22,09

Colpophyllia natans	               0,09	         1,69	  10,68	   1,08	   14,16	36,25

Siderastrea siderea	               1,30	         0,38	  10,32	   1,34	   13,69	49,93

Montastrea cavernosa	               0,76	         1,07	   8,53	   1,13	   11,31	61,25

Meandrina meandrites	               0,83	         0,06	   6,59	   0,72	    8,74	69,98

Diploria labyrinthiformis	               0,75	         0,00	   5,78	   0,97	    7,67	77,65

Groups Cubagua  &  Mochima

Average dissimilarity = 80,49

	Group Cubagua	Group Mochima	       	       	        	     

Species	     Av.Abund	     Av.Abund	Av.Diss	Diss/SD	Contrib%	Cum.%

Pseudodiploria strigosa	         1,46	         1,47	  24,56	   0,95	   30,51	30,51

Colpophyllia natans	         0,83	         1,69	  21,50	   0,96	   26,71	57,22

Montastrea cavernosa	         0,00	         1,07	  12,13	   0,77	   15,07	72,29

Siderastrea siderea	         0,15	         0,38	   5,18	   0,60	    6,44	78,73

Groups Los Frailes  &  Mochima

Average dissimilarity = 82,94

	Group Los Frailes	Group Mochima	       	       	        	     

Species	         Av.Abund	     Av.Abund	Av.Diss	Diss/SD	Contrib%	Cum.%

Pseudodiploria strigosa	             1,91	         1,47	  29,75	   1,03	   35,87	35,87

Colpophyllia natans	             0,00	         1,69	  16,69	   0,99	   20,12	56,00

Montastrea cavernosa	             0,00	         1,07	  12,85	   0,74	   15,50	71,49

Porites astreoides	             0,56	         0,24	   8,21	   0,84	    9,89	81,39

Groups Los Roques  &  Mochima

Average dissimilarity = 86,84

	Group Los Roques	Group Mochima	       	       	        	     

Species	        Av.Abund	     Av.Abund	Av.Diss	Diss/SD	Contrib%	Cum.%

Orbicella faveolata	            2,80	         0,09	  16,55	   1,46	   19,05	19,05

Colpophyllia natans	            1,14	         1,69	   9,58	   1,21	   11,04	30,09

Pseudodiploria strigosa	            0,41	         1,47	   7,54	   1,17	    8,68	38,77

Acropora cervicornis	            0,70	         0,00	   7,44	   0,38	    8,57	47,34

Orbicella annularis	            1,31	         0,00	   6,98	   0,84	    8,04	55,38

Montastrea cavernosa	            0,59	         1,07	   6,39	   1,04	    7,35	62,74

Siderastrea siderea	            0,79	         0,38	   5,32	   0,80	    6,13	68,87

Madracis mirabilis	            1,00	         0,00	   5,09	   0,62	    5,86	74,73

Porites astreoides	            0,70	         0,24	   4,32	   0,82	    4,98	79,71

Groups Chichiriviche  &  Morrocoy

Average dissimilarity = 90,51

	Group Chichiriviche	Group Morrocoy	       	       	        	     

Species	           Av.Abund	      Av.Abund	Av.Diss	Diss/SD	Contrib%	Cum.%

Orbicella faveolata	               0,06	          2,89	  16,99	   1,53	   18,78	18,78

Pseudodiploria strigosa	               2,36	          0,37	  15,00	   1,51	   16,58	35,35

Siderastrea siderea	               1,30	          0,11	   8,52	   1,60	    9,42	44,77

Colpophyllia natans	               0,09	          1,45	   8,15	   1,04	    9,01	53,78

Meandrina meandrites	               0,83	          0,00	   5,27	   0,72	    5,82	59,60

Porites astreoides	               0,26	          0,73	   5,26	   0,82	    5,81	65,41

Montastrea cavernosa	               0,76	          0,12	   5,07	   0,91	    5,60	71,01

Diploria labyrinthiformis	               0,75	          0,00	   4,71	   0,97	    5,21	76,21

Groups Cubagua  &  Morrocoy

Average dissimilarity = 88,90

	Group Cubagua	Group Morrocoy	       	       	        	     

Species	     Av.Abund	      Av.Abund	Av.Diss	Diss/SD	Contrib%	Cum.%

Orbicella faveolata	         0,12	          2,89	  24,90	   1,47	   28,01	28,01

Pseudodiploria strigosa	         1,46	          0,37	  14,17	   0,88	   15,94	43,95

Colpophyllia natans	         0,83	          1,45	  14,17	   0,97	   15,93	59,89

Porites astreoides	         0,23	          0,73	   8,57	   0,74	    9,64	69,53

Agaricia humilis	         0,00	          0,36	   3,93	   0,68	    4,42	73,95

Agaricia sp.	         0,00	          0,43	   3,06	   0,64	    3,45	77,40

Groups Los Frailes  &  Morrocoy

Average dissimilarity = 91,23

	Group Los Frailes	Group Morrocoy	       	       	        	     

Species	         Av.Abund	      Av.Abund	Av.Diss	Diss/SD	Contrib%	Cum.%

Orbicella faveolata	             0,00	          2,89	  25,78	   1,48	   28,26	28,26

Pseudodiploria strigosa	             1,91	          0,37	  18,62	   1,03	   20,42	48,68

Colpophyllia natans	             0,00	          1,45	  11,51	   0,97	   12,62	61,29

Porites astreoides	             0,56	          0,73	   9,15	   0,80	   10,03	71,32

Agaricia humilis	             0,00	          0,36	   4,03	   0,66	    4,42	75,74

Groups Los Roques  &  Morrocoy

Average dissimilarity = 71,46

	Group Los Roques	Group Morrocoy	       	       	        	     

Species	        Av.Abund	      Av.Abund	Av.Diss	Diss/SD	Contrib%	Cum.%

Orbicella faveolata	            2,80	          2,89	  11,50	   1,19	   16,10	16,10

Colpophyllia natans	            1,14	          1,45	   7,61	   1,17	   10,65	26,74

Orbicella annularis	            1,31	          0,27	   6,34	   0,93	    8,87	35,62

Acropora cervicornis	            0,70	          0,00	   5,63	   0,38	    7,88	43,50

Porites astreoides	            0,70	          0,73	   4,87	   0,98	    6,81	50,31

Madracis mirabilis	            1,00	          0,04	   4,54	   0,64	    6,36	56,67

Siderastrea siderea	            0,79	          0,11	   4,10	   0,72	    5,74	62,40

Montastrea cavernosa	            0,59	          0,12	   3,16	   0,64	    4,42	66,82

Agaricia sp.	            0,31	          0,43	   2,95	   0,70	    4,13	70,95

Pseudodiploria strigosa	            0,41	          0,37	   2,92	   0,82	    4,09	75,04

Groups Mochima  &  Morrocoy

Average dissimilarity = 87,31

	Group Mochima	Group Morrocoy	       	       	        	     

Species	     Av.Abund	      Av.Abund	Av.Diss	Diss/SD	Contrib%	Cum.%

Orbicella faveolata	         0,09	          2,89	  22,09	   1,28	   25,31	25,31

Colpophyllia natans	         1,69	          1,45	  13,56	   1,09	   15,54	40,84

Pseudodiploria strigosa	         1,47	          0,37	   9,82	   1,04	   11,25	52,10

Montastrea cavernosa	         1,07	          0,12	   7,60	   0,80	    8,70	60,80

Porites astreoides	         0,24	          0,73	   7,10	   0,66	    8,13	68,93

Agaricia humilis	         0,00	          0,36	   3,46	   0,61	    3,96	72,89

Siderastrea siderea	         0,38	          0,11	   3,27	   0,55	    3,75	76,64

Groups Chichiriviche  &  Ocumare

Average dissimilarity = 75,72

	Group Chichiriviche	Group Ocumare	       	       	        	     

Species	           Av.Abund	     Av.Abund	Av.Diss	Diss/SD	Contrib%	Cum.%

Orbicella faveolata	               0,06	         2,41	  13,83	   1,60	   18,27	18,27

Pseudodiploria strigosa	               2,36	         0,82	  10,65	   1,78	   14,06	32,33

Colpophyllia natans	               0,09	         1,57	   8,86	   1,00	   11,70	44,03

Siderastrea siderea	               1,30	         0,78	   7,69	   1,54	   10,15	54,18

Montastrea cavernosa	               0,76	         1,16	   6,37	   1,20	    8,41	62,59

Porites astreoides	               0,26	         1,14	   6,28	   1,33	    8,30	70,89

Meandrina meandrites	               0,83	         0,04	   4,84	   0,79	    6,39	77,28

Groups Cubagua  &  Ocumare

Average dissimilarity = 83,40

	Group Cubagua	Group Ocumare	       	       	        	     

Species	     Av.Abund	     Av.Abund	Av.Diss	Diss/SD	Contrib%	Cum.%

Orbicella faveolata	         0,12	         2,41	  19,07	   1,54	   22,86	22,86

Colpophyllia natans	         0,83	         1,57	  13,44	   1,04	   16,12	38,98

Pseudodiploria strigosa	         1,46	         0,82	  12,05	   1,30	   14,45	53,43

Montastrea cavernosa	         0,00	         1,16	   9,86	   1,06	   11,82	65,25

Porites astreoides	         0,23	         1,14	   9,56	   1,43	   11,47	76,72

Groups Los Frailes  &  Ocumare

Average dissimilarity = 84,07

	Group Los Frailes	Group Ocumare	       	       	        	     

Species	         Av.Abund	     Av.Abund	Av.Diss	Diss/SD	Contrib%	Cum.%

Orbicella faveolata	             0,00	         2,41	  19,69	   1,59	   23,42	23,42

Pseudodiploria strigosa	             1,91	         0,82	  14,22	   1,39	   16,92	40,34

Colpophyllia natans	             0,00	         1,57	  12,40	   0,93	   14,75	55,08

Montastrea cavernosa	             0,00	         1,16	   9,99	   1,05	   11,89	66,97

Porites astreoides	             0,56	         1,14	   8,16	   1,18	    9,71	76,68

Groups Los Roques  &  Ocumare

Average dissimilarity = 66,76

	Group Los Roques	Group Ocumare	       	       	        	     

Species	        Av.Abund	     Av.Abund	Av.Diss	Diss/SD	Contrib%	Cum.%

Orbicella faveolata	            2,80	         2,41	   9,06	   1,11	   13,56	13,56

Colpophyllia natans	            1,14	         1,57	   7,56	   1,16	   11,32	24,89

Orbicella annularis	            1,31	         0,34	   6,20	   0,93	    9,28	34,17

Montastrea cavernosa	            0,59	         1,16	   5,54	   1,19	    8,30	42,47

Siderastrea siderea	            0,79	         0,78	   5,48	   0,91	    8,20	50,67

Porites astreoides	            0,70	         1,14	   5,12	   1,39	    7,67	58,35

Acropora cervicornis	            0,70	         0,00	   4,92	   0,41	    7,37	65,72

Madracis mirabilis	            1,00	         0,07	   4,38	   0,66	    6,55	72,27

Pseudodiploria strigosa	            0,41	         0,82	   4,15	   1,16	    6,21	78,48

Groups Mochima  &  Ocumare

Average dissimilarity = 76,42

	Group Mochima	Group Ocumare	       	       	        	     

Species	     Av.Abund	     Av.Abund	Av.Diss	Diss/SD	Contrib%	Cum.%

Orbicella faveolata	         0,09	         2,41	  16,88	   1,39	   22,09	22,09

Colpophyllia natans	         1,69	         1,57	  12,84	   1,08	   16,81	38,90

Pseudodiploria strigosa	         1,47	         0,82	   9,79	   1,30	   12,81	51,70

Montastrea cavernosa	         1,07	         1,16	   8,88	   1,04	   11,62	63,33

Porites astreoides	         0,24	         1,14	   7,87	   1,16	   10,30	73,63

Siderastrea siderea	         0,38	         0,78	   7,10	   0,69	    9,30	82,92

Groups Morrocoy  &  Ocumare

Average dissimilarity = 66,70

	Group Morrocoy	Group Ocumare	       	       	        	     

Species	      Av.Abund	     Av.Abund	Av.Diss	Diss/SD	Contrib%	Cum.%

Orbicella faveolata	          2,89	         2,41	  13,08	   1,26	   19,62	19,62

Colpophyllia natans	          1,45	         1,57	  10,09	   1,11	   15,13	34,74

Montastrea cavernosa	          0,12	         1,16	   6,94	   1,04	   10,41	45,15

Porites astreoides	          0,73	         1,14	   6,10	   1,13	    9,15	54,30

Siderastrea siderea	          0,11	         0,78	   5,27	   0,65	    7,91	62,21

Pseudodiploria strigosa	          0,37	         0,82	   4,99	   0,94	    7,48	69,68

Orbicella annularis	          0,27	         0,34	   3,21	   0,53	    4,81	74,49

Agaricia humilis	          0,36	         0,13	   2,28	   0,83	    3,42	77,91
