# Supplementary material for: On the importance of spatial scales on beta diversity of coral assemblages: a study from Venezuelan coral reefs
Source: PeerJ. 2020 May 4;8:e9082. doi: 10.7717/peerj.9082 (PMC7204821; doi:10.7717/peerj.9082)
Supplement: Supplementary file 6 [file peerj-08-9082-s006.odt]

Groups La Pared  &  Media Legua

Average dissimilarity = 52,37

	Group La Pared	Group Media Legua	       	       	        	     

Species	      Av.Abund	         Av.Abund	Av.Diss	Diss/SD	Contrib%	Cum.%

Meandrina meandrites	          1,94	             0,60	  10,43	   1,32	   19,92	19,92

Montastrea cavernosa	          1,38	             0,45	   7,21	   1,61	   13,78	33,69

Diploria labyrinthiformis	          0,72	             0,17	   4,71	   1,05	    8,99	42,68

Pseudodiploria strigosa	          2,33	             2,20	   4,42	   1,20	    8,44	51,13

Other (Scleractinian massive)	          0,62	             0,17	   4,22	   1,02	    8,06	59,19

Siderastrea siderea	          1,37	             0,85	   4,13	   1,25	    7,89	67,08

Porites astreoides	          0,45	             0,17	   2,74	   1,01	    5,24	72,32

Isophyllia sp.	          0,35	             0,00	   2,19	   0,56	    4,19	76,50

Groups La Pared  &  Punta de Media Legua

Average dissimilarity = 56,78

	Group La Pared	Group Punta de Media Legua	       	       	        	     

Species	      Av.Abund	                  Av.Abund	Av.Diss	Diss/SD	Contrib%	Cum.%

Meandrina meandrites	          1,94	                      0,27	  10,78	   1,41	   18,98	18,98

Pseudodiploria strigosa	          2,33	                      1,63	   7,68	   1,02	   13,53	32,51

Montastrea cavernosa	          1,38	                      1,14	   5,93	   1,08	   10,44	42,96

Diploria labyrinthiformis	          0,72	                      0,83	   5,89	   1,20	   10,38	53,34

Siderastrea siderea	          1,37	                      1,59	   5,23	   2,91	    9,21	62,55

Other (Scleractinian massive)	          0,62	                      0,17	   4,09	   1,00	    7,19	69,74

Porites astreoides	          0,45	                      0,27	   2,88	   0,98	    5,07	74,81

Isophyllia sp.	          0,35	                      0,00	   2,12	   0,54	    3,74	78,55

Groups Media Legua  &  Punta de Media Legua

Average dissimilarity = 55,11

	Group Media Legua	Group Punta de Media Legua	       	       	        	     

Species	         Av.Abund	                  Av.Abund	Av.Diss	Diss/SD	Contrib%	Cum.%

Pseudodiploria strigosa	             2,20	                      1,63	  10,32	   0,97	   18,73	18,73

Siderastrea siderea	             0,85	                      1,59	   8,14	   1,77	   14,77	33,50

Montastrea cavernosa	             0,45	                      1,14	   8,03	   1,32	   14,57	48,07

Meandrina meandrites	             0,60	                      0,27	   6,66	   0,64	   12,08	60,16

Diploria labyrinthiformis	             0,17	                      0,83	   5,42	   0,89	    9,84	70,00

Other (Scleractinian massive)	             0,17	                      0,17	   2,63	   0,70	    4,78	74,77

Porites astreoides	             0,17	                      0,27	   2,55	   0,79	    4,63	79,40

Groups La Pared  &  Petaquire

Average dissimilarity = 55,59

	Group La Pared	Group Petaquire	       	       	        	     

Species	      Av.Abund	       Av.Abund	Av.Diss	Diss/SD	Contrib%	Cum.%

Meandrina meandrites	          1,94	           0,00	  11,63	   1,50	   20,92	20,92

Montastrea cavernosa	          1,38	           0,23	   7,67	   1,57	   13,81	34,72

Pseudodiploria strigosa	          2,33	           2,75	   5,00	   1,44	    8,99	43,71

Diploria labyrinthiformis	          0,72	           0,23	   4,65	   1,07	    8,36	52,07

Other (Scleractinian massive)	          0,62	           0,23	   4,17	   1,01	    7,51	59,58

Siderastrea siderea	          1,37	           0,92	   3,32	   1,39	    5,97	65,55

Porites astreoides	          0,45	           0,00	   2,66	   0,91	    4,79	70,34

Siderastrea sp.	          0,17	           0,37	   2,57	   0,85	    4,63	74,97

Isophyllia sp.	          0,35	           0,00	   2,17	   0,55	    3,90	78,88

Groups Media Legua  &  Petaquire

Average dissimilarity = 42,25

	Group Media Legua	Group Petaquire	       	       	        	     

Species	         Av.Abund	       Av.Abund	Av.Diss	Diss/SD	Contrib%	Cum.%

Pseudodiploria strigosa	             2,20	           2,75	   6,33	   1,27	   14,97	14,97

Meandrina meandrites	             0,60	           0,00	   5,74	   0,54	   13,60	28,57

Siderastrea siderea	             0,85	           0,92	   5,04	   1,43	   11,93	40,50

Montastrea cavernosa	             0,45	           0,23	   4,06	   1,00	    9,61	50,11

Siderastrea sp.	             0,00	           0,37	   3,18	   0,67	    7,54	57,64

Other (Scleractinian massive)	             0,17	           0,23	   2,64	   0,79	    6,25	63,90

Diploria labyrinthiformis	             0,17	           0,23	   2,56	   0,80	    6,07	69,96

Agaricia sp.	             0,17	           0,23	   2,53	   0,78	    5,98	75,94

Groups Punta de Media Legua  &  Petaquire

Average dissimilarity = 55,01

	Group Punta de Media Legua	Group Petaquire	       	       	        	     

Species	                  Av.Abund	       Av.Abund	Av.Diss	Diss/SD	Contrib%	Cum.%

Pseudodiploria strigosa	                      1,63	           2,75	  13,43	   1,06	   24,41	24,41

Montastrea cavernosa	                      1,14	           0,23	   8,55	   1,31	   15,54	39,95

Siderastrea siderea	                      1,59	           0,92	   5,99	   1,15	   10,88	50,83

Diploria labyrinthiformis	                      0,83	           0,23	   5,55	   0,93	   10,09	60,93

Siderastrea sp.	                      0,00	           0,37	   3,13	   0,63	    5,69	66,61

Other (Scleractinian massive)	                      0,17	           0,23	   2,79	   0,72	    5,07	71,69

Colpophyllia natans	                      0,17	           0,23	   2,15	   0,77	    3,90	75,59

Groups La Pared  &  Playa Tiburon

Average dissimilarity = 43,05

	Group La Pared	Group Playa Tiburon	       	       	        	     

Species	      Av.Abund	           Av.Abund	Av.Diss	Diss/SD	Contrib%	Cum.%

Meandrina meandrites	          1,94	               0,75	   8,44	   1,39	   19,60	19,60

Montastrea cavernosa	          1,38	               0,93	   5,28	   1,42	   12,26	31,86

Diploria labyrinthiformis	          0,72	               1,38	   4,86	   1,53	   11,29	43,15

Pseudodiploria strigosa	          2,33	               2,38	   4,02	   1,40	    9,34	52,49

Other (Scleractinian massive)	          0,62	               0,17	   3,47	   1,09	    8,07	60,56

Porites astreoides	          0,45	               0,40	   3,21	   1,07	    7,45	68,01

Agaricia humilis	          0,35	               0,45	   2,95	   1,07	    6,85	74,86

Siderastrea siderea	          1,37	               1,64	   2,80	   1,06	    6,51	81,37

Groups Media Legua  &  Playa Tiburon

Average dissimilarity = 47,64

	Group Media Legua	Group Playa Tiburon	       	       	        	     

Species	         Av.Abund	           Av.Abund	Av.Diss	Diss/SD	Contrib%	Cum.%

Diploria labyrinthiformis	             0,17	               1,38	   8,77	   2,03	   18,41	18,41

Meandrina meandrites	             0,60	               0,75	   7,00	   0,92	   14,70	33,11

Siderastrea siderea	             0,85	               1,64	   6,64	   1,20	   13,94	47,05

Montastrea cavernosa	             0,45	               0,93	   5,57	   1,21	   11,70	58,75

Pseudodiploria strigosa	             2,20	               2,38	   4,72	   1,45	    9,92	68,67

Porites astreoides	             0,17	               0,40	   3,29	   0,75	    6,91	75,58

Groups Punta de Media Legua  &  Playa Tiburon

Average dissimilarity = 51,11

	Group Punta de Media Legua	Group Playa Tiburon	       	       	        	     

Species	                  Av.Abund	           Av.Abund	Av.Diss	Diss/SD	Contrib%	Cum.%

Pseudodiploria strigosa	                      1,63	               2,38	   8,76	   1,07	   17,15	17,15

Diploria labyrinthiformis	                      0,83	               1,38	   8,59	   1,65	   16,81	33,96

Siderastrea siderea	                      1,59	               1,64	   6,89	   1,46	   13,49	47,45

Montastrea cavernosa	                      1,14	               0,93	   6,11	   1,23	   11,96	59,40

Meandrina meandrites	                      0,27	               0,75	   5,29	   0,88	   10,36	69,76

Porites astreoides	                      0,27	               0,40	   3,30	   0,71	    6,45	76,21

Groups Petaquire  &  Playa Tiburon

Average dissimilarity = 47,75

	Group Petaquire	Group Playa Tiburon	       	       	        	     

Species	       Av.Abund	           Av.Abund	Av.Diss	Diss/SD	Contrib%	Cum.%

Diploria labyrinthiformis	           0,23	               1,38	   8,26	   1,76	   17,29	17,29

Siderastrea siderea	           0,92	               1,64	   5,99	   1,22	   12,55	29,84

Montastrea cavernosa	           0,23	               0,93	   5,98	   1,19	   12,52	42,35

Pseudodiploria strigosa	           2,75	               2,38	   5,25	   1,59	   10,99	53,34

Meandrina meandrites	           0,00	               0,75	   5,04	   0,78	   10,56	63,90

Agaricia humilis	           0,00	               0,45	   3,08	   0,91	    6,45	70,36

Porites astreoides	           0,00	               0,40	   2,63	   0,55	    5,51	75,86

Groups La Pared  &  Punta Mono

Average dissimilarity = 44,34

	Group La Pared	Group Punta Mono	       	       	        	     

Species	      Av.Abund	        Av.Abund	Av.Diss	Diss/SD	Contrib%	Cum.%

Meandrina meandrites	          1,94	            1,21	   7,40	   1,57	   16,68	16,68

Montastrea cavernosa	          1,38	            0,27	   6,62	   1,46	   14,94	31,62

Pseudodiploria strigosa	          2,33	            2,96	   5,01	   1,41	   11,29	42,91

Diploria labyrinthiformis	          0,72	            1,02	   4,17	   1,90	    9,40	52,30

Other (Scleractinian massive)	          0,62	            0,17	   3,60	   0,99	    8,11	60,41

Porites astreoides	          0,45	            0,17	   2,37	   1,00	    5,34	65,75

Agaricia agaricites (agaricites)	          0,27	            0,17	   2,15	   0,73	    4,84	70,59

Siderastrea siderea	          1,37	            1,34	   2,07	   1,52	    4,66	75,25

Groups Media Legua  &  Punta Mono

Average dissimilarity = 46,26

	Group Media Legua	Group Punta Mono	       	       	        	     

Species	         Av.Abund	        Av.Abund	Av.Diss	Diss/SD	Contrib%	Cum.%

Meandrina meandrites	             0,60	            1,21	   9,30	   1,62	   20,11	20,11

Pseudodiploria strigosa	             2,20	            2,96	   7,02	   1,82	   15,18	35,29

Diploria labyrinthiformis	             0,17	            1,02	   6,44	   1,83	   13,92	49,21

Siderastrea siderea	             0,85	            1,34	   5,02	   1,24	   10,86	60,07

Montastrea cavernosa	             0,45	            0,27	   3,66	   1,00	    7,92	67,99

Other (Scleractinian flower cup)	             0,00	            0,27	   2,08	   0,56	    4,49	72,48

Pseudodiploria clivosa	             0,17	            0,17	   2,02	   0,72	    4,36	76,84

Groups Punta de Media Legua  &  Punta Mono

Average dissimilarity = 55,01

	Group Punta de Media Legua	Group Punta Mono	       	       	        	     

Species	                  Av.Abund	        Av.Abund	Av.Diss	Diss/SD	Contrib%	Cum.%

Pseudodiploria strigosa	                      1,63	            2,96	  11,94	   1,24	   21,71	21,71

Meandrina meandrites	                      0,27	            1,21	   8,27	   1,26	   15,03	36,73

Diploria labyrinthiformis	                      0,83	            1,02	   7,24	   1,74	   13,16	49,90

Montastrea cavernosa	                      1,14	            0,27	   6,94	   1,24	   12,61	62,51

Siderastrea siderea	                      1,59	            1,34	   6,04	   1,85	   10,97	73,48

Porites astreoides	                      0,27	            0,17	   2,11	   0,79	    3,84	77,32

Groups Petaquire  &  Punta Mono

Average dissimilarity = 45,92

	Group Petaquire	Group Punta Mono	       	       	        	     

Species	       Av.Abund	        Av.Abund	Av.Diss	Diss/SD	Contrib%	Cum.%

Meandrina meandrites	           0,00	            1,21	   9,28	   1,55	   20,22	20,22

Diploria labyrinthiformis	           0,23	            1,02	   5,93	   1,53	   12,92	33,13

Pseudodiploria strigosa	           2,75	            2,96	   5,73	   1,57	   12,49	45,62

Siderastrea siderea	           0,92	            1,34	   4,22	   1,47	    9,18	54,80

Montastrea cavernosa	           0,23	            0,27	   2,74	   0,86	    5,96	60,76

Siderastrea sp.	           0,37	            0,00	   2,61	   0,66	    5,69	66,46

Other (Scleractinian massive)	           0,23	            0,17	   2,07	   0,79	    4,51	70,96

Other (Scleractinian flower cup)	           0,00	            0,27	   2,06	   0,54	    4,49	75,45

Groups Playa Tiburon  &  Punta Mono

Average dissimilarity = 38,72

	Group Playa Tiburon	Group Punta Mono	       	       	        	     

Species	           Av.Abund	        Av.Abund	Av.Diss	Diss/SD	Contrib%	Cum.%

Meandrina meandrites	               0,75	            1,21	   6,77	   1,56	   17,49	17,49

Montastrea cavernosa	               0,93	            0,27	   5,24	   1,21	   13,54	31,03

Pseudodiploria strigosa	               2,38	            2,96	   5,14	   1,41	   13,27	44,30

Siderastrea siderea	               1,64	            1,34	   3,63	   0,99	    9,37	53,67

Diploria labyrinthiformis	               1,38	            1,02	   3,60	   1,26	    9,29	62,96

Porites astreoides	               0,40	            0,17	   2,79	   0,74	    7,22	70,18

Agaricia humilis	               0,45	            0,00	   2,64	   0,93	    6,82	77,00

Groups La Pared  &  Charagato

Average dissimilarity = 92,25

	Group La Pared	Group Charagato	       	       	        	     

Species	      Av.Abund	       Av.Abund	Av.Diss	Diss/SD	Contrib%	Cum.%

Pseudodiploria strigosa	          2,33	           0,40	  16,64	   1,75	   18,04	18,04

Meandrina meandrites	          1,94	           0,00	  14,23	   1,48	   15,43	33,47

Colpophyllia natans	          0,00	           1,86	  13,06	   1,45	   14,16	47,62

Montastrea cavernosa	          1,38	           0,00	  10,08	   1,49	   10,93	58,55

Siderastrea siderea	          1,37	           0,27	   9,34	   1,75	   10,12	68,67

Diploria labyrinthiformis	          0,72	           0,00	   5,94	   0,93	    6,44	75,12

Groups Media Legua  &  Charagato

Average dissimilarity = 87,17

	Group Media Legua	Group Charagato	       	       	        	     

Species	         Av.Abund	       Av.Abund	Av.Diss	Diss/SD	Contrib%	Cum.%

Pseudodiploria strigosa	             2,20	           0,40	  26,72	   1,73	   30,66	30,66

Colpophyllia natans	             0,17	           1,86	  19,87	   1,59	   22,79	53,44

Siderastrea siderea	             0,85	           0,27	  10,76	   1,18	   12,34	65,78

Meandrina meandrites	             0,60	           0,00	   8,24	   0,53	    9,46	75,24

Groups Punta de Media Legua  &  Charagato

Average dissimilarity = 90,36

	Group Punta de Media Legua	Group Charagato	       	       	        	     

Species	                  Av.Abund	       Av.Abund	Av.Diss	Diss/SD	Contrib%	Cum.%

Colpophyllia natans	                      0,17	           1,86	  20,52	   1,20	   22,71	22,71

Pseudodiploria strigosa	                      1,63	           0,40	  18,19	   1,06	   20,13	42,84

Siderastrea siderea	                      1,59	           0,27	  16,12	   2,08	   17,84	60,68

Montastrea cavernosa	                      1,14	           0,00	  12,84	   1,13	   14,21	74,89

Diploria labyrinthiformis	                      0,83	           0,00	   6,07	   0,77	    6,72	81,60

Groups Petaquire  &  Charagato

Average dissimilarity = 86,52

	Group Petaquire	Group Charagato	       	       	        	     

Species	       Av.Abund	       Av.Abund	Av.Diss	Diss/SD	Contrib%	Cum.%

Pseudodiploria strigosa	           2,75	           0,40	  33,71	   1,73	   38,97	38,97

Colpophyllia natans	           0,23	           1,86	  19,85	   1,38	   22,94	61,90

Siderastrea siderea	           0,92	           0,27	  11,03	   1,77	   12,75	74,66

Siderastrea sp.	           0,37	           0,00	   4,34	   0,65	    5,01	79,67

Groups Playa Tiburon  &  Charagato

Average dissimilarity = 90,75

	Group Playa Tiburon	Group Charagato	       	       	        	     

Species	           Av.Abund	       Av.Abund	Av.Diss	Diss/SD	Contrib%	Cum.%

Pseudodiploria strigosa	               2,38	           0,40	  19,75	   1,85	   21,76	21,76

Colpophyllia natans	               0,00	           1,86	  15,31	   1,45	   16,87	38,63

Siderastrea siderea	               1,64	           0,27	  13,69	   1,54	   15,08	53,72

Diploria labyrinthiformis	               1,38	           0,00	  12,69	   2,62	   13,99	67,70

Montastrea cavernosa	               0,93	           0,00	   8,80	   1,27	    9,70	77,40

Groups Punta Mono  &  Charagato

Average dissimilarity = 90,41

	Group Punta Mono	Group Charagato	       	       	        	     

Species	        Av.Abund	       Av.Abund	Av.Diss	Diss/SD	Contrib%	Cum.%

Pseudodiploria strigosa	            2,96	           0,40	  25,83	   2,35	   28,57	28,57

Colpophyllia natans	            0,00	           1,86	  16,07	   1,42	   17,77	46,34

Meandrina meandrites	            1,21	           0,00	  12,36	   1,52	   13,67	60,01

Siderastrea siderea	            1,34	           0,27	  11,34	   1,57	   12,55	72,56

Diploria labyrinthiformis	            1,02	           0,00	   9,96	   2,79	   11,02	83,58

Groups La Pared  &  Punta Conejo

Average dissimilarity = 74,61

	Group La Pared	Group Punta Conejo	       	       	        	     

Species	      Av.Abund	          Av.Abund	Av.Diss	Diss/SD	Contrib%	Cum.%

Meandrina meandrites	          1,94	              0,00	  14,15	   1,50	   18,96	18,96

Pseudodiploria strigosa	          2,33	              1,99	  11,82	   1,33	   15,84	34,80

Montastrea cavernosa	          1,38	              0,00	  10,02	   1,51	   13,43	48,23

Siderastrea siderea	          1,37	              0,17	   9,76	   2,34	   13,09	61,31

Diploria labyrinthiformis	          0,72	              0,00	   5,89	   0,94	    7,90	69,21

Other (Scleractinian massive)	          0,62	              0,17	   5,29	   0,95	    7,09	76,30

Groups Media Legua  &  Punta Conejo

Average dissimilarity = 64,40

	Group Media Legua	Group Punta Conejo	       	       	        	     

Species	         Av.Abund	          Av.Abund	Av.Diss	Diss/SD	Contrib%	Cum.%

Pseudodiploria strigosa	             2,20	              1,99	  19,98	   1,57	   31,03	31,03

Siderastrea siderea	             0,85	              0,17	  10,39	   1,23	   16,14	47,17

Meandrina meandrites	             0,60	              0,00	   8,07	   0,54	   12,53	59,70

Montastrea cavernosa	             0,45	              0,00	   5,39	   0,91	    8,37	68,07

Other (Scleractinian massive)	             0,17	              0,17	   3,23	   0,72	    5,01	73,07

Oculina diffusa	             0,00	              0,17	   2,96	   0,55	    4,60	77,68

Groups Punta de Media Legua  &  Punta Conejo

Average dissimilarity = 74,36

	Group Punta de Media Legua	Group Punta Conejo	       	       	        	     

Species	                  Av.Abund	          Av.Abund	Av.Diss	Diss/SD	Contrib%	Cum.%

Pseudodiploria strigosa	                      1,63	              1,99	  20,38	   1,29	   27,41	27,41

Siderastrea siderea	                      1,59	              0,17	  15,12	   2,00	   20,33	47,73

Montastrea cavernosa	                      1,14	              0,00	  12,54	   1,18	   16,87	64,60

Diploria labyrinthiformis	                      0,83	              0,00	   6,03	   0,78	    8,11	72,71

Other (Scleractinian massive)	                      0,17	              0,17	   3,83	   0,65	    5,14	77,85

Groups Petaquire  &  Punta Conejo

Average dissimilarity = 59,39

	Group Petaquire	Group Punta Conejo	       	       	        	     

Species	       Av.Abund	          Av.Abund	Av.Diss	Diss/SD	Contrib%	Cum.%

Pseudodiploria strigosa	           2,75	              1,99	  22,21	   1,24	   37,40	37,40

Siderastrea siderea	           0,92	              0,17	  10,13	   1,61	   17,05	54,45

Siderastrea sp.	           0,37	              0,00	   4,27	   0,66	    7,19	61,64

Other (Scleractinian massive)	           0,23	              0,17	   3,46	   0,78	    5,82	67,46

Oculina diffusa	           0,00	              0,17	   3,08	   0,51	    5,19	72,65

Diploria labyrinthiformis	           0,23	              0,00	   2,69	   0,66	    4,53	77,19

Groups Playa Tiburon  &  Punta Conejo

Average dissimilarity = 71,41

	Group Playa Tiburon	Group Punta Conejo	       	       	        	     

Species	           Av.Abund	          Av.Abund	Av.Diss	Diss/SD	Contrib%	Cum.%

Pseudodiploria strigosa	               2,38	              1,99	  14,20	   1,48	   19,88	19,88

Siderastrea siderea	               1,64	              0,17	  14,08	   1,84	   19,71	39,60

Diploria labyrinthiformis	               1,38	              0,00	  12,56	   2,74	   17,59	57,19

Montastrea cavernosa	               0,93	              0,00	   8,70	   1,29	   12,19	69,37

Meandrina meandrites	               0,75	              0,00	   6,31	   0,79	    8,84	78,21

Groups Punta Mono  &  Punta Conejo

Average dissimilarity = 66,76

	Group Punta Mono	Group Punta Conejo	       	       	        	     

Species	        Av.Abund	          Av.Abund	Av.Diss	Diss/SD	Contrib%	Cum.%

Pseudodiploria strigosa	            2,96	              1,99	  16,30	   1,25	   24,41	24,41

Meandrina meandrites	            1,21	              0,00	  12,19	   1,56	   18,25	42,67

Siderastrea siderea	            1,34	              0,17	  11,75	   2,03	   17,60	60,27

Diploria labyrinthiformis	            1,02	              0,00	   9,84	   2,96	   14,73	75,00

Groups Charagato  &  Punta Conejo

Average dissimilarity = 90,55

	Group Charagato	Group Punta Conejo	       	       	        	     

Species	       Av.Abund	          Av.Abund	Av.Diss	Diss/SD	Contrib%	Cum.%

Pseudodiploria strigosa	           0,40	              1,99	  36,79	   1,20	   40,63	40,63

Colpophyllia natans	           1,86	              0,00	  32,59	   1,34	   35,99	76,62

Groups La Pared  &  La Muerta

Average dissimilarity = 77,25

	Group La Pared	Group La Muerta	       	       	        	     

Species	      Av.Abund	       Av.Abund	Av.Diss	Diss/SD	Contrib%	Cum.%

Meandrina meandrites	          1,94	           0,00	  13,59	   1,46	   17,59	17,59

Siderastrea siderea	          1,37	           0,00	  10,45	   3,16	   13,53	31,12

Montastrea cavernosa	          1,38	           0,00	   9,63	   1,47	   12,46	43,58

Pseudodiploria strigosa	          2,33	           1,99	   9,54	   0,97	   12,34	55,93

Diploria labyrinthiformis	          0,72	           0,00	   5,65	   0,92	    7,31	63,24

Other (Scleractinian massive)	          0,62	           0,00	   5,13	   0,86	    6,64	69,88

Porites astreoides	          0,45	           0,52	   4,92	   1,07	    6,37	76,25

Groups Media Legua  &  La Muerta

Average dissimilarity = 65,09

	Group Media Legua	Group La Muerta	       	       	        	     

Species	         Av.Abund	       Av.Abund	Av.Diss	Diss/SD	Contrib%	Cum.%

Pseudodiploria strigosa	             2,20	           1,99	  15,51	   0,89	   23,83	23,83

Siderastrea siderea	             0,85	           0,00	  10,74	   1,25	   16,50	40,33

Meandrina meandrites	             0,60	           0,00	   7,68	   0,52	   11,81	52,14

Colpophyllia natans	             0,17	           0,62	   7,37	   0,74	   11,32	63,46

Porites astreoides	             0,17	           0,52	   5,52	   0,76	    8,47	71,93

Montastrea cavernosa	             0,45	           0,00	   5,13	   0,88	    7,89	79,82

Groups Punta de Media Legua  &  La Muerta

Average dissimilarity = 78,26

	Group Punta de Media Legua	Group La Muerta	       	       	        	     

Species	                  Av.Abund	       Av.Abund	Av.Diss	Diss/SD	Contrib%	Cum.%

Pseudodiploria strigosa	                      1,63	           1,99	  17,97	   0,99	   22,96	22,96

Siderastrea siderea	                      1,59	           0,00	  16,25	   2,34	   20,77	43,73

Montastrea cavernosa	                      1,14	           0,00	  12,03	   1,10	   15,37	59,10

Colpophyllia natans	                      0,17	           0,62	   6,64	   0,60	    8,48	67,58

Diploria labyrinthiformis	                      0,83	           0,00	   5,79	   0,77	    7,40	74,99

Porites astreoides	                      0,27	           0,52	   5,32	   0,69	    6,80	81,78

Groups Petaquire  &  La Muerta

Average dissimilarity = 61,33

	Group Petaquire	Group La Muerta	       	       	        	     

Species	       Av.Abund	       Av.Abund	Av.Diss	Diss/SD	Contrib%	Cum.%

Pseudodiploria strigosa	           2,75	           1,99	  18,01	   0,77	   29,38	29,38

Siderastrea siderea	           0,92	           0,00	  11,32	   2,17	   18,46	47,83

Colpophyllia natans	           0,23	           0,62	   7,31	   0,72	   11,92	59,75

Porites astreoides	           0,00	           0,52	   4,29	   0,54	    6,99	66,74

Orbicella faveolata	           0,23	           0,35	   4,25	   0,84	    6,93	73,68

Siderastrea sp.	           0,37	           0,00	   4,07	   0,64	    6,63	80,31

Groups Playa Tiburon  &  La Muerta

Average dissimilarity = 73,16

	Group Playa Tiburon	Group La Muerta	       	       	        	     

Species	           Av.Abund	       Av.Abund	Av.Diss	Diss/SD	Contrib%	Cum.%

Siderastrea siderea	               1,64	           0,00	  14,71	   2,10	   20,10	20,10

Diploria labyrinthiformis	               1,38	           0,00	  12,01	   2,46	   16,42	36,52

Pseudodiploria strigosa	               2,38	           1,99	  11,06	   0,95	   15,12	51,65

Montastrea cavernosa	               0,93	           0,00	   8,32	   1,24	   11,37	63,02

Meandrina meandrites	               0,75	           0,00	   6,05	   0,77	    8,27	71,28

Porites astreoides	               0,40	           0,52	   5,38	   0,77	    7,35	78,63

Groups Punta Mono  &  La Muerta

Average dissimilarity = 70,10

	Group Punta Mono	Group La Muerta	       	       	        	     

Species	        Av.Abund	       Av.Abund	Av.Diss	Diss/SD	Contrib%	Cum.%

Pseudodiploria strigosa	            2,96	           1,99	  13,84	   0,96	   19,74	19,74

Siderastrea siderea	            1,34	           0,00	  12,54	   2,55	   17,88	37,62

Meandrina meandrites	            1,21	           0,00	  11,64	   1,47	   16,61	54,23

Diploria labyrinthiformis	            1,02	           0,00	   9,40	   2,56	   13,42	67,64

Colpophyllia natans	            0,00	           0,62	   4,87	   0,55	    6,95	74,59

Porites astreoides	            0,17	           0,52	   4,43	   0,74	    6,31	80,90

Groups Charagato  &  La Muerta

Average dissimilarity = 84,17

	Group Charagato	Group La Muerta	       	       	        	     

Species	       Av.Abund	       Av.Abund	Av.Diss	Diss/SD	Contrib%	Cum.%

Colpophyllia natans	           1,86	           0,62	  33,33	   1,17	   39,61	39,61

Pseudodiploria strigosa	           0,40	           1,99	  32,85	   1,26	   39,03	78,63

Groups Punta Conejo  &  La Muerta

Average dissimilarity = 68,94

	Group Punta Conejo	Group La Muerta	       	       	        	     

Species	          Av.Abund	       Av.Abund	Av.Diss	Diss/SD	Contrib%	Cum.%

Pseudodiploria strigosa	              1,99	           1,99	  35,27	   1,11	   51,17	51,17

Oculina diffusa	              0,17	           0,00	   9,15	   0,36	   13,27	64,43

Colpophyllia natans	              0,00	           0,62	   8,50	   0,54	   12,34	76,77

Groups La Pared  &  Cominoto

Average dissimilarity = 64,66

	Group La Pared	Group Cominoto	       	       	        	     

Species	      Av.Abund	      Av.Abund	Av.Diss	Diss/SD	Contrib%	Cum.%

Meandrina meandrites	          1,94	          0,00	  13,11	   1,55	   20,27	20,27

Siderastrea siderea	          1,37	          0,00	   9,99	   4,66	   15,45	35,72

Montastrea cavernosa	          1,38	          0,00	   9,29	   1,56	   14,36	50,08

Pseudodiploria strigosa	          2,33	          2,96	   5,80	   1,07	    8,97	59,05

Diploria labyrinthiformis	          0,72	          0,00	   5,39	   0,96	    8,33	67,38

Other (Scleractinian massive)	          0,62	          0,00	   4,87	   0,90	    7,52	74,90

Porites astreoides	          0,45	          0,58	   4,46	   1,10	    6,90	81,80

Groups Media Legua  &  Cominoto

Average dissimilarity = 49,13

	Group Media Legua	Group Cominoto	       	       	        	     

Species	         Av.Abund	      Av.Abund	Av.Diss	Diss/SD	Contrib%	Cum.%

Pseudodiploria strigosa	             2,20	          2,96	   9,75	   1,25	   19,84	19,84

Siderastrea siderea	             0,85	          0,00	   9,60	   1,47	   19,54	39,38

Meandrina meandrites	             0,60	          0,00	   6,89	   0,56	   14,03	53,41

Porites astreoides	             0,17	          0,58	   6,44	   0,91	   13,10	66,51

Montastrea cavernosa	             0,45	          0,00	   4,69	   0,95	    9,54	76,06

Groups Punta de Media Legua  &  Cominoto

Average dissimilarity = 68,37

	Group Punta de Media Legua	Group Cominoto	       	       	        	     

Species	                  Av.Abund	      Av.Abund	Av.Diss	Diss/SD	Contrib%	Cum.%

Pseudodiploria strigosa	                      1,63	          2,96	  19,55	   1,07	   28,59	28,59

Siderastrea siderea	                      1,59	          0,00	  14,29	   4,08	   20,89	49,48

Montastrea cavernosa	                      1,14	          0,00	  10,80	   1,34	   15,80	65,28

Porites astreoides	                      0,27	          0,58	   6,81	   0,83	    9,96	75,24

Groups Petaquire  &  Cominoto

Average dissimilarity = 43,03

	Group Petaquire	Group Cominoto	       	       	        	     

Species	       Av.Abund	      Av.Abund	Av.Diss	Diss/SD	Contrib%	Cum.%

Siderastrea siderea	           0,92	          0,00	  10,02	   4,29	   23,28	23,28

Pseudodiploria strigosa	           2,75	          2,96	   7,56	   1,22	   17,57	40,85

Porites astreoides	           0,00	          0,58	   6,42	   0,81	   14,91	55,76

Siderastrea sp.	           0,37	          0,00	   3,74	   0,68	    8,69	64,45

Diploria labyrinthiformis	           0,23	          0,00	   2,36	   0,68	    5,48	69,93

Montastrea cavernosa	           0,23	          0,00	   2,36	   0,68	    5,48	75,41

Groups Playa Tiburon  &  Cominoto

Average dissimilarity = 59,58

	Group Playa Tiburon	Group Cominoto	       	       	        	     

Species	           Av.Abund	      Av.Abund	Av.Diss	Diss/SD	Contrib%	Cum.%

Siderastrea siderea	               1,64	          0,00	  13,83	   2,56	   23,22	23,22

Diploria labyrinthiformis	               1,38	          0,00	  11,35	   3,03	   19,05	42,27

Montastrea cavernosa	               0,93	          0,00	   7,82	   1,34	   13,13	55,39

Pseudodiploria strigosa	               2,38	          2,96	   6,97	   1,30	   11,69	67,09

Meandrina meandrites	               0,75	          0,00	   5,77	   0,80	    9,68	76,76

Groups Punta Mono  &  Cominoto

Average dissimilarity = 53,35

	Group Punta Mono	Group Cominoto	       	       	        	     

Species	        Av.Abund	      Av.Abund	Av.Diss	Diss/SD	Contrib%	Cum.%

Siderastrea siderea	            1,34	          0,00	  11,69	   3,74	   21,92	21,92

Meandrina meandrites	            1,21	          0,00	  10,82	   1,66	   20,29	42,20

Diploria labyrinthiformis	            1,02	          0,00	   8,80	   3,47	   16,49	58,69

Pseudodiploria strigosa	            2,96	          2,96	   6,51	   1,23	   12,21	70,90

Porites astreoides	            0,17	          0,58	   5,05	   0,88	    9,46	80,36

Groups Charagato  &  Cominoto

Average dissimilarity = 91,87

	Group Charagato	Group Cominoto	       	       	        	     

Species	       Av.Abund	      Av.Abund	Av.Diss	Diss/SD	Contrib%	Cum.%

Pseudodiploria strigosa	           0,40	          2,96	  50,39	   1,77	   54,84	54,84

Colpophyllia natans	           1,86	          0,00	  25,58	   1,55	   27,84	82,69

Groups Punta Conejo  &  Cominoto

Average dissimilarity = 49,93

	Group Punta Conejo	Group Cominoto	       	       	        	     

Species	          Av.Abund	      Av.Abund	Av.Diss	Diss/SD	Contrib%	Cum.%

Pseudodiploria strigosa	              1,99	          2,96	  31,72	   1,11	   63,53	63,53

Porites astreoides	              0,17	          0,58	   9,73	   0,84	   19,48	83,01

Groups La Muerta  &  Cominoto

Average dissimilarity = 50,28

	Group La Muerta	Group Cominoto	       	       	        	     

Species	       Av.Abund	      Av.Abund	Av.Diss	Diss/SD	Contrib%	Cum.%

Pseudodiploria strigosa	           1,99	          2,96	  28,09	   0,79	   55,87	55,87

Porites astreoides	           0,52	          0,58	  11,59	   0,95	   23,04	78,92

Groups La Pared  &  La Pecha

Average dissimilarity = 96,81

	Group La Pared	Group La Pecha	       	       	        	     

Species	      Av.Abund	      Av.Abund	Av.Diss	Diss/SD	Contrib%	Cum.%

Pseudodiploria strigosa	          2,33	          0,17	  21,11	   2,23	   21,81	21,81

Meandrina meandrites	          1,94	          0,00	  16,96	   1,54	   17,52	39,33

Siderastrea siderea	          1,37	          0,00	  13,35	   3,92	   13,79	53,12

Montastrea cavernosa	          1,38	          0,00	  12,01	   1,55	   12,40	65,52

Diploria labyrinthiformis	          0,72	          0,00	   7,27	   0,97	    7,51	73,03

Other (Scleractinian massive)	          0,62	          0,00	   6,68	   0,88	    6,90	79,93

Groups Media Legua  &  La Pecha

Average dissimilarity = 94,07

	Group Media Legua	Group La Pecha	       	       	        	     

Species	         Av.Abund	      Av.Abund	Av.Diss	Diss/SD	Contrib%	Cum.%

Pseudodiploria strigosa	             2,20	          0,17	  38,05	   2,94	   40,45	40,45

Siderastrea siderea	             0,85	          0,00	  15,78	   1,46	   16,78	57,22

Meandrina meandrites	             0,60	          0,00	  11,25	   0,56	   11,96	69,18

Montastrea cavernosa	             0,45	          0,00	   7,28	   0,96	    7,74	76,92

Groups Punta de Media Legua  &  La Pecha

Average dissimilarity = 95,32

	Group Punta de Media Legua	Group La Pecha	       	       	        	     

Species	                  Av.Abund	      Av.Abund	Av.Diss	Diss/SD	Contrib%	Cum.%

Pseudodiploria strigosa	                      1,63	          0,17	  26,40	   1,19	   27,70	27,70

Siderastrea siderea	                      1,59	          0,00	  24,05	   4,77	   25,23	52,93

Montastrea cavernosa	                      1,14	          0,00	  17,37	   1,16	   18,22	71,15

Diploria labyrinthiformis	                      0,83	          0,00	   7,23	   0,80	    7,59	78,74

Groups Petaquire  &  La Pecha

Average dissimilarity = 93,83

	Group Petaquire	Group La Pecha	       	       	        	     

Species	       Av.Abund	      Av.Abund	Av.Diss	Diss/SD	Contrib%	Cum.%

Pseudodiploria strigosa	           2,75	          0,17	  48,81	   2,72	   52,02	52,02

Siderastrea siderea	           0,92	          0,00	  16,81	   3,37	   17,92	69,94

Siderastrea sp.	           0,37	          0,00	   5,70	   0,68	    6,07	76,01

Groups Playa Tiburon  &  La Pecha

Average dissimilarity = 96,07

	Group Playa Tiburon	Group La Pecha	       	       	        	     

Species	           Av.Abund	      Av.Abund	Av.Diss	Diss/SD	Contrib%	Cum.%

Pseudodiploria strigosa	               2,38	          0,17	  26,29	   3,20	   27,37	27,37

Siderastrea siderea	               1,64	          0,00	  19,58	   2,50	   20,38	47,74

Diploria labyrinthiformis	               1,38	          0,00	  15,83	   3,22	   16,48	64,22

Montastrea cavernosa	               0,93	          0,00	  11,08	   1,33	   11,53	75,76

Groups Punta Mono  &  La Pecha

Average dissimilarity = 95,77

	Group Punta Mono	Group La Pecha	       	       	        	     

Species	        Av.Abund	      Av.Abund	Av.Diss	Diss/SD	Contrib%	Cum.%

Pseudodiploria strigosa	            2,96	          0,17	  34,27	   6,04	   35,78	35,78

Siderastrea siderea	            1,34	          0,00	  16,96	   3,38	   17,71	53,49

Meandrina meandrites	            1,21	          0,00	  15,88	   1,66	   16,58	70,07

Diploria labyrinthiformis	            1,02	          0,00	  12,65	   3,61	   13,21	83,28

Groups Charagato  &  La Pecha

Average dissimilarity = 98,49

	Group Charagato	Group La Pecha	       	       	        	     

Species	       Av.Abund	      Av.Abund	Av.Diss	Diss/SD	Contrib%	Cum.%

Colpophyllia natans	           1,86	          0,00	  65,53	   2,44	   66,54	66,54

Pseudodiploria strigosa	           0,40	          0,17	  18,17	   0,67	   18,45	84,98

Groups Punta Conejo  &  La Pecha

Average dissimilarity = 91,77

	Group Punta Conejo	Group La Pecha	       	       	        	     

Species	          Av.Abund	      Av.Abund	Av.Diss	Diss/SD	Contrib%	Cum.%

Pseudodiploria strigosa	              1,99	          0,17	  58,94	   1,65	   64,23	64,23

Oculina diffusa	              0,17	          0,00	  21,88	   0,54	   23,84	88,07

Groups La Muerta  &  La Pecha

Average dissimilarity = 92,87

	Group La Muerta	Group La Pecha	       	       	        	     

Species	       Av.Abund	      Av.Abund	Av.Diss	Diss/SD	Contrib%	Cum.%

Pseudodiploria strigosa	           1,99	          0,17	  62,87	   2,38	   67,69	67,69

Colpophyllia natans	           0,62	          0,00	  14,68	   0,64	   15,81	83,50

Groups Cominoto  &  La Pecha

Average dissimilarity = 91,75

	Group Cominoto	Group La Pecha	       	       	        	     

Species	      Av.Abund	      Av.Abund	Av.Diss	Diss/SD	Contrib%	Cum.%

Pseudodiploria strigosa	          2,96	          0,17	  77,06	   3,58	   83,99	83,99

Groups La Pared  &  Puerto Real

Average dissimilarity = 64,77

	Group La Pared	Group Puerto Real	       	       	        	     

Species	      Av.Abund	         Av.Abund	Av.Diss	Diss/SD	Contrib%	Cum.%

Meandrina meandrites	          1,94	             0,00	  12,34	   1,55	   19,05	19,05

Siderastrea siderea	          1,37	             0,00	   9,35	   4,65	   14,44	33,49

Montastrea cavernosa	          1,38	             0,00	   8,75	   1,55	   13,50	46,99

Pseudodiploria strigosa	          2,33	             2,60	   6,66	   1,37	   10,28	57,27

Diploria labyrinthiformis	          0,72	             0,00	   5,03	   0,96	    7,77	65,05

Porites astreoides	          0,45	             1,10	   4,64	   1,27	    7,16	72,21

Other (Scleractinian massive)	          0,62	             0,35	   4,40	   1,22	    6,79	79,00

Groups Media Legua  &  Puerto Real

Average dissimilarity = 54,59

	Group Media Legua	Group Puerto Real	       	       	        	     

Species	         Av.Abund	         Av.Abund	Av.Diss	Diss/SD	Contrib%	Cum.%

Porites astreoides	             0,17	             1,10	   9,84	   2,53	   18,02	18,02

Pseudodiploria strigosa	             2,20	             2,60	   9,07	   1,29	   16,61	34,63

Siderastrea siderea	             0,85	             0,00	   8,70	   1,46	   15,94	50,57

Meandrina meandrites	             0,60	             0,00	   6,25	   0,56	   11,44	62,01

Montastrea cavernosa	             0,45	             0,00	   4,28	   0,94	    7,84	69,86

Other (Scleractinian massive)	             0,17	             0,35	   3,63	   0,95	    6,64	76,50

Groups Punta de Media Legua  &  Puerto Real

Average dissimilarity = 69,31

	Group Punta de Media Legua	Group Puerto Real	       	       	        	     

Species	                  Av.Abund	         Av.Abund	Av.Diss	Diss/SD	Contrib%	Cum.%

Pseudodiploria strigosa	                      1,63	             2,60	  14,88	   1,07	   21,46	21,46

Siderastrea siderea	                      1,59	             0,00	  13,05	   3,51	   18,83	40,30

Montastrea cavernosa	                      1,14	             0,00	   9,87	   1,35	   14,24	54,54

Porites astreoides	                      0,27	             1,10	   9,74	   1,43	   14,06	68,59

Diploria labyrinthiformis	                      0,83	             0,00	   5,26	   0,78	    7,59	76,18

Groups Petaquire  &  Puerto Real

Average dissimilarity = 52,58

	Group Petaquire	Group Puerto Real	       	       	        	     

Species	       Av.Abund	         Av.Abund	Av.Diss	Diss/SD	Contrib%	Cum.%

Porites astreoides	           0,00	             1,10	  11,38	   4,00	   21,65	21,65

Pseudodiploria strigosa	           2,75	             2,60	   9,32	   1,45	   17,72	39,37

Siderastrea siderea	           0,92	             0,00	   9,08	   4,01	   17,27	56,64

Other (Scleractinian massive)	           0,23	             0,35	   3,62	   0,91	    6,88	63,52

Siderastrea sp.	           0,37	             0,00	   3,42	   0,67	    6,51	70,03

Porites sp.	           0,00	             0,27	   3,13	   0,53	    5,95	75,99

Groups Playa Tiburon  &  Puerto Real

Average dissimilarity = 61,97

	Group Playa Tiburon	Group Puerto Real	       	       	        	     

Species	           Av.Abund	         Av.Abund	Av.Diss	Diss/SD	Contrib%	Cum.%

Siderastrea siderea	               1,64	             0,00	  12,83	   2,53	   20,70	20,70

Diploria labyrinthiformis	               1,38	             0,00	  10,55	   2,95	   17,02	37,71

Porites astreoides	               0,40	             1,10	   7,65	   2,79	   12,35	50,06

Pseudodiploria strigosa	               2,38	             2,60	   7,30	   1,43	   11,78	61,83

Montastrea cavernosa	               0,93	             0,00	   7,25	   1,33	   11,70	73,54

Meandrina meandrites	               0,75	             0,00	   5,38	   0,80	    8,69	82,22

Groups Punta Mono  &  Puerto Real

Average dissimilarity = 60,34

	Group Punta Mono	Group Puerto Real	       	       	        	     

Species	        Av.Abund	         Av.Abund	Av.Diss	Diss/SD	Contrib%	Cum.%

Siderastrea siderea	            1,34	             0,00	  10,81	   3,64	   17,91	17,91

Meandrina meandrites	            1,21	             0,00	   9,98	   1,64	   16,55	34,46

Pseudodiploria strigosa	            2,96	             2,60	   8,42	   1,55	   13,95	48,41

Diploria labyrinthiformis	            1,02	             0,00	   8,14	   3,33	   13,49	61,89

Porites astreoides	            0,17	             1,10	   7,83	   2,30	   12,97	74,86

Other (Scleractinian massive)	            0,17	             0,35	   2,88	   0,93	    4,78	79,64

Groups Charagato  &  Puerto Real

Average dissimilarity = 92,83

	Group Charagato	Group Puerto Real	       	       	        	     

Species	       Av.Abund	         Av.Abund	Av.Diss	Diss/SD	Contrib%	Cum.%

Pseudodiploria strigosa	           0,40	             2,60	  35,17	   1,79	   37,89	37,89

Colpophyllia natans	           1,86	             0,00	  22,65	   1,52	   24,40	62,29

Porites astreoides	           0,00	             1,10	  17,14	   2,77	   18,47	80,75

Groups Punta Conejo  &  Puerto Real

Average dissimilarity = 57,33

	Group Punta Conejo	Group Puerto Real	       	       	        	     

Species	          Av.Abund	         Av.Abund	Av.Diss	Diss/SD	Contrib%	Cum.%

Pseudodiploria strigosa	              1,99	             2,60	  25,02	   1,38	   43,65	43,65

Porites astreoides	              0,17	             1,10	  14,81	   1,98	   25,84	69,49

Other (Scleractinian massive)	              0,17	             0,35	   5,32	   0,90	    9,28	78,77

Groups La Muerta  &  Puerto Real

Average dissimilarity = 58,70

	Group La Muerta	Group Puerto Real	       	       	        	     

Species	       Av.Abund	         Av.Abund	Av.Diss	Diss/SD	Contrib%	Cum.%

Pseudodiploria strigosa	           1,99	             2,60	  21,69	   0,98	   36,95	36,95

Porites astreoides	           0,52	             1,10	  15,64	   2,24	   26,65	63,60

Colpophyllia natans	           0,62	             0,00	   6,56	   0,56	   11,17	74,77

Other (Scleractinian massive)	           0,00	             0,35	   5,11	   0,85	    8,71	83,48

Groups Cominoto  &  Puerto Real

Average dissimilarity = 32,37

	Group Cominoto	Group Puerto Real	       	       	        	     

Species	      Av.Abund	         Av.Abund	Av.Diss	Diss/SD	Contrib%	Cum.%

Pseudodiploria strigosa	          2,96	             2,60	  12,32	   1,36	   38,07	38,07

Porites astreoides	          0,58	             1,10	   9,88	   2,16	   30,53	68,60

Other (Scleractinian massive)	          0,00	             0,35	   4,45	   0,96	   13,75	82,35

Groups La Pecha  &  Puerto Real

Average dissimilarity = 93,15

	Group La Pecha	Group Puerto Real	       	       	        	     

Species	      Av.Abund	         Av.Abund	Av.Diss	Diss/SD	Contrib%	Cum.%

Pseudodiploria strigosa	          0,17	             2,60	  50,69	   3,32	   54,41	54,41

Porites astreoides	          0,00	             1,10	  24,37	   5,32	   26,16	80,58

Groups La Pared  &  Cayo Agua

Average dissimilarity = 96,83

	Group La Pared	Group Cayo Agua	       	       	        	     

Species	      Av.Abund	       Av.Abund	Av.Diss	Diss/SD	Contrib%	Cum.%

Madracis mirabilis	          0,00	           3,47	  13,74	   4,22	   14,19	14,19

Orbicella faveolata	          0,00	           3,24	  12,97	   6,03	   13,39	27,58

Orbicella annularis	          0,00	           3,05	  12,05	   3,49	   12,45	40,03

Pseudodiploria strigosa	          2,33	           0,00	   9,30	   3,00	    9,61	49,64

Meandrina meandrites	          1,94	           0,00	   7,46	   1,56	    7,71	57,35

Siderastrea siderea	          1,37	           0,00	   5,47	   5,69	    5,65	63,00

Montastrea cavernosa	          1,38	           0,17	   4,97	   1,67	    5,14	68,13

Colpophyllia natans	          0,00	           1,15	   4,52	   1,27	    4,66	72,80

Madracis decactis	          0,00	           1,10	   4,22	   0,96	    4,36	77,16

Groups Media Legua  &  Cayo Agua

Average dissimilarity = 95,28

	Group Media Legua	Group Cayo Agua	       	       	        	     

Species	         Av.Abund	       Av.Abund	Av.Diss	Diss/SD	Contrib%	Cum.%

Madracis mirabilis	             0,00	           3,47	  17,23	   4,40	   18,08	18,08

Orbicella faveolata	             0,17	           3,24	  15,44	   4,94	   16,20	34,29

Orbicella annularis	             0,00	           3,05	  15,10	   3,60	   15,85	50,14

Pseudodiploria strigosa	             2,20	           0,00	  10,95	   4,34	   11,49	61,63

Colpophyllia natans	             0,17	           1,15	   5,25	   1,26	    5,52	67,14

Madracis decactis	             0,00	           1,10	   5,25	   0,96	    5,51	72,65

Siderastrea siderea	             0,85	           0,00	   4,22	   1,44	    4,43	77,08

Groups Punta de Media Legua  &  Cayo Agua

Average dissimilarity = 96,94

	Group Punta de Media Legua	Group Cayo Agua	       	       	        	     

Species	                  Av.Abund	       Av.Abund	Av.Diss	Diss/SD	Contrib%	Cum.%

Madracis mirabilis	                      0,00	           3,47	  16,67	   3,36	   17,19	17,19

Orbicella faveolata	                      0,00	           3,24	  15,77	   3,90	   16,27	33,46

Orbicella annularis	                      0,00	           3,05	  14,61	   2,96	   15,07	48,53

Pseudodiploria strigosa	                      1,63	           0,00	   7,13	   1,63	    7,36	55,88

Siderastrea siderea	                      1,59	           0,00	   6,85	   2,21	    7,06	62,94

Colpophyllia natans	                      0,17	           1,15	   5,16	   1,16	    5,33	68,27

Madracis decactis	                      0,00	           1,10	   5,08	   0,94	    5,24	73,51

Montastrea cavernosa	                      1,14	           0,17	   4,76	   1,53	    4,91	78,41

Groups Petaquire  &  Cayo Agua

Average dissimilarity = 95,84

	Group Petaquire	Group Cayo Agua	       	       	        	     

Species	       Av.Abund	       Av.Abund	Av.Diss	Diss/SD	Contrib%	Cum.%

Madracis mirabilis	           0,00	           3,47	  17,06	   4,02	   17,80	17,80

Orbicella faveolata	           0,23	           3,24	  15,11	   3,82	   15,77	33,57

Orbicella annularis	           0,00	           3,05	  14,95	   3,37	   15,60	49,17

Pseudodiploria strigosa	           2,75	           0,00	  13,42	   6,49	   14,00	63,17

Madracis decactis	           0,00	           1,10	   5,20	   0,95	    5,42	68,59

Colpophyllia natans	           0,23	           1,15	   5,12	   1,20	    5,34	73,93

Siderastrea siderea	           0,92	           0,00	   4,46	   3,38	    4,66	78,59

Groups Playa Tiburon  &  Cayo Agua

Average dissimilarity = 96,58

	Group Playa Tiburon	Group Cayo Agua	       	       	        	     

Species	           Av.Abund	       Av.Abund	Av.Diss	Diss/SD	Contrib%	Cum.%

Madracis mirabilis	               0,00	           3,47	  14,94	   4,22	   15,47	15,47

Orbicella faveolata	               0,00	           3,24	  14,11	   5,84	   14,61	30,09

Orbicella annularis	               0,00	           3,05	  13,10	   3,50	   13,57	43,65

Pseudodiploria strigosa	               2,38	           0,00	  10,25	   4,29	   10,61	54,27

Siderastrea siderea	               1,64	           0,00	   7,08	   2,48	    7,33	61,60

Diploria labyrinthiformis	               1,38	           0,00	   5,89	   2,80	    6,09	67,69

Colpophyllia natans	               0,00	           1,15	   4,91	   1,27	    5,08	72,77

Madracis decactis	               0,00	           1,10	   4,58	   0,96	    4,74	77,51

Groups Punta Mono  &  Cayo Agua

Average dissimilarity = 98,89

	Group Punta Mono	Group Cayo Agua	       	       	        	     

Species	        Av.Abund	       Av.Abund	Av.Diss	Diss/SD	Contrib%	Cum.%

Madracis mirabilis	            0,00	           3,47	  15,25	   4,10	   15,42	15,42

Orbicella faveolata	            0,00	           3,24	  14,41	   5,48	   14,57	29,99

Orbicella annularis	            0,00	           3,05	  13,37	   3,43	   13,52	43,52

Pseudodiploria strigosa	            2,96	           0,00	  12,83	   6,36	   12,98	56,49

Siderastrea siderea	            1,34	           0,00	   5,87	   3,49	    5,93	62,43

Meandrina meandrites	            1,21	           0,00	   5,36	   1,60	    5,42	67,84

Colpophyllia natans	            0,00	           1,15	   5,01	   1,26	    5,06	72,91

Madracis decactis	            0,00	           1,10	   4,67	   0,96	    4,72	77,62

Groups Charagato  &  Cayo Agua

Average dissimilarity = 91,44

	Group Charagato	Group Cayo Agua	       	       	        	     

Species	       Av.Abund	       Av.Abund	Av.Diss	Diss/SD	Contrib%	Cum.%

Madracis mirabilis	           0,00	           3,47	  20,06	   3,91	   21,94	21,94

Orbicella faveolata	           0,00	           3,24	  19,03	   4,62	   20,81	42,75

Orbicella annularis	           0,00	           3,05	  17,57	   3,33	   19,22	61,97

Colpophyllia natans	           1,86	           1,15	   7,91	   1,39	    8,65	70,62

Madracis decactis	           0,00	           1,10	   6,07	   0,95	    6,64	77,26

Groups Punta Conejo  &  Cayo Agua

Average dissimilarity = 99,09

	Group Punta Conejo	Group Cayo Agua	       	       	        	     

Species	          Av.Abund	       Av.Abund	Av.Diss	Diss/SD	Contrib%	Cum.%

Madracis mirabilis	              0,00	           3,47	  19,99	   4,08	   20,17	20,17

Orbicella faveolata	              0,00	           3,24	  18,95	   4,92	   19,13	39,30

Orbicella annularis	              0,00	           3,05	  17,51	   3,43	   17,67	56,97

Pseudodiploria strigosa	              1,99	           0,00	  10,66	   1,35	   10,76	67,73

Colpophyllia natans	              0,00	           1,15	   6,54	   1,25	    6,60	74,33

Madracis decactis	              0,00	           1,10	   6,05	   0,96	    6,10	80,43

Groups La Muerta  &  Cayo Agua

Average dissimilarity = 92,23

	Group La Muerta	Group Cayo Agua	       	       	        	     

Species	       Av.Abund	       Av.Abund	Av.Diss	Diss/SD	Contrib%	Cum.%

Madracis mirabilis	           0,00	           3,47	  19,31	   3,72	   20,94	20,94

Orbicella annularis	           0,00	           3,05	  16,92	   3,20	   18,34	39,28

Orbicella faveolata	           0,35	           3,24	  16,70	   2,76	   18,10	57,39

Pseudodiploria strigosa	           1,99	           0,00	  10,20	   1,63	   11,06	68,45

Colpophyllia natans	           0,62	           1,15	   6,61	   1,27	    7,17	75,62

Groups Cominoto  &  Cayo Agua

Average dissimilarity = 97,38

	Group Cominoto	Group Cayo Agua	       	       	        	     

Species	      Av.Abund	       Av.Abund	Av.Diss	Diss/SD	Contrib%	Cum.%

Madracis mirabilis	          0,00	           3,47	  18,85	   4,51	   19,36	19,36

Orbicella faveolata	          0,00	           3,24	  17,86	   6,02	   18,34	37,70

Orbicella annularis	          0,00	           3,05	  16,52	   3,67	   16,96	54,67

Pseudodiploria strigosa	          2,96	           0,00	  16,24	   5,07	   16,67	71,34

Colpophyllia natans	          0,00	           1,15	   6,18	   1,27	    6,34	77,68

Groups La Pecha  &  Cayo Agua

Average dissimilarity = 100,00

	Group La Pecha	Group Cayo Agua	       	       	        	     

Species	      Av.Abund	       Av.Abund	Av.Diss	Diss/SD	Contrib%	Cum.%

Madracis mirabilis	          0,00	           3,47	  23,09	   4,57	   23,09	23,09

Orbicella faveolata	          0,00	           3,24	  21,95	   5,52	   21,95	45,04

Orbicella annularis	          0,00	           3,05	  20,22	   3,72	   20,22	65,26

Colpophyllia natans	          0,00	           1,15	   7,55	   1,26	    7,55	72,80

Madracis decactis	          0,00	           1,10	   6,94	   0,97	    6,94	79,74

Groups Puerto Real  &  Cayo Agua

Average dissimilarity = 95,11

	Group Puerto Real	Group Cayo Agua	       	       	        	     

Species	         Av.Abund	       Av.Abund	Av.Diss	Diss/SD	Contrib%	Cum.%

Madracis mirabilis	             0,00	           3,47	  17,95	   4,43	   18,87	18,87

Orbicella faveolata	             0,00	           3,24	  16,99	   5,94	   17,86	36,73

Orbicella annularis	             0,00	           3,05	  15,73	   3,62	   16,54	53,27

Pseudodiploria strigosa	             2,60	           0,00	  13,35	   2,88	   14,04	67,31

Colpophyllia natans	             0,00	           1,15	   5,88	   1,27	    6,19	73,49

Madracis decactis	             0,00	           1,10	   5,46	   0,97	    5,74	79,23

Groups La Pared  &  Boca de Cote

Average dissimilarity = 65,97

	Group La Pared	Group Boca de Cote	       	       	        	     

Species	      Av.Abund	          Av.Abund	Av.Diss	Diss/SD	Contrib%	Cum.%

Orbicella faveolata	          0,00	              3,10	  11,97	   3,94	   18,15	18,15

Pseudodiploria strigosa	          2,33	              0,62	   6,98	   1,73	   10,57	28,72

Meandrina meandrites	          1,94	              0,17	   6,97	   1,66	   10,56	39,28

Porites astreoides	          0,45	              1,92	   5,82	   2,18	    8,82	48,10

Orbicella annularis	          0,00	              1,35	   5,03	   1,52	    7,62	55,72

Siderastrea siderea	          1,37	              2,54	   4,48	   2,16	    6,79	62,51

Montastrea cavernosa	          1,38	              1,76	   3,79	   1,20	    5,74	68,25

Madracis mirabilis	          0,00	              1,01	   3,62	   0,73	    5,49	73,74

Diploria labyrinthiformis	          0,72	              1,37	   3,50	   1,47	    5,31	79,05

Groups Media Legua  &  Boca de Cote

Average dissimilarity = 76,44

	Group Media Legua	Group Boca de Cote	       	       	        	     

Species	         Av.Abund	          Av.Abund	Av.Diss	Diss/SD	Contrib%	Cum.%

Orbicella faveolata	             0,17	              3,10	  14,10	   3,46	   18,45	18,45

Porites astreoides	             0,17	              1,92	   8,58	   3,09	   11,22	29,67

Siderastrea siderea	             0,85	              2,54	   8,15	   2,11	   10,67	40,34

Pseudodiploria strigosa	             2,20	              0,62	   7,91	   1,73	   10,34	50,68

Montastrea cavernosa	             0,45	              1,76	   6,54	   1,64	    8,55	59,24

Orbicella annularis	             0,00	              1,35	   6,22	   1,53	    8,13	67,37

Diploria labyrinthiformis	             0,17	              1,37	   6,00	   2,01	    7,85	75,22

Groups Punta de Media Legua  &  Boca de Cote

Average dissimilarity = 68,59

	Group Punta de Media Legua	Group Boca de Cote	       	       	        	     

Species	                  Av.Abund	          Av.Abund	Av.Diss	Diss/SD	Contrib%	Cum.%

Orbicella faveolata	                      0,00	              3,10	  14,45	   3,16	   21,07	21,07

Porites astreoides	                      0,27	              1,92	   8,06	   2,08	   11,75	32,82

Siderastrea siderea	                      1,59	              2,54	   6,08	   1,28	    8,87	41,69

Orbicella annularis	                      0,00	              1,35	   6,02	   1,46	    8,77	50,46

Pseudodiploria strigosa	                      1,63	              0,62	   5,78	   1,44	    8,43	58,89

Diploria labyrinthiformis	                      0,83	              1,37	   5,73	   1,77	    8,36	67,24

Montastrea cavernosa	                      1,14	              1,76	   4,88	   1,20	    7,12	74,36

Madracis mirabilis	                      0,00	              1,01	   4,30	   0,73	    6,27	80,63

Groups Petaquire  &  Boca de Cote

Average dissimilarity = 78,75

	Group Petaquire	Group Boca de Cote	       	       	        	     

Species	       Av.Abund	          Av.Abund	Av.Diss	Diss/SD	Contrib%	Cum.%

Orbicella faveolata	           0,23	              3,10	  13,79	   2,97	   17,51	17,51

Pseudodiploria strigosa	           2,75	              0,62	  10,33	   2,24	   13,12	30,63

Porites astreoides	           0,00	              1,92	   9,27	   3,88	   11,77	42,40

Siderastrea siderea	           0,92	              2,54	   7,80	   2,35	    9,90	52,31

Montastrea cavernosa	           0,23	              1,76	   7,23	   1,77	    9,18	61,49

Orbicella annularis	           0,00	              1,35	   6,16	   1,50	    7,82	69,31

Diploria labyrinthiformis	           0,23	              1,37	   5,66	   1,82	    7,18	76,49

Groups Playa Tiburon  &  Boca de Cote

Average dissimilarity = 59,41

	Group Playa Tiburon	Group Boca de Cote	       	       	        	     

Species	           Av.Abund	          Av.Abund	Av.Diss	Diss/SD	Contrib%	Cum.%

Orbicella faveolata	               0,00	              3,10	  13,00	   3,90	   21,87	21,87

Pseudodiploria strigosa	               2,38	              0,62	   7,58	   1,86	   12,77	34,64

Porites astreoides	               0,40	              1,92	   6,64	   1,92	   11,18	45,82

Orbicella annularis	               0,00	              1,35	   5,44	   1,52	    9,16	54,98

Montastrea cavernosa	               0,93	              1,76	   4,46	   1,38	    7,51	62,49

Siderastrea siderea	               1,64	              2,54	   4,46	   1,68	    7,50	69,99

Madracis mirabilis	               0,00	              1,01	   3,91	   0,73	    6,58	76,57

Groups Punta Mono  &  Boca de Cote

Average dissimilarity = 71,04

	Group Punta Mono	Group Boca de Cote	       	       	        	     

Species	        Av.Abund	          Av.Abund	Av.Diss	Diss/SD	Contrib%	Cum.%

Orbicella faveolata	            0,00	              3,10	  13,26	   3,79	   18,66	18,66

Pseudodiploria strigosa	            2,96	              0,62	  10,05	   2,39	   14,15	32,81

Porites astreoides	            0,17	              1,92	   7,61	   2,99	   10,71	43,51

Montastrea cavernosa	            0,27	              1,76	   6,57	   1,84	    9,25	52,76

Orbicella annularis	            0,00	              1,35	   5,55	   1,52	    7,81	60,57

Siderastrea siderea	            1,34	              2,54	   5,31	   1,96	    7,48	68,05

Meandrina meandrites	            1,21	              0,17	   4,78	   1,63	    6,73	74,78

Madracis mirabilis	            0,00	              1,01	   3,98	   0,73	    5,60	80,38

Groups Charagato  &  Boca de Cote

Average dissimilarity = 94,98

	Group Charagato	Group Boca de Cote	       	       	        	     

Species	       Av.Abund	          Av.Abund	Av.Diss	Diss/SD	Contrib%	Cum.%

Orbicella faveolata	           0,00	              3,10	  17,33	   3,50	   18,24	18,24

Siderastrea siderea	           0,27	              2,54	  12,95	   2,62	   13,63	31,88

Porites astreoides	           0,00	              1,92	  10,88	   3,65	   11,46	43,33

Montastrea cavernosa	           0,00	              1,76	   9,71	   2,26	   10,22	53,56

Colpophyllia natans	           1,86	              0,17	   9,23	   1,43	    9,71	63,27

Diploria labyrinthiformis	           0,00	              1,37	   7,91	   2,45	    8,33	71,60

Orbicella annularis	           0,00	              1,35	   7,15	   1,51	    7,53	79,14

Groups Punta Conejo  &  Boca de Cote

Average dissimilarity = 91,90

	Group Punta Conejo	Group Boca de Cote	       	       	        	     

Species	          Av.Abund	          Av.Abund	Av.Diss	Diss/SD	Contrib%	Cum.%

Orbicella faveolata	              0,00	              3,10	  17,27	   3,62	   18,79	18,79

Siderastrea siderea	              0,17	              2,54	  13,31	   3,25	   14,48	33,27

Porites astreoides	              0,17	              1,92	   9,99	   2,80	   10,87	44,15

Montastrea cavernosa	              0,00	              1,76	   9,68	   2,29	   10,53	54,68

Pseudodiploria strigosa	              1,99	              0,62	   9,12	   1,31	    9,92	64,59

Diploria labyrinthiformis	              0,00	              1,37	   7,88	   2,50	    8,58	73,17

Orbicella annularis	              0,00	              1,35	   7,13	   1,52	    7,76	80,93

Groups La Muerta  &  Boca de Cote

Average dissimilarity = 88,12

	Group La Muerta	Group Boca de Cote	       	       	        	     

Species	       Av.Abund	          Av.Abund	Av.Diss	Diss/SD	Contrib%	Cum.%

Orbicella faveolata	           0,35	              3,10	  15,13	   2,35	   17,17	17,17

Siderastrea siderea	           0,00	              2,54	  13,69	   3,85	   15,53	32,70

Montastrea cavernosa	           0,00	              1,76	   9,36	   2,22	   10,62	43,32

Pseudodiploria strigosa	           1,99	              0,62	   8,66	   1,62	    9,83	53,15

Porites astreoides	           0,52	              1,92	   8,66	   1,71	    9,83	62,98

Diploria labyrinthiformis	           0,00	              1,37	   7,62	   2,41	    8,64	71,62

Orbicella annularis	           0,00	              1,35	   6,90	   1,49	    7,83	79,46

Groups Cominoto  &  Boca de Cote

Average dissimilarity = 88,24

	Group Cominoto	Group Boca de Cote	       	       	        	     

Species	      Av.Abund	          Av.Abund	Av.Diss	Diss/SD	Contrib%	Cum.%

Orbicella faveolata	          0,00	              3,10	  16,31	   3,97	   18,49	18,49

Siderastrea siderea	          0,00	              2,54	  13,37	   4,80	   15,16	33,64

Pseudodiploria strigosa	          2,96	              0,62	  12,78	   2,27	   14,48	48,13

Montastrea cavernosa	          0,00	              1,76	   9,15	   2,37	   10,37	58,49

Diploria labyrinthiformis	          0,00	              1,37	   7,43	   2,64	    8,42	66,91

Porites astreoides	          0,58	              1,92	   7,35	   1,81	    8,33	75,24

Groups La Pecha  &  Boca de Cote

Average dissimilarity = 99,05

	Group La Pecha	Group Boca de Cote	       	       	        	     

Species	      Av.Abund	          Av.Abund	Av.Diss	Diss/SD	Contrib%	Cum.%

Orbicella faveolata	          0,00	              3,10	  19,88	   3,84	   20,07	20,07

Siderastrea siderea	          0,00	              2,54	  16,31	   4,55	   16,46	36,54

Porites astreoides	          0,00	              1,92	  12,51	   4,02	   12,63	49,16

Montastrea cavernosa	          0,00	              1,76	  11,13	   2,37	   11,23	60,40

Diploria labyrinthiformis	          0,00	              1,37	   9,12	   2,54	    9,21	69,60

Orbicella annularis	          0,00	              1,35	   8,15	   1,54	    8,23	77,83

Groups Puerto Real  &  Boca de Cote

Average dissimilarity = 82,47

	Group Puerto Real	Group Boca de Cote	       	       	        	     

Species	         Av.Abund	          Av.Abund	Av.Diss	Diss/SD	Contrib%	Cum.%

Orbicella faveolata	             0,00	              3,10	  15,54	   3,95	   18,85	18,85

Siderastrea siderea	             0,00	              2,54	  12,74	   4,76	   15,45	34,30

Pseudodiploria strigosa	             2,60	              0,62	  10,13	   1,62	   12,28	46,59

Montastrea cavernosa	             0,00	              1,76	   8,72	   2,35	   10,57	57,16

Diploria labyrinthiformis	             0,00	              1,37	   7,07	   2,65	    8,58	65,74

Orbicella annularis	             0,00	              1,35	   6,46	   1,54	    7,83	73,57

Madracis mirabilis	             0,00	              1,01	   4,61	   0,74	    5,59	79,15

Groups Cayo Agua  &  Boca de Cote

Average dissimilarity = 58,79

	Group Cayo Agua	Group Boca de Cote	       	       	        	     

Species	       Av.Abund	          Av.Abund	Av.Diss	Diss/SD	Contrib%	Cum.%

Madracis mirabilis	           3,47	              1,01	   8,78	   1,87	   14,94	14,94

Siderastrea siderea	           0,00	              2,54	   8,38	   5,00	   14,26	29,20

Orbicella annularis	           3,05	              1,35	   6,05	   1,54	   10,29	39,49

Montastrea cavernosa	           0,17	              1,76	   5,15	   1,96	    8,76	48,25

Diploria labyrinthiformis	           0,00	              1,37	   4,61	   2,82	    7,84	56,09

Porites astreoides	           0,69	              1,92	   4,41	   1,78	    7,51	63,60

Madracis decactis	           1,10	              0,00	   3,53	   0,96	    6,01	69,61

Colpophyllia natans	           1,15	              0,17	   3,52	   1,24	    5,98	75,59

Groups La Pared  &  Dos Mosquises Sur

Average dissimilarity = 89,92

	Group La Pared	Group Dos Mosquises Sur	       	       	        	     

Species	      Av.Abund	               Av.Abund	Av.Diss	Diss/SD	Contrib%	Cum.%

Orbicella faveolata	          0,00	                   3,41	  14,44	   3,32	   16,06	16,06

Colpophyllia natans	          0,00	                   2,54	  10,75	   4,08	   11,95	28,01

Madracis mirabilis	          0,00	                   2,55	  10,15	   1,60	   11,28	39,29

Orbicella annularis	          0,00	                   2,43	   9,69	   1,47	   10,78	50,07

Pseudodiploria strigosa	          2,33	                   0,45	   8,30	   2,03	    9,23	59,30

Meandrina meandrites	          1,94	                   0,00	   7,83	   1,55	    8,71	68,01

Montastrea cavernosa	          1,38	                   0,00	   5,56	   1,55	    6,18	74,18

Siderastrea siderea	          1,37	                   0,52	   3,62	   1,51	    4,03	78,21

Groups Media Legua  &  Dos Mosquises Sur

Average dissimilarity = 87,34

	Group Media Legua	Group Dos Mosquises Sur	       	       	        	     

Species	         Av.Abund	               Av.Abund	Av.Diss	Diss/SD	Contrib%	Cum.%

Orbicella faveolata	             0,17	                   3,41	  17,55	   2,89	   20,10	20,10

Colpophyllia natans	             0,17	                   2,54	  12,82	   3,28	   14,68	34,78

Madracis mirabilis	             0,00	                   2,55	  12,74	   1,61	   14,59	49,37

Orbicella annularis	             0,00	                   2,43	  12,18	   1,48	   13,94	63,31

Pseudodiploria strigosa	             2,20	                   0,45	   9,62	   2,04	   11,02	74,32

Siderastrea siderea	             0,85	                   0,52	   3,80	   1,32	    4,35	78,67

Groups Punta de Media Legua  &  Dos Mosquises Sur

Average dissimilarity = 90,36

	Group Punta de Media Legua	Group Dos Mosquises Sur	       	       	        	     

Species	                  Av.Abund	               Av.Abund	Av.Diss	Diss/SD	Contrib%	Cum.%

Orbicella faveolata	                      0,00	                   3,41	  17,88	   2,56	   19,79	19,79

Colpophyllia natans	                      0,17	                   2,54	  12,62	   2,44	   13,97	33,75

Madracis mirabilis	                      0,00	                   2,55	  12,32	   1,52	   13,64	47,39

Orbicella annularis	                      0,00	                   2,43	  11,78	   1,40	   13,04	60,43

Pseudodiploria strigosa	                      1,63	                   0,45	   6,91	   1,50	    7,65	68,08

Siderastrea siderea	                      1,59	                   0,52	   5,67	   1,70	    6,27	74,35

Montastrea cavernosa	                      1,14	                   0,00	   5,41	   1,42	    5,99	80,34

Groups Petaquire  &  Dos Mosquises Sur

Average dissimilarity = 85,40

	Group Petaquire	Group Dos Mosquises Sur	       	       	        	     

Species	       Av.Abund	               Av.Abund	Av.Diss	Diss/SD	Contrib%	Cum.%

Orbicella faveolata	           0,23	                   3,41	  17,20	   2,54	   20,14	20,14

Madracis mirabilis	           0,00	                   2,55	  12,62	   1,57	   14,77	34,92

Colpophyllia natans	           0,23	                   2,54	  12,52	   2,74	   14,65	49,57

Orbicella annularis	           0,00	                   2,43	  12,06	   1,44	   14,12	63,69

Pseudodiploria strigosa	           2,75	                   0,45	  12,00	   2,33	   14,05	77,74

Groups Playa Tiburon  &  Dos Mosquises Sur

Average dissimilarity = 90,45

	Group Playa Tiburon	Group Dos Mosquises Sur	       	       	        	     

Species	           Av.Abund	               Av.Abund	Av.Diss	Diss/SD	Contrib%	Cum.%

Orbicella faveolata	               0,00	                   3,41	  15,81	   3,21	   17,48	17,48

Colpophyllia natans	               0,00	                   2,54	  11,76	   3,92	   13,01	30,49

Madracis mirabilis	               0,00	                   2,55	  11,04	   1,60	   12,21	42,70

Orbicella annularis	               0,00	                   2,43	  10,55	   1,47	   11,66	54,36

Pseudodiploria strigosa	               2,38	                   0,45	   8,82	   1,91	    9,75	64,11

Diploria labyrinthiformis	               1,38	                   0,00	   6,22	   2,73	    6,87	70,99

Siderastrea siderea	               1,64	                   0,52	   5,33	   1,53	    5,89	76,87

Groups Punta Mono  &  Dos Mosquises Sur

Average dissimilarity = 90,57

	Group Punta Mono	Group Dos Mosquises Sur	       	       	        	     

Species	        Av.Abund	               Av.Abund	Av.Diss	Diss/SD	Contrib%	Cum.%

Orbicella faveolata	            0,00	                   3,41	  16,17	   3,11	   17,86	17,86

Colpophyllia natans	            0,00	                   2,54	  12,03	   3,75	   13,28	31,14

Pseudodiploria strigosa	            2,96	                   0,45	  11,52	   2,50	   12,72	43,86

Madracis mirabilis	            0,00	                   2,55	  11,27	   1,59	   12,44	56,30

Orbicella annularis	            0,00	                   2,43	  10,77	   1,46	   11,89	68,19

Meandrina meandrites	            1,21	                   0,00	   5,67	   1,58	    6,26	74,45

Diploria labyrinthiformis	            1,02	                   0,00	   4,70	   2,93	    5,19	79,64

Groups Charagato  &  Dos Mosquises Sur

Average dissimilarity = 79,52

	Group Charagato	Group Dos Mosquises Sur	       	       	        	     

Species	       Av.Abund	               Av.Abund	Av.Diss	Diss/SD	Contrib%	Cum.%

Orbicella faveolata	           0,00	                   3,41	  21,94	   2,67	   27,60	27,60

Madracis mirabilis	           0,00	                   2,55	  14,86	   1,57	   18,68	46,28

Orbicella annularis	           0,00	                   2,43	  14,21	   1,44	   17,87	64,15

Colpophyllia natans	           1,86	                   2,54	   8,64	   1,13	   10,87	75,02

Groups Punta Conejo  &  Dos Mosquises Sur

Average dissimilarity = 94,70

	Group Punta Conejo	Group Dos Mosquises Sur	       	       	        	     

Species	          Av.Abund	               Av.Abund	Av.Diss	Diss/SD	Contrib%	Cum.%

Orbicella faveolata	              0,00	                   3,41	  21,83	   2,75	   23,06	23,06

Colpophyllia natans	              0,00	                   2,54	  16,23	   3,23	   17,14	40,19

Madracis mirabilis	              0,00	                   2,55	  14,80	   1,59	   15,63	55,82

Orbicella annularis	              0,00	                   2,43	  14,15	   1,45	   14,95	70,77

Pseudodiploria strigosa	              1,99	                   0,45	  10,83	   1,39	   11,44	82,21

Groups La Muerta  &  Dos Mosquises Sur

Average dissimilarity = 86,14

	Group La Muerta	Group Dos Mosquises Sur	       	       	        	     

Species	       Av.Abund	               Av.Abund	Av.Diss	Diss/SD	Contrib%	Cum.%

Orbicella faveolata	           0,35	                   3,41	  19,33	   2,06	   22,44	22,44

Madracis mirabilis	           0,00	                   2,55	  14,30	   1,56	   16,60	39,04

Orbicella annularis	           0,00	                   2,43	  13,67	   1,43	   15,87	54,91

Colpophyllia natans	           0,62	                   2,54	  12,58	   1,55	   14,61	69,52

Pseudodiploria strigosa	           1,99	                   0,45	   9,90	   1,49	   11,50	81,01

Groups Cominoto  &  Dos Mosquises Sur

Average dissimilarity = 94,01

	Group Cominoto	Group Dos Mosquises Sur	       	       	        	     

Species	      Av.Abund	               Av.Abund	Av.Diss	Diss/SD	Contrib%	Cum.%

Orbicella faveolata	          0,00	                   3,41	  20,41	   3,05	   21,71	21,71

Colpophyllia natans	          0,00	                   2,54	  15,18	   3,70	   16,14	37,86

Pseudodiploria strigosa	          2,96	                   0,45	  14,90	   2,37	   15,85	53,71

Madracis mirabilis	          0,00	                   2,55	  13,95	   1,62	   14,84	68,55

Orbicella annularis	          0,00	                   2,43	  13,34	   1,48	   14,19	82,73

Groups La Pecha  &  Dos Mosquises Sur

Average dissimilarity = 99,41

	Group La Pecha	Group Dos Mosquises Sur	       	       	        	     

Species	      Av.Abund	               Av.Abund	Av.Diss	Diss/SD	Contrib%	Cum.%

Orbicella faveolata	          0,00	                   3,41	  25,71	   2,76	   25,86	25,86

Colpophyllia natans	          0,00	                   2,54	  19,10	   3,27	   19,21	45,07

Madracis mirabilis	          0,00	                   2,55	  17,12	   1,62	   17,22	62,29

Orbicella annularis	          0,00	                   2,43	  16,38	   1,48	   16,48	78,78

Groups Puerto Real  &  Dos Mosquises Sur

Average dissimilarity = 92,00

	Group Puerto Real	Group Dos Mosquises Sur	       	       	        	     

Species	         Av.Abund	               Av.Abund	Av.Diss	Diss/SD	Contrib%	Cum.%

Orbicella faveolata	             0,00	                   3,41	  19,32	   3,08	   21,00	21,00

Colpophyllia natans	             0,00	                   2,54	  14,37	   3,74	   15,62	36,63

Madracis mirabilis	             0,00	                   2,55	  13,27	   1,61	   14,43	51,05

Orbicella annularis	             0,00	                   2,43	  12,69	   1,48	   13,79	64,85

Pseudodiploria strigosa	             2,60	                   0,45	  12,14	   1,90	   13,20	78,04

Groups Cayo Agua  &  Dos Mosquises Sur

Average dissimilarity = 40,51

	Group Cayo Agua	Group Dos Mosquises Sur	       	       	        	     

Species	       Av.Abund	               Av.Abund	Av.Diss	Diss/SD	Contrib%	Cum.%

Madracis mirabilis	           3,47	                   2,55	   5,52	   1,04	   13,63	13,63

Orbicella annularis	           3,05	                   2,43	   5,42	   1,12	   13,38	27,01

Colpophyllia natans	           1,15	                   2,54	   5,16	   1,47	   12,74	39,75

Madracis decactis	           1,10	                   0,17	   3,78	   1,09	    9,33	49,08

Porites astreoides	           0,69	                   0,17	   2,54	   0,83	    6,26	55,34

Orbicella sp.	           0,68	                   0,00	   2,42	   0,91	    5,97	61,31

Orbicella faveolata	           3,24	                   3,41	   2,36	   1,34	    5,82	67,13

Siderastrea siderea	           0,00	                   0,52	   1,92	   0,83	    4,73	71,86

Pseudodiploria strigosa	           0,00	                   0,45	   1,56	   0,56	    3,86	75,71

Groups Boca de Cote  &  Dos Mosquises Sur

Average dissimilarity = 62,16

	Group Boca de Cote	Group Dos Mosquises Sur	       	       	        	     

Species	          Av.Abund	               Av.Abund	Av.Diss	Diss/SD	Contrib%	Cum.%

Colpophyllia natans	              0,17	                   2,54	   8,30	   3,44	   13,35	13,35

Madracis mirabilis	              1,01	                   2,55	   7,25	   1,43	   11,67	25,02

Siderastrea siderea	              2,54	                   0,52	   6,86	   2,65	   11,04	36,06

Orbicella annularis	              1,35	                   2,43	   6,18	   1,53	    9,94	46,00

Porites astreoides	              1,92	                   0,17	   6,11	   3,06	    9,84	55,84

Montastrea cavernosa	              1,76	                   0,00	   5,99	   2,31	    9,64	65,48

Diploria labyrinthiformis	              1,37	                   0,00	   4,81	   2,75	    7,73	73,21

Orbicella faveolata	              3,10	                   3,41	   3,03	   1,22	    4,88	78,09

Groups La Pared  &  Madrisqui

Average dissimilarity = 89,95

	Group La Pared	Group Madrisqui	       	       	        	     

Species	      Av.Abund	       Av.Abund	Av.Diss	Diss/SD	Contrib%	Cum.%

Orbicella faveolata	          0,00	           2,67	  13,29	   4,68	   14,78	14,78

Pseudodiploria strigosa	          2,33	           0,00	  11,71	   2,87	   13,02	27,80

Colpophyllia natans	          0,00	           1,96	   9,47	   1,65	   10,53	38,33

Meandrina meandrites	          1,94	           0,00	   9,28	   1,55	   10,32	48,65

Siderastrea siderea	          1,37	           0,00	   6,89	   4,99	    7,66	56,31

Montastrea cavernosa	          1,38	           0,52	   5,92	   1,31	    6,59	62,89

Porites astreoides	          0,45	           1,41	   5,87	   1,55	    6,52	69,41

Agaricia sp.	          0,17	           0,93	   4,26	   0,80	    4,74	74,15

Diploria labyrinthiformis	          0,72	           0,00	   3,68	   0,94	    4,10	78,25

Groups Media Legua  &  Madrisqui

Average dissimilarity = 91,15

	Group Media Legua	Group Madrisqui	       	       	        	     

Species	         Av.Abund	       Av.Abund	Av.Diss	Diss/SD	Contrib%	Cum.%

Orbicella faveolata	             0,17	           2,67	  16,69	   3,90	   18,31	18,31

Pseudodiploria strigosa	             2,20	           0,00	  14,74	   4,04	   16,17	34,48

Colpophyllia natans	             0,17	           1,96	  12,07	   1,77	   13,24	47,73

Porites astreoides	             0,17	           1,41	   8,69	   1,59	    9,54	57,26

Siderastrea siderea	             0,85	           0,00	   5,67	   1,43	    6,22	63,49

Agaricia sp.	             0,17	           0,93	   5,57	   0,82	    6,11	69,60

Montastrea cavernosa	             0,45	           0,52	   5,21	   0,94	    5,72	75,32

Groups Punta de Media Legua  &  Madrisqui

Average dissimilarity = 92,47

	Group Punta de Media Legua	Group Madrisqui	       	       	        	     

Species	                  Av.Abund	       Av.Abund	Av.Diss	Diss/SD	Contrib%	Cum.%

Orbicella faveolata	                      0,00	           2,67	  17,33	   3,05	   18,74	18,74

Colpophyllia natans	                      0,17	           1,96	  11,83	   1,49	   12,79	31,53

Pseudodiploria strigosa	                      1,63	           0,00	   9,34	   1,63	   10,10	41,63

Siderastrea siderea	                      1,59	           0,00	   8,91	   2,48	    9,63	51,26

Porites astreoides	                      0,27	           1,41	   8,32	   1,32	    9,00	60,26

Montastrea cavernosa	                      1,14	           0,52	   6,81	   1,20	    7,36	67,62

Agaricia sp.	                      0,00	           0,93	   5,30	   0,72	    5,73	73,35

Eusmilia fastigata	                      0,00	           0,68	   4,10	   0,91	    4,43	77,79

Groups Petaquire  &  Madrisqui

Average dissimilarity = 92,54

	Group Petaquire	Group Madrisqui	       	       	        	     

Species	       Av.Abund	       Av.Abund	Av.Diss	Diss/SD	Contrib%	Cum.%

Pseudodiploria strigosa	           2,75	           0,00	  17,99	   5,92	   19,44	19,44

Orbicella faveolata	           0,23	           2,67	  16,35	   2,99	   17,67	37,10

Colpophyllia natans	           0,23	           1,96	  11,86	   1,65	   12,82	49,92

Porites astreoides	           0,00	           1,41	   9,12	   1,52	    9,85	59,77

Siderastrea siderea	           0,92	           0,00	   5,96	   3,49	    6,44	66,21

Agaricia sp.	           0,23	           0,93	   5,52	   0,80	    5,97	72,18

Montastrea cavernosa	           0,23	           0,52	   4,56	   0,72	    4,93	77,11

Groups Playa Tiburon  &  Madrisqui

Average dissimilarity = 91,90

	Group Playa Tiburon	Group Madrisqui	       	       	        	     

Species	           Av.Abund	       Av.Abund	Av.Diss	Diss/SD	Contrib%	Cum.%

Orbicella faveolata	               0,00	           2,67	  14,80	   4,59	   16,11	16,11

Pseudodiploria strigosa	               2,38	           0,00	  13,18	   4,08	   14,34	30,45

Colpophyllia natans	               0,00	           1,96	  10,51	   1,65	   11,43	41,88

Siderastrea siderea	               1,64	           0,00	   9,10	   2,47	    9,90	51,78

Diploria labyrinthiformis	               1,38	           0,00	   7,54	   2,79	    8,20	59,99

Porites astreoides	               0,40	           1,41	   6,83	   1,37	    7,43	67,41

Montastrea cavernosa	               0,93	           0,52	   5,51	   1,35	    6,00	73,41

Agaricia sp.	               0,35	           0,93	   4,77	   0,88	    5,19	78,60

Groups Punta Mono  &  Madrisqui

Average dissimilarity = 96,46

	Group Punta Mono	Group Madrisqui	       	       	        	     

Species	        Av.Abund	       Av.Abund	Av.Diss	Diss/SD	Contrib%	Cum.%

Pseudodiploria strigosa	            2,96	           0,00	  16,54	   6,51	   17,15	17,15

Orbicella faveolata	            0,00	           2,67	  15,22	   4,29	   15,78	32,93

Colpophyllia natans	            0,00	           1,96	  10,79	   1,63	   11,18	44,11

Siderastrea siderea	            1,34	           0,00	   7,58	   3,46	    7,86	51,97

Porites astreoides	            0,17	           1,41	   7,44	   1,55	    7,71	59,68

Meandrina meandrites	            1,21	           0,00	   6,95	   1,60	    7,21	66,89

Diploria labyrinthiformis	            1,02	           0,00	   5,73	   3,05	    5,94	72,84

Agaricia sp.	            0,00	           0,93	   4,75	   0,74	    4,93	77,76

Groups Charagato  &  Madrisqui

Average dissimilarity = 82,79

	Group Charagato	Group Madrisqui	       	       	        	     

Species	       Av.Abund	       Av.Abund	Av.Diss	Diss/SD	Contrib%	Cum.%

Orbicella faveolata	           0,00	           2,67	  22,29	   3,61	   26,92	26,92

Colpophyllia natans	           1,86	           1,96	  12,03	   1,22	   14,53	41,45

Porites astreoides	           0,00	           1,41	  11,43	   1,51	   13,80	55,25

Agaricia sp.	           0,00	           0,93	   6,59	   0,74	    7,96	63,21

Eusmilia fastigata	           0,00	           0,68	   5,19	   0,94	    6,27	69,47

Montastrea cavernosa	           0,00	           0,52	   5,12	   0,54	    6,19	75,66

Groups Punta Conejo  &  Madrisqui

Average dissimilarity = 97,52

	Group Punta Conejo	Group Madrisqui	       	       	        	     

Species	          Av.Abund	       Av.Abund	Av.Diss	Diss/SD	Contrib%	Cum.%

Orbicella faveolata	              0,00	           2,67	  22,10	   3,89	   22,66	22,66

Colpophyllia natans	              0,00	           1,96	  15,39	   1,61	   15,78	38,44

Pseudodiploria strigosa	              1,99	           0,00	  14,77	   1,36	   15,15	53,59

Porites astreoides	              0,17	           1,41	  10,77	   1,51	   11,04	64,64

Agaricia sp.	              0,00	           0,93	   6,55	   0,74	    6,72	71,36

Eusmilia fastigata	              0,00	           0,68	   5,15	   0,95	    5,28	76,64

Groups La Muerta  &  Madrisqui

Average dissimilarity = 85,76

	Group La Muerta	Group Madrisqui	       	       	        	     

Species	       Av.Abund	       Av.Abund	Av.Diss	Diss/SD	Contrib%	Cum.%

Orbicella faveolata	           0,35	           2,67	  19,06	   2,16	   22,22	22,22

Pseudodiploria strigosa	           1,99	           0,00	  13,93	   1,62	   16,24	38,47

Colpophyllia natans	           0,62	           1,96	  13,00	   1,24	   15,15	53,62

Porites astreoides	           0,52	           1,41	  10,03	   1,29	   11,69	65,32

Agaricia sp.	           0,00	           0,93	   6,30	   0,73	    7,35	72,66

Eusmilia fastigata	           0,00	           0,68	   4,94	   0,93	    5,77	78,43

Groups Cominoto  &  Madrisqui

Average dissimilarity = 93,88

	Group Cominoto	Group Madrisqui	       	       	        	     

Species	      Av.Abund	       Av.Abund	Av.Diss	Diss/SD	Contrib%	Cum.%

Pseudodiploria strigosa	          2,96	           0,00	  22,63	   4,47	   24,11	24,11

Orbicella faveolata	          0,00	           2,67	  20,19	   4,99	   21,51	45,62

Colpophyllia natans	          0,00	           1,96	  14,15	   1,67	   15,07	60,68

Porites astreoides	          0,58	           1,41	   8,58	   1,48	    9,13	69,82

Agaricia sp.	          0,00	           0,93	   6,09	   0,75	    6,49	76,31

Groups La Pecha  &  Madrisqui

Average dissimilarity = 100,00

	Group La Pecha	Group Madrisqui	       	       	        	     

Species	      Av.Abund	       Av.Abund	Av.Diss	Diss/SD	Contrib%	Cum.%

Orbicella faveolata	          0,00	           2,67	  27,25	   4,69	   27,25	27,25

Colpophyllia natans	          0,00	           1,96	  18,75	   1,66	   18,75	46,00

Porites astreoides	          0,00	           1,41	  13,91	   1,59	   13,91	59,91

Agaricia sp.	          0,00	           0,93	   7,81	   0,76	    7,81	67,72

Montastrea cavernosa	          0,00	           0,52	   6,45	   0,56	    6,45	74,17

Eusmilia fastigata	          0,00	           0,68	   6,25	   0,97	    6,25	80,42

Groups Puerto Real  &  Madrisqui

Average dissimilarity = 86,00

	Group Puerto Real	Group Madrisqui	       	       	        	     

Species	         Av.Abund	       Av.Abund	Av.Diss	Diss/SD	Contrib%	Cum.%

Orbicella faveolata	             0,00	           2,67	  18,87	   4,85	   21,94	21,94

Pseudodiploria strigosa	             2,60	           0,00	  18,15	   2,91	   21,11	43,05

Colpophyllia natans	             0,00	           1,96	  13,26	   1,67	   15,42	58,47

Porites astreoides	             1,10	           1,41	   6,00	   2,30	    6,97	65,44

Agaricia sp.	             0,17	           0,93	   5,84	   0,81	    6,79	72,23

Eusmilia fastigata	             0,00	           0,68	   4,45	   0,96	    5,18	77,41

Groups Cayo Agua  &  Madrisqui

Average dissimilarity = 66,41

	Group Cayo Agua	Group Madrisqui	       	       	        	     

Species	       Av.Abund	       Av.Abund	Av.Diss	Diss/SD	Contrib%	Cum.%

Madracis mirabilis	           3,47	           0,00	  14,14	   4,26	   21,29	21,29

Orbicella annularis	           3,05	           0,00	  12,40	   3,52	   18,67	39,96

Colpophyllia natans	           1,15	           1,96	   5,66	   1,48	    8,52	48,49

Porites astreoides	           0,69	           1,41	   4,70	   1,43	    7,08	55,56

Madracis decactis	           1,10	           0,00	   4,34	   0,96	    6,53	62,09

Agaricia sp.	           0,00	           0,93	   3,51	   0,74	    5,29	67,38

Orbicella faveolata	           3,24	           2,67	   2,86	   1,25	    4,31	71,69

Orbicella sp.	           0,68	           0,00	   2,81	   0,91	    4,23	75,92

Groups Boca de Cote  &  Madrisqui

Average dissimilarity = 65,70

	Group Boca de Cote	Group Madrisqui	       	       	        	     

Species	          Av.Abund	       Av.Abund	Av.Diss	Diss/SD	Contrib%	Cum.%

Siderastrea siderea	              2,54	           0,00	  10,09	   4,77	   15,36	15,36

Colpophyllia natans	              0,17	           1,96	   7,32	   1,70	   11,14	26,50

Montastrea cavernosa	              1,76	           0,52	   5,78	   1,79	    8,80	35,30

Diploria labyrinthiformis	              1,37	           0,00	   5,57	   2,72	    8,48	43,78

Orbicella annularis	              1,35	           0,00	   5,16	   1,53	    7,86	51,64

Madracis mirabilis	              1,01	           0,00	   3,72	   0,73	    5,66	57,29

Agaricia sp.	              0,00	           0,93	   3,43	   0,73	    5,22	62,51

Orbicella faveolata	              3,10	           2,67	   3,24	   1,29	    4,92	67,44

Porites astreoides	              1,92	           1,41	   3,23	   1,02	    4,91	72,35

Eusmilia fastigata	              0,00	           0,68	   2,58	   0,95	    3,93	76,28

Groups Dos Mosquises Sur  &  Madrisqui

Average dissimilarity = 60,86

	Group Dos Mosquises Sur	Group Madrisqui	       	       	        	     

Species	               Av.Abund	       Av.Abund	Av.Diss	Diss/SD	Contrib%	Cum.%

Madracis mirabilis	                   2,55	           0,00	  10,44	   1,60	   17,15	17,15

Orbicella annularis	                   2,43	           0,00	   9,97	   1,48	   16,39	33,54

Porites astreoides	                   0,17	           1,41	   5,64	   1,56	    9,26	42,81

Orbicella faveolata	                   3,41	           2,67	   4,14	   1,16	    6,80	49,60

Colpophyllia natans	                   2,54	           1,96	   4,09	   0,82	    6,72	56,32

Agaricia sp.	                   0,00	           0,93	   3,68	   0,74	    6,05	62,38

Eusmilia fastigata	                   0,00	           0,68	   2,78	   0,95	    4,57	66,95

Montastrea cavernosa	                   0,00	           0,52	   2,43	   0,56	    3,99	70,94

Siderastrea siderea	                   0,52	           0,00	   2,37	   0,82	    3,89	74,83

Pseudodiploria strigosa	                   0,45	           0,00	   1,90	   0,56	    3,12	77,95

Groups La Pared  &  Pelona de Rabusqui

Average dissimilarity = 93,98

	Group La Pared	Group Pelona de Rabusqui	       	       	        	     

Species	      Av.Abund	                Av.Abund	Av.Diss	Diss/SD	Contrib%	Cum.%

Orbicella faveolata	          0,00	                    4,91	  22,59	   6,22	   24,03	24,03

Pseudodiploria strigosa	          2,33	                    0,17	  10,01	   2,38	   10,65	34,69

Orbicella annularis	          0,00	                    2,00	   9,00	   1,51	    9,58	44,27

Meandrina meandrites	          1,94	                    0,00	   8,53	   1,54	    9,08	53,34

Colpophyllia natans	          0,00	                    1,87	   8,40	   2,25	    8,94	62,28

Montastrea cavernosa	          1,38	                    0,00	   6,05	   1,54	    6,44	68,72

Siderastrea siderea	          1,37	                    0,27	   5,01	   1,97	    5,33	74,05

Agaricia sp.	          0,17	                    1,10	   4,32	   1,14	    4,59	78,65

Groups Media Legua  &  Pelona de Rabusqui

Average dissimilarity = 89,83

	Group Media Legua	Group Pelona de Rabusqui	       	       	        	     

Species	         Av.Abund	                Av.Abund	Av.Diss	Diss/SD	Contrib%	Cum.%

Orbicella faveolata	             0,17	                    4,91	  28,57	   5,17	   31,81	31,81

Pseudodiploria strigosa	             2,20	                    0,17	  12,25	   2,88	   13,64	45,44

Orbicella annularis	             0,00	                    2,00	  11,72	   1,52	   13,05	58,49

Colpophyllia natans	             0,17	                    1,87	   9,88	   1,90	   11,00	69,49

Agaricia sp.	             0,17	                    1,10	   5,61	   1,14	    6,24	75,73

Groups Punta de Media Legua  &  Pelona de Rabusqui

Average dissimilarity = 94,29

	Group Punta de Media Legua	Group Pelona de Rabusqui	       	       	        	     

Species	                  Av.Abund	                Av.Abund	Av.Diss	Diss/SD	Contrib%	Cum.%

Orbicella faveolata	                      0,00	                    4,91	  28,69	   3,36	   30,43	30,43

Orbicella annularis	                      0,00	                    2,00	  11,36	   1,41	   12,05	42,48

Colpophyllia natans	                      0,17	                    1,87	   9,83	   1,69	   10,42	52,90

Pseudodiploria strigosa	                      1,63	                    0,17	   8,04	   1,62	    8,52	61,43

Siderastrea siderea	                      1,59	                    0,27	   7,14	   2,02	    7,57	69,00

Agaricia sp.	                      0,00	                    1,10	   6,43	   1,35	    6,82	75,82

Groups Petaquire  &  Pelona de Rabusqui

Average dissimilarity = 88,55

	Group Petaquire	Group Pelona de Rabusqui	       	       	        	     

Species	       Av.Abund	                Av.Abund	Av.Diss	Diss/SD	Contrib%	Cum.%

Orbicella faveolata	           0,23	                    4,91	  28,11	   3,89	   31,75	31,75

Pseudodiploria strigosa	           2,75	                    0,17	  15,18	   3,70	   17,14	48,89

Orbicella annularis	           0,00	                    2,00	  11,61	   1,48	   13,11	62,00

Colpophyllia natans	           0,23	                    1,87	   9,60	   1,71	   10,84	72,84

Agaricia sp.	           0,23	                    1,10	   5,37	   1,04	    6,06	78,90

Groups Playa Tiburon  &  Pelona de Rabusqui

Average dissimilarity = 91,22

	Group Playa Tiburon	Group Pelona de Rabusqui	       	       	        	     

Species	           Av.Abund	                Av.Abund	Av.Diss	Diss/SD	Contrib%	Cum.%

Orbicella faveolata	               0,00	                    4,91	  24,94	   5,77	   27,34	27,34

Pseudodiploria strigosa	               2,38	                    0,17	  11,19	   3,02	   12,27	39,61

Orbicella annularis	               0,00	                    2,00	   9,92	   1,51	   10,87	50,49

Colpophyllia natans	               0,00	                    1,87	   9,25	   2,25	   10,14	60,62

Diploria labyrinthiformis	               1,38	                    0,00	   6,85	   2,71	    7,51	68,13

Siderastrea siderea	               1,64	                    0,27	   6,83	   1,63	    7,49	75,62

Groups Punta Mono  &  Pelona de Rabusqui

Average dissimilarity = 95,52

	Group Punta Mono	Group Pelona de Rabusqui	       	       	        	     

Species	        Av.Abund	                Av.Abund	Av.Diss	Diss/SD	Contrib%	Cum.%

Orbicella faveolata	            0,00	                    4,91	  25,58	   5,22	   26,77	26,77

Pseudodiploria strigosa	            2,96	                    0,17	  14,21	   4,01	   14,88	41,65

Orbicella annularis	            0,00	                    2,00	  10,16	   1,50	   10,64	52,29

Colpophyllia natans	            0,00	                    1,87	   9,47	   2,22	    9,92	62,21

Meandrina meandrites	            1,21	                    0,00	   6,28	   1,58	    6,58	68,78

Agaricia sp.	            0,00	                    1,10	   5,73	   1,44	    6,00	74,79

Siderastrea siderea	            1,34	                    0,27	   5,41	   1,65	    5,66	80,45

Groups Charagato  &  Pelona de Rabusqui

Average dissimilarity = 83,12

	Group Charagato	Group Pelona de Rabusqui	       	       	        	     

Species	       Av.Abund	                Av.Abund	Av.Diss	Diss/SD	Contrib%	Cum.%

Orbicella faveolata	           0,00	                    4,91	  36,06	   3,75	   43,38	43,38

Orbicella annularis	           0,00	                    2,00	  14,19	   1,47	   17,07	60,45

Colpophyllia natans	           1,86	                    1,87	   9,57	   1,36	   11,51	71,97

Agaricia sp.	           0,00	                    1,10	   8,08	   1,40	    9,72	81,69

Groups Punta Conejo  &  Pelona de Rabusqui

Average dissimilarity = 97,98

	Group Punta Conejo	Group Pelona de Rabusqui	       	       	        	     

Species	          Av.Abund	                Av.Abund	Av.Diss	Diss/SD	Contrib%	Cum.%

Orbicella faveolata	              0,00	                    4,91	  35,82	   4,05	   36,55	36,55

Orbicella annularis	              0,00	                    2,00	  14,10	   1,49	   14,39	50,95

Colpophyllia natans	              0,00	                    1,87	  13,07	   2,21	   13,34	64,29

Pseudodiploria strigosa	              1,99	                    0,17	  12,56	   1,33	   12,82	77,11

Groups La Muerta  &  Pelona de Rabusqui

Average dissimilarity = 89,47

	Group La Muerta	Group Pelona de Rabusqui	       	       	        	     

Species	       Av.Abund	                Av.Abund	Av.Diss	Diss/SD	Contrib%	Cum.%

Orbicella faveolata	           0,35	                    4,91	  32,51	   2,79	   36,33	36,33

Orbicella annularis	           0,00	                    2,00	  13,56	   1,45	   15,16	51,49

Pseudodiploria strigosa	           1,99	                    0,17	  11,95	   1,63	   13,36	64,85

Colpophyllia natans	           0,62	                    1,87	  11,00	   1,56	   12,30	77,14

Groups Cominoto  &  Pelona de Rabusqui

Average dissimilarity = 98,07

	Group Cominoto	Group Pelona de Rabusqui	       	       	        	     

Species	      Av.Abund	                Av.Abund	Av.Diss	Diss/SD	Contrib%	Cum.%

Orbicella faveolata	          0,00	                    4,91	  33,10	   5,40	   33,75	33,75

Pseudodiploria strigosa	          2,96	                    0,17	  18,92	   3,28	   19,30	53,05

Orbicella annularis	          0,00	                    2,00	  13,07	   1,53	   13,33	66,38

Colpophyllia natans	          0,00	                    1,87	  12,14	   2,31	   12,38	78,76

Groups La Pecha  &  Pelona de Rabusqui

Average dissimilarity = 99,43

	Group La Pecha	Group Pelona de Rabusqui	       	       	        	     

Species	      Av.Abund	                Av.Abund	Av.Diss	Diss/SD	Contrib%	Cum.%

Orbicella faveolata	          0,00	                    4,91	  43,18	   4,27	   43,43	43,43

Orbicella annularis	          0,00	                    2,00	  16,91	   1,53	   17,00	60,43

Colpophyllia natans	          0,00	                    1,87	  15,60	   2,33	   15,69	76,12

Groups Puerto Real  &  Pelona de Rabusqui

Average dissimilarity = 96,16

	Group Puerto Real	Group Pelona de Rabusqui	       	       	        	     

Species	         Av.Abund	                Av.Abund	Av.Diss	Diss/SD	Contrib%	Cum.%

Orbicella faveolata	             0,00	                    4,91	  31,13	   5,46	   32,38	32,38

Pseudodiploria strigosa	             2,60	                    0,17	  15,19	   2,34	   15,80	48,18

Orbicella annularis	             0,00	                    2,00	  12,31	   1,53	   12,80	60,98

Colpophyllia natans	             0,00	                    1,87	  11,45	   2,29	   11,91	72,89

Porites astreoides	             1,10	                    0,00	   6,93	   6,41	    7,21	80,10

Groups Cayo Agua  &  Pelona de Rabusqui

Average dissimilarity = 53,27

	Group Cayo Agua	Group Pelona de Rabusqui	       	       	        	     

Species	       Av.Abund	                Av.Abund	Av.Diss	Diss/SD	Contrib%	Cum.%

Madracis mirabilis	           3,47	                    0,00	  13,14	   4,14	   24,67	24,67

Orbicella faveolata	           3,24	                    4,91	   6,36	   4,30	   11,94	36,61

Orbicella annularis	           3,05	                    2,00	   5,76	   1,27	   10,81	47,41

Colpophyllia natans	           1,15	                    1,87	   4,37	   1,38	    8,20	55,61

Agaricia sp.	           0,00	                    1,10	   4,20	   1,48	    7,89	63,50

Madracis decactis	           1,10	                    0,00	   4,04	   0,96	    7,58	71,08

Orbicella sp.	           0,68	                    0,74	   3,96	   1,09	    7,43	78,51

Groups Boca de Cote  &  Pelona de Rabusqui

Average dissimilarity = 64,75

	Group Boca de Cote	Group Pelona de Rabusqui	       	       	        	     

Species	          Av.Abund	                Av.Abund	Av.Diss	Diss/SD	Contrib%	Cum.%

Siderastrea siderea	              2,54	                    0,27	   8,35	   3,12	   12,90	12,90

Porites astreoides	              1,92	                    0,00	   7,16	   4,21	   11,06	23,96

Orbicella faveolata	              3,10	                    4,91	   6,84	   2,09	   10,56	34,52

Montastrea cavernosa	              1,76	                    0,00	   6,44	   2,31	    9,95	44,47

Colpophyllia natans	              0,17	                    1,87	   6,26	   1,87	    9,67	54,14

Diploria labyrinthiformis	              1,37	                    0,00	   5,18	   2,71	    7,99	62,13

Orbicella annularis	              1,35	                    2,00	   5,15	   1,32	    7,96	70,09

Agaricia sp.	              0,00	                    1,10	   4,10	   1,47	    6,33	76,42

Groups Dos Mosquises Sur  &  Pelona de Rabusqui

Average dissimilarity = 46,79

	Group Dos Mosquises Sur	Group Pelona de Rabusqui	       	       	        	     

Species	               Av.Abund	                Av.Abund	Av.Diss	Diss/SD	Contrib%	Cum.%

Madracis mirabilis	                   2,55	                    0,00	   9,70	   1,59	   20,73	20,73

Orbicella annularis	                   2,43	                    2,00	   6,67	   1,22	   14,25	34,98

Orbicella faveolata	                   3,41	                    4,91	   5,92	   2,02	   12,65	47,64

Agaricia sp.	                   0,00	                    1,10	   4,41	   1,46	    9,43	57,07

Colpophyllia natans	                   2,54	                    1,87	   3,63	   1,06	    7,75	64,81

Orbicella sp.	                   0,00	                    0,74	   2,63	   0,56	    5,63	70,45

Siderastrea siderea	                   0,52	                    0,27	   2,35	   0,96	    5,03	75,48

Groups Madrisqui  &  Pelona de Rabusqui

Average dissimilarity = 58,61

	Group Madrisqui	Group Pelona de Rabusqui	       	       	        	     

Species	       Av.Abund	                Av.Abund	Av.Diss	Diss/SD	Contrib%	Cum.%

Orbicella faveolata	           2,67	                    4,91	  10,77	   3,02	   18,38	18,38

Orbicella annularis	           0,00	                    2,00	   9,30	   1,51	   15,87	34,25

Porites astreoides	           1,41	                    0,00	   6,53	   1,55	   11,13	45,38

Colpophyllia natans	           1,96	                    1,87	   5,35	   1,15	    9,13	54,51

Agaricia sp.	           0,93	                    1,10	   5,30	   1,26	    9,05	63,56

Orbicella sp.	           0,00	                    0,74	   3,06	   0,56	    5,22	68,78

Eusmilia fastigata	           0,68	                    0,00	   3,04	   0,95	    5,18	73,96

Montastrea cavernosa	           0,52	                    0,00	   2,69	   0,55	    4,58	78,55

Groups La Pared  &  Salinas

Average dissimilarity = 59,92

	Group La Pared	Group Salinas	       	       	        	     

Species	      Av.Abund	     Av.Abund	Av.Diss	Diss/SD	Contrib%	Cum.%

Orbicella faveolata	          0,00	         2,24	  10,60	   2,08	   17,70	17,70

Meandrina meandrites	          1,94	         0,00	   8,73	   1,53	   14,57	32,26

Pseudodiploria strigosa	          2,33	         1,32	   6,21	   1,29	   10,36	42,62

Diploria labyrinthiformis	          0,72	         1,40	   4,93	   1,32	    8,23	50,85

Montastrea cavernosa	          1,38	         1,66	   4,82	   1,05	    8,04	58,89

Siderastrea siderea	          1,37	         2,20	   4,44	   1,40	    7,41	66,30

Porites astreoides	          0,45	         0,68	   3,08	   1,18	    5,15	71,44

Other (Scleractinian massive)	          0,62	         0,17	   3,04	   0,98	    5,08	76,52

Groups Media Legua  &  Salinas

Average dissimilarity = 66,30

	Group Media Legua	Group Salinas	       	       	        	     

Species	         Av.Abund	     Av.Abund	Av.Diss	Diss/SD	Contrib%	Cum.%

Orbicella faveolata	             0,17	         2,24	  13,00	   1,77	   19,61	19,61

Siderastrea siderea	             0,85	         2,20	   8,69	   1,48	   13,10	32,71

Montastrea cavernosa	             0,45	         1,66	   8,39	   1,86	   12,66	45,37

Diploria labyrinthiformis	             0,17	         1,40	   7,77	   1,54	   11,72	57,09

Pseudodiploria strigosa	             2,20	         1,32	   6,87	   1,07	   10,37	67,46

Porites astreoides	             0,17	         0,68	   4,05	   1,08	    6,10	73,56

Meandrina meandrites	             0,60	         0,00	   3,74	   0,55	    5,64	79,21

Groups Punta de Media Legua  &  Salinas

Average dissimilarity = 60,24

	Group Punta de Media Legua	Group Salinas	       	       	        	     

Species	                  Av.Abund	     Av.Abund	Av.Diss	Diss/SD	Contrib%	Cum.%

Orbicella faveolata	                      0,00	         2,24	  13,65	   1,71	   22,67	22,67

Diploria labyrinthiformis	                      0,83	         1,40	   7,51	   1,33	   12,46	35,13

Siderastrea siderea	                      1,59	         2,20	   7,33	   1,21	   12,18	47,31

Montastrea cavernosa	                      1,14	         1,66	   6,73	   1,22	   11,17	58,48

Pseudodiploria strigosa	                      1,63	         1,32	   6,52	   1,21	   10,82	69,30

Porites astreoides	                      0,27	         0,68	   3,93	   0,95	    6,53	75,83

Groups Petaquire  &  Salinas

Average dissimilarity = 66,08

	Group Petaquire	Group Salinas	       	       	        	     

Species	       Av.Abund	     Av.Abund	Av.Diss	Diss/SD	Contrib%	Cum.%

Orbicella faveolata	           0,23	         2,24	  12,70	   1,60	   19,21	19,21

Pseudodiploria strigosa	           2,75	         1,32	   9,08	   1,21	   13,74	32,95

Montastrea cavernosa	           0,23	         1,66	   8,82	   1,73	   13,34	46,29

Siderastrea siderea	           0,92	         2,20	   8,19	   1,48	   12,39	58,68

Diploria labyrinthiformis	           0,23	         1,40	   7,55	   1,52	   11,43	70,11

Porites astreoides	           0,00	         0,68	   4,01	   0,94	    6,07	76,18

Groups Playa Tiburon  &  Salinas

Average dissimilarity = 53,29

	Group Playa Tiburon	Group Salinas	       	       	        	     

Species	           Av.Abund	     Av.Abund	Av.Diss	Diss/SD	Contrib%	Cum.%

Orbicella faveolata	               0,00	         2,24	  11,76	   2,02	   22,06	22,06

Pseudodiploria strigosa	               2,38	         1,32	   6,40	   1,15	   12,01	34,07

Montastrea cavernosa	               0,93	         1,66	   6,05	   1,60	   11,35	45,42

Siderastrea siderea	               1,64	         2,20	   4,88	   1,30	    9,16	54,58

Diploria labyrinthiformis	               1,38	         1,40	   4,39	   1,22	    8,23	62,82

Porites astreoides	               0,40	         0,68	   3,76	   1,04	    7,06	69,87

Meandrina meandrites	               0,75	         0,00	   3,67	   0,78	    6,89	76,76

Groups Punta Mono  &  Salinas

Average dissimilarity = 63,09

	Group Punta Mono	Group Salinas	       	       	        	     

Species	        Av.Abund	     Av.Abund	Av.Diss	Diss/SD	Contrib%	Cum.%

Orbicella faveolata	            0,00	         2,24	  12,07	   1,98	   19,13	19,13

Pseudodiploria strigosa	            2,96	         1,32	   9,08	   1,48	   14,39	33,52

Montastrea cavernosa	            0,27	         1,66	   7,67	   1,63	   12,15	45,67

Meandrina meandrites	            1,21	         0,00	   6,47	   1,57	   10,25	55,92

Siderastrea siderea	            1,34	         2,20	   5,39	   1,29	    8,54	64,47

Diploria labyrinthiformis	            1,02	         1,40	   4,89	   1,76	    7,76	72,22

Porites astreoides	            0,17	         0,68	   3,49	   1,07	    5,53	77,76

Groups Charagato  &  Salinas

Average dissimilarity = 90,10

	Group Charagato	Group Salinas	       	       	        	     

Species	       Av.Abund	     Av.Abund	Av.Diss	Diss/SD	Contrib%	Cum.%

Orbicella faveolata	           0,00	         2,24	  17,39	   1,72	   19,30	19,30

Siderastrea siderea	           0,27	         2,20	  15,09	   1,72	   16,75	36,05

Colpophyllia natans	           1,86	         0,27	  11,61	   1,54	   12,88	48,93

Montastrea cavernosa	           0,00	         1,66	  11,35	   1,60	   12,60	61,53

Diploria labyrinthiformis	           0,00	         1,40	   9,87	   1,42	   10,96	72,49

Pseudodiploria strigosa	           0,40	         1,32	   8,36	   1,27	    9,28	81,77

Groups Punta Conejo  &  Salinas

Average dissimilarity = 85,15

	Group Punta Conejo	Group Salinas	       	       	        	     

Species	          Av.Abund	     Av.Abund	Av.Diss	Diss/SD	Contrib%	Cum.%

Orbicella faveolata	              0,00	         2,24	  17,25	   1,76	   20,26	20,26

Siderastrea siderea	              0,17	         2,20	  15,47	   1,98	   18,17	38,42

Pseudodiploria strigosa	              1,99	         1,32	  11,48	   1,47	   13,48	51,90

Montastrea cavernosa	              0,00	         1,66	  11,29	   1,62	   13,26	65,16

Diploria labyrinthiformis	              0,00	         1,40	   9,81	   1,43	   11,52	76,68

Groups La Muerta  &  Salinas

Average dissimilarity = 81,62

	Group La Muerta	Group Salinas	       	       	        	     

Species	       Av.Abund	     Av.Abund	Av.Diss	Diss/SD	Contrib%	Cum.%

Siderastrea siderea	           0,00	         2,20	  15,90	   2,17	   19,48	19,48

Orbicella faveolata	           0,35	         2,24	  14,81	   1,38	   18,14	37,62

Montastrea cavernosa	           0,00	         1,66	  10,86	   1,57	   13,31	50,93

Pseudodiploria strigosa	           1,99	         1,32	   9,74	   1,31	   11,93	62,86

Diploria labyrinthiformis	           0,00	         1,40	   9,43	   1,40	   11,56	74,42

Porites astreoides	           0,52	         0,68	   5,83	   1,10	    7,14	81,56

Groups Cominoto  &  Salinas

Average dissimilarity = 79,70

	Group Cominoto	Group Salinas	       	       	        	     

Species	      Av.Abund	     Av.Abund	Av.Diss	Diss/SD	Contrib%	Cum.%

Orbicella faveolata	          0,00	         2,24	  15,83	   1,92	   19,86	19,86

Siderastrea siderea	          0,00	         2,20	  15,24	   2,51	   19,13	38,99

Pseudodiploria strigosa	          2,96	         1,32	  12,25	   1,32	   15,37	54,35

Montastrea cavernosa	          0,00	         1,66	  10,50	   1,67	   13,17	67,53

Diploria labyrinthiformis	          0,00	         1,40	   9,10	   1,49	   11,42	78,95

Groups La Pecha  &  Salinas

Average dissimilarity = 97,95

	Group La Pecha	Group Salinas	       	       	        	     

Species	      Av.Abund	     Av.Abund	Av.Diss	Diss/SD	Contrib%	Cum.%

Orbicella faveolata	          0,00	         2,24	  21,09	   1,74	   21,54	21,54

Siderastrea siderea	          0,00	         2,20	  20,13	   2,39	   20,55	42,09

Montastrea cavernosa	          0,00	         1,66	  13,43	   1,66	   13,71	55,80

Diploria labyrinthiformis	          0,00	         1,40	  11,73	   1,44	   11,98	67,78

Pseudodiploria strigosa	          0,17	         1,32	  10,27	   1,58	   10,48	78,26

Groups Puerto Real  &  Salinas

Average dissimilarity = 76,93

	Group Puerto Real	Group Salinas	       	       	        	     

Species	         Av.Abund	     Av.Abund	Av.Diss	Diss/SD	Contrib%	Cum.%

Orbicella faveolata	             0,00	         2,24	  14,83	   1,94	   19,28	19,28

Siderastrea siderea	             0,00	         2,20	  14,31	   2,50	   18,60	37,88

Montastrea cavernosa	             0,00	         1,66	   9,91	   1,67	   12,88	50,76

Pseudodiploria strigosa	             2,60	         1,32	   9,69	   1,16	   12,59	63,36

Diploria labyrinthiformis	             0,00	         1,40	   8,58	   1,49	   11,15	74,51

Porites astreoides	             1,10	         0,68	   4,24	   1,32	    5,51	80,02

Groups Cayo Agua  &  Salinas

Average dissimilarity = 75,30

	Group Cayo Agua	Group Salinas	       	       	        	     

Species	       Av.Abund	     Av.Abund	Av.Diss	Diss/SD	Contrib%	Cum.%

Madracis mirabilis	           3,47	         0,00	  13,40	   4,03	   17,80	17,80

Orbicella annularis	           3,05	         0,35	  10,59	   2,34	   14,07	31,87

Siderastrea siderea	           0,00	         2,20	   8,51	   2,62	   11,30	43,17

Montastrea cavernosa	           0,17	         1,66	   5,80	   1,82	    7,70	50,87

Diploria labyrinthiformis	           0,00	         1,40	   5,24	   1,55	    6,96	57,83

Pseudodiploria strigosa	           0,00	         1,32	   4,90	   1,53	    6,51	64,34

Orbicella faveolata	           3,24	         2,24	   4,37	   1,30	    5,80	70,14

Madracis decactis	           1,10	         0,00	   4,12	   0,96	    5,47	75,61

Groups Boca de Cote  &  Salinas

Average dissimilarity = 41,74

	Group Boca de Cote	Group Salinas	       	       	        	     

Species	          Av.Abund	     Av.Abund	Av.Diss	Diss/SD	Contrib%	Cum.%

Porites astreoides	              1,92	         0,68	   4,90	   1,62	   11,73	11,73

Orbicella faveolata	              3,10	         2,24	   4,40	   1,32	   10,55	22,27

Orbicella annularis	              1,35	         0,35	   4,39	   1,31	   10,51	32,78

Montastrea cavernosa	              1,76	         1,66	   3,84	   1,22	    9,20	41,98

Pseudodiploria strigosa	              0,62	         1,32	   3,83	   1,26	    9,17	51,15

Madracis mirabilis	              1,01	         0,00	   3,54	   0,73	    8,48	59,62

Siderastrea siderea	              2,54	         2,20	   3,06	   1,30	    7,32	66,94

Diploria labyrinthiformis	              1,37	         1,40	   2,90	   1,14	    6,96	73,90

Agaricia humilis	              0,62	         0,27	   2,27	   1,37	    5,44	79,34

Groups Dos Mosquises Sur  &  Salinas

Average dissimilarity = 70,41

	Group Dos Mosquises Sur	Group Salinas	       	       	        	     

Species	               Av.Abund	     Av.Abund	Av.Diss	Diss/SD	Contrib%	Cum.%

Madracis mirabilis	                   2,55	         0,00	   9,89	   1,59	   14,05	14,05

Colpophyllia natans	                   2,54	         0,27	   9,19	   3,29	   13,05	27,10

Orbicella annularis	                   2,43	         0,35	   8,91	   1,44	   12,66	39,76

Siderastrea siderea	                   0,52	         2,20	   6,87	   1,81	    9,75	49,51

Montastrea cavernosa	                   0,00	         1,66	   6,40	   1,65	    9,09	58,60

Orbicella faveolata	                   3,41	         2,24	   5,53	   1,26	    7,85	66,45

Diploria labyrinthiformis	                   0,00	         1,40	   5,49	   1,53	    7,80	74,25

Pseudodiploria strigosa	                   0,45	         1,32	   4,65	   1,35	    6,60	80,85

Groups Madrisqui  &  Salinas

Average dissimilarity = 69,54

	Group Madrisqui	Group Salinas	       	       	        	     

Species	       Av.Abund	     Av.Abund	Av.Diss	Diss/SD	Contrib%	Cum.%

Siderastrea siderea	           0,00	         2,20	  10,64	   2,54	   15,30	15,30

Colpophyllia natans	           1,96	         0,27	   8,51	   1,84	   12,23	27,53

Montastrea cavernosa	           0,52	         1,66	   6,50	   1,33	    9,35	36,88

Diploria labyrinthiformis	           0,00	         1,40	   6,49	   1,52	    9,33	46,21

Pseudodiploria strigosa	           0,00	         1,32	   6,05	   1,52	    8,71	54,92

Porites astreoides	           1,41	         0,68	   5,28	   1,40	    7,59	62,51

Orbicella faveolata	           2,67	         2,24	   4,44	   1,29	    6,39	68,89

Agaricia sp.	           0,93	         0,00	   4,10	   0,73	    5,89	74,79

Eusmilia fastigata	           0,68	         0,00	   3,11	   0,94	    4,48	79,26

Groups Pelona de Rabusqui  &  Salinas

Average dissimilarity = 70,70

	Group Pelona de Rabusqui	Group Salinas	       	       	        	     

Species	                Av.Abund	     Av.Abund	Av.Diss	Diss/SD	Contrib%	Cum.%

Orbicella faveolata	                    4,91	         2,24	  11,91	   2,57	   16,84	16,84

Siderastrea siderea	                    0,27	         2,20	   8,51	   1,94	   12,03	28,87

Orbicella annularis	                    2,00	         0,35	   8,12	   1,42	   11,48	40,35

Colpophyllia natans	                    1,87	         0,27	   7,07	   2,00	   10,00	50,35

Montastrea cavernosa	                    0,00	         1,66	   6,95	   1,65	    9,83	60,18

Diploria labyrinthiformis	                    0,00	         1,40	   5,97	   1,52	    8,45	68,63

Pseudodiploria strigosa	                    0,17	         1,32	   5,28	   1,52	    7,47	76,10

Groups La Pared  &  La Venada

Average dissimilarity = 94,79

	Group La Pared	Group La Venada	       	       	        	     

Species	      Av.Abund	       Av.Abund	Av.Diss	Diss/SD	Contrib%	Cum.%

Acropora cervicornis	          0,00	           4,54	  28,73	   7,10	   30,31	30,31

Pseudodiploria strigosa	          2,33	           0,27	  13,27	   2,09	   14,00	44,31

Meandrina meandrites	          1,94	           0,00	  11,57	   1,55	   12,21	56,51

Siderastrea siderea	          1,37	           0,00	   8,72	   4,74	    9,20	65,72

Montastrea cavernosa	          1,38	           0,00	   8,20	   1,55	    8,65	74,37

Diploria labyrinthiformis	          0,72	           0,00	   4,69	   0,95	    4,94	79,31

Groups Media Legua  &  La Venada

Average dissimilarity = 94,82

	Group Media Legua	Group La Venada	       	       	        	     

Species	         Av.Abund	       Av.Abund	Av.Diss	Diss/SD	Contrib%	Cum.%

Acropora cervicornis	             0,00	           4,54	  42,39	   7,62	   44,71	44,71

Pseudodiploria strigosa	             2,20	           0,27	  18,24	   2,48	   19,23	63,94

Siderastrea siderea	             0,85	           0,00	   7,85	   1,45	    8,28	72,22

Meandrina meandrites	             0,60	           0,00	   5,64	   0,56	    5,94	78,17

Groups Punta de Media Legua  &  La Venada

Average dissimilarity = 97,00

	Group Punta de Media Legua	Group La Venada	       	       	        	     

Species	                  Av.Abund	       Av.Abund	Av.Diss	Diss/SD	Contrib%	Cum.%

Acropora cervicornis	                      0,00	           4,54	  41,93	   2,73	   43,23	43,23

Siderastrea siderea	                      1,59	           0,00	  11,90	   3,15	   12,27	55,50

Pseudodiploria strigosa	                      1,63	           0,27	  11,76	   1,55	   12,13	67,62

Montastrea cavernosa	                      1,14	           0,00	   8,99	   1,37	    9,26	76,89

Groups Petaquire  &  La Venada

Average dissimilarity = 94,78

	Group Petaquire	Group La Venada	       	       	        	     

Species	       Av.Abund	       Av.Abund	Av.Diss	Diss/SD	Contrib%	Cum.%

Acropora cervicornis	           0,00	           4,54	  42,20	   4,37	   44,53	44,53

Pseudodiploria strigosa	           2,75	           0,27	  22,71	   3,22	   23,97	68,49

Siderastrea siderea	           0,92	           0,00	   8,21	   3,89	    8,66	77,15

Groups Playa Tiburon  &  La Venada

Average dissimilarity = 93,91

	Group Playa Tiburon	Group La Venada	       	       	        	     

Species	           Av.Abund	       Av.Abund	Av.Diss	Diss/SD	Contrib%	Cum.%

Acropora cervicornis	               0,00	           4,54	  33,01	   6,44	   35,15	35,15

Pseudodiploria strigosa	               2,38	           0,27	  15,39	   2,61	   16,39	51,54

Siderastrea siderea	               1,64	           0,00	  11,84	   2,52	   12,61	64,15

Diploria labyrinthiformis	               1,38	           0,00	   9,76	   2,91	   10,39	74,55

Montastrea cavernosa	               0,93	           0,00	   6,70	   1,33	    7,13	81,68

Groups Punta Mono  &  La Venada

Average dissimilarity = 95,76

	Group Punta Mono	Group La Venada	       	       	        	     

Species	        Av.Abund	       Av.Abund	Av.Diss	Diss/SD	Contrib%	Cum.%

Acropora cervicornis	            0,00	           4,54	  34,33	   5,18	   35,85	35,85

Pseudodiploria strigosa	            2,96	           0,27	  19,80	   3,71	   20,68	56,53

Siderastrea siderea	            1,34	           0,00	   9,95	   3,61	   10,39	66,92

Meandrina meandrites	            1,21	           0,00	   9,17	   1,63	    9,58	76,50

Groups Charagato  &  La Venada

Average dissimilarity = 99,00

	Group Charagato	Group La Venada	       	       	        	     

Species	       Av.Abund	       Av.Abund	Av.Diss	Diss/SD	Contrib%	Cum.%

Acropora cervicornis	           0,00	           4,54	  60,09	   3,18	   60,70	60,70

Colpophyllia natans	           1,86	           0,00	  20,00	   1,51	   20,20	80,91

Groups Punta Conejo  &  La Venada

Average dissimilarity = 96,03

	Group Punta Conejo	Group La Venada	       	       	        	     

Species	          Av.Abund	       Av.Abund	Av.Diss	Diss/SD	Contrib%	Cum.%

Acropora cervicornis	              0,00	           4,54	  58,83	   3,71	   61,26	61,26

Pseudodiploria strigosa	              1,99	           0,27	  20,36	   1,38	   21,20	82,46

Groups La Muerta  &  La Venada

Average dissimilarity = 96,46

	Group La Muerta	Group La Venada	       	       	        	     

Species	       Av.Abund	       Av.Abund	Av.Diss	Diss/SD	Contrib%	Cum.%

Acropora cervicornis	           0,00	           4,54	  56,08	   2,73	   58,14	58,14

Pseudodiploria strigosa	           1,99	           0,27	  18,97	   1,66	   19,67	77,81

Groups Cominoto  &  La Venada

Average dissimilarity = 94,94

	Group Cominoto	Group La Venada	       	       	        	     

Species	      Av.Abund	       Av.Abund	Av.Diss	Diss/SD	Contrib%	Cum.%

Acropora cervicornis	          0,00	           4,54	  50,48	   8,74	   53,17	53,17

Pseudodiploria strigosa	          2,96	           0,27	  30,51	   3,01	   32,14	85,30

Groups La Pecha  &  La Venada

Average dissimilarity = 98,92

	Group La Pecha	Group La Venada	       	       	        	     

Species	      Av.Abund	       Av.Abund	Av.Diss	Diss/SD	Contrib%	Cum.%

Acropora cervicornis	          0,00	           4,54	  81,56	   5,90	   82,45	82,45

Groups Puerto Real  &  La Venada

Average dissimilarity = 94,56

	Group Puerto Real	Group La Venada	       	       	        	     

Species	         Av.Abund	       Av.Abund	Av.Diss	Diss/SD	Contrib%	Cum.%

Acropora cervicornis	             0,00	           4,54	  45,83	   7,63	   48,47	48,47

Pseudodiploria strigosa	             2,60	           0,27	  23,20	   2,27	   24,53	73,01

Porites astreoides	             1,10	           0,00	  11,11	   7,56	   11,75	84,76

Groups Cayo Agua  &  La Venada

Average dissimilarity = 96,03

	Group Cayo Agua	Group La Venada	       	       	        	     

Species	       Av.Abund	       Av.Abund	Av.Diss	Diss/SD	Contrib%	Cum.%

Acropora cervicornis	           0,35	           4,54	  20,74	   7,00	   21,60	21,60

Madracis mirabilis	           3,47	           0,00	  17,02	   4,39	   17,73	39,33

Orbicella faveolata	           3,24	           0,00	  16,10	   5,99	   16,77	56,10

Orbicella annularis	           3,05	           0,00	  14,92	   3,60	   15,54	71,63

Colpophyllia natans	           1,15	           0,00	   5,58	   1,27	    5,81	77,45

Groups Boca de Cote  &  La Venada

Average dissimilarity = 97,47

	Group Boca de Cote	Group La Venada	       	       	        	     

Species	          Av.Abund	       Av.Abund	Av.Diss	Diss/SD	Contrib%	Cum.%

Acropora cervicornis	              0,00	           4,54	  21,72	   8,53	   22,29	22,29

Orbicella faveolata	              3,10	           0,00	  14,76	   3,95	   15,15	37,43

Siderastrea siderea	              2,54	           0,00	  12,10	   4,77	   12,42	49,85

Porites astreoides	              1,92	           0,00	   9,25	   4,23	    9,49	59,34

Montastrea cavernosa	              1,76	           0,00	   8,28	   2,35	    8,50	67,84

Diploria labyrinthiformis	              1,37	           0,00	   6,71	   2,67	    6,88	74,72

Orbicella annularis	              1,35	           0,00	   6,15	   1,53	    6,31	81,03

Groups Dos Mosquises Sur  &  La Venada

Average dissimilarity = 99,35

	Group Dos Mosquises Sur	Group La Venada	       	       	        	     

Species	               Av.Abund	       Av.Abund	Av.Diss	Diss/SD	Contrib%	Cum.%

Acropora cervicornis	                   0,00	           4,54	  23,90	   7,69	   24,05	24,05

Orbicella faveolata	                   3,41	           0,00	  18,23	   3,14	   18,35	42,40

Colpophyllia natans	                   2,54	           0,00	  13,56	   3,82	   13,65	56,04

Madracis mirabilis	                   2,55	           0,00	  12,58	   1,61	   12,67	68,71

Orbicella annularis	                   2,43	           0,00	  12,03	   1,48	   12,11	80,82

Groups Madrisqui  &  La Venada

Average dissimilarity = 97,68

	Group Madrisqui	Group La Venada	       	       	        	     

Species	       Av.Abund	       Av.Abund	Av.Diss	Diss/SD	Contrib%	Cum.%

Acropora cervicornis	           0,00	           4,54	  30,05	   7,80	   30,76	30,76

Orbicella faveolata	           2,67	           0,00	  17,56	   4,84	   17,97	48,74

Colpophyllia natans	           1,96	           0,00	  12,38	   1,66	   12,67	61,41

Porites astreoides	           1,41	           0,00	   9,06	   1,58	    9,27	70,68

Agaricia sp.	           0,93	           0,17	   5,50	   0,82	    5,63	76,31

Groups Pelona de Rabusqui  &  La Venada

Average dissimilarity = 96,85

	Group Pelona de Rabusqui	Group La Venada	       	       	        	     

Species	                Av.Abund	       Av.Abund	Av.Diss	Diss/SD	Contrib%	Cum.%

Orbicella faveolata	                    4,91	           0,00	  29,17	   5,66	   30,11	30,11

Acropora cervicornis	                    0,00	           4,54	  26,81	   6,61	   27,68	57,79

Orbicella annularis	                    2,00	           0,00	  11,56	   1,52	   11,93	69,72

Colpophyllia natans	                    1,87	           0,00	  10,75	   2,28	   11,10	80,82

Groups Salinas  &  La Venada

Average dissimilarity = 97,17

	Group Salinas	Group La Venada	       	       	        	     

Species	     Av.Abund	       Av.Abund	Av.Diss	Diss/SD	Contrib%	Cum.%

Acropora cervicornis	         0,00	           4,54	  27,72	   5,74	   28,53	28,53

Orbicella faveolata	         2,24	           0,00	  13,85	   1,97	   14,25	42,78

Siderastrea siderea	         2,20	           0,00	  13,37	   2,51	   13,76	56,54

Montastrea cavernosa	         1,66	           0,00	   9,32	   1,66	    9,59	66,13

Diploria labyrinthiformis	         1,40	           0,00	   8,06	   1,50	    8,29	74,42

Pseudodiploria strigosa	         1,32	           0,27	   6,87	   1,44	    7,07	81,49

Groups La Pared  &  San Agustin

Average dissimilarity = 97,85

	Group La Pared	Group San Agustin	       	       	        	     

Species	      Av.Abund	         Av.Abund	Av.Diss	Diss/SD	Contrib%	Cum.%

Pseudodiploria strigosa	          2,33	             0,00	  22,71	   2,53	   23,21	23,21

Meandrina meandrites	          1,94	             0,00	  16,96	   1,54	   17,34	40,54

Siderastrea siderea	          1,37	             0,00	  13,35	   3,92	   13,64	54,18

Montastrea cavernosa	          1,38	             0,17	  11,45	   1,63	   11,70	65,89

Diploria labyrinthiformis	          0,72	             0,00	   7,27	   0,97	    7,43	73,31

Other (Scleractinian massive)	          0,62	             0,00	   6,68	   0,88	    6,83	80,15

Groups Media Legua  &  San Agustin

Average dissimilarity = 97,34

	Group Media Legua	Group San Agustin	       	       	        	     

Species	         Av.Abund	         Av.Abund	Av.Diss	Diss/SD	Contrib%	Cum.%

Pseudodiploria strigosa	             2,20	             0,00	  41,01	   3,77	   42,13	42,13

Siderastrea siderea	             0,85	             0,00	  15,78	   1,46	   16,21	58,35

Meandrina meandrites	             0,60	             0,00	  11,25	   0,56	   11,56	69,90

Montastrea cavernosa	             0,45	             0,17	   7,59	   1,03	    7,79	77,70

Groups Punta de Media Legua  &  San Agustin

Average dissimilarity = 96,36

	Group Punta de Media Legua	Group San Agustin	       	       	        	     

Species	                  Av.Abund	         Av.Abund	Av.Diss	Diss/SD	Contrib%	Cum.%

Pseudodiploria strigosa	                      1,63	             0,00	  27,70	   1,18	   28,75	28,75

Siderastrea siderea	                      1,59	             0,00	  24,05	   4,77	   24,96	53,70

Montastrea cavernosa	                      1,14	             0,17	  17,12	   1,21	   17,76	71,47

Diploria labyrinthiformis	                      0,83	             0,00	   7,23	   0,80	    7,51	78,97

Groups Petaquire  &  San Agustin

Average dissimilarity = 98,34

	Group Petaquire	Group San Agustin	       	       	        	     

Species	       Av.Abund	         Av.Abund	Av.Diss	Diss/SD	Contrib%	Cum.%

Pseudodiploria strigosa	           2,75	             0,00	  51,90	   3,12	   52,77	52,77

Siderastrea siderea	           0,92	             0,00	  16,81	   3,37	   17,09	69,87

Siderastrea sp.	           0,37	             0,00	   5,70	   0,68	    5,79	75,66

Groups Playa Tiburon  &  San Agustin

Average dissimilarity = 97,05

	Group Playa Tiburon	Group San Agustin	       	       	        	     

Species	           Av.Abund	         Av.Abund	Av.Diss	Diss/SD	Contrib%	Cum.%

Pseudodiploria strigosa	               2,38	             0,00	  28,26	   4,02	   29,12	29,12

Siderastrea siderea	               1,64	             0,00	  19,58	   2,50	   20,17	49,29

Diploria labyrinthiformis	               1,38	             0,00	  15,83	   3,22	   16,31	65,60

Montastrea cavernosa	               0,93	             0,17	  10,10	   1,27	   10,41	76,00

Groups Punta Mono  &  San Agustin

Average dissimilarity = 99,18

	Group Punta Mono	Group San Agustin	       	       	        	     

Species	        Av.Abund	         Av.Abund	Av.Diss	Diss/SD	Contrib%	Cum.%

Pseudodiploria strigosa	            2,96	             0,00	  36,38	  14,44	   36,68	36,68

Siderastrea siderea	            1,34	             0,00	  16,96	   3,38	   17,10	53,78

Meandrina meandrites	            1,21	             0,00	  15,88	   1,66	   16,01	69,79

Diploria labyrinthiformis	            1,02	             0,00	  12,65	   3,61	   12,76	82,55

Groups Charagato  &  San Agustin

Average dissimilarity = 100,00

	Group Charagato	Group San Agustin	       	       	        	     

Species	       Av.Abund	         Av.Abund	Av.Diss	Diss/SD	Contrib%	Cum.%

Colpophyllia natans	           1,86	             0,00	  65,53	   2,44	   65,53	65,53

Montastrea cavernosa	           0,00	             0,17	  12,10	   0,43	   12,10	77,63

Groups Punta Conejo  &  San Agustin

Average dissimilarity = 100,00

	Group Punta Conejo	Group San Agustin	       	       	        	     

Species	          Av.Abund	         Av.Abund	Av.Diss	Diss/SD	Contrib%	Cum.%

Pseudodiploria strigosa	              1,99	             0,00	  59,93	   1,59	   59,93	59,93

Oculina diffusa	              0,17	             0,00	  21,88	   0,54	   21,88	81,81

Groups La Muerta  &  San Agustin

Average dissimilarity = 100,00

	Group La Muerta	Group San Agustin	       	       	        	     

Species	       Av.Abund	         Av.Abund	Av.Diss	Diss/SD	Contrib%	Cum.%

Pseudodiploria strigosa	           1,99	             0,00	  58,74	   2,07	   58,74	58,74

Colpophyllia natans	           0,62	             0,00	  14,68	   0,64	   14,68	73,42

Montastrea cavernosa	           0,00	             0,17	  11,26	   0,41	   11,26	84,68

Groups Cominoto  &  San Agustin

Average dissimilarity = 100,00

	Group Cominoto	Group San Agustin	       	       	        	     

Species	      Av.Abund	         Av.Abund	Av.Diss	Diss/SD	Contrib%	Cum.%

Pseudodiploria strigosa	          2,96	             0,00	  81,19	   4,53	   81,19	81,19

Groups La Pecha  &  San Agustin

Average dissimilarity = 100,00

	Group La Pecha	Group San Agustin	       	       	        	      

Species	      Av.Abund	         Av.Abund	Av.Diss	Diss/SD	Contrib%	 Cum.%

Pseudodiploria strigosa	          0,17	             0,00	  50,00	   1,00	   50,00	 50,00

Montastrea cavernosa	          0,00	             0,17	  50,00	   1,00	   50,00	100,00

Groups Puerto Real  &  San Agustin

Average dissimilarity = 100,00

	Group Puerto Real	Group San Agustin	       	       	        	     

Species	         Av.Abund	         Av.Abund	Av.Diss	Diss/SD	Contrib%	Cum.%

Pseudodiploria strigosa	             2,60	             0,00	  54,11	   4,54	   54,11	54,11

Porites astreoides	             1,10	             0,00	  24,37	   5,32	   24,37	78,48

Groups Cayo Agua  &  San Agustin

Average dissimilarity = 99,38

	Group Cayo Agua	Group San Agustin	       	       	        	     

Species	       Av.Abund	         Av.Abund	Av.Diss	Diss/SD	Contrib%	Cum.%

Madracis mirabilis	           3,47	             0,00	  23,09	   4,57	   23,24	23,24

Orbicella faveolata	           3,24	             0,00	  21,95	   5,52	   22,08	45,32

Orbicella annularis	           3,05	             0,00	  20,22	   3,72	   20,34	65,66

Colpophyllia natans	           1,15	             0,00	   7,55	   1,26	    7,59	73,26

Madracis decactis	           1,10	             0,00	   6,94	   0,97	    6,98	80,24

Groups Boca de Cote  &  San Agustin

Average dissimilarity = 97,83

	Group Boca de Cote	Group San Agustin	       	       	        	     

Species	          Av.Abund	         Av.Abund	Av.Diss	Diss/SD	Contrib%	Cum.%

Orbicella faveolata	              3,10	             0,00	  19,88	   3,84	   20,32	20,32

Siderastrea siderea	              2,54	             0,00	  16,31	   4,55	   16,67	36,99

Porites astreoides	              1,92	             0,00	  12,51	   4,02	   12,79	49,78

Montastrea cavernosa	              1,76	             0,17	  10,04	   1,94	   10,26	60,04

Diploria labyrinthiformis	              1,37	             0,00	   9,12	   2,54	    9,32	69,36

Orbicella annularis	              1,35	             0,00	   8,15	   1,54	    8,33	77,69

Groups Dos Mosquises Sur  &  San Agustin

Average dissimilarity = 100,00

	Group Dos Mosquises Sur	Group San Agustin	       	       	        	     

Species	               Av.Abund	         Av.Abund	Av.Diss	Diss/SD	Contrib%	Cum.%

Orbicella faveolata	                   3,41	             0,00	  25,71	   2,76	   25,71	25,71

Colpophyllia natans	                   2,54	             0,00	  19,10	   3,27	   19,10	44,80

Madracis mirabilis	                   2,55	             0,00	  17,12	   1,62	   17,12	61,92

Orbicella annularis	                   2,43	             0,00	  16,38	   1,48	   16,38	78,31

Groups Madrisqui  &  San Agustin

Average dissimilarity = 98,99

	Group Madrisqui	Group San Agustin	       	       	        	     

Species	       Av.Abund	         Av.Abund	Av.Diss	Diss/SD	Contrib%	Cum.%

Orbicella faveolata	           2,67	             0,00	  27,25	   4,69	   27,53	27,53

Colpophyllia natans	           1,96	             0,00	  18,75	   1,66	   18,94	46,47

Porites astreoides	           1,41	             0,00	  13,91	   1,59	   14,05	60,52

Agaricia sp.	           0,93	             0,00	   7,81	   0,76	    7,89	68,41

Montastrea cavernosa	           0,52	             0,17	   7,14	   0,68	    7,21	75,62

Groups Pelona de Rabusqui  &  San Agustin

Average dissimilarity = 100,00

	Group Pelona de Rabusqui	Group San Agustin	       	       	        	     

Species	                Av.Abund	         Av.Abund	Av.Diss	Diss/SD	Contrib%	Cum.%

Orbicella faveolata	                    4,91	             0,00	  43,18	   4,27	   43,18	43,18

Orbicella annularis	                    2,00	             0,00	  16,91	   1,53	   16,91	60,08

Colpophyllia natans	                    1,87	             0,00	  15,60	   2,33	   15,60	75,69

Groups Salinas  &  San Agustin

Average dissimilarity = 97,95

	Group Salinas	Group San Agustin	       	       	        	     

Species	     Av.Abund	         Av.Abund	Av.Diss	Diss/SD	Contrib%	Cum.%

Orbicella faveolata	         2,24	             0,00	  21,09	   1,74	   21,54	21,54

Siderastrea siderea	         2,20	             0,00	  20,13	   2,39	   20,55	42,09

Montastrea cavernosa	         1,66	             0,17	  12,90	   1,77	   13,17	55,26

Diploria labyrinthiformis	         1,40	             0,00	  11,73	   1,44	   11,98	67,24

Pseudodiploria strigosa	         1,32	             0,00	  10,80	   1,52	   11,02	78,26

Groups La Venada  &  San Agustin

Average dissimilarity = 100,00

	Group La Venada	Group San Agustin	       	       	        	     

Species	       Av.Abund	         Av.Abund	Av.Diss	Diss/SD	Contrib%	Cum.%

Acropora cervicornis	           4,54	             0,00	  81,56	   5,90	   81,56	81,56

Groups La Pared  &  Blanca

Average dissimilarity = 59,21

	Group La Pared	Group Blanca	       	       	        	     

Species	      Av.Abund	    Av.Abund	Av.Diss	Diss/SD	Contrib%	Cum.%

Colpophyllia natans	          0,00	        3,84	  17,08	   6,86	   28,85	28,85

Meandrina meandrites	          1,94	        0,35	   7,61	   1,54	   12,86	41,71

Dichocoenia stokesii	          0,00	        1,17	   4,95	   1,58	    8,36	50,08

Siderastrea siderea	          1,37	        0,52	   4,18	   1,46	    7,06	57,14

Montastrea cavernosa	          1,38	        1,70	   3,41	   0,98	    5,77	62,90

Pseudodiploria strigosa	          2,33	        2,94	   3,27	   1,05	    5,52	68,43

Diploria labyrinthiformis	          0,72	        0,00	   3,26	   0,94	    5,50	73,93

Other (Scleractinian massive)	          0,62	        0,35	   2,83	   1,29	    4,78	78,71

Groups Media Legua  &  Blanca

Average dissimilarity = 61,44

	Group Media Legua	Group Blanca	       	       	        	     

Species	         Av.Abund	    Av.Abund	Av.Diss	Diss/SD	Contrib%	Cum.%

Colpophyllia natans	             0,17	        3,84	  21,17	   5,52	   34,46	34,46

Montastrea cavernosa	             0,45	        1,70	   7,40	   1,87	   12,05	46,51

Dichocoenia stokesii	             0,00	        1,17	   6,34	   1,59	   10,31	56,82

Pseudodiploria strigosa	             2,20	        2,94	   4,64	   1,38	    7,54	64,37

Meandrina meandrites	             0,60	        0,35	   4,45	   0,77	    7,25	71,61

Siderastrea siderea	             0,85	        0,52	   4,13	   1,27	    6,73	78,34

Groups Punta de Media Legua  &  Blanca

Average dissimilarity = 64,76

	Group Punta de Media Legua	Group Blanca	       	       	        	     

Species	                  Av.Abund	    Av.Abund	Av.Diss	Diss/SD	Contrib%	Cum.%

Colpophyllia natans	                      0,17	        3,84	  20,75	   3,11	   32,05	32,05

Pseudodiploria strigosa	                      1,63	        2,94	   8,51	   1,10	   13,15	45,19

Siderastrea siderea	                      1,59	        0,52	   6,16	   1,53	    9,52	54,71

Dichocoenia stokesii	                      0,00	        1,17	   6,13	   1,49	    9,47	64,18

Montastrea cavernosa	                      1,14	        1,70	   4,70	   0,94	    7,26	71,44

Diploria labyrinthiformis	                      0,83	        0,00	   3,53	   0,76	    5,45	76,89

Groups Petaquire  &  Blanca

Average dissimilarity = 60,59

	Group Petaquire	Group Blanca	       	       	        	     

Species	       Av.Abund	    Av.Abund	Av.Diss	Diss/SD	Contrib%	Cum.%

Colpophyllia natans	           0,23	        3,84	  20,79	   3,99	   34,31	34,31

Montastrea cavernosa	           0,23	        1,70	   8,52	   2,36	   14,06	48,36

Dichocoenia stokesii	           0,00	        1,17	   6,27	   1,54	   10,36	58,72

Siderastrea siderea	           0,92	        0,52	   3,67	   1,38	    6,05	64,77

Pseudodiploria strigosa	           2,75	        2,94	   3,44	   1,56	    5,67	70,45

Porites astreoides	           0,00	        0,62	   3,44	   1,60	    5,67	76,12

Groups Playa Tiburon  &  Blanca

Average dissimilarity = 61,51

	Group Playa Tiburon	Group Blanca	       	       	        	     

Species	           Av.Abund	    Av.Abund	Av.Diss	Diss/SD	Contrib%	Cum.%

Colpophyllia natans	               0,00	        3,84	  18,80	   6,40	   30,56	30,56

Diploria labyrinthiformis	               1,38	        0,00	   6,62	   2,71	   10,76	41,32

Siderastrea siderea	               1,64	        0,52	   6,03	   1,43	    9,80	51,12

Dichocoenia stokesii	               0,00	        1,17	   5,42	   1,58	    8,82	59,94

Montastrea cavernosa	               0,93	        1,70	   4,54	   1,46	    7,38	67,32

Meandrina meandrites	               0,75	        0,35	   3,94	   0,97	    6,40	73,72

Pseudodiploria strigosa	               2,38	        2,94	   3,55	   1,35	    5,78	79,49

Groups Punta Mono  &  Blanca

Average dissimilarity = 62,85

	Group Punta Mono	Group Blanca	       	       	        	     

Species	        Av.Abund	    Av.Abund	Av.Diss	Diss/SD	Contrib%	Cum.%

Colpophyllia natans	            0,00	        3,84	  19,26	   5,76	   30,64	30,64

Montastrea cavernosa	            0,27	        1,70	   7,32	   2,05	   11,64	42,28

Dichocoenia stokesii	            0,00	        1,17	   5,55	   1,57	    8,82	51,11

Meandrina meandrites	            1,21	        0,35	   5,34	   1,43	    8,50	59,61

Diploria labyrinthiformis	            1,02	        0,00	   5,02	   2,92	    7,98	67,59

Siderastrea siderea	            1,34	        0,52	   4,80	   1,54	    7,64	75,23

Groups Charagato  &  Blanca

Average dissimilarity = 71,47

	Group Charagato	Group Blanca	       	       	        	     

Species	       Av.Abund	    Av.Abund	Av.Diss	Diss/SD	Contrib%	Cum.%

Pseudodiploria strigosa	           0,40	        2,94	  18,26	   2,27	   25,55	25,55

Colpophyllia natans	           1,86	        3,84	  15,14	   1,23	   21,19	46,74

Montastrea cavernosa	           0,00	        1,70	  11,91	   2,97	   16,66	63,40

Dichocoenia stokesii	           0,00	        1,17	   7,53	   1,54	   10,53	73,93

Porites astreoides	           0,00	        0,62	   4,16	   1,61	    5,82	79,76

Groups Punta Conejo  &  Blanca

Average dissimilarity = 74,50

	Group Punta Conejo	Group Blanca	       	       	        	     

Species	          Av.Abund	    Av.Abund	Av.Diss	Diss/SD	Contrib%	Cum.%

Colpophyllia natans	              0,00	        3,84	  26,54	   4,65	   35,63	35,63

Montastrea cavernosa	              0,00	        1,70	  11,84	   3,10	   15,89	51,51

Pseudodiploria strigosa	              1,99	        2,94	  10,83	   1,15	   14,54	66,05

Dichocoenia stokesii	              0,00	        1,17	   7,49	   1,56	   10,06	76,10

Groups La Muerta  &  Blanca

Average dissimilarity = 68,66

	Group La Muerta	Group Blanca	       	       	        	     

Species	       Av.Abund	    Av.Abund	Av.Diss	Diss/SD	Contrib%	Cum.%

Colpophyllia natans	           0,62	        3,84	  21,91	   2,13	   31,91	31,91

Montastrea cavernosa	           0,00	        1,70	  11,38	   2,86	   16,58	48,48

Pseudodiploria strigosa	           1,99	        2,94	   8,86	   0,88	   12,91	61,40

Dichocoenia stokesii	           0,00	        1,17	   7,22	   1,52	   10,51	71,91

Porites astreoides	           0,52	        0,62	   5,14	   1,69	    7,48	79,39

Groups Cominoto  &  Blanca

Average dissimilarity = 61,91

	Group Cominoto	Group Blanca	       	       	        	     

Species	      Av.Abund	    Av.Abund	Av.Diss	Diss/SD	Contrib%	Cum.%

Colpophyllia natans	          0,00	        3,84	  24,66	   6,32	   39,83	39,83

Montastrea cavernosa	          0,00	        1,70	  10,99	   3,55	   17,75	57,57

Dichocoenia stokesii	          0,00	        1,17	   7,00	   1,60	   11,31	68,88

Porites astreoides	          0,58	        0,62	   3,92	   1,35	    6,33	75,22

Groups La Pecha  &  Blanca

Average dissimilarity = 97,27

	Group La Pecha	Group Blanca	       	       	        	     

Species	      Av.Abund	    Av.Abund	Av.Diss	Diss/SD	Contrib%	Cum.%

Colpophyllia natans	          0,00	        3,84	  31,66	   5,21	   32,55	32,55

Pseudodiploria strigosa	          0,17	        2,94	  22,87	   3,70	   23,52	56,07

Montastrea cavernosa	          0,00	        1,70	  14,14	   3,21	   14,53	70,60

Dichocoenia stokesii	          0,00	        1,17	   8,82	   1,60	    9,07	79,67

Groups Puerto Real  &  Blanca

Average dissimilarity = 62,98

	Group Puerto Real	Group Blanca	       	       	        	     

Species	         Av.Abund	    Av.Abund	Av.Diss	Diss/SD	Contrib%	Cum.%

Colpophyllia natans	             0,00	        3,84	  23,26	   6,29	   36,93	36,93

Montastrea cavernosa	             0,00	        1,70	  10,36	   3,57	   16,45	53,38

Dichocoenia stokesii	             0,00	        1,17	   6,63	   1,59	   10,52	63,90

Pseudodiploria strigosa	             2,60	        2,94	   5,74	   1,58	    9,11	73,01

Porites astreoides	             1,10	        0,62	   3,00	   1,24	    4,77	77,78

Groups Cayo Agua  &  Blanca

Average dissimilarity = 88,06

	Group Cayo Agua	Group Blanca	       	       	        	     

Species	       Av.Abund	    Av.Abund	Av.Diss	Diss/SD	Contrib%	Cum.%

Madracis mirabilis	           3,47	        0,00	  12,80	   4,11	   14,54	14,54

Orbicella faveolata	           3,24	        0,00	  12,07	   5,86	   13,71	28,25

Orbicella annularis	           3,05	        0,00	  11,23	   3,43	   12,76	41,01

Pseudodiploria strigosa	           0,00	        2,94	  10,92	   6,32	   12,40	53,40

Colpophyllia natans	           1,15	        3,84	  10,06	   2,69	   11,42	64,82

Montastrea cavernosa	           0,17	        1,70	   5,66	   3,19	    6,42	71,25

Dichocoenia stokesii	           0,00	        1,17	   4,17	   1,58	    4,73	75,98

Groups Boca de Cote  &  Blanca

Average dissimilarity = 75,91

	Group Boca de Cote	Group Blanca	       	       	        	     

Species	          Av.Abund	    Av.Abund	Av.Diss	Diss/SD	Contrib%	Cum.%

Colpophyllia natans	              0,17	        3,84	  13,36	   5,59	   17,60	17,60

Orbicella faveolata	              3,10	        0,00	  11,17	   3,88	   14,72	32,31

Pseudodiploria strigosa	              0,62	        2,94	   8,58	   2,55	   11,30	43,61

Siderastrea siderea	              2,54	        0,52	   7,45	   2,30	    9,81	53,43

Diploria labyrinthiformis	              1,37	        0,00	   5,04	   2,71	    6,64	60,07

Porites astreoides	              1,92	        0,62	   4,78	   2,22	    6,30	66,37

Orbicella annularis	              1,35	        0,00	   4,70	   1,52	    6,20	72,56

Dichocoenia stokesii	              0,00	        1,17	   4,07	   1,57	    5,37	77,93

Groups Dos Mosquises Sur  &  Blanca

Average dissimilarity = 73,37

	Group Dos Mosquises Sur	Group Blanca	       	       	        	     

Species	               Av.Abund	    Av.Abund	Av.Diss	Diss/SD	Contrib%	Cum.%

Orbicella faveolata	                   3,41	        0,00	  13,39	   3,33	   18,24	18,24

Pseudodiploria strigosa	                   0,45	        2,94	   9,73	   2,61	   13,26	31,50

Madracis mirabilis	                   2,55	        0,00	   9,45	   1,59	   12,88	44,38

Orbicella annularis	                   2,43	        0,00	   9,02	   1,47	   12,30	56,68

Montastrea cavernosa	                   0,00	        1,70	   6,64	   3,92	    9,05	65,74

Colpophyllia natans	                   2,54	        3,84	   4,99	   3,56	    6,80	72,54

Dichocoenia stokesii	                   0,00	        1,17	   4,36	   1,57	    5,94	78,48

Groups Madrisqui  &  Blanca

Average dissimilarity = 73,39

	Group Madrisqui	Group Blanca	       	       	        	     

Species	       Av.Abund	    Av.Abund	Av.Diss	Diss/SD	Contrib%	Cum.%

Pseudodiploria strigosa	           0,00	        2,94	  13,50	   5,46	   18,40	18,40

Orbicella faveolata	           2,67	        0,00	  12,18	   4,55	   16,59	34,99

Colpophyllia natans	           1,96	        3,84	   8,94	   1,40	   12,19	47,17

Montastrea cavernosa	           0,52	        1,70	   6,25	   2,07	    8,52	55,69

Dichocoenia stokesii	           0,00	        1,17	   5,11	   1,58	    6,96	62,65

Porites astreoides	           1,41	        0,62	   4,98	   1,84	    6,79	69,44

Agaricia sp.	           0,93	        0,00	   3,89	   0,73	    5,30	74,74

Eusmilia fastigata	           0,68	        0,00	   2,95	   0,95	    4,01	78,75

Groups Pelona de Rabusqui  &  Blanca

Average dissimilarity = 82,31

	Group Pelona de Rabusqui	Group Blanca	       	       	        	     

Species	                Av.Abund	    Av.Abund	Av.Diss	Diss/SD	Contrib%	Cum.%

Orbicella faveolata	                    4,91	        0,00	  20,81	   6,14	   25,29	25,29

Pseudodiploria strigosa	                    0,17	        2,94	  11,78	   4,00	   14,31	39,60

Colpophyllia natans	                    1,87	        3,84	   8,49	   1,92	   10,32	49,92

Orbicella annularis	                    2,00	        0,00	   8,31	   1,50	   10,10	60,01

Montastrea cavernosa	                    0,00	        1,70	   7,22	   3,77	    8,77	68,79

Dichocoenia stokesii	                    0,00	        1,17	   4,72	   1,57	    5,73	74,52

Agaricia sp.	                    1,10	        0,00	   4,67	   1,45	    5,67	80,19

Groups Salinas  &  Blanca

Average dissimilarity = 68,31

	Group Salinas	Group Blanca	       	       	        	     

Species	     Av.Abund	    Av.Abund	Av.Diss	Diss/SD	Contrib%	Cum.%

Colpophyllia natans	         0,27	        3,84	  15,26	   6,36	   22,34	22,34

Orbicella faveolata	         2,24	        0,00	   9,74	   2,09	   14,26	36,60

Siderastrea siderea	         2,20	        0,52	   7,59	   1,66	   11,11	47,71

Pseudodiploria strigosa	         1,32	        2,94	   7,30	   1,46	   10,69	58,40

Diploria labyrinthiformis	         1,40	        0,00	   5,80	   1,52	    8,49	66,89

Dichocoenia stokesii	         0,00	        1,17	   4,82	   1,56	    7,06	73,95

Montastrea cavernosa	         1,66	        1,70	   3,76	   1,18	    5,50	79,45

Groups La Venada  &  Blanca

Average dissimilarity = 97,18

	Group La Venada	Group Blanca	       	       	        	     

Species	       Av.Abund	    Av.Abund	Av.Diss	Diss/SD	Contrib%	Cum.%

Acropora cervicornis	           4,54	        0,00	  25,75	   6,81	   26,50	26,50

Colpophyllia natans	           0,00	        3,84	  21,85	   6,46	   22,49	48,98

Pseudodiploria strigosa	           0,27	        2,94	  15,31	   3,33	   15,76	64,74

Montastrea cavernosa	           0,00	        1,70	   9,73	   3,62	   10,01	74,75

Dichocoenia stokesii	           0,00	        1,17	   6,25	   1,59	    6,43	81,18

Groups San Agustin  &  Blanca

Average dissimilarity = 97,27

	Group San Agustin	Group Blanca	       	       	        	     

Species	         Av.Abund	    Av.Abund	Av.Diss	Diss/SD	Contrib%	Cum.%

Colpophyllia natans	             0,00	        3,84	  31,66	   5,21	   32,55	32,55

Pseudodiploria strigosa	             0,00	        2,94	  24,24	   4,35	   24,92	57,47

Montastrea cavernosa	             0,17	        1,70	  12,77	   2,51	   13,13	70,60

Dichocoenia stokesii	             0,00	        1,17	   8,82	   1,60	    9,07	79,67

Groups La Pared  &  Carabela

Average dissimilarity = 97,85

	Group La Pared	Group Carabela	       	       	        	     

Species	      Av.Abund	      Av.Abund	Av.Diss	Diss/SD	Contrib%	Cum.%

Pseudodiploria strigosa	          2,33	          0,00	  21,48	   2,49	   21,95	21,95

Meandrina meandrites	          1,94	          0,00	  16,15	   1,52	   16,50	38,46

Siderastrea siderea	          1,37	          0,00	  12,63	   3,76	   12,91	51,36

Montastrea cavernosa	          1,38	          0,17	  10,87	   1,63	   11,11	62,48

Diploria labyrinthiformis	          0,72	          0,00	   6,86	   0,96	    7,02	69,49

Other (Scleractinian massive)	          0,62	          0,00	   6,29	   0,88	    6,43	75,92

Groups Media Legua  &  Carabela

Average dissimilarity = 96,27

	Group Media Legua	Group Carabela	       	       	        	     

Species	         Av.Abund	      Av.Abund	Av.Diss	Diss/SD	Contrib%	Cum.%

Pseudodiploria strigosa	             2,20	          0,00	  37,39	   3,28	   38,84	38,84

Siderastrea siderea	             0,85	          0,00	  14,39	   1,41	   14,94	53,79

Meandrina meandrites	             0,60	          0,00	  10,27	   0,55	   10,67	64,46

Colpophyllia natans	             0,17	          0,66	  10,20	   0,73	   10,59	75,05

Groups Punta de Media Legua  &  Carabela

Average dissimilarity = 95,77

	Group Punta de Media Legua	Group Carabela	       	       	        	     

Species	                  Av.Abund	      Av.Abund	Av.Diss	Diss/SD	Contrib%	Cum.%

Pseudodiploria strigosa	                      1,63	          0,00	  24,75	   1,17	   25,84	25,84

Siderastrea siderea	                      1,59	          0,00	  21,76	   3,76	   22,72	48,56

Montastrea cavernosa	                      1,14	          0,17	  15,62	   1,22	   16,31	64,87

Colpophyllia natans	                      0,17	          0,66	   9,47	   0,56	    9,89	74,76

Diploria labyrinthiformis	                      0,83	          0,00	   6,89	   0,79	    7,19	81,95

Groups Petaquire  &  Carabela

Average dissimilarity = 97,22

	Group Petaquire	Group Carabela	       	       	        	     

Species	       Av.Abund	      Av.Abund	Av.Diss	Diss/SD	Contrib%	Cum.%

Pseudodiploria strigosa	           2,75	          0,00	  46,97	   2,86	   48,31	48,31

Siderastrea siderea	           0,92	          0,00	  15,25	   3,00	   15,69	64,00

Colpophyllia natans	           0,23	          0,66	  10,12	   0,68	   10,41	74,41

Siderastrea sp.	           0,37	          0,00	   5,26	   0,67	    5,41	79,83

Groups Playa Tiburon  &  Carabela

Average dissimilarity = 97,05

	Group Playa Tiburon	Group Carabela	       	       	        	     

Species	           Av.Abund	      Av.Abund	Av.Diss	Diss/SD	Contrib%	Cum.%

Pseudodiploria strigosa	               2,38	          0,00	  26,47	   3,70	   27,27	27,27

Siderastrea siderea	               1,64	          0,00	  18,33	   2,41	   18,88	46,15

Diploria labyrinthiformis	               1,38	          0,00	  14,86	   3,01	   15,31	61,46

Montastrea cavernosa	               0,93	          0,17	   9,39	   1,26	    9,67	71,14

Meandrina meandrites	               0,75	          0,00	   7,36	   0,80	    7,59	78,72

Groups Punta Mono  &  Carabela

Average dissimilarity = 99,18

	Group Punta Mono	Group Carabela	       	       	        	     

Species	        Av.Abund	      Av.Abund	Av.Diss	Diss/SD	Contrib%	Cum.%

Pseudodiploria strigosa	            2,96	          0,00	  33,96	   7,56	   34,25	34,25

Siderastrea siderea	            1,34	          0,00	  15,80	   3,18	   15,93	50,18

Meandrina meandrites	            1,21	          0,00	  14,77	   1,62	   14,89	65,07

Diploria labyrinthiformis	            1,02	          0,00	  11,81	   3,31	   11,91	76,98

Groups Charagato  &  Carabela

Average dissimilarity = 84,82

	Group Charagato	Group Carabela	       	       	        	     

Species	       Av.Abund	      Av.Abund	Av.Diss	Diss/SD	Contrib%	Cum.%

Colpophyllia natans	           1,86	          0,66	  55,03	   1,61	   64,88	64,88

Montastrea cavernosa	           0,00	          0,17	  11,23	   0,42	   13,24	78,13

Groups Punta Conejo  &  Carabela

Average dissimilarity = 100,00

	Group Punta Conejo	Group Carabela	       	       	        	     

Species	          Av.Abund	      Av.Abund	Av.Diss	Diss/SD	Contrib%	Cum.%

Pseudodiploria strigosa	              1,99	          0,00	  51,89	   1,45	   51,89	51,89

Oculina diffusa	              0,17	          0,00	  16,93	   0,48	   16,93	68,81

Colpophyllia natans	              0,00	          0,66	  14,03	   0,52	   14,03	82,85

Groups La Muerta  &  Carabela

Average dissimilarity = 95,38

	Group La Muerta	Group Carabela	       	       	        	     

Species	       Av.Abund	      Av.Abund	Av.Diss	Diss/SD	Contrib%	Cum.%

Pseudodiploria strigosa	           1,99	          0,00	  48,32	   1,65	   50,66	50,66

Colpophyllia natans	           0,62	          0,66	  23,40	   0,74	   24,53	75,19

Groups Cominoto  &  Carabela

Average dissimilarity = 100,00

	Group Cominoto	Group Carabela	       	       	        	     

Species	      Av.Abund	      Av.Abund	Av.Diss	Diss/SD	Contrib%	Cum.%

Pseudodiploria strigosa	          2,96	          0,00	  72,05	   3,35	   72,05	72,05

Porites astreoides	          0,58	          0,00	  13,11	   0,84	   13,11	85,16

Groups La Pecha  &  Carabela

Average dissimilarity = 100,00

	Group La Pecha	Group Carabela	       	       	        	      

Species	      Av.Abund	      Av.Abund	Av.Diss	Diss/SD	Contrib%	 Cum.%

Colpophyllia natans	          0,00	          0,66	  37,92	   0,77	   37,92	 37,92

Montastrea cavernosa	          0,00	          0,17	  35,00	   0,74	   35,00	 72,92

Pseudodiploria strigosa	          0,17	          0,00	  27,08	   0,65	   27,08	100,00

Groups Puerto Real  &  Carabela

Average dissimilarity = 100,00

	Group Puerto Real	Group Carabela	       	       	        	     

Species	         Av.Abund	      Av.Abund	Av.Diss	Diss/SD	Contrib%	Cum.%

Pseudodiploria strigosa	             2,60	          0,00	  48,98	   3,50	   48,98	48,98

Porites astreoides	             1,10	          0,00	  21,96	   3,96	   21,96	70,94

Colpophyllia natans	             0,00	          0,66	   9,37	   0,55	    9,37	80,31

Groups Cayo Agua  &  Carabela

Average dissimilarity = 96,13

	Group Cayo Agua	Group Carabela	       	       	        	     

Species	       Av.Abund	      Av.Abund	Av.Diss	Diss/SD	Contrib%	Cum.%

Madracis mirabilis	           3,47	          0,00	  22,21	   4,37	   23,10	23,10

Orbicella faveolata	           3,24	          0,00	  21,09	   5,25	   21,94	45,05

Orbicella annularis	           3,05	          0,00	  19,45	   3,61	   20,23	65,27

Colpophyllia natans	           1,15	          0,66	   7,82	   1,33	    8,13	73,40

Madracis decactis	           1,10	          0,00	   6,69	   0,96	    6,96	80,36

Groups Boca de Cote  &  Carabela

Average dissimilarity = 97,42

	Group Boca de Cote	Group Carabela	       	       	        	     

Species	          Av.Abund	      Av.Abund	Av.Diss	Diss/SD	Contrib%	Cum.%

Orbicella faveolata	              3,10	          0,00	  19,14	   3,74	   19,65	19,65

Siderastrea siderea	              2,54	          0,00	  15,70	   4,41	   16,11	35,76

Porites astreoides	              1,92	          0,00	  12,04	   3,92	   12,35	48,11

Montastrea cavernosa	              1,76	          0,17	   9,63	   1,93	    9,88	58,00

Diploria labyrinthiformis	              1,37	          0,00	   8,77	   2,52	    9,00	67,00

Orbicella annularis	              1,35	          0,00	   7,86	   1,53	    8,07	75,06

Groups Dos Mosquises Sur  &  Carabela

Average dissimilarity = 92,40

	Group Dos Mosquises Sur	Group Carabela	       	       	        	     

Species	               Av.Abund	      Av.Abund	Av.Diss	Diss/SD	Contrib%	Cum.%

Orbicella faveolata	                   3,41	          0,00	  24,59	   2,74	   26,61	26,61

Madracis mirabilis	                   2,55	          0,00	  16,46	   1,61	   17,81	44,43

Orbicella annularis	                   2,43	          0,00	  15,75	   1,47	   17,04	61,47

Colpophyllia natans	                   2,54	          0,66	  14,78	   1,62	   16,00	77,47

Groups Madrisqui  &  Carabela

Average dissimilarity = 91,64

	Group Madrisqui	Group Carabela	       	       	        	     

Species	       Av.Abund	      Av.Abund	Av.Diss	Diss/SD	Contrib%	Cum.%

Orbicella faveolata	           2,67	          0,00	  25,74	   4,31	   28,09	28,09

Colpophyllia natans	           1,96	          0,66	  15,83	   1,30	   17,27	45,36

Porites astreoides	           1,41	          0,00	  13,15	   1,56	   14,35	59,71

Agaricia sp.	           0,93	          0,00	   7,45	   0,75	    8,13	67,84

Montastrea cavernosa	           0,52	          0,17	   6,73	   0,69	    7,34	75,18

Groups Pelona de Rabusqui  &  Carabela

Average dissimilarity = 93,61

	Group Pelona de Rabusqui	Group Carabela	       	       	        	     

Species	                Av.Abund	      Av.Abund	Av.Diss	Diss/SD	Contrib%	Cum.%

Orbicella faveolata	                    4,91	          0,00	  41,04	   4,13	   43,83	43,83

Orbicella annularis	                    2,00	          0,00	  16,09	   1,51	   17,19	61,02

Colpophyllia natans	                    1,87	          0,66	  13,20	   1,66	   14,10	75,12

Groups Salinas  &  Carabela

Average dissimilarity = 96,66

	Group Salinas	Group Carabela	       	       	        	     

Species	     Av.Abund	      Av.Abund	Av.Diss	Diss/SD	Contrib%	Cum.%

Orbicella faveolata	         2,24	          0,00	  19,97	   1,73	   20,66	20,66

Siderastrea siderea	         2,20	          0,00	  19,09	   2,35	   19,74	40,40

Montastrea cavernosa	         1,66	          0,17	  12,28	   1,78	   12,70	53,11

Diploria labyrinthiformis	         1,40	          0,00	  11,18	   1,44	   11,56	64,67

Pseudodiploria strigosa	         1,32	          0,00	  10,30	   1,51	   10,65	75,32

Groups La Venada  &  Carabela

Average dissimilarity = 100,00

	Group La Venada	Group Carabela	       	       	        	     

Species	       Av.Abund	      Av.Abund	Av.Diss	Diss/SD	Contrib%	Cum.%

Acropora cervicornis	           4,54	          0,00	  74,58	   4,51	   74,58	74,58

Colpophyllia natans	           0,00	          0,66	   8,17	   0,55	    8,17	82,74

Groups San Agustin  &  Carabela

Average dissimilarity = 90,00

	Group San Agustin	Group Carabela	       	       	        	      

Species	         Av.Abund	      Av.Abund	Av.Diss	Diss/SD	Contrib%	 Cum.%

Montastrea cavernosa	             0,17	          0,17	  52,08	   1,02	   57,87	 57,87

Colpophyllia natans	             0,00	          0,66	  37,92	   0,77	   42,13	100,00

Groups Blanca  &  Carabela

Average dissimilarity = 88,29

	Group Blanca	Group Carabela	       	       	        	     

Species	    Av.Abund	      Av.Abund	Av.Diss	Diss/SD	Contrib%	Cum.%

Colpophyllia natans	        3,84	          0,66	  25,69	   2,20	   29,10	29,10

Pseudodiploria strigosa	        2,94	          0,00	  23,10	   4,18	   26,17	55,27

Montastrea cavernosa	        1,70	          0,17	  12,10	   2,53	   13,71	68,98

Dichocoenia stokesii	        1,17	          0,00	   8,44	   1,58	    9,56	78,54

Groups La Pared  &  Punta Cruz

Average dissimilarity = 72,09

	Group La Pared	Group Punta Cruz	       	       	        	     

Species	      Av.Abund	        Av.Abund	Av.Diss	Diss/SD	Contrib%	Cum.%

Meandrina meandrites	          1,94	            0,00	  11,63	   1,54	   16,14	16,14

Pseudodiploria strigosa	          2,33	            1,07	  10,54	   1,37	   14,62	30,76

Dichocoenia stokesii	          0,00	            1,29	   8,01	   1,59	   11,11	41,87

Siderastrea siderea	          1,37	            0,49	   7,39	   2,73	   10,25	52,12

Montastrea cavernosa	          1,38	            1,66	   6,11	   1,11	    8,48	60,59

Colpophyllia natans	          0,00	            0,85	   5,36	   1,28	    7,43	68,02

Diploria labyrinthiformis	          0,72	            0,00	   4,72	   0,95	    6,54	74,57

Other (Scleractinian massive)	          0,62	            0,00	   4,24	   0,90	    5,88	80,44

Groups Media Legua  &  Punta Cruz

Average dissimilarity = 70,53

	Group Media Legua	Group Punta Cruz	       	       	        	     

Species	         Av.Abund	        Av.Abund	Av.Diss	Diss/SD	Contrib%	Cum.%

Pseudodiploria strigosa	             2,20	            1,07	  14,75	   1,48	   20,91	20,91

Montastrea cavernosa	             0,45	            1,66	  12,12	   1,44	   17,19	38,10

Dichocoenia stokesii	             0,00	            1,29	  11,73	   1,59	   16,63	54,73

Siderastrea siderea	             0,85	            0,49	   8,66	   1,47	   12,28	67,01

Colpophyllia natans	             0,17	            0,85	   7,21	   1,19	   10,22	77,23

Groups Punta de Media Legua  &  Punta Cruz

Average dissimilarity = 68,39

	Group Punta de Media Legua	Group Punta Cruz	       	       	        	     

Species	                  Av.Abund	        Av.Abund	Av.Diss	Diss/SD	Contrib%	Cum.%

Pseudodiploria strigosa	                      1,63	            1,07	  11,74	   1,29	   17,17	17,17

Dichocoenia stokesii	                      0,00	            1,29	  11,60	   1,34	   16,96	34,12

Siderastrea siderea	                      1,59	            0,49	  11,38	   2,19	   16,65	50,77

Montastrea cavernosa	                      1,14	            1,66	   9,67	   0,95	   14,13	64,91

Colpophyllia natans	                      0,17	            0,85	   7,38	   1,00	   10,79	75,70

Groups Petaquire  &  Punta Cruz

Average dissimilarity = 72,34

	Group Petaquire	Group Punta Cruz	       	       	        	     

Species	       Av.Abund	        Av.Abund	Av.Diss	Diss/SD	Contrib%	Cum.%

Pseudodiploria strigosa	           2,75	            1,07	  18,11	   1,58	   25,03	25,03

Montastrea cavernosa	           0,23	            1,66	  13,55	   1,48	   18,72	43,76

Dichocoenia stokesii	           0,00	            1,29	  11,68	   1,49	   16,14	59,90

Siderastrea siderea	           0,92	            0,49	   8,82	   2,42	   12,19	72,09

Colpophyllia natans	           0,23	            0,85	   7,09	   1,07	    9,80	81,89

Groups Playa Tiburon  &  Punta Cruz

Average dissimilarity = 70,73

	Group Playa Tiburon	Group Punta Cruz	       	       	        	     

Species	           Av.Abund	        Av.Abund	Av.Diss	Diss/SD	Contrib%	Cum.%

Pseudodiploria strigosa	               2,38	            1,07	  12,20	   1,51	   17,25	17,25

Siderastrea siderea	               1,64	            0,49	  10,06	   2,03	   14,22	31,47

Diploria labyrinthiformis	               1,38	            0,00	   9,83	   2,84	   13,90	45,37

Dichocoenia stokesii	               0,00	            1,29	   9,18	   1,58	   12,97	58,34

Montastrea cavernosa	               0,93	            1,66	   7,40	   1,34	   10,46	68,80

Colpophyllia natans	               0,00	            0,85	   6,15	   1,26	    8,70	77,50

Groups Punta Mono  &  Punta Cruz

Average dissimilarity = 77,96

	Group Punta Mono	Group Punta Cruz	       	       	        	     

Species	        Av.Abund	        Av.Abund	Av.Diss	Diss/SD	Contrib%	Cum.%

Pseudodiploria strigosa	            2,96	            1,07	  15,56	   1,65	   19,96	19,96

Montastrea cavernosa	            0,27	            1,66	  11,15	   1,60	   14,30	34,27

Dichocoenia stokesii	            0,00	            1,29	   9,54	   1,54	   12,23	46,50

Meandrina meandrites	            1,21	            0,00	   9,25	   1,61	   11,87	58,37

Siderastrea siderea	            1,34	            0,49	   8,74	   2,57	   11,21	69,58

Diploria labyrinthiformis	            1,02	            0,00	   7,56	   3,16	    9,70	79,28

Groups Charagato  &  Punta Cruz

Average dissimilarity = 81,71

	Group Charagato	Group Punta Cruz	       	       	        	     

Species	       Av.Abund	        Av.Abund	Av.Diss	Diss/SD	Contrib%	Cum.%

Montastrea cavernosa	           0,00	            1,66	  22,38	   1,62	   27,39	27,39

Colpophyllia natans	           1,86	            0,85	  17,44	   1,48	   21,35	48,74

Dichocoenia stokesii	           0,00	            1,29	  16,54	   1,38	   20,24	68,98

Pseudodiploria strigosa	           0,40	            1,07	  12,08	   0,95	   14,79	83,77

Groups Punta Conejo  &  Punta Cruz

Average dissimilarity = 86,35

	Group Punta Conejo	Group Punta Cruz	       	       	        	     

Species	          Av.Abund	        Av.Abund	Av.Diss	Diss/SD	Contrib%	Cum.%

Montastrea cavernosa	              0,00	            1,66	  21,86	   1,72	   25,32	25,32

Pseudodiploria strigosa	              1,99	            1,07	  20,44	   1,37	   23,67	48,99

Dichocoenia stokesii	              0,00	            1,29	  16,19	   1,44	   18,75	67,74

Colpophyllia natans	              0,00	            0,85	  11,03	   1,11	   12,77	80,52

Groups La Muerta  &  Punta Cruz

Average dissimilarity = 82,62

	Group La Muerta	Group Punta Cruz	       	       	        	     

Species	       Av.Abund	        Av.Abund	Av.Diss	Diss/SD	Contrib%	Cum.%

Montastrea cavernosa	           0,00	            1,66	  20,87	   1,52	   25,26	25,26

Pseudodiploria strigosa	           1,99	            1,07	  18,21	   1,35	   22,04	47,29

Dichocoenia stokesii	           0,00	            1,29	  15,45	   1,32	   18,70	65,99

Colpophyllia natans	           0,62	            0,85	  12,49	   1,20	   15,11	81,11

Groups Cominoto  &  Punta Cruz

Average dissimilarity = 80,67

	Group Cominoto	Group Punta Cruz	       	       	        	     

Species	      Av.Abund	        Av.Abund	Av.Diss	Diss/SD	Contrib%	Cum.%

Pseudodiploria strigosa	          2,96	            1,07	  24,30	   1,47	   30,12	30,12

Montastrea cavernosa	          0,00	            1,66	  18,62	   2,04	   23,09	53,21

Dichocoenia stokesii	          0,00	            1,29	  13,92	   1,59	   17,26	70,47

Colpophyllia natans	          0,00	            0,85	   9,43	   1,22	   11,69	82,15

Groups La Pecha  &  Punta Cruz

Average dissimilarity = 97,69

	Group La Pecha	Group Punta Cruz	       	       	        	     

Species	      Av.Abund	        Av.Abund	Av.Diss	Diss/SD	Contrib%	Cum.%

Montastrea cavernosa	          0,00	            1,66	  30,72	   1,79	   31,45	31,45

Dichocoenia stokesii	          0,00	            1,29	  22,37	   1,50	   22,90	54,35

Colpophyllia natans	          0,00	            0,85	  15,41	   1,12	   15,77	70,12

Pseudodiploria strigosa	          0,17	            1,07	  15,38	   1,05	   15,74	85,86

Groups Puerto Real  &  Punta Cruz

Average dissimilarity = 84,03

	Group Puerto Real	Group Punta Cruz	       	       	        	     

Species	         Av.Abund	        Av.Abund	Av.Diss	Diss/SD	Contrib%	Cum.%

Pseudodiploria strigosa	             2,60	            1,07	  19,15	   1,47	   22,79	22,79

Montastrea cavernosa	             0,00	            1,66	  16,88	   2,05	   20,09	42,88

Dichocoenia stokesii	             0,00	            1,29	  12,67	   1,58	   15,07	57,95

Porites astreoides	             1,10	            0,00	  11,27	   5,81	   13,41	71,36

Colpophyllia natans	             0,00	            0,85	   8,55	   1,23	   10,18	81,54

Groups Cayo Agua  &  Punta Cruz

Average dissimilarity = 91,02

	Group Cayo Agua	Group Punta Cruz	       	       	        	     

Species	       Av.Abund	        Av.Abund	Av.Diss	Diss/SD	Contrib%	Cum.%

Madracis mirabilis	           3,47	            0,00	  17,09	   4,27	   18,77	18,77

Orbicella faveolata	           3,24	            0,17	  15,33	   4,62	   16,85	35,62

Orbicella annularis	           3,05	            0,00	  14,98	   3,53	   16,46	52,08

Montastrea cavernosa	           0,17	            1,66	   7,27	   1,88	    7,98	60,06

Dichocoenia stokesii	           0,00	            1,29	   6,27	   1,63	    6,89	66,95

Madracis decactis	           1,10	            0,00	   5,21	   0,96	    5,72	72,67

Pseudodiploria strigosa	           0,00	            1,07	   4,89	   0,88	    5,37	78,04

Groups Boca de Cote  &  Punta Cruz

Average dissimilarity = 77,68

	Group Boca de Cote	Group Punta Cruz	       	       	        	     

Species	          Av.Abund	        Av.Abund	Av.Diss	Diss/SD	Contrib%	Cum.%

Orbicella faveolata	              3,10	            0,17	  14,01	   3,35	   18,04	18,04

Siderastrea siderea	              2,54	            0,49	   9,77	   2,16	   12,58	30,62

Porites astreoides	              1,92	            0,00	   9,29	   4,12	   11,96	42,57

Diploria labyrinthiformis	              1,37	            0,00	   6,73	   2,63	    8,67	51,24

Orbicella annularis	              1,35	            0,00	   6,17	   1,53	    7,94	59,18

Dichocoenia stokesii	              0,00	            1,29	   6,08	   1,61	    7,83	67,01

Pseudodiploria strigosa	              0,62	            1,07	   4,91	   1,09	    6,31	73,33

Madracis mirabilis	              1,01	            0,00	   4,41	   0,74	    5,67	79,00

Groups Dos Mosquises Sur  &  Punta Cruz

Average dissimilarity = 85,84

	Group Dos Mosquises Sur	Group Punta Cruz	       	       	        	     

Species	               Av.Abund	        Av.Abund	Av.Diss	Diss/SD	Contrib%	Cum.%

Orbicella faveolata	                   3,41	            0,17	  17,43	   2,81	   20,30	20,30

Madracis mirabilis	                   2,55	            0,00	  12,64	   1,60	   14,72	35,02

Orbicella annularis	                   2,43	            0,00	  12,08	   1,47	   14,07	49,09

Colpophyllia natans	                   2,54	            0,85	   9,16	   1,93	   10,68	59,77

Montastrea cavernosa	                   0,00	            1,66	   8,75	   2,16	   10,19	69,96

Dichocoenia stokesii	                   0,00	            1,29	   6,68	   1,60	    7,78	77,74

Groups Madrisqui  &  Punta Cruz

Average dissimilarity = 83,96

	Group Madrisqui	Group Punta Cruz	       	       	        	     

Species	       Av.Abund	        Av.Abund	Av.Diss	Diss/SD	Contrib%	Cum.%

Orbicella faveolata	           2,67	            0,17	  16,57	   3,64	   19,74	19,74

Colpophyllia natans	           1,96	            0,85	  10,01	   1,96	   11,92	31,66

Montastrea cavernosa	           0,52	            1,66	   9,14	   1,71	   10,89	42,54

Porites astreoides	           1,41	            0,00	   9,11	   1,56	   10,85	53,40

Dichocoenia stokesii	           0,00	            1,29	   8,37	   1,60	    9,97	63,36

Pseudodiploria strigosa	           0,00	            1,07	   6,39	   0,88	    7,61	70,97

Agaricia sp.	           0,93	            0,00	   5,42	   0,74	    6,46	77,43

Groups Pelona de Rabusqui  &  Punta Cruz

Average dissimilarity = 87,51

	Group Pelona de Rabusqui	Group Punta Cruz	       	       	        	     

Species	                Av.Abund	        Av.Abund	Av.Diss	Diss/SD	Contrib%	Cum.%

Orbicella faveolata	                    4,91	            0,17	  28,34	   4,75	   32,38	32,38

Orbicella annularis	                    2,00	            0,00	  11,62	   1,51	   13,27	45,66

Montastrea cavernosa	                    0,00	            1,66	   9,82	   2,11	   11,22	56,88

Dichocoenia stokesii	                    0,00	            1,29	   7,48	   1,58	    8,55	65,42

Colpophyllia natans	                    1,87	            0,85	   7,02	   1,42	    8,02	73,44

Agaricia sp.	                    1,10	            0,00	   6,58	   1,45	    7,51	80,95

Groups Salinas  &  Punta Cruz

Average dissimilarity = 70,15

	Group Salinas	Group Punta Cruz	       	       	        	     

Species	     Av.Abund	        Av.Abund	Av.Diss	Diss/SD	Contrib%	Cum.%

Orbicella faveolata	         2,24	            0,17	  12,91	   1,74	   18,41	18,41

Siderastrea siderea	         2,20	            0,49	  11,03	   1,82	   15,72	34,12

Diploria labyrinthiformis	         1,40	            0,00	   8,10	   1,49	   11,54	45,67

Dichocoenia stokesii	         0,00	            1,29	   7,73	   1,56	   11,02	56,69

Pseudodiploria strigosa	         1,32	            1,07	   7,27	   1,31	   10,36	67,04

Montastrea cavernosa	         1,66	            1,66	   6,15	   1,13	    8,77	75,81

Groups La Venada  &  Punta Cruz

Average dissimilarity = 98,06

	Group La Venada	Group Punta Cruz	       	       	        	     

Species	       Av.Abund	        Av.Abund	Av.Diss	Diss/SD	Contrib%	Cum.%

Acropora cervicornis	           4,54	            0,00	  41,92	   6,13	   42,75	42,75

Montastrea cavernosa	           0,00	            1,66	  15,24	   2,07	   15,54	58,29

Dichocoenia stokesii	           0,00	            1,29	  11,47	   1,59	   11,70	69,99

Pseudodiploria strigosa	           0,27	            1,07	   8,71	   0,99	    8,88	78,87

Groups San Agustin  &  Punta Cruz

Average dissimilarity = 94,13

	Group San Agustin	Group Punta Cruz	       	       	        	     

Species	         Av.Abund	        Av.Abund	Av.Diss	Diss/SD	Contrib%	Cum.%

Montastrea cavernosa	             0,17	            1,66	  27,79	   1,52	   29,52	29,52

Dichocoenia stokesii	             0,00	            1,29	  22,37	   1,50	   23,76	53,28

Colpophyllia natans	             0,00	            0,85	  15,41	   1,12	   16,37	69,65

Pseudodiploria strigosa	             0,00	            1,07	  14,75	   0,91	   15,67	85,32

Groups Blanca  &  Punta Cruz

Average dissimilarity = 54,04

	Group Blanca	Group Punta Cruz	       	       	        	     

Species	    Av.Abund	        Av.Abund	Av.Diss	Diss/SD	Contrib%	Cum.%

Colpophyllia natans	        3,84	            0,85	  17,16	   3,45	   31,76	31,76

Pseudodiploria strigosa	        2,94	            1,07	  11,57	   1,49	   21,41	53,16

Dichocoenia stokesii	        1,17	            1,29	   4,72	   1,08	    8,73	61,90

Siderastrea siderea	        0,52	            0,49	   4,17	   0,94	    7,72	69,62

Montastrea cavernosa	        1,70	            1,66	   3,75	   1,37	    6,93	76,55

Groups Carabela  &  Punta Cruz

Average dissimilarity = 88,88

	Group Carabela	Group Punta Cruz	       	       	        	     

Species	      Av.Abund	        Av.Abund	Av.Diss	Diss/SD	Contrib%	Cum.%

Montastrea cavernosa	          0,17	            1,66	  25,05	   1,49	   28,18	28,18

Dichocoenia stokesii	          0,00	            1,29	  20,47	   1,47	   23,03	51,21

Colpophyllia natans	          0,66	            0,85	  17,14	   1,29	   19,29	70,50

Pseudodiploria strigosa	          0,00	            1,07	  13,72	   0,90	   15,43	85,93

Groups La Pared  &  Gabarra

Average dissimilarity = 71,06

	Group La Pared	Group Gabarra	       	       	        	     

Species	      Av.Abund	     Av.Abund	Av.Diss	Diss/SD	Contrib%	Cum.%

Meandrina meandrites	          1,94	         0,00	  13,06	   1,50	   18,38	18,38

Montastrea cavernosa	          1,38	         0,00	   9,25	   1,51	   13,02	31,40

Pseudodiploria strigosa	          2,33	         1,57	   7,98	   1,21	   11,23	42,63

Colpophyllia natans	          0,00	         1,17	   7,70	   1,27	   10,83	53,46

Siderastrea siderea	          1,37	         0,76	   6,85	   1,40	    9,64	63,11

Diploria labyrinthiformis	          0,72	         0,00	   5,38	   0,94	    7,58	70,68

Other (Scleractinian massive)	          0,62	         0,00	   4,87	   0,88	    6,85	77,54

Groups Media Legua  &  Gabarra

Average dissimilarity = 57,73

	Group Media Legua	Group Gabarra	       	       	        	     

Species	         Av.Abund	     Av.Abund	Av.Diss	Diss/SD	Contrib%	Cum.%

Pseudodiploria strigosa	             2,20	         1,57	  11,04	   1,07	   19,12	19,12

Colpophyllia natans	             0,17	         1,17	  10,98	   1,30	   19,02	38,14

Siderastrea siderea	             0,85	         0,76	   9,52	   1,27	   16,48	54,63

Meandrina meandrites	             0,60	         0,00	   7,03	   0,53	   12,18	66,81

Montastrea cavernosa	             0,45	         0,00	   4,75	   0,90	    8,23	75,03

Groups Punta de Media Legua  &  Gabarra

Average dissimilarity = 67,82

	Group Punta de Media Legua	Group Gabarra	       	       	        	     

Species	                  Av.Abund	     Av.Abund	Av.Diss	Diss/SD	Contrib%	Cum.%

Siderastrea siderea	                      1,59	         0,76	  12,65	   1,81	   18,65	18,65

Pseudodiploria strigosa	                      1,63	         1,57	  12,46	   1,37	   18,37	37,02

Montastrea cavernosa	                      1,14	         0,00	  11,03	   1,19	   16,27	53,29

Colpophyllia natans	                      0,17	         1,17	  11,03	   1,07	   16,26	69,55

Diploria labyrinthiformis	                      0,83	         0,00	   5,57	   0,77	    8,21	77,76

Groups Petaquire  &  Gabarra

Average dissimilarity = 55,25

	Group Petaquire	Group Gabarra	       	       	        	     

Species	       Av.Abund	     Av.Abund	Av.Diss	Diss/SD	Contrib%	Cum.%

Pseudodiploria strigosa	           2,75	         1,57	  14,53	   1,07	   26,31	26,31

Colpophyllia natans	           0,23	         1,17	  10,81	   1,18	   19,56	45,87

Siderastrea siderea	           0,92	         0,76	   9,42	   1,79	   17,04	62,91

Siderastrea sp.	           0,37	         0,00	   3,78	   0,65	    6,84	69,75

Diploria labyrinthiformis	           0,23	         0,00	   2,38	   0,65	    4,32	74,07

Montastrea cavernosa	           0,23	         0,00	   2,38	   0,65	    4,32	78,38

Groups Playa Tiburon  &  Gabarra

Average dissimilarity = 66,08

	Group Playa Tiburon	Group Gabarra	       	       	        	     

Species	           Av.Abund	     Av.Abund	Av.Diss	Diss/SD	Contrib%	Cum.%

Diploria labyrinthiformis	               1,38	         0,00	  11,37	   2,64	   17,21	17,21

Siderastrea siderea	               1,64	         0,76	  10,04	   1,27	   15,19	32,39

Pseudodiploria strigosa	               2,38	         1,57	   8,96	   1,22	   13,55	45,95

Colpophyllia natans	               0,00	         1,17	   8,89	   1,27	   13,45	59,40

Montastrea cavernosa	               0,93	         0,00	   7,85	   1,28	   11,88	71,28

Meandrina meandrites	               0,75	         0,00	   5,76	   0,78	    8,72	80,00

Groups Punta Mono  &  Gabarra

Average dissimilarity = 64,26

	Group Punta Mono	Group Gabarra	       	       	        	     

Species	        Av.Abund	     Av.Abund	Av.Diss	Diss/SD	Contrib%	Cum.%

Pseudodiploria strigosa	            2,96	         1,57	  12,95	   1,52	   20,15	20,15

Meandrina meandrites	            1,21	         0,00	  10,91	   1,54	   16,97	37,12

Colpophyllia natans	            0,00	         1,17	   9,26	   1,24	   14,42	51,53

Diploria labyrinthiformis	            1,02	         0,00	   8,85	   2,82	   13,77	65,30

Siderastrea siderea	            1,34	         0,76	   8,35	   1,29	   13,00	78,30

Groups Charagato  &  Gabarra

Average dissimilarity = 72,18

	Group Charagato	Group Gabarra	       	       	        	     

Species	       Av.Abund	     Av.Abund	Av.Diss	Diss/SD	Contrib%	Cum.%

Pseudodiploria strigosa	           0,40	         1,57	  28,34	   1,19	   39,26	39,26

Colpophyllia natans	           1,86	         1,17	  24,63	   1,13	   34,12	73,38

Siderastrea siderea	           0,27	         0,76	  10,90	   0,92	   15,10	88,48

Groups Punta Conejo  &  Gabarra

Average dissimilarity = 67,14

	Group Punta Conejo	Group Gabarra	       	       	        	     

Species	          Av.Abund	     Av.Abund	Av.Diss	Diss/SD	Contrib%	Cum.%

Pseudodiploria strigosa	              1,99	         1,57	  26,28	   1,58	   39,14	39,14

Colpophyllia natans	              0,00	         1,17	  16,34	   1,20	   24,34	63,47

Siderastrea siderea	              0,17	         0,76	  10,62	   0,97	   15,82	79,29

Groups La Muerta  &  Gabarra

Average dissimilarity = 64,89

	Group La Muerta	Group Gabarra	       	       	        	     

Species	       Av.Abund	     Av.Abund	Av.Diss	Diss/SD	Contrib%	Cum.%

Pseudodiploria strigosa	           1,99	         1,57	  25,69	   0,98	   39,60	39,60

Colpophyllia natans	           0,62	         1,17	  17,10	   1,12	   26,36	65,96

Siderastrea siderea	           0,00	         0,76	   9,79	   0,84	   15,09	81,04

Groups Cominoto  &  Gabarra

Average dissimilarity = 56,32

	Group Cominoto	Group Gabarra	       	       	        	     

Species	      Av.Abund	     Av.Abund	Av.Diss	Diss/SD	Contrib%	Cum.%

Pseudodiploria strigosa	          2,96	         1,57	  23,30	   1,12	   41,38	41,38

Colpophyllia natans	          0,00	         1,17	  13,86	   1,29	   24,60	65,98

Siderastrea siderea	          0,00	         0,76	   8,77	   0,92	   15,57	81,54

Groups La Pecha  &  Gabarra

Average dissimilarity = 89,07

	Group La Pecha	Group Gabarra	       	       	        	     

Species	      Av.Abund	     Av.Abund	Av.Diss	Diss/SD	Contrib%	Cum.%

Pseudodiploria strigosa	          0,17	         1,57	  44,87	   1,41	   50,38	50,38

Colpophyllia natans	          0,00	         1,17	  23,11	   1,28	   25,94	76,32

Groups Puerto Real  &  Gabarra

Average dissimilarity = 62,19

	Group Puerto Real	Group Gabarra	       	       	        	     

Species	         Av.Abund	     Av.Abund	Av.Diss	Diss/SD	Contrib%	Cum.%

Pseudodiploria strigosa	             2,60	         1,57	  16,74	   1,18	   26,92	26,92

Porites astreoides	             1,10	         0,17	  12,55	   1,87	   20,18	47,10

Colpophyllia natans	             0,00	         1,17	  12,52	   1,28	   20,13	67,23

Siderastrea siderea	             0,00	         0,76	   7,94	   0,91	   12,77	80,00

Groups Cayo Agua  &  Gabarra

Average dissimilarity = 92,60

	Group Cayo Agua	Group Gabarra	       	       	        	     

Species	       Av.Abund	     Av.Abund	Av.Diss	Diss/SD	Contrib%	Cum.%

Madracis mirabilis	           3,47	         0,00	  18,75	   4,00	   20,24	20,24

Orbicella faveolata	           3,24	         0,00	  17,76	   4,90	   19,18	39,42

Orbicella annularis	           3,05	         0,00	  16,42	   3,38	   17,74	57,16

Pseudodiploria strigosa	           0,00	         1,57	   8,28	   2,34	    8,94	66,10

Madracis decactis	           1,10	         0,00	   5,69	   0,96	    6,14	72,24

Colpophyllia natans	           1,15	         1,17	   5,58	   1,30	    6,03	78,26

Groups Boca de Cote  &  Gabarra

Average dissimilarity = 84,97

	Group Boca de Cote	Group Gabarra	       	       	        	     

Species	          Av.Abund	     Av.Abund	Av.Diss	Diss/SD	Contrib%	Cum.%

Orbicella faveolata	              3,10	         0,00	  16,22	   3,61	   19,09	19,09

Siderastrea siderea	              2,54	         0,76	   9,69	   1,70	   11,40	30,49

Porites astreoides	              1,92	         0,17	   9,35	   2,84	   11,00	41,49

Montastrea cavernosa	              1,76	         0,00	   9,09	   2,28	   10,70	52,19

Diploria labyrinthiformis	              1,37	         0,00	   7,39	   2,51	    8,70	60,89

Orbicella annularis	              1,35	         0,00	   6,72	   1,51	    7,91	68,80

Pseudodiploria strigosa	              0,62	         1,57	   6,00	   1,44	    7,06	75,86

Groups Dos Mosquises Sur  &  Gabarra

Average dissimilarity = 80,39

	Group Dos Mosquises Sur	Group Gabarra	       	       	        	     

Species	               Av.Abund	     Av.Abund	Av.Diss	Diss/SD	Contrib%	Cum.%

Orbicella faveolata	                   3,41	         0,00	  20,32	   2,79	   25,27	25,27

Madracis mirabilis	                   2,55	         0,00	  13,87	   1,58	   17,26	42,53

Orbicella annularis	                   2,43	         0,00	  13,26	   1,45	   16,50	59,03

Colpophyllia natans	                   2,54	         1,17	   8,74	   1,17	   10,88	69,91

Pseudodiploria strigosa	                   0,45	         1,57	   7,72	   1,81	    9,60	79,50

Groups Madrisqui  &  Gabarra

Average dissimilarity = 86,75

	Group Madrisqui	Group Gabarra	       	       	        	     

Species	       Av.Abund	     Av.Abund	Av.Diss	Diss/SD	Contrib%	Cum.%

Orbicella faveolata	           2,67	         0,00	  20,18	   3,78	   23,26	23,26

Pseudodiploria strigosa	           0,00	         1,57	  11,42	   2,37	   13,16	36,42

Colpophyllia natans	           1,96	         1,17	  10,62	   1,29	   12,24	48,66

Porites astreoides	           1,41	         0,17	   9,83	   1,51	   11,33	59,99

Agaricia sp.	           0,93	         0,00	   6,07	   0,74	    6,99	66,98

Siderastrea siderea	           0,00	         0,76	   5,16	   0,90	    5,95	72,93

Eusmilia fastigata	           0,68	         0,00	   4,74	   0,94	    5,46	78,39

Groups Pelona de Rabusqui  &  Gabarra

Average dissimilarity = 84,93

	Group Pelona de Rabusqui	Group Gabarra	       	       	        	     

Species	                Av.Abund	     Av.Abund	Av.Diss	Diss/SD	Contrib%	Cum.%

Orbicella faveolata	                    4,91	         0,00	  33,01	   4,09	   38,87	38,87

Orbicella annularis	                    2,00	         0,00	  13,03	   1,48	   15,34	54,21

Pseudodiploria strigosa	                    0,17	         1,57	   9,11	   1,77	   10,73	64,94

Colpophyllia natans	                    1,87	         1,17	   7,83	   1,22	    9,22	74,16

Agaricia sp.	                    1,10	         0,00	   7,40	   1,41	    8,71	82,87

Groups Salinas  &  Gabarra

Average dissimilarity = 75,27

	Group Salinas	Group Gabarra	       	       	        	     

Species	     Av.Abund	     Av.Abund	Av.Diss	Diss/SD	Contrib%	Cum.%

Orbicella faveolata	         2,24	         0,00	  15,81	   1,79	   21,01	21,01

Siderastrea siderea	         2,20	         0,76	  11,20	   1,36	   14,88	35,89

Montastrea cavernosa	         1,66	         0,00	  10,46	   1,61	   13,90	49,79

Diploria labyrinthiformis	         1,40	         0,00	   9,07	   1,44	   12,05	61,84

Colpophyllia natans	         0,27	         1,17	   7,01	   1,39	    9,32	71,16

Pseudodiploria strigosa	         1,32	         1,57	   6,74	   1,27	    8,95	80,11

Groups La Venada  &  Gabarra

Average dissimilarity = 95,54

	Group La Venada	Group Gabarra	       	       	        	     

Species	       Av.Abund	     Av.Abund	Av.Diss	Diss/SD	Contrib%	Cum.%

Acropora cervicornis	           4,54	         0,00	  51,42	   3,39	   53,82	53,82

Pseudodiploria strigosa	           0,27	         1,57	  14,57	   1,86	   15,25	69,08

Colpophyllia natans	           0,00	         1,17	  11,27	   1,28	   11,79	80,87

Groups San Agustin  &  Gabarra

Average dissimilarity = 100,00

	Group San Agustin	Group Gabarra	       	       	        	     

Species	         Av.Abund	     Av.Abund	Av.Diss	Diss/SD	Contrib%	Cum.%

Pseudodiploria strigosa	             0,00	         1,57	  50,34	   1,82	   50,34	50,34

Colpophyllia natans	             0,00	         1,17	  23,11	   1,28	   23,11	73,45

Siderastrea siderea	             0,00	         0,76	  14,37	   0,93	   14,37	87,82

Groups Blanca  &  Gabarra

Average dissimilarity = 62,66

	Group Blanca	Group Gabarra	       	       	        	     

Species	    Av.Abund	     Av.Abund	Av.Diss	Diss/SD	Contrib%	Cum.%

Colpophyllia natans	        3,84	         1,17	  17,69	   1,98	   28,24	28,24

Montastrea cavernosa	        1,70	         0,00	  10,95	   3,12	   17,47	45,71

Pseudodiploria strigosa	        2,94	         1,57	   9,17	   1,41	   14,63	60,34

Dichocoenia stokesii	        1,17	         0,00	   6,97	   1,55	   11,12	71,46

Siderastrea siderea	        0,52	         0,76	   4,78	   1,15	    7,62	79,09

Groups Carabela  &  Gabarra

Average dissimilarity = 92,26

	Group Carabela	Group Gabarra	       	       	        	     

Species	      Av.Abund	     Av.Abund	Av.Diss	Diss/SD	Contrib%	Cum.%

Pseudodiploria strigosa	          0,00	         1,57	  42,75	   1,65	   46,34	46,34

Colpophyllia natans	          0,66	         1,17	  24,81	   1,14	   26,90	73,24

Siderastrea siderea	          0,00	         0,76	  13,11	   0,91	   14,21	87,45

Groups Punta Cruz  &  Gabarra

Average dissimilarity = 73,07

	Group Punta Cruz	Group Gabarra	       	       	        	     

Species	        Av.Abund	     Av.Abund	Av.Diss	Diss/SD	Contrib%	Cum.%

Montastrea cavernosa	            1,66	         0,00	  19,04	   1,69	   26,06	26,06

Pseudodiploria strigosa	            1,07	         1,57	  14,53	   1,71	   19,89	45,95

Dichocoenia stokesii	            1,29	         0,00	  14,18	   1,42	   19,41	65,36

Colpophyllia natans	            0,85	         1,17	  10,48	   1,18	   14,35	79,70

Groups La Pared  &  Garrapata

Average dissimilarity = 58,29

	Group La Pared	Group Garrapata	       	       	        	     

Species	      Av.Abund	       Av.Abund	Av.Diss	Diss/SD	Contrib%	Cum.%

Colpophyllia natans	          0,00	           3,62	  16,68	   4,24	   28,61	28,61

Meandrina meandrites	          1,94	           0,00	   8,57	   1,57	   14,69	43,30

Montastrea cavernosa	          1,38	           2,71	   6,39	   1,27	   10,96	54,27

Pseudodiploria strigosa	          2,33	           3,23	   4,12	   1,10	    7,07	61,33

Siderastrea siderea	          1,37	           0,52	   3,95	   2,20	    6,78	68,11

Diploria labyrinthiformis	          0,72	           0,00	   3,38	   0,95	    5,79	73,91

Other (Scleractinian massive)	          0,62	           0,17	   2,97	   1,05	    5,09	79,00

Groups Media Legua  &  Garrapata

Average dissimilarity = 59,39

	Group Media Legua	Group Garrapata	       	       	        	     

Species	         Av.Abund	       Av.Abund	Av.Diss	Diss/SD	Contrib%	Cum.%

Colpophyllia natans	             0,17	           3,62	  20,75	   3,90	   34,93	34,93

Montastrea cavernosa	             0,45	           2,71	  13,71	   3,46	   23,09	58,03

Pseudodiploria strigosa	             2,20	           3,23	   6,23	   1,61	   10,49	68,51

Meandrina meandrites	             0,60	           0,00	   3,62	   0,56	    6,10	74,61

Siderastrea siderea	             0,85	           0,52	   3,50	   1,31	    5,89	80,50

Groups Punta de Media Legua  &  Garrapata

Average dissimilarity = 61,74

	Group Punta de Media Legua	Group Garrapata	       	       	        	     

Species	                  Av.Abund	       Av.Abund	Av.Diss	Diss/SD	Contrib%	Cum.%

Colpophyllia natans	                      0,17	           3,62	  20,35	   2,70	   32,96	32,96

Pseudodiploria strigosa	                      1,63	           3,23	  10,34	   1,23	   16,75	49,71

Montastrea cavernosa	                      1,14	           2,71	   9,74	   1,43	   15,78	65,48

Siderastrea siderea	                      1,59	           0,52	   5,06	   1,13	    8,20	73,68

Diploria labyrinthiformis	                      0,83	           0,00	   3,65	   0,77	    5,92	79,59

Groups Petaquire  &  Garrapata

Average dissimilarity = 56,80

	Group Petaquire	Group Garrapata	       	       	        	     

Species	       Av.Abund	       Av.Abund	Av.Diss	Diss/SD	Contrib%	Cum.%

Colpophyllia natans	           0,23	           3,62	  20,36	   3,18	   35,84	35,84

Montastrea cavernosa	           0,23	           2,71	  14,82	   4,01	   26,08	61,92

Pseudodiploria strigosa	           2,75	           3,23	   4,47	   1,67	    7,87	69,79

Porites astreoides	           0,00	           0,62	   3,71	   1,44	    6,54	76,32

Groups Playa Tiburon  &  Garrapata

Average dissimilarity = 59,74

	Group Playa Tiburon	Group Garrapata	       	       	        	     

Species	           Av.Abund	       Av.Abund	Av.Diss	Diss/SD	Contrib%	Cum.%

Colpophyllia natans	               0,00	           3,62	  18,40	   4,20	   30,80	30,80

Montastrea cavernosa	               0,93	           2,71	   9,07	   2,23	   15,18	45,97

Diploria labyrinthiformis	               1,38	           0,00	   6,87	   2,86	   11,50	57,48

Siderastrea siderea	               1,64	           0,52	   5,66	   1,53	    9,48	66,96

Pseudodiploria strigosa	               2,38	           3,23	   4,66	   1,45	    7,80	74,76

Porites astreoides	               0,40	           0,62	   3,59	   1,62	    6,01	80,77

Groups Punta Mono  &  Garrapata

Average dissimilarity = 62,02

	Group Punta Mono	Group Garrapata	       	       	        	     

Species	        Av.Abund	       Av.Abund	Av.Diss	Diss/SD	Contrib%	Cum.%

Colpophyllia natans	            0,00	           3,62	  18,86	   4,02	   30,40	30,40

Montastrea cavernosa	            0,27	           2,71	  12,80	   3,40	   20,64	51,04

Meandrina meandrites	            1,21	           0,00	   6,30	   1,62	   10,16	61,20

Diploria labyrinthiformis	            1,02	           0,00	   5,21	   3,13	    8,40	69,60

Siderastrea siderea	            1,34	           0,52	   4,20	   1,61	    6,78	76,38

Groups Charagato  &  Garrapata

Average dissimilarity = 70,90

	Group Charagato	Group Garrapata	       	       	        	     

Species	       Av.Abund	       Av.Abund	Av.Diss	Diss/SD	Contrib%	Cum.%

Pseudodiploria strigosa	           0,40	           3,23	  21,18	   2,69	   29,87	29,87

Montastrea cavernosa	           0,00	           2,71	  19,71	   5,05	   27,80	57,68

Colpophyllia natans	           1,86	           3,62	  15,33	   1,26	   21,62	79,29

Groups Punta Conejo  &  Garrapata

Average dissimilarity = 72,16

	Group Punta Conejo	Group Garrapata	       	       	        	     

Species	          Av.Abund	       Av.Abund	Av.Diss	Diss/SD	Contrib%	Cum.%

Colpophyllia natans	              0,00	           3,62	  26,20	   3,86	   36,31	36,31

Montastrea cavernosa	              0,00	           2,71	  19,59	   5,63	   27,15	63,46

Pseudodiploria strigosa	              1,99	           3,23	  12,18	   1,12	   16,87	80,34

Groups La Muerta  &  Garrapata

Average dissimilarity = 65,83

	Group La Muerta	Group Garrapata	       	       	        	     

Species	       Av.Abund	       Av.Abund	Av.Diss	Diss/SD	Contrib%	Cum.%

Colpophyllia natans	           0,62	           3,62	  21,54	   1,98	   32,72	32,72

Montastrea cavernosa	           0,00	           2,71	  18,83	   4,49	   28,61	61,33

Pseudodiploria strigosa	           1,99	           3,23	  10,66	   0,96	   16,19	77,52

Groups Cominoto  &  Garrapata

Average dissimilarity = 58,46

	Group Cominoto	Group Garrapata	       	       	        	     

Species	      Av.Abund	       Av.Abund	Av.Diss	Diss/SD	Contrib%	Cum.%

Colpophyllia natans	          0,00	           3,62	  24,29	   4,52	   41,56	41,56

Montastrea cavernosa	          0,00	           2,71	  18,17	   8,52	   31,08	72,63

Porites astreoides	          0,58	           0,62	   4,19	   1,35	    7,17	79,81

Groups La Pecha  &  Garrapata

Average dissimilarity = 97,13

	Group La Pecha	Group Garrapata	       	       	        	     

Species	      Av.Abund	       Av.Abund	Av.Diss	Diss/SD	Contrib%	Cum.%

Colpophyllia natans	          0,00	           3,62	  31,38	   4,50	   32,30	32,30

Pseudodiploria strigosa	          0,17	           3,23	  26,46	   6,00	   27,25	59,55

Montastrea cavernosa	          0,00	           2,71	  23,47	   8,21	   24,16	83,71

Groups Puerto Real  &  Garrapata

Average dissimilarity = 61,22

	Group Puerto Real	Group Garrapata	       	       	        	     

Species	         Av.Abund	       Av.Abund	Av.Diss	Diss/SD	Contrib%	Cum.%

Colpophyllia natans	             0,00	           3,62	  22,88	   4,42	   37,37	37,37

Montastrea cavernosa	             0,00	           2,71	  17,11	   7,92	   27,95	65,32

Pseudodiploria strigosa	             2,60	           3,23	   6,66	   1,54	   10,88	76,20

Groups Cayo Agua  &  Garrapata

Average dissimilarity = 85,05

	Group Cayo Agua	Group Garrapata	       	       	        	     

Species	       Av.Abund	       Av.Abund	Av.Diss	Diss/SD	Contrib%	Cum.%

Madracis mirabilis	           3,47	           0,00	  13,20	   4,43	   15,53	15,53

Pseudodiploria strigosa	           0,00	           3,23	  12,34	   8,36	   14,51	30,03

Orbicella annularis	           3,05	           0,00	  11,58	   3,61	   13,62	43,65

Orbicella faveolata	           3,24	           0,35	  11,13	   3,77	   13,08	56,74

Montastrea cavernosa	           0,17	           2,71	   9,66	   6,63	   11,36	68,09

Colpophyllia natans	           1,15	           3,62	   9,57	   2,06	   11,25	79,35

Groups Boca de Cote  &  Garrapata

Average dissimilarity = 70,47

	Group Boca de Cote	Group Garrapata	       	       	        	     

Species	          Av.Abund	       Av.Abund	Av.Diss	Diss/SD	Contrib%	Cum.%

Colpophyllia natans	              0,17	           3,62	  12,94	   3,64	   18,37	18,37

Orbicella faveolata	              3,10	           0,35	  10,22	   2,82	   14,50	32,87

Pseudodiploria strigosa	              0,62	           3,23	   9,90	   2,87	   14,05	46,91

Siderastrea siderea	              2,54	           0,52	   7,51	   3,35	   10,66	57,57

Diploria labyrinthiformis	              1,37	           0,00	   5,20	   2,80	    7,38	64,95

Porites astreoides	              1,92	           0,62	   4,87	   2,30	    6,91	71,86

Orbicella annularis	              1,35	           0,00	   4,84	   1,53	    6,87	78,73

Groups Dos Mosquises Sur  &  Garrapata

Average dissimilarity = 70,26

	Group Dos Mosquises Sur	Group Garrapata	       	       	        	     

Species	               Av.Abund	       Av.Abund	Av.Diss	Diss/SD	Contrib%	Cum.%

Orbicella faveolata	                   3,41	           0,35	  12,43	   2,71	   17,70	17,70

Pseudodiploria strigosa	                   0,45	           3,23	  11,18	   2,97	   15,91	33,61

Montastrea cavernosa	                   0,00	           2,71	  10,87	   7,14	   15,48	49,09

Madracis mirabilis	                   2,55	           0,00	   9,75	   1,61	   13,87	62,96

Orbicella annularis	                   2,43	           0,00	   9,31	   1,49	   13,25	76,21

Groups Madrisqui  &  Garrapata

Average dissimilarity = 68,98

	Group Madrisqui	Group Garrapata	       	       	        	     

Species	       Av.Abund	       Av.Abund	Av.Diss	Diss/SD	Contrib%	Cum.%

Pseudodiploria strigosa	           0,00	           3,23	  15,34	   7,45	   22,23	22,23

Orbicella faveolata	           2,67	           0,35	  10,97	   2,88	   15,91	38,14

Montastrea cavernosa	           0,52	           2,71	  10,19	   2,31	   14,77	52,91

Colpophyllia natans	           1,96	           3,62	   8,75	   1,29	   12,68	65,60

Porites astreoides	           1,41	           0,62	   5,12	   1,89	    7,43	73,02

Agaricia sp.	           0,93	           0,00	   4,02	   0,74	    5,83	78,86

Groups Pelona de Rabusqui  &  Garrapata

Average dissimilarity = 78,75

	Group Pelona de Rabusqui	Group Garrapata	       	       	        	     

Species	                Av.Abund	       Av.Abund	Av.Diss	Diss/SD	Contrib%	Cum.%

Orbicella faveolata	                    4,91	           0,35	  20,02	   5,32	   25,42	25,42

Pseudodiploria strigosa	                    0,17	           3,23	  13,43	   4,71	   17,05	42,47

Montastrea cavernosa	                    0,00	           2,71	  11,84	   6,72	   15,04	57,51

Orbicella annularis	                    2,00	           0,00	   8,59	   1,53	   10,91	68,43

Colpophyllia natans	                    1,87	           3,62	   8,14	   1,62	   10,34	78,76

Groups Salinas  &  Garrapata

Average dissimilarity = 61,44

	Group Salinas	Group Garrapata	       	       	        	     

Species	     Av.Abund	       Av.Abund	Av.Diss	Diss/SD	Contrib%	Cum.%

Colpophyllia natans	         0,27	           3,62	  14,80	   3,76	   24,09	24,09

Pseudodiploria strigosa	         1,32	           3,23	   8,83	   1,70	   14,37	38,46

Orbicella faveolata	         2,24	           0,35	   8,71	   1,71	   14,17	52,64

Siderastrea siderea	         2,20	           0,52	   7,49	   1,88	   12,19	64,82

Diploria labyrinthiformis	         1,40	           0,00	   6,00	   1,54	    9,76	74,59

Montastrea cavernosa	         1,66	           2,71	   5,23	   0,97	    8,51	83,09

Groups La Venada  &  Garrapata

Average dissimilarity = 97,06

	Group La Venada	Group Garrapata	       	       	        	     

Species	       Av.Abund	       Av.Abund	Av.Diss	Diss/SD	Contrib%	Cum.%

Acropora cervicornis	           4,54	           0,00	  26,84	  15,01	   27,65	27,65

Colpophyllia natans	           0,00	           3,62	  21,46	   4,39	   22,11	49,77

Pseudodiploria strigosa	           0,27	           3,23	  17,63	   4,11	   18,16	67,93

Montastrea cavernosa	           0,00	           2,71	  16,05	   7,78	   16,53	84,46

Groups San Agustin  &  Garrapata

Average dissimilarity = 97,13

	Group San Agustin	Group Garrapata	       	       	        	     

Species	         Av.Abund	       Av.Abund	Av.Diss	Diss/SD	Contrib%	Cum.%

Colpophyllia natans	             0,00	           3,62	  31,38	   4,50	   32,30	32,30

Pseudodiploria strigosa	             0,00	           3,23	  27,90	   9,25	   28,72	61,03

Montastrea cavernosa	             0,17	           2,71	  22,03	   5,23	   22,69	83,71

Groups Blanca  &  Garrapata

Average dissimilarity = 24,57

	Group Blanca	Group Garrapata	       	       	        	     

Species	    Av.Abund	       Av.Abund	Av.Diss	Diss/SD	Contrib%	Cum.%

Dichocoenia stokesii	        1,17	           0,17	   4,41	   1,63	   17,94	17,94

Montastrea cavernosa	        1,70	           2,71	   4,24	   2,33	   17,26	35,19

Colpophyllia natans	        3,84	           3,62	   2,39	   0,92	    9,71	44,90

Siderastrea siderea	        0,52	           0,52	   2,15	   1,34	    8,74	53,64

Pseudodiploria strigosa	        2,94	           3,23	   1,90	   1,22	    7,73	61,37

Meandrina sp.	        0,49	           0,00	   1,74	   0,56	    7,07	68,44

Porites astreoides	        0,62	           0,62	   1,72	   1,07	    6,99	75,43

Groups Carabela  &  Garrapata

Average dissimilarity = 88,02

	Group Carabela	Group Garrapata	       	       	        	     

Species	      Av.Abund	       Av.Abund	Av.Diss	Diss/SD	Contrib%	Cum.%

Pseudodiploria strigosa	          0,00	           3,23	  26,57	   7,40	   30,18	30,18

Colpophyllia natans	          0,66	           3,62	  25,48	   2,12	   28,94	59,13

Montastrea cavernosa	          0,17	           2,71	  20,91	   5,11	   23,76	82,88

Groups Punta Cruz  &  Garrapata

Average dissimilarity = 55,32

	Group Punta Cruz	Group Garrapata	       	       	        	     

Species	        Av.Abund	       Av.Abund	Av.Diss	Diss/SD	Contrib%	Cum.%

Colpophyllia natans	            0,85	           3,62	  16,57	   2,57	   29,96	29,96

Pseudodiploria strigosa	            1,07	           3,23	  13,46	   1,60	   24,34	54,30

Dichocoenia stokesii	            1,29	           0,17	   7,01	   1,71	   12,68	66,97

Montastrea cavernosa	            1,66	           2,71	   6,45	   1,39	   11,66	78,63

Groups Gabarra  &  Garrapata

Average dissimilarity = 60,85

	Group Gabarra	Group Garrapata	       	       	        	     

Species	     Av.Abund	       Av.Abund	Av.Diss	Diss/SD	Contrib%	Cum.%

Montastrea cavernosa	         0,00	           2,71	  18,10	   5,38	   29,75	29,75

Colpophyllia natans	         1,17	           3,62	  17,04	   1,69	   28,00	57,74

Pseudodiploria strigosa	         1,57	           3,23	  11,46	   1,70	   18,84	76,58

Groups La Pared  &  Boca Seca

Average dissimilarity = 88,32

	Group La Pared	Group Boca Seca	       	       	        	     

Species	      Av.Abund	       Av.Abund	Av.Diss	Diss/SD	Contrib%	Cum.%

Pseudodiploria strigosa	          2,33	           0,45	  14,29	   1,76	   16,18	16,18

Meandrina meandrites	          1,94	           0,00	  13,20	   1,52	   14,95	31,12

Siderastrea siderea	          1,37	           0,00	  10,09	   3,92	   11,42	42,54

Montastrea cavernosa	          1,38	           0,00	   9,35	   1,52	   10,59	53,13

Orbicella faveolata	          0,00	           0,90	   6,18	   0,89	    7,00	60,13

Diploria labyrinthiformis	          0,72	           0,00	   5,44	   0,95	    6,16	66,30

Other (Scleractinian massive)	          0,62	           0,00	   4,92	   0,89	    5,57	71,87

Agaricia humilis	          0,35	           0,52	   4,48	   1,82	    5,08	76,94

Groups Media Legua  &  Boca Seca

Average dissimilarity = 85,50

	Group Media Legua	Group Boca Seca	       	       	        	     

Species	         Av.Abund	       Av.Abund	Av.Diss	Diss/SD	Contrib%	Cum.%

Pseudodiploria strigosa	             2,20	           0,45	  21,46	   1,89	   25,09	25,09

Siderastrea siderea	             0,85	           0,00	   9,87	   1,39	   11,55	36,64

Orbicella faveolata	             0,17	           0,90	   9,65	   1,03	   11,29	47,93

Porites astreoides	             0,17	           0,75	   7,23	   1,19	    8,45	56,38

Meandrina meandrites	             0,60	           0,00	   7,09	   0,55	    8,29	64,67

Agaricia humilis	             0,00	           0,52	   6,58	   1,55	    7,69	72,36

Colpophyllia natans	             0,17	           0,49	   5,94	   0,76	    6,95	79,31

Groups Punta de Media Legua  &  Boca Seca

Average dissimilarity = 91,76

	Group Punta de Media Legua	Group Boca Seca	       	       	        	     

Species	                  Av.Abund	       Av.Abund	Av.Diss	Diss/SD	Contrib%	Cum.%

Siderastrea siderea	                      1,59	           0,00	  14,71	   3,41	   16,04	16,04

Pseudodiploria strigosa	                      1,63	           0,45	  14,25	   1,35	   15,53	31,57

Montastrea cavernosa	                      1,14	           0,00	  11,09	   1,26	   12,08	43,65

Orbicella faveolata	                      0,00	           0,90	   9,83	   0,83	   10,71	54,35

Porites astreoides	                      0,27	           0,75	   7,88	   1,12	    8,58	62,94

Agaricia humilis	                      0,00	           0,52	   6,85	   1,18	    7,47	70,41

Diploria labyrinthiformis	                      0,83	           0,00	   5,63	   0,78	    6,13	76,54

Groups Petaquire  &  Boca Seca

Average dissimilarity = 87,73

	Group Petaquire	Group Boca Seca	       	       	        	     

Species	       Av.Abund	       Av.Abund	Av.Diss	Diss/SD	Contrib%	Cum.%

Pseudodiploria strigosa	           2,75	           0,45	  27,24	   2,19	   31,05	31,05

Siderastrea siderea	           0,92	           0,00	  10,32	   3,20	   11,76	42,81

Orbicella faveolata	           0,23	           0,90	   9,69	   0,96	   11,05	53,86

Porites astreoides	           0,00	           0,75	   7,95	   1,24	    9,06	62,92

Agaricia humilis	           0,00	           0,52	   6,65	   1,38	    7,58	70,50

Colpophyllia natans	           0,23	           0,49	   5,94	   0,74	    6,77	77,28

Groups Playa Tiburon  &  Boca Seca

Average dissimilarity = 85,17

	Group Playa Tiburon	Group Boca Seca	       	       	        	     

Species	           Av.Abund	       Av.Abund	Av.Diss	Diss/SD	Contrib%	Cum.%

Pseudodiploria strigosa	               2,38	           0,45	  17,01	   2,10	   19,97	19,97

Siderastrea siderea	               1,64	           0,00	  14,03	   2,37	   16,47	36,45

Diploria labyrinthiformis	               1,38	           0,00	  11,50	   2,78	   13,50	49,95

Montastrea cavernosa	               0,93	           0,00	   7,93	   1,30	    9,32	59,26

Orbicella faveolata	               0,00	           0,90	   7,23	   0,90	    8,48	67,75

Porites astreoides	               0,40	           0,75	   6,38	   1,31	    7,49	75,24

Groups Punta Mono  &  Boca Seca

Average dissimilarity = 91,25

	Group Punta Mono	Group Boca Seca	       	       	        	     

Species	        Av.Abund	       Av.Abund	Av.Diss	Diss/SD	Contrib%	Cum.%

Pseudodiploria strigosa	            2,96	           0,45	  22,31	   2,81	   24,45	24,45

Siderastrea siderea	            1,34	           0,00	  11,89	   3,19	   13,03	37,48

Meandrina meandrites	            1,21	           0,00	  11,01	   1,59	   12,07	49,55

Diploria labyrinthiformis	            1,02	           0,00	   8,93	   3,06	    9,79	59,34

Orbicella faveolata	            0,00	           0,90	   7,57	   0,89	    8,30	67,64

Porites astreoides	            0,17	           0,75	   5,70	   1,14	    6,24	73,88

Agaricia humilis	            0,00	           0,52	   5,01	   1,48	    5,49	79,38

Groups Charagato  &  Boca Seca

Average dissimilarity = 90,16

	Group Charagato	Group Boca Seca	       	       	        	     

Species	       Av.Abund	       Av.Abund	Av.Diss	Diss/SD	Contrib%	Cum.%

Colpophyllia natans	           1,86	           0,49	  25,93	   1,33	   28,76	28,76

Orbicella faveolata	           0,00	           0,90	  16,05	   0,83	   17,80	46,57

Porites astreoides	           0,00	           0,75	  12,56	   1,15	   13,93	60,49

Agaricia humilis	           0,00	           0,52	  12,17	   1,08	   13,50	73,99

Pseudodiploria strigosa	           0,40	           0,45	   8,23	   1,06	    9,13	83,12

Groups Punta Conejo  &  Boca Seca

Average dissimilarity = 89,07

	Group Punta Conejo	Group Boca Seca	       	       	        	     

Species	          Av.Abund	       Av.Abund	Av.Diss	Diss/SD	Contrib%	Cum.%

Pseudodiploria strigosa	              1,99	           0,45	  26,72	   1,34	   30,00	30,00

Orbicella faveolata	              0,00	           0,90	  15,17	   0,88	   17,03	47,03

Agaricia humilis	              0,00	           0,52	  11,31	   1,22	   12,69	59,73

Porites astreoides	              0,17	           0,75	  11,22	   1,11	   12,60	72,32

Colpophyllia natans	              0,00	           0,49	   6,77	   0,54	    7,60	79,92

Groups La Muerta  &  Boca Seca

Average dissimilarity = 84,19

	Group La Muerta	Group Boca Seca	       	       	        	     

Species	       Av.Abund	       Av.Abund	Av.Diss	Diss/SD	Contrib%	Cum.%

Pseudodiploria strigosa	           1,99	           0,45	  24,51	   1,53	   29,11	29,11

Orbicella faveolata	           0,35	           0,90	  15,28	   0,83	   18,15	47,26

Porites astreoides	           0,52	           0,75	  13,37	   1,25	   15,88	63,14

Colpophyllia natans	           0,62	           0,49	  11,46	   0,74	   13,62	76,76

Groups Cominoto  &  Boca Seca

Average dissimilarity = 81,76

	Group Cominoto	Group Boca Seca	       	       	        	     

Species	      Av.Abund	       Av.Abund	Av.Diss	Diss/SD	Contrib%	Cum.%

Pseudodiploria strigosa	          2,96	           0,45	  39,01	   2,13	   47,71	47,71

Orbicella faveolata	          0,00	           0,90	  11,94	   0,95	   14,60	62,31

Porites astreoides	          0,58	           0,75	   9,79	   1,16	   11,98	74,29

Agaricia humilis	          0,00	           0,52	   8,41	   1,55	   10,28	84,57

Groups La Pecha  &  Boca Seca

Average dissimilarity = 97,10

	Group La Pecha	Group Boca Seca	       	       	        	     

Species	      Av.Abund	       Av.Abund	Av.Diss	Diss/SD	Contrib%	Cum.%

Orbicella faveolata	          0,00	           0,90	  24,93	   0,94	   25,68	25,68

Agaricia humilis	          0,00	           0,52	  20,21	   1,34	   20,81	46,49

Porites astreoides	          0,00	           0,75	  18,68	   1,33	   19,24	65,73

Pseudodiploria strigosa	          0,17	           0,45	   9,97	   0,97	   10,27	76,00

Groups Puerto Real  &  Boca Seca

Average dissimilarity = 75,45

	Group Puerto Real	Group Boca Seca	       	       	        	     

Species	         Av.Abund	       Av.Abund	Av.Diss	Diss/SD	Contrib%	Cum.%

Pseudodiploria strigosa	             2,60	           0,45	  28,05	   1,87	   37,18	37,18

Orbicella faveolata	             0,00	           0,90	  10,60	   0,94	   14,05	51,22

Porites astreoides	             1,10	           0,75	   8,38	   1,44	   11,10	62,33

Agaricia humilis	             0,00	           0,52	   7,33	   1,55	    9,72	72,04

Colpophyllia natans	             0,00	           0,49	   5,19	   0,56	    6,88	78,93

Groups Cayo Agua  &  Boca Seca

Average dissimilarity = 82,89

	Group Cayo Agua	Group Boca Seca	       	       	        	     

Species	       Av.Abund	       Av.Abund	Av.Diss	Diss/SD	Contrib%	Cum.%

Madracis mirabilis	           3,47	           0,00	  18,93	   4,15	   22,84	22,84

Orbicella annularis	           3,05	           0,00	  16,58	   3,47	   20,01	42,84

Orbicella faveolata	           3,24	           0,90	  13,22	   1,98	   15,95	58,80

Colpophyllia natans	           1,15	           0,49	   6,09	   1,29	    7,35	66,15

Madracis decactis	           1,10	           0,00	   5,74	   0,96	    6,93	73,07

Porites astreoides	           0,69	           0,75	   4,49	   1,15	    5,41	78,49

Groups Boca de Cote  &  Boca Seca

Average dissimilarity = 76,84

	Group Boca de Cote	Group Boca Seca	       	       	        	     

Species	          Av.Abund	       Av.Abund	Av.Diss	Diss/SD	Contrib%	Cum.%

Siderastrea siderea	              2,54	           0,00	  13,42	   4,36	   17,47	17,47

Orbicella faveolata	              3,10	           0,90	  12,08	   1,82	   15,72	33,19

Montastrea cavernosa	              1,76	           0,00	   9,18	   2,31	   11,94	45,14

Diploria labyrinthiformis	              1,37	           0,00	   7,46	   2,55	    9,71	54,85

Orbicella annularis	              1,35	           0,00	   6,78	   1,52	    8,83	63,67

Porites astreoides	              1,92	           0,75	   6,59	   1,65	    8,58	72,25

Madracis mirabilis	              1,01	           0,00	   4,83	   0,74	    6,28	78,53

Groups Dos Mosquises Sur  &  Boca Seca

Average dissimilarity = 82,18

	Group Dos Mosquises Sur	Group Boca Seca	       	       	        	     

Species	               Av.Abund	       Av.Abund	Av.Diss	Diss/SD	Contrib%	Cum.%

Orbicella faveolata	                   3,41	           0,90	  15,47	   1,68	   18,83	18,83

Madracis mirabilis	                   2,55	           0,00	  14,01	   1,59	   17,04	35,87

Orbicella annularis	                   2,43	           0,00	  13,39	   1,46	   16,30	52,16

Colpophyllia natans	                   2,54	           0,49	  12,62	   1,82	   15,35	67,52

Porites astreoides	                   0,17	           0,75	   3,82	   1,15	    4,64	72,16

Pseudodiploria strigosa	                   0,45	           0,45	   3,79	   1,00	    4,62	76,78

Groups Madrisqui  &  Boca Seca

Average dissimilarity = 73,93

	Group Madrisqui	Group Boca Seca	       	       	        	     

Species	       Av.Abund	       Av.Abund	Av.Diss	Diss/SD	Contrib%	Cum.%

Orbicella faveolata	           2,67	           0,90	  14,40	   1,70	   19,48	19,48

Colpophyllia natans	           1,96	           0,49	  12,82	   1,44	   17,34	36,82

Porites astreoides	           1,41	           0,75	   8,14	   1,52	   11,01	47,83

Agaricia sp.	           0,93	           0,17	   6,23	   0,80	    8,42	56,26

Eusmilia fastigata	           0,68	           0,00	   4,79	   0,95	    6,47	62,73

Montastrea cavernosa	           0,52	           0,00	   4,62	   0,55	    6,24	68,98

Agaricia humilis	           0,00	           0,52	   4,21	   1,59	    5,70	74,67

Pseudodiploria strigosa	           0,00	           0,45	   2,99	   0,92	    4,05	78,72

Groups Pelona de Rabusqui  &  Boca Seca

Average dissimilarity = 79,39

	Group Pelona de Rabusqui	Group Boca Seca	       	       	        	     

Species	                Av.Abund	       Av.Abund	Av.Diss	Diss/SD	Contrib%	Cum.%

Orbicella faveolata	                    4,91	           0,90	  27,63	   2,71	   34,81	34,81

Orbicella annularis	                    2,00	           0,00	  13,17	   1,50	   16,59	51,39

Colpophyllia natans	                    1,87	           0,49	  10,62	   1,71	   13,38	64,77

Agaricia sp.	                    1,10	           0,17	   6,46	   1,13	    8,14	72,92

Porites astreoides	                    0,00	           0,75	   4,77	   1,28	    6,01	78,92

Groups Salinas  &  Boca Seca

Average dissimilarity = 78,59

	Group Salinas	Group Boca Seca	       	       	        	     

Species	     Av.Abund	       Av.Abund	Av.Diss	Diss/SD	Contrib%	Cum.%

Siderastrea siderea	         2,20	           0,00	  15,38	   2,37	   19,57	19,57

Orbicella faveolata	         2,24	           0,90	  11,64	   1,28	   14,81	34,38

Montastrea cavernosa	         1,66	           0,00	  10,57	   1,63	   13,45	47,82

Diploria labyrinthiformis	         1,40	           0,00	   9,17	   1,46	   11,67	59,49

Pseudodiploria strigosa	         1,32	           0,45	   7,40	   1,44	    9,42	68,91

Porites astreoides	         0,68	           0,75	   5,05	   1,39	    6,43	75,33

Groups La Venada  &  Boca Seca

Average dissimilarity = 94,87

	Group La Venada	Group Boca Seca	       	       	        	     

Species	       Av.Abund	       Av.Abund	Av.Diss	Diss/SD	Contrib%	Cum.%

Acropora cervicornis	           4,54	           0,00	  51,82	   4,39	   54,63	54,63

Orbicella faveolata	           0,00	           0,90	   9,39	   0,93	    9,89	64,52

Porites astreoides	           0,00	           0,75	   7,73	   1,32	    8,15	72,67

Agaricia humilis	           0,40	           0,52	   7,56	   1,87	    7,97	80,64

Groups San Agustin  &  Boca Seca

Average dissimilarity = 100,00

	Group San Agustin	Group Boca Seca	       	       	        	     

Species	         Av.Abund	       Av.Abund	Av.Diss	Diss/SD	Contrib%	Cum.%

Orbicella faveolata	             0,00	           0,90	  24,93	   0,94	   24,93	24,93

Agaricia humilis	             0,00	           0,52	  20,21	   1,34	   20,21	45,14

Porites astreoides	             0,00	           0,75	  18,68	   1,33	   18,68	63,82

Colpophyllia natans	             0,00	           0,49	   9,54	   0,56	    9,54	73,36

Pseudodiploria strigosa	             0,00	           0,45	   8,12	   0,94	    8,12	81,48

Groups Blanca  &  Boca Seca

Average dissimilarity = 84,08

	Group Blanca	Group Boca Seca	       	       	        	     

Species	    Av.Abund	       Av.Abund	Av.Diss	Diss/SD	Contrib%	Cum.%

Colpophyllia natans	        3,84	           0,49	  21,97	   2,79	   26,13	26,13

Pseudodiploria strigosa	        2,94	           0,45	  16,44	   2,69	   19,55	45,68

Montastrea cavernosa	        1,70	           0,00	  11,06	   3,25	   13,16	58,84

Dichocoenia stokesii	        1,17	           0,00	   7,04	   1,57	    8,38	67,22

Orbicella faveolata	        0,00	           0,90	   5,48	   0,89	    6,52	73,74

Agaricia humilis	        0,00	           0,52	   3,50	   1,58	    4,16	77,90

Groups Carabela  &  Boca Seca

Average dissimilarity = 96,79

	Group Carabela	Group Boca Seca	       	       	        	     

Species	      Av.Abund	       Av.Abund	Av.Diss	Diss/SD	Contrib%	Cum.%

Orbicella faveolata	          0,00	           0,90	  21,78	   0,90	   22,50	22,50

Agaricia humilis	          0,00	           0,52	  17,30	   1,24	   17,87	40,37

Colpophyllia natans	          0,66	           0,49	  16,86	   0,75	   17,42	57,79

Porites astreoides	          0,00	           0,75	  16,56	   1,27	   17,11	74,90

Pseudodiploria strigosa	          0,00	           0,45	   7,43	   0,92	    7,67	82,57

Groups Punta Cruz  &  Boca Seca

Average dissimilarity = 90,44

	Group Punta Cruz	Group Boca Seca	       	       	        	     

Species	        Av.Abund	       Av.Abund	Av.Diss	Diss/SD	Contrib%	Cum.%

Montastrea cavernosa	            1,66	           0,00	  19,16	   1,84	   21,19	21,19

Dichocoenia stokesii	            1,29	           0,00	  14,29	   1,49	   15,80	36,98

Pseudodiploria strigosa	            1,07	           0,45	  10,73	   1,09	   11,87	48,85

Colpophyllia natans	            0,85	           0,49	  10,36	   1,23	   11,46	60,31

Orbicella faveolata	            0,17	           0,90	   9,55	   1,01	   10,55	70,87

Porites astreoides	            0,00	           0,75	   7,85	   1,31	    8,68	79,55

Groups Gabarra  &  Boca Seca

Average dissimilarity = 82,20

	Group Gabarra	Group Boca Seca	       	       	        	     

Species	     Av.Abund	       Av.Abund	Av.Diss	Diss/SD	Contrib%	Cum.%

Pseudodiploria strigosa	         1,57	           0,45	  17,80	   1,42	   21,65	21,65

Colpophyllia natans	         1,17	           0,49	  14,54	   1,15	   17,69	39,35

Orbicella faveolata	         0,00	           0,90	  12,72	   0,85	   15,47	54,82

Porites astreoides	         0,17	           0,75	   9,43	   1,06	   11,47	66,29

Agaricia humilis	         0,00	           0,52	   9,22	   1,15	   11,22	77,51

Groups Garrapata  &  Boca Seca

Average dissimilarity = 81,27

	Group Garrapata	Group Boca Seca	       	       	        	     

Species	       Av.Abund	       Av.Abund	Av.Diss	Diss/SD	Contrib%	Cum.%

Colpophyllia natans	           3,62	           0,49	  21,49	   2,40	   26,44	26,44

Pseudodiploria strigosa	           3,23	           0,45	  19,09	   3,24	   23,49	49,93

Montastrea cavernosa	           2,71	           0,00	  18,30	   6,07	   22,51	72,44

Orbicella faveolata	           0,35	           0,90	   6,01	   1,05	    7,39	79,83

Groups La Pared  &  Bajo Caiman

Average dissimilarity = 97,14

	Group La Pared	Group Bajo Caiman	       	       	        	     

Species	      Av.Abund	         Av.Abund	Av.Diss	Diss/SD	Contrib%	Cum.%

Pseudodiploria strigosa	          2,33	             0,00	  17,61	   2,60	   18,13	18,13

Meandrina meandrites	          1,94	             0,00	  13,53	   1,52	   13,93	32,06

Siderastrea siderea	          1,37	             0,00	  10,36	   4,01	   10,67	42,72

Montastrea cavernosa	          1,38	             0,00	   9,59	   1,53	    9,87	52,59

Orbicella faveolata	          0,00	             1,12	   8,36	   3,31	    8,60	61,20

Porites astreoides	          0,45	             1,01	   7,25	   1,09	    7,46	68,66

Diploria labyrinthiformis	          0,72	             0,00	   5,59	   0,95	    5,76	74,42

Other (Scleractinian massive)	          0,62	             0,00	   5,07	   0,89	    5,21	79,63

Groups Media Legua  &  Bajo Caiman

Average dissimilarity = 93,26

	Group Media Legua	Group Bajo Caiman	       	       	        	     

Species	         Av.Abund	         Av.Abund	Av.Diss	Diss/SD	Contrib%	Cum.%

Pseudodiploria strigosa	             2,20	             0,00	  26,73	   3,31	   28,66	28,66

Orbicella faveolata	             0,17	             1,12	  11,48	   2,03	   12,31	40,97

Porites astreoides	             0,17	             1,01	  11,04	   0,94	   11,84	52,81

Siderastrea siderea	             0,85	             0,00	  10,28	   1,40	   11,02	63,83

Meandrina meandrites	             0,60	             0,00	   7,37	   0,55	    7,91	71,73

Colpophyllia natans	             0,17	             0,45	   6,41	   0,73	    6,87	78,60

Groups Punta de Media Legua  &  Bajo Caiman

Average dissimilarity = 97,75

	Group Punta de Media Legua	Group Bajo Caiman	       	       	        	     

Species	                  Av.Abund	         Av.Abund	Av.Diss	Diss/SD	Contrib%	Cum.%

Pseudodiploria strigosa	                      1,63	             0,00	  16,59	   1,40	   16,97	16,97

Siderastrea siderea	                      1,59	             0,00	  15,30	   3,51	   15,65	32,62

Orbicella faveolata	                      0,00	             1,12	  14,06	   1,73	   14,38	47,00

Montastrea cavernosa	                      1,14	             0,00	  11,51	   1,25	   11,77	58,78

Porites astreoides	                      0,27	             1,01	  11,02	   0,79	   11,27	70,05

Colpophyllia natans	                      0,17	             0,45	   6,03	   0,58	    6,17	76,21

Groups Petaquire  &  Bajo Caiman

Average dissimilarity = 94,64

	Group Petaquire	Group Bajo Caiman	       	       	        	     

Species	       Av.Abund	         Av.Abund	Av.Diss	Diss/SD	Contrib%	Cum.%

Pseudodiploria strigosa	           2,75	             0,00	  32,78	   3,59	   34,64	34,64

Orbicella faveolata	           0,23	             1,12	  11,53	   1,51	   12,18	46,82

Porites astreoides	           0,00	             1,01	  10,84	   0,81	   11,45	58,27

Siderastrea siderea	           0,92	             0,00	  10,75	   3,24	   11,35	69,62

Colpophyllia natans	           0,23	             0,45	   6,46	   0,70	    6,83	76,45

Groups Playa Tiburon  &  Bajo Caiman

Average dissimilarity = 97,35

	Group Playa Tiburon	Group Bajo Caiman	       	       	        	     

Species	           Av.Abund	         Av.Abund	Av.Diss	Diss/SD	Contrib%	Cum.%

Pseudodiploria strigosa	               2,38	             0,00	  20,93	   3,69	   21,50	21,50

Siderastrea siderea	               1,64	             0,00	  14,47	   2,41	   14,86	36,36

Diploria labyrinthiformis	               1,38	             0,00	  11,84	   2,84	   12,16	48,52

Orbicella faveolata	               0,00	             1,12	   9,89	   3,18	   10,16	58,68

Porites astreoides	               0,40	             1,01	   8,63	   0,96	    8,87	67,55

Montastrea cavernosa	               0,93	             0,00	   8,18	   1,31	    8,40	75,95

Groups Punta Mono  &  Bajo Caiman

Average dissimilarity = 98,75

	Group Punta Mono	Group Bajo Caiman	       	       	        	     

Species	        Av.Abund	         Av.Abund	Av.Diss	Diss/SD	Contrib%	Cum.%

Pseudodiploria strigosa	            2,96	             0,00	  26,55	   6,61	   26,89	26,89

Siderastrea siderea	            1,34	             0,00	  12,27	   3,26	   12,43	39,32

Meandrina meandrites	            1,21	             0,00	  11,38	   1,60	   11,52	50,84

Orbicella faveolata	            0,00	             1,12	  10,42	   2,84	   10,55	61,39

Diploria labyrinthiformis	            1,02	             0,00	   9,22	   3,14	    9,34	70,73

Porites astreoides	            0,17	             1,01	   8,68	   0,91	    8,79	79,52

Groups Charagato  &  Bajo Caiman

Average dissimilarity = 90,34

	Group Charagato	Group Bajo Caiman	       	       	        	     

Species	       Av.Abund	         Av.Abund	Av.Diss	Diss/SD	Contrib%	Cum.%

Colpophyllia natans	           1,86	             0,45	  27,58	   1,32	   30,53	30,53

Orbicella faveolata	           0,00	             1,12	  25,39	   1,18	   28,11	58,64

Porites astreoides	           0,00	             1,01	  16,62	   0,77	   18,40	77,04

Groups Punta Conejo  &  Bajo Caiman

Average dissimilarity = 98,13

	Group Punta Conejo	Group Bajo Caiman	       	       	        	     

Species	          Av.Abund	         Av.Abund	Av.Diss	Diss/SD	Contrib%	Cum.%

Pseudodiploria strigosa	              1,99	             0,00	  30,51	   1,41	   31,09	31,09

Orbicella faveolata	              0,00	             1,12	  23,10	   1,64	   23,54	54,63

Porites astreoides	              0,17	             1,01	  16,40	   0,85	   16,72	71,35

Colpophyllia natans	              0,00	             0,45	   8,46	   0,53	    8,62	79,96

Groups La Muerta  &  Bajo Caiman

Average dissimilarity = 87,91

	Group La Muerta	Group Bajo Caiman	       	       	        	     

Species	       Av.Abund	         Av.Abund	Av.Diss	Diss/SD	Contrib%	Cum.%

Pseudodiploria strigosa	           1,99	             0,00	  27,20	   1,46	   30,94	30,94

Orbicella faveolata	           0,35	             1,12	  21,58	   0,92	   24,55	55,49

Porites astreoides	           0,52	             1,01	  16,73	   0,83	   19,03	74,52

Colpophyllia natans	           0,62	             0,45	  13,17	   0,72	   14,99	89,51

Groups Cominoto  &  Bajo Caiman

Average dissimilarity = 93,13

	Group Cominoto	Group Bajo Caiman	       	       	        	     

Species	      Av.Abund	         Av.Abund	Av.Diss	Diss/SD	Contrib%	Cum.%

Pseudodiploria strigosa	          2,96	             0,00	  46,28	   3,19	   49,70	49,70

Orbicella faveolata	          0,00	             1,12	  17,17	   3,24	   18,44	68,14

Porites astreoides	          0,58	             1,01	  15,01	   1,14	   16,12	84,25

Groups La Pecha  &  Bajo Caiman

Average dissimilarity = 100,00

	Group La Pecha	Group Bajo Caiman	       	       	        	     

Species	      Av.Abund	         Av.Abund	Av.Diss	Diss/SD	Contrib%	Cum.%

Orbicella faveolata	          0,00	             1,12	  42,99	   1,44	   42,99	42,99

Porites astreoides	          0,00	             1,01	  23,97	   0,83	   23,97	66,96

Colpophyllia natans	          0,00	             0,45	  13,46	   0,56	   13,46	80,43

Groups Puerto Real  &  Bajo Caiman

Average dissimilarity = 86,26

	Group Puerto Real	Group Bajo Caiman	       	       	        	     

Species	         Av.Abund	         Av.Abund	Av.Diss	Diss/SD	Contrib%	Cum.%

Pseudodiploria strigosa	             2,60	             0,00	  34,04	   2,96	   39,46	39,46

Orbicella faveolata	             0,00	             1,12	  15,04	   3,22	   17,43	56,89

Porites astreoides	             1,10	             1,01	  14,14	   1,97	   16,40	73,29

Colpophyllia natans	             0,00	             0,45	   5,91	   0,55	    6,85	80,14

Groups Cayo Agua  &  Bajo Caiman

Average dissimilarity = 76,63

	Group Cayo Agua	Group Bajo Caiman	       	       	        	     

Species	       Av.Abund	         Av.Abund	Av.Diss	Diss/SD	Contrib%	Cum.%

Madracis mirabilis	           3,47	             0,00	  19,31	   4,25	   25,21	25,21

Orbicella annularis	           3,05	             0,45	  14,55	   2,47	   18,98	44,19

Orbicella faveolata	           3,24	             1,12	  12,05	   3,37	   15,73	59,91

Colpophyllia natans	           1,15	             0,45	   6,11	   1,30	    7,97	67,88

Porites astreoides	           0,69	             1,01	   6,10	   1,14	    7,96	75,84

Groups Boca de Cote  &  Bajo Caiman

Average dissimilarity = 75,69

	Group Boca de Cote	Group Bajo Caiman	       	       	        	     

Species	          Av.Abund	         Av.Abund	Av.Diss	Diss/SD	Contrib%	Cum.%

Siderastrea siderea	              2,54	             0,00	  13,69	   4,46	   18,09	18,09

Orbicella faveolata	              3,10	             1,12	  10,65	   2,26	   14,07	32,16

Montastrea cavernosa	              1,76	             0,00	   9,36	   2,32	   12,37	44,53

Diploria labyrinthiformis	              1,37	             0,00	   7,62	   2,56	   10,06	54,59

Porites astreoides	              1,92	             1,01	   7,16	   1,52	    9,46	64,05

Orbicella annularis	              1,35	             0,45	   5,91	   1,52	    7,81	71,85

Madracis mirabilis	              1,01	             0,00	   4,91	   0,74	    6,49	78,34

Groups Dos Mosquises Sur  &  Bajo Caiman

Average dissimilarity = 76,14

	Group Dos Mosquises Sur	Group Bajo Caiman	       	       	        	     

Species	               Av.Abund	         Av.Abund	Av.Diss	Diss/SD	Contrib%	Cum.%

Madracis mirabilis	                   2,55	             0,00	  14,29	   1,60	   18,77	18,77

Orbicella faveolata	                   3,41	             1,12	  14,25	   2,09	   18,71	37,48

Colpophyllia natans	                   2,54	             0,45	  12,91	   1,99	   16,95	54,44

Orbicella annularis	                   2,43	             0,45	  12,63	   1,57	   16,59	71,03

Porites astreoides	                   0,17	             1,01	   5,79	   0,94	    7,60	78,63

Groups Madrisqui  &  Bajo Caiman

Average dissimilarity = 68,71

	Group Madrisqui	Group Bajo Caiman	       	       	        	     

Species	       Av.Abund	         Av.Abund	Av.Diss	Diss/SD	Contrib%	Cum.%

Colpophyllia natans	           1,96	             0,45	  13,16	   1,53	   19,15	19,15

Orbicella faveolata	           2,67	             1,12	  12,16	   2,30	   17,70	36,85

Porites astreoides	           1,41	             1,01	   9,47	   1,30	   13,78	50,63

Agaricia sp.	           0,93	             0,00	   6,28	   0,75	    9,14	59,77

Eusmilia fastigata	           0,68	             0,00	   4,91	   0,95	    7,15	66,92

Montastrea cavernosa	           0,52	             0,00	   4,76	   0,55	    6,93	73,85

Orbicella annularis	           0,00	             0,45	   3,27	   0,93	    4,76	78,61

Groups Pelona de Rabusqui  &  Bajo Caiman

Average dissimilarity = 75,43

	Group Pelona de Rabusqui	Group Bajo Caiman	       	       	        	     

Species	                Av.Abund	         Av.Abund	Av.Diss	Diss/SD	Contrib%	Cum.%

Orbicella faveolata	                    4,91	             1,12	  26,50	   3,90	   35,13	35,13

Orbicella annularis	                    2,00	             0,45	  12,08	   1,56	   16,02	51,15

Colpophyllia natans	                    1,87	             0,45	  10,74	   1,77	   14,23	65,39

Agaricia sp.	                    1,10	             0,00	   7,67	   1,44	   10,17	75,55

Groups Salinas  &  Bajo Caiman

Average dissimilarity = 77,81

	Group Salinas	Group Bajo Caiman	       	       	        	     

Species	     Av.Abund	         Av.Abund	Av.Diss	Diss/SD	Contrib%	Cum.%

Siderastrea siderea	         2,20	             0,00	  15,78	   2,39	   20,28	20,28

Montastrea cavernosa	         1,66	             0,00	  10,82	   1,64	   13,90	34,18

Diploria labyrinthiformis	         1,40	             0,00	   9,39	   1,46	   12,07	46,25

Orbicella faveolata	         2,24	             1,12	   8,82	   1,11	   11,33	57,59

Pseudodiploria strigosa	         1,32	             0,00	   8,69	   1,51	   11,17	68,76

Porites astreoides	         0,68	             1,01	   7,27	   1,16	    9,34	78,10

Groups La Venada  &  Bajo Caiman

Average dissimilarity = 100,00

	Group La Venada	Group Bajo Caiman	       	       	        	     

Species	       Av.Abund	         Av.Abund	Av.Diss	Diss/SD	Contrib%	Cum.%

Acropora cervicornis	           4,54	             0,00	  53,89	   4,57	   53,89	53,89

Orbicella faveolata	           0,00	             1,12	  13,14	   3,35	   13,14	67,03

Porites astreoides	           0,00	             1,01	  10,56	   0,86	   10,56	77,59

Groups San Agustin  &  Bajo Caiman

Average dissimilarity = 100,00

	Group San Agustin	Group Bajo Caiman	       	       	        	     

Species	         Av.Abund	         Av.Abund	Av.Diss	Diss/SD	Contrib%	Cum.%

Orbicella faveolata	             0,00	             1,12	  42,99	   1,44	   42,99	42,99

Porites astreoides	             0,00	             1,01	  23,97	   0,83	   23,97	66,96

Colpophyllia natans	             0,00	             0,45	  13,46	   0,56	   13,46	80,43

Groups Blanca  &  Bajo Caiman

Average dissimilarity = 90,44

	Group Blanca	Group Bajo Caiman	       	       	        	     

Species	    Av.Abund	         Av.Abund	Av.Diss	Diss/SD	Contrib%	Cum.%

Colpophyllia natans	        3,84	             0,45	  22,49	   3,19	   24,86	24,86

Pseudodiploria strigosa	        2,94	             0,00	  19,46	   4,42	   21,52	46,38

Montastrea cavernosa	        1,70	             0,00	  11,33	   3,29	   12,53	58,91

Orbicella faveolata	        0,00	             1,12	   7,35	   3,29	    8,13	67,04

Dichocoenia stokesii	        1,17	             0,00	   7,20	   1,58	    7,96	75,00

Groups Carabela  &  Bajo Caiman

Average dissimilarity = 96,15

	Group Carabela	Group Bajo Caiman	       	       	        	     

Species	      Av.Abund	         Av.Abund	Av.Diss	Diss/SD	Contrib%	Cum.%

Orbicella faveolata	          0,00	             1,12	  36,45	   1,35	   37,91	37,91

Porites astreoides	          0,00	             1,01	  21,50	   0,81	   22,36	60,27

Colpophyllia natans	          0,66	             0,45	  20,07	   0,77	   20,87	81,15

Groups Punta Cruz  &  Bajo Caiman

Average dissimilarity = 91,28

	Group Punta Cruz	Group Bajo Caiman	       	       	        	     

Species	        Av.Abund	         Av.Abund	Av.Diss	Diss/SD	Contrib%	Cum.%

Montastrea cavernosa	            1,66	             0,00	  19,94	   1,84	   21,85	21,85

Dichocoenia stokesii	            1,29	             0,00	  14,85	   1,50	   16,27	38,12

Orbicella faveolata	            0,17	             1,12	  11,49	   1,91	   12,59	50,71

Porites astreoides	            0,00	             1,01	  10,72	   0,85	   11,74	62,45

Pseudodiploria strigosa	            1,07	             0,00	  10,57	   0,88	   11,58	74,02

Colpophyllia natans	            0,85	             0,45	  10,46	   1,18	   11,46	85,48

Groups Gabarra  &  Bajo Caiman

Average dissimilarity = 91,65

	Group Gabarra	Group Bajo Caiman	       	       	        	     

Species	     Av.Abund	         Av.Abund	Av.Diss	Diss/SD	Contrib%	Cum.%

Pseudodiploria strigosa	         1,57	             0,00	  23,42	   2,32	   25,55	25,55

Orbicella faveolata	         0,00	             1,12	  18,92	   1,50	   20,64	46,20

Colpophyllia natans	         1,17	             0,45	  15,36	   1,08	   16,76	62,96

Porites astreoides	         0,17	             1,01	  14,00	   0,84	   15,28	78,24

Groups Garrapata  &  Bajo Caiman

Average dissimilarity = 86,18

	Group Garrapata	Group Bajo Caiman	       	       	        	     

Species	       Av.Abund	         Av.Abund	Av.Diss	Diss/SD	Contrib%	Cum.%

Pseudodiploria strigosa	           3,23	             0,00	  22,30	   6,80	   25,88	25,88

Colpophyllia natans	           3,62	             0,45	  22,00	   2,66	   25,52	51,40

Montastrea cavernosa	           2,71	             0,00	  18,75	   6,50	   21,75	73,15

Porites astreoides	           0,62	             1,01	   6,83	   1,42	    7,93	81,08

Groups Boca Seca  &  Bajo Caiman

Average dissimilarity = 71,18

	Group Boca Seca	Group Bajo Caiman	       	       	        	     

Species	       Av.Abund	         Av.Abund	Av.Diss	Diss/SD	Contrib%	Cum.%

Porites astreoides	           0,75	             1,01	  15,84	   1,23	   22,25	22,25

Orbicella faveolata	           0,90	             1,12	  14,11	   1,60	   19,83	42,08

Colpophyllia natans	           0,49	             0,45	  10,43	   0,73	   14,66	56,74

Agaricia humilis	           0,52	             0,00	   9,57	   1,32	   13,45	70,18

Orbicella annularis	           0,00	             0,45	   6,13	   0,91	    8,61	78,79

Groups La Pared  &  Medio

Average dissimilarity = 95,74

	Group La Pared	Group Medio	       	       	        	     

Species	      Av.Abund	   Av.Abund	Av.Diss	Diss/SD	Contrib%	Cum.%

Orbicella faveolata	          0,00	       4,49	  23,40	   3,98	   24,44	24,44

Pseudodiploria strigosa	          2,33	       0,17	  11,14	   2,38	   11,64	36,08

Meandrina meandrites	          1,94	       0,00	   9,52	   1,52	    9,94	46,02

Siderastrea siderea	          1,37	       0,00	   7,08	   4,35	    7,39	53,41

Montastrea cavernosa	          1,38	       0,00	   6,75	   1,52	    7,05	60,46

Agaricia sp.	          0,17	       1,23	   5,40	   1,23	    5,64	66,10

Madracis sp.	          0,00	       1,01	   4,23	   0,56	    4,42	70,53

Diploria labyrinthiformis	          0,72	       0,00	   3,79	   0,94	    3,96	74,48

Other (Scleractinian massive)	          0,62	       0,00	   3,37	   0,90	    3,52	78,01

Groups Media Legua  &  Medio

Average dissimilarity = 92,78

	Group Media Legua	Group Medio	       	       	        	     

Species	         Av.Abund	   Av.Abund	Av.Diss	Diss/SD	Contrib%	Cum.%

Orbicella faveolata	             0,17	       4,49	  30,84	   3,43	   33,24	33,24

Pseudodiploria strigosa	             2,20	       0,17	  14,15	   2,86	   15,25	48,49

Agaricia sp.	             0,17	       1,23	   7,13	   1,29	    7,68	56,17

Siderastrea siderea	             0,85	       0,00	   5,91	   1,39	    6,37	62,54

Madracis sp.	             0,00	       1,01	   5,39	   0,56	    5,81	68,35

Agaricia agaricites (danai)	             0,00	       0,75	   4,29	   0,82	    4,62	72,97

Meandrina meandrites	             0,60	       0,00	   4,23	   0,55	    4,56	77,53

Groups Punta de Media Legua  &  Medio

Average dissimilarity = 97,94

	Group Punta de Media Legua	Group Medio	       	       	        	     

Species	                  Av.Abund	   Av.Abund	Av.Diss	Diss/SD	Contrib%	Cum.%

Orbicella faveolata	                      0,00	       4,49	  31,22	   2,49	   31,88	31,88

Siderastrea siderea	                      1,59	       0,00	   9,23	   2,44	    9,42	41,30

Pseudodiploria strigosa	                      1,63	       0,17	   9,21	   1,65	    9,40	50,70

Agaricia sp.	                      0,00	       1,23	   7,51	   1,27	    7,67	58,37

Montastrea cavernosa	                      1,14	       0,00	   6,93	   1,37	    7,08	65,45

Madracis sp.	                      0,00	       1,01	   5,21	   0,54	    5,32	70,77

Agaricia agaricites (danai)	                      0,00	       0,75	   4,15	   0,79	    4,24	75,02

Groups Petaquire  &  Medio

Average dissimilarity = 92,10

	Group Petaquire	Group Medio	       	       	        	     

Species	       Av.Abund	   Av.Abund	Av.Diss	Diss/SD	Contrib%	Cum.%

Orbicella faveolata	           0,23	       4,49	  30,39	   2,80	   33,00	33,00

Pseudodiploria strigosa	           2,75	       0,17	  17,54	   3,68	   19,05	52,05

Agaricia sp.	           0,23	       1,23	   6,97	   1,22	    7,57	59,62

Siderastrea siderea	           0,92	       0,00	   6,21	   3,15	    6,74	66,36

Madracis sp.	           0,00	       1,01	   5,34	   0,55	    5,80	72,15

Agaricia agaricites (danai)	           0,00	       0,75	   4,25	   0,81	    4,61	76,76

Groups Playa Tiburon  &  Medio

Average dissimilarity = 92,25

	Group Playa Tiburon	Group Medio	       	       	        	     

Species	           Av.Abund	   Av.Abund	Av.Diss	Diss/SD	Contrib%	Cum.%

Orbicella faveolata	               0,00	       4,49	  26,22	   3,76	   28,42	28,42

Pseudodiploria strigosa	               2,38	       0,17	  12,60	   3,05	   13,66	42,09

Siderastrea siderea	               1,64	       0,00	   9,39	   2,36	   10,18	52,26

Diploria labyrinthiformis	               1,38	       0,00	   7,77	   2,66	    8,43	60,69

Agaricia sp.	               0,35	       1,23	   5,49	   1,20	    5,95	66,64

Montastrea cavernosa	               0,93	       0,00	   5,31	   1,30	    5,76	72,40

Madracis sp.	               0,00	       1,01	   4,63	   0,56	    5,02	77,42

Groups Punta Mono  &  Medio

Average dissimilarity = 97,95

	Group Punta Mono	Group Medio	       	       	        	     

Species	        Av.Abund	   Av.Abund	Av.Diss	Diss/SD	Contrib%	Cum.%

Orbicella faveolata	            0,00	       4,49	  27,02	   3,50	   27,59	27,59

Pseudodiploria strigosa	            2,96	       0,17	  16,06	   4,19	   16,39	43,98

Siderastrea siderea	            1,34	       0,00	   7,83	   3,19	    8,00	51,98

Meandrina meandrites	            1,21	       0,00	   7,19	   1,57	    7,34	59,32

Agaricia sp.	            0,00	       1,23	   6,63	   1,30	    6,77	66,08

Diploria labyrinthiformis	            1,02	       0,00	   5,92	   2,88	    6,04	72,13

Madracis sp.	            0,00	       1,01	   4,73	   0,56	    4,83	76,96

Groups Charagato  &  Medio

Average dissimilarity = 95,25

	Group Charagato	Group Medio	       	       	        	     

Species	       Av.Abund	   Av.Abund	Av.Diss	Diss/SD	Contrib%	Cum.%

Orbicella faveolata	           0,00	       4,49	  41,11	   2,60	   43,15	43,15

Colpophyllia natans	           1,86	       0,35	  13,29	   1,36	   13,95	57,10

Agaricia sp.	           0,00	       1,23	   9,60	   1,37	   10,08	67,18

Madracis sp.	           0,00	       1,01	   6,36	   0,55	    6,68	73,86

Agaricia agaricites (danai)	           0,00	       0,75	   5,16	   0,82	    5,41	79,28

Groups Punta Conejo  &  Medio

Average dissimilarity = 97,88

	Group Punta Conejo	Group Medio	       	       	        	     

Species	          Av.Abund	   Av.Abund	Av.Diss	Diss/SD	Contrib%	Cum.%

Orbicella faveolata	              0,00	       4,49	  40,65	   2,77	   41,54	41,54

Pseudodiploria strigosa	              1,99	       0,17	  14,90	   1,35	   15,22	56,75

Agaricia sp.	              0,00	       1,23	   9,52	   1,38	    9,73	66,48

Madracis sp.	              0,00	       1,01	   6,34	   0,55	    6,47	72,95

Agaricia agaricites (danai)	              0,00	       0,75	   5,13	   0,83	    5,24	78,19

Groups La Muerta  &  Medio

Average dissimilarity = 92,47

	Group La Muerta	Group Medio	       	       	        	     

Species	       Av.Abund	   Av.Abund	Av.Diss	Diss/SD	Contrib%	Cum.%

Orbicella faveolata	           0,35	       4,49	  36,70	   2,09	   39,69	39,69

Pseudodiploria strigosa	           1,99	       0,17	  14,09	   1,66	   15,23	54,92

Agaricia sp.	           0,00	       1,23	   9,13	   1,34	    9,88	64,80

Madracis sp.	           0,00	       1,01	   6,11	   0,55	    6,61	71,41

Colpophyllia natans	           0,62	       0,35	   6,09	   0,78	    6,59	77,99

Groups Cominoto  &  Medio

Average dissimilarity = 97,26

	Group Cominoto	Group Medio	       	       	        	     

Species	      Av.Abund	   Av.Abund	Av.Diss	Diss/SD	Contrib%	Cum.%

Orbicella faveolata	          0,00	       4,49	  36,65	   3,49	   37,68	37,68

Pseudodiploria strigosa	          2,96	       0,17	  22,42	   3,14	   23,05	60,73

Agaricia sp.	          0,00	       1,23	   8,71	   1,39	    8,96	69,69

Madracis sp.	          0,00	       1,01	   5,94	   0,56	    6,11	75,80

Groups La Pecha  &  Medio

Average dissimilarity = 99,12

	Group La Pecha	Group Medio	       	       	        	     

Species	      Av.Abund	   Av.Abund	Av.Diss	Diss/SD	Contrib%	Cum.%

Orbicella faveolata	          0,00	       4,49	  51,47	   2,84	   51,93	51,93

Agaricia sp.	          0,00	       1,23	  11,70	   1,49	   11,81	63,73

Madracis sp.	          0,00	       1,01	   7,41	   0,56	    7,48	71,21

Agaricia tenuifolia	          0,00	       0,58	   6,23	   0,56	    6,29	77,50

Groups Puerto Real  &  Medio

Average dissimilarity = 95,70

	Group Puerto Real	Group Medio	       	       	        	     

Species	         Av.Abund	   Av.Abund	Av.Diss	Diss/SD	Contrib%	Cum.%

Orbicella faveolata	             0,00	       4,49	  34,03	   3,53	   35,56	35,56

Pseudodiploria strigosa	             2,60	       0,17	  17,69	   2,35	   18,48	54,05

Porites astreoides	             1,10	       0,00	   8,19	   4,95	    8,56	62,61

Agaricia sp.	             0,17	       1,23	   7,57	   1,29	    7,91	70,52

Madracis sp.	             0,00	       1,01	   5,63	   0,56	    5,89	76,41

Groups Cayo Agua  &  Medio

Average dissimilarity = 68,86

	Group Cayo Agua	Group Medio	       	       	        	     

Species	       Av.Abund	   Av.Abund	Av.Diss	Diss/SD	Contrib%	Cum.%

Madracis mirabilis	           3,47	       0,00	  14,43	   4,01	   20,95	20,95

Orbicella annularis	           3,05	       0,00	  12,65	   3,37	   18,37	39,32

Orbicella faveolata	           3,24	       4,49	   5,45	   2,06	    7,91	47,23

Agaricia sp.	           0,00	       1,23	   4,80	   1,26	    6,97	54,20

Madracis decactis	           1,10	       0,17	   4,45	   1,09	    6,46	60,66

Colpophyllia natans	           1,15	       0,35	   4,35	   1,24	    6,32	66,98

Madracis sp.	           0,17	       1,01	   3,96	   0,66	    5,75	72,73

Porites astreoides	           0,69	       0,00	   3,04	   0,77	    4,42	77,14

Groups Boca de Cote  &  Medio

Average dissimilarity = 69,75

	Group Boca de Cote	Group Medio	       	       	        	     

Species	          Av.Abund	   Av.Abund	Av.Diss	Diss/SD	Contrib%	Cum.%

Siderastrea siderea	              2,54	       0,00	  10,29	   4,41	   14,75	14,75

Porites astreoides	              1,92	       0,00	   7,86	   4,01	   11,26	26,02

Montastrea cavernosa	              1,76	       0,00	   7,05	   2,28	   10,11	36,13

Orbicella faveolata	              3,10	       4,49	   6,12	   1,55	    8,77	44,90

Diploria labyrinthiformis	              1,37	       0,00	   5,68	   2,63	    8,15	53,05

Orbicella annularis	              1,35	       0,00	   5,26	   1,51	    7,54	60,59

Agaricia sp.	              0,00	       1,23	   4,68	   1,25	    6,71	67,29

Madracis mirabilis	              1,01	       0,00	   3,78	   0,73	    5,42	72,72

Madracis sp.	              0,00	       1,01	   3,49	   0,56	    5,01	77,72

Groups Dos Mosquises Sur  &  Medio

Average dissimilarity = 65,47

	Group Dos Mosquises Sur	Group Medio	       	       	        	     

Species	               Av.Abund	   Av.Abund	Av.Diss	Diss/SD	Contrib%	Cum.%

Madracis mirabilis	                   2,55	       0,00	  10,66	   1,58	   16,27	16,27

Orbicella annularis	                   2,43	       0,00	  10,18	   1,46	   15,55	31,82

Colpophyllia natans	                   2,54	       0,35	   9,80	   2,46	   14,96	46,78

Orbicella faveolata	                   3,41	       4,49	   5,25	   1,54	    8,03	54,81

Agaricia sp.	                   0,00	       1,23	   5,04	   1,26	    7,70	62,51

Madracis sp.	                   0,17	       1,01	   4,17	   0,67	    6,38	68,89

Agaricia tenuifolia	                   0,17	       0,58	   2,92	   0,70	    4,47	73,35

Agaricia agaricites (danai)	                   0,00	       0,75	   2,90	   0,80	    4,43	77,78

Groups Madrisqui  &  Medio

Average dissimilarity = 63,69

	Group Madrisqui	Group Medio	       	       	        	     

Species	       Av.Abund	   Av.Abund	Av.Diss	Diss/SD	Contrib%	Cum.%

Orbicella faveolata	           2,67	       4,49	  10,12	   1,96	   15,89	15,89

Colpophyllia natans	           1,96	       0,35	   9,24	   1,64	   14,51	30,40

Porites astreoides	           1,41	       0,00	   7,34	   1,54	   11,52	41,92

Agaricia sp.	           0,93	       1,23	   6,46	   1,29	   10,14	52,06

Madracis sp.	           0,00	       1,01	   4,36	   0,56	    6,85	58,91

Agaricia agaricites (danai)	           0,35	       0,75	   4,09	   1,02	    6,42	65,33

Eusmilia fastigata	           0,68	       0,00	   3,40	   0,94	    5,34	70,67

Agaricia tenuifolia	           0,00	       0,58	   3,08	   0,56	    4,83	75,51

Groups Pelona de Rabusqui  &  Medio

Average dissimilarity = 44,97

	Group Pelona de Rabusqui	Group Medio	       	       	        	     

Species	                Av.Abund	   Av.Abund	Av.Diss	Diss/SD	Contrib%	Cum.%

Orbicella annularis	                    2,00	       0,00	   9,53	   1,49	   21,20	21,20

Colpophyllia natans	                    1,87	       0,35	   7,69	   1,80	   17,10	38,30

Agaricia sp.	                    1,10	       1,23	   4,58	   1,26	   10,18	48,48

Madracis sp.	                    0,00	       1,01	   4,04	   0,56	    8,98	57,47

Agaricia agaricites (danai)	                    0,00	       0,75	   3,15	   0,80	    7,01	64,48

Orbicella sp.	                    0,74	       0,00	   3,12	   0,55	    6,95	71,42

Agaricia tenuifolia	                    0,00	       0,58	   2,80	   0,55	    6,23	77,66

Groups Salinas  &  Medio

Average dissimilarity = 74,21

	Group Salinas	Group Medio	       	       	        	     

Species	     Av.Abund	   Av.Abund	Av.Diss	Diss/SD	Contrib%	Cum.%

Orbicella faveolata	         2,24	       4,49	  11,41	   1,87	   15,37	15,37

Siderastrea siderea	         2,20	       0,00	  10,92	   2,44	   14,72	30,09

Montastrea cavernosa	         1,66	       0,00	   7,72	   1,63	   10,40	40,49

Diploria labyrinthiformis	         1,40	       0,00	   6,65	   1,49	    8,96	49,45

Pseudodiploria strigosa	         1,32	       0,17	   5,84	   1,55	    7,87	57,32

Agaricia sp.	         0,00	       1,23	   5,65	   1,27	    7,61	64,94

Madracis sp.	         0,00	       1,01	   4,13	   0,55	    5,56	70,50

Porites astreoides	         0,68	       0,00	   3,27	   0,95	    4,40	74,90

Agaricia agaricites (danai)	         0,00	       0,75	   3,22	   0,80	    4,34	79,24

Groups La Venada  &  Medio

Average dissimilarity = 96,07

	Group La Venada	Group Medio	       	       	        	     

Species	       Av.Abund	   Av.Abund	Av.Diss	Diss/SD	Contrib%	Cum.%

Orbicella faveolata	           0,00	       4,49	  31,48	   3,65	   32,76	32,76

Acropora cervicornis	           4,54	       0,00	  31,29	   5,20	   32,56	65,33

Agaricia sp.	           0,17	       1,23	   7,00	   1,29	    7,29	72,62

Madracis sp.	           0,00	       1,01	   5,32	   0,56	    5,54	78,16

Groups San Agustin  &  Medio

Average dissimilarity = 100,00

	Group San Agustin	Group Medio	       	       	        	     

Species	         Av.Abund	   Av.Abund	Av.Diss	Diss/SD	Contrib%	Cum.%

Orbicella faveolata	             0,00	       4,49	  51,47	   2,84	   51,47	51,47

Agaricia sp.	             0,00	       1,23	  11,70	   1,49	   11,70	63,17

Madracis sp.	             0,00	       1,01	   7,41	   0,56	    7,41	70,58

Agaricia tenuifolia	             0,00	       0,58	   6,23	   0,56	    6,23	76,82

Groups Blanca  &  Medio

Average dissimilarity = 95,11

	Group Blanca	Group Medio	       	       	        	     

Species	    Av.Abund	   Av.Abund	Av.Diss	Diss/SD	Contrib%	Cum.%

Orbicella faveolata	        0,00	       4,49	  21,33	   4,01	   22,43	22,43

Colpophyllia natans	        3,84	       0,35	  16,44	   3,89	   17,29	39,72

Pseudodiploria strigosa	        2,94	       0,17	  13,02	   4,05	   13,69	53,40

Montastrea cavernosa	        1,70	       0,00	   8,04	   3,52	    8,45	61,86

Agaricia sp.	        0,00	       1,23	   5,34	   1,27	    5,62	67,47

Dichocoenia stokesii	        1,17	       0,00	   5,22	   1,56	    5,49	72,97

Madracis sp.	        0,00	       1,01	   3,93	   0,56	    4,13	77,10

Groups Carabela  &  Medio

Average dissimilarity = 98,52

	Group Carabela	Group Medio	       	       	        	     

Species	      Av.Abund	   Av.Abund	Av.Diss	Diss/SD	Contrib%	Cum.%

Orbicella faveolata	          0,00	       4,49	  48,25	   2,77	   48,98	48,98

Agaricia sp.	          0,00	       1,23	  11,06	   1,45	   11,22	60,20

Colpophyllia natans	          0,66	       0,35	   7,78	   0,78	    7,89	68,09

Madracis sp.	          0,00	       1,01	   7,10	   0,56	    7,21	75,30

Groups Punta Cruz  &  Medio

Average dissimilarity = 93,93

	Group Punta Cruz	Group Medio	       	       	        	     

Species	        Av.Abund	   Av.Abund	Av.Diss	Diss/SD	Contrib%	Cum.%

Orbicella faveolata	            0,17	       4,49	  30,57	   3,25	   32,55	32,55

Montastrea cavernosa	            1,66	       0,00	  11,47	   2,01	   12,21	44,76

Dichocoenia stokesii	            1,29	       0,00	   8,70	   1,55	    9,27	54,02

Agaricia sp.	            0,00	       1,23	   7,66	   1,34	    8,15	62,18

Pseudodiploria strigosa	            1,07	       0,17	   6,72	   0,98	    7,15	69,33

Colpophyllia natans	            0,85	       0,35	   5,54	   1,21	    5,90	75,22

Groups Gabarra  &  Medio

Average dissimilarity = 94,17

	Group Gabarra	Group Medio	       	       	        	     

Species	     Av.Abund	   Av.Abund	Av.Diss	Diss/SD	Contrib%	Cum.%

Orbicella faveolata	         0,00	       4,49	  36,76	   2,79	   39,04	39,04

Pseudodiploria strigosa	         1,57	       0,17	  10,61	   1,75	   11,26	50,30

Agaricia sp.	         0,00	       1,23	   8,71	   1,35	    9,25	59,55

Colpophyllia natans	         1,17	       0,35	   8,07	   1,27	    8,57	68,12

Madracis sp.	         0,00	       1,01	   5,91	   0,55	    6,28	74,40

Siderastrea siderea	         0,76	       0,00	   5,38	   0,89	    5,71	80,11

Groups Garrapata  &  Medio

Average dissimilarity = 91,54

	Group Garrapata	Group Medio	       	       	        	     

Species	       Av.Abund	   Av.Abund	Av.Diss	Diss/SD	Contrib%	Cum.%

Orbicella faveolata	           0,35	       4,49	  20,45	   3,65	   22,34	22,34

Colpophyllia natans	           3,62	       0,35	  15,98	   2,96	   17,45	39,79

Pseudodiploria strigosa	           3,23	       0,17	  14,88	   4,91	   16,25	56,05

Montastrea cavernosa	           2,71	       0,00	  13,21	   5,94	   14,43	70,47

Agaricia sp.	           0,00	       1,23	   5,53	   1,29	    6,05	76,52

Groups Boca Seca  &  Medio

Average dissimilarity = 75,44

	Group Boca Seca	Group Medio	       	       	        	     

Species	       Av.Abund	   Av.Abund	Av.Diss	Diss/SD	Contrib%	Cum.%

Orbicella faveolata	           0,90	       4,49	  30,34	   2,11	   40,21	40,21

Agaricia sp.	           0,17	       1,23	   8,21	   1,29	   10,88	51,09

Madracis sp.	           0,00	       1,01	   5,97	   0,56	    7,91	59,01

Porites astreoides	           0,75	       0,00	   5,65	   1,26	    7,49	66,50

Colpophyllia natans	           0,49	       0,35	   5,04	   0,78	    6,68	73,18

Agaricia agaricites (danai)	           0,00	       0,75	   4,80	   0,82	    6,36	79,54

Groups Bajo Caiman  &  Medio

Average dissimilarity = 80,01

	Group Bajo Caiman	Group Medio	       	       	        	     

Species	         Av.Abund	   Av.Abund	Av.Diss	Diss/SD	Contrib%	Cum.%

Orbicella faveolata	             1,12	       4,49	  28,99	   2,64	   36,24	36,24

Agaricia sp.	             0,00	       1,23	   9,04	   1,38	   11,30	47,54

Porites astreoides	             1,01	       0,00	   7,72	   0,84	    9,65	57,19

Madracis sp.	             0,00	       1,01	   6,10	   0,56	    7,63	64,82

Colpophyllia natans	             0,45	       0,35	   5,15	   0,76	    6,43	71,25

Agaricia agaricites (danai)	             0,00	       0,75	   4,91	   0,83	    6,14	77,39

Groups La Pared  &  Mero

Average dissimilarity = 86,71

	Group La Pared	Group Mero	       	       	        	     

Species	      Av.Abund	  Av.Abund	Av.Diss	Diss/SD	Contrib%	Cum.%

Pseudodiploria strigosa	          2,33	      0,17	  15,86	   1,85	   18,29	18,29

Meandrina meandrites	          1,94	      0,00	  12,81	   1,41	   14,78	33,07

Montastrea cavernosa	          1,38	      0,00	   9,08	   1,42	   10,47	43,54

Siderastrea siderea	          1,37	      0,62	   6,60	   1,07	    7,61	51,14

Porites astreoides	          0,45	      1,14	   6,04	   1,32	    6,97	58,11

Colpophyllia natans	          0,00	      1,06	   5,47	   0,85	    6,31	64,42

Diploria labyrinthiformis	          0,72	      0,00	   5,30	   0,90	    6,11	70,53

Other (Scleractinian massive)	          0,62	      0,00	   4,80	   0,83	    5,54	76,07

Groups Media Legua  &  Mero

Average dissimilarity = 86,96

	Group Media Legua	Group Mero	       	       	        	     

Species	         Av.Abund	  Av.Abund	Av.Diss	Diss/SD	Contrib%	Cum.%

Pseudodiploria strigosa	             2,20	      0,17	  24,68	   1,75	   28,38	28,38

Porites astreoides	             0,17	      1,14	   9,95	   1,43	   11,45	39,83

Siderastrea siderea	             0,85	      0,62	   8,79	   1,01	   10,11	49,94

Colpophyllia natans	             0,17	      1,06	   8,13	   1,04	    9,35	59,29

Meandrina meandrites	             0,60	      0,00	   7,09	   0,51	    8,16	67,45

Porites porites	             0,00	      0,55	   5,13	   0,87	    5,90	73,34

Montastrea cavernosa	             0,45	      0,00	   4,76	   0,84	    5,47	78,81

Groups Punta de Media Legua  &  Mero

Average dissimilarity = 88,62

	Group Punta de Media Legua	Group Mero	       	       	        	     

Species	                  Av.Abund	  Av.Abund	Av.Diss	Diss/SD	Contrib%	Cum.%

Pseudodiploria strigosa	                      1,63	      0,17	  16,23	   0,99	   18,31	18,31

Siderastrea siderea	                      1,59	      0,62	  13,01	   1,47	   14,67	32,99

Montastrea cavernosa	                      1,14	      0,00	  11,16	   1,04	   12,59	45,58

Porites astreoides	                      0,27	      1,14	   9,74	   1,10	   10,99	56,57

Colpophyllia natans	                      0,17	      1,06	   7,50	   0,90	    8,46	65,03

Diploria labyrinthiformis	                      0,83	      0,00	   5,46	   0,75	    6,16	71,19

Porites porites	                      0,00	      0,55	   5,17	   0,76	    5,83	77,02

Groups Petaquire  &  Mero

Average dissimilarity = 88,15

	Group Petaquire	Group Mero	       	       	        	     

Species	       Av.Abund	  Av.Abund	Av.Diss	Diss/SD	Contrib%	Cum.%

Pseudodiploria strigosa	           2,75	      0,17	  30,91	   1,69	   35,06	35,06

Porites astreoides	           0,00	      1,14	  10,33	   1,32	   11,72	46,79

Siderastrea siderea	           0,92	      0,62	   8,67	   1,25	    9,83	56,62

Colpophyllia natans	           0,23	      1,06	   7,96	   1,02	    9,03	65,64

Porites porites	           0,00	      0,55	   5,14	   0,82	    5,83	71,47

Orbicella faveolata	           0,23	      0,45	   4,19	   0,97	    4,76	76,23

Groups Playa Tiburon  &  Mero

Average dissimilarity = 86,52

	Group Playa Tiburon	Group Mero	       	       	        	     

Species	           Av.Abund	  Av.Abund	Av.Diss	Diss/SD	Contrib%	Cum.%

Pseudodiploria strigosa	               2,38	      0,17	  18,94	   2,11	   21,90	21,90

Diploria labyrinthiformis	               1,38	      0,00	  11,23	   2,21	   12,98	34,88

Siderastrea siderea	               1,64	      0,62	  10,29	   1,10	   11,90	46,77

Porites astreoides	               0,40	      1,14	   7,78	   1,28	    8,99	55,76

Montastrea cavernosa	               0,93	      0,00	   7,77	   1,19	    8,98	64,74

Colpophyllia natans	               0,00	      1,06	   6,12	   0,86	    7,08	71,82

Meandrina meandrites	               0,75	      0,00	   5,67	   0,76	    6,55	78,37

Groups Punta Mono  &  Mero

Average dissimilarity = 88,59

	Group Punta Mono	Group Mero	       	       	        	     

Species	        Av.Abund	  Av.Abund	Av.Diss	Diss/SD	Contrib%	Cum.%

Pseudodiploria strigosa	            2,96	      0,17	  24,32	   2,51	   27,45	27,45

Meandrina meandrites	            1,21	      0,00	  10,84	   1,39	   12,24	39,69

Diploria labyrinthiformis	            1,02	      0,00	   8,77	   2,26	    9,90	49,60

Siderastrea siderea	            1,34	      0,62	   8,36	   1,08	    9,43	59,03

Porites astreoides	            0,17	      1,14	   7,98	   1,38	    9,01	68,04

Colpophyllia natans	            0,00	      1,06	   6,31	   0,86	    7,12	75,16

Groups Charagato  &  Mero

Average dissimilarity = 86,06

	Group Charagato	Group Mero	       	       	        	     

Species	       Av.Abund	  Av.Abund	Av.Diss	Diss/SD	Contrib%	Cum.%

Colpophyllia natans	           1,86	      1,06	  29,98	   1,08	   34,84	34,84

Porites astreoides	           0,00	      1,14	  16,15	   1,32	   18,77	53,60

Porites porites	           0,00	      0,55	   8,45	   0,76	    9,82	63,42

Siderastrea siderea	           0,27	      0,62	   7,51	   1,02	    8,73	72,15

Orbicella faveolata	           0,00	      0,45	   6,17	   0,86	    7,17	79,32

Groups Punta Conejo  &  Mero

Average dissimilarity = 94,28

	Group Punta Conejo	Group Mero	       	       	        	     

Species	          Av.Abund	  Av.Abund	Av.Diss	Diss/SD	Contrib%	Cum.%

Pseudodiploria strigosa	              1,99	      0,17	  31,58	   1,02	   33,50	33,50

Porites astreoides	              0,17	      1,14	  14,35	   1,26	   15,22	48,72

Colpophyllia natans	              0,00	      1,06	   9,48	   0,88	   10,05	58,77

Oculina diffusa	              0,17	      0,00	   8,52	   0,34	    9,04	67,81

Porites porites	              0,00	      0,55	   7,62	   0,76	    8,09	75,89

Groups La Muerta  &  Mero

Average dissimilarity = 88,07

	Group La Muerta	Group Mero	       	       	        	     

Species	       Av.Abund	  Av.Abund	Av.Diss	Diss/SD	Contrib%	Cum.%

Pseudodiploria strigosa	           1,99	      0,17	  28,70	   1,12	   32,59	32,59

Porites astreoides	           0,52	      1,14	  15,64	   1,21	   17,75	50,35

Colpophyllia natans	           0,62	      1,06	  14,16	   0,96	   16,08	66,42

Porites porites	           0,00	      0,55	   7,85	   0,73	    8,91	75,33

Groups Cominoto  &  Mero

Average dissimilarity = 89,30

	Group Cominoto	Group Mero	       	       	        	     

Species	      Av.Abund	  Av.Abund	Av.Diss	Diss/SD	Contrib%	Cum.%

Pseudodiploria strigosa	          2,96	      0,17	  44,83	   1,59	   50,20	50,20

Porites astreoides	          0,58	      1,14	  12,66	   1,16	   14,18	64,38

Colpophyllia natans	          0,00	      1,06	   8,54	   0,89	    9,56	73,95

Porites porites	          0,00	      0,55	   6,23	   0,85	    6,98	80,92

Groups La Pecha  &  Mero

Average dissimilarity = 99,05

	Group La Pecha	Group Mero	       	       	        	     

Species	      Av.Abund	  Av.Abund	Av.Diss	Diss/SD	Contrib%	Cum.%

Porites astreoides	          0,00	      1,14	  26,09	   1,92	   26,33	26,33

Colpophyllia natans	          0,00	      1,06	  14,78	   1,15	   14,92	41,25

Porites porites	          0,00	      0,55	  14,27	   0,90	   14,41	55,66

Pseudodiploria strigosa	          0,17	      0,17	  11,53	   0,42	   11,64	67,30

Orbicella faveolata	          0,00	      0,45	  10,16	   1,03	   10,26	77,56

Groups Puerto Real  &  Mero

Average dissimilarity = 81,07

	Group Puerto Real	Group Mero	       	       	        	     

Species	         Av.Abund	  Av.Abund	Av.Diss	Diss/SD	Contrib%	Cum.%

Pseudodiploria strigosa	             2,60	      0,17	  31,90	   1,71	   39,35	39,35

Porites astreoides	             1,10	      1,14	   9,22	   0,88	   11,38	50,73

Colpophyllia natans	             0,00	      1,06	   7,93	   0,88	    9,79	60,51

Porites porites	             0,00	      0,55	   5,59	   0,86	    6,90	67,41

Siderastrea siderea	             0,00	      0,62	   5,03	   0,96	    6,20	73,61

Other (Scleractinian massive)	             0,35	      0,00	   4,70	   0,81	    5,80	79,41

Groups Cayo Agua  &  Mero

Average dissimilarity = 84,17

	Group Cayo Agua	Group Mero	       	       	        	     

Species	       Av.Abund	  Av.Abund	Av.Diss	Diss/SD	Contrib%	Cum.%

Madracis mirabilis	           3,47	      0,27	  17,27	   2,56	   20,51	20,51

Orbicella annularis	           3,05	      0,00	  16,08	   2,93	   19,10	39,62

Orbicella faveolata	           3,24	      0,45	  15,32	   2,64	   18,20	57,81

Colpophyllia natans	           1,15	      1,06	   6,25	   1,24	    7,42	65,23

Madracis decactis	           1,10	      0,17	   5,59	   1,00	    6,64	71,88

Porites astreoides	           0,69	      1,14	   5,21	   1,25	    6,19	78,07

Groups Boca de Cote  &  Mero

Average dissimilarity = 77,24

	Group Boca de Cote	Group Mero	       	       	        	     

Species	          Av.Abund	  Av.Abund	Av.Diss	Diss/SD	Contrib%	Cum.%

Orbicella faveolata	              3,10	      0,45	  13,87	   2,26	   17,96	17,96

Siderastrea siderea	              2,54	      0,62	  10,38	   1,78	   13,44	31,40

Montastrea cavernosa	              1,76	      0,00	   8,90	   2,12	   11,53	42,92

Diploria labyrinthiformis	              1,37	      0,00	   7,24	   2,29	    9,37	52,29

Orbicella annularis	              1,35	      0,00	   6,58	   1,46	    8,52	60,81

Porites astreoides	              1,92	      1,14	   5,20	   1,03	    6,74	67,54

Madracis mirabilis	              1,01	      0,27	   4,91	   0,82	    6,36	73,90

Colpophyllia natans	              0,17	      1,06	   4,43	   0,93	    5,74	79,64

Groups Dos Mosquises Sur  &  Mero

Average dissimilarity = 81,55

	Group Dos Mosquises Sur	Group Mero	       	       	        	     

Species	               Av.Abund	  Av.Abund	Av.Diss	Diss/SD	Contrib%	Cum.%

Orbicella faveolata	                   3,41	      0,45	  17,71	   1,98	   21,72	21,72

Madracis mirabilis	                   2,55	      0,27	  13,09	   1,49	   16,05	37,77

Orbicella annularis	                   2,43	      0,00	  12,99	   1,39	   15,92	53,69

Colpophyllia natans	                   2,54	      1,06	  11,30	   1,42	   13,86	67,55

Porites astreoides	                   0,17	      1,14	   5,52	   1,41	    6,77	74,32

Siderastrea siderea	                   0,52	      0,62	   3,74	   0,97	    4,58	78,90

Groups Madrisqui  &  Mero

Average dissimilarity = 75,96

	Group Madrisqui	Group Mero	       	       	        	     

Species	       Av.Abund	  Av.Abund	Av.Diss	Diss/SD	Contrib%	Cum.%

Orbicella faveolata	           2,67	      0,45	  17,11	   2,02	   22,53	22,53

Colpophyllia natans	           1,96	      1,06	  12,29	   1,30	   16,17	38,70

Porites astreoides	           1,41	      1,14	   7,44	   1,07	    9,80	48,50

Agaricia sp.	           0,93	      0,00	   5,95	   0,72	    7,83	56,33

Eusmilia fastigata	           0,68	      0,00	   4,65	   0,91	    6,12	62,46

Montastrea cavernosa	           0,52	      0,00	   4,51	   0,53	    5,94	68,40

Porites porites	           0,00	      0,55	   3,56	   0,91	    4,69	73,09

Siderastrea siderea	           0,00	      0,62	   3,51	   0,96	    4,63	77,71

Groups Pelona de Rabusqui  &  Mero

Average dissimilarity = 85,79

	Group Pelona de Rabusqui	Group Mero	       	       	        	     

Species	                Av.Abund	  Av.Abund	Av.Diss	Diss/SD	Contrib%	Cum.%

Orbicella faveolata	                    4,91	      0,45	  29,96	   2,57	   34,92	34,92

Orbicella annularis	                    2,00	      0,00	  12,79	   1,40	   14,91	49,83

Colpophyllia natans	                    1,87	      1,06	  10,01	   1,46	   11,67	61,50

Agaricia sp.	                    1,10	      0,00	   7,26	   1,34	    8,47	69,96

Porites astreoides	                    0,00	      1,14	   6,54	   1,41	    7,63	77,59

Groups Salinas  &  Mero

Average dissimilarity = 79,76

	Group Salinas	Group Mero	       	       	        	     

Species	     Av.Abund	  Av.Abund	Av.Diss	Diss/SD	Contrib%	Cum.%

Orbicella faveolata	         2,24	      0,45	  13,01	   1,25	   16,32	16,32

Siderastrea siderea	         2,20	      0,62	  11,84	   1,30	   14,85	31,17

Montastrea cavernosa	         1,66	      0,00	  10,26	   1,52	   12,86	44,03

Diploria labyrinthiformis	         1,40	      0,00	   8,90	   1,36	   11,16	55,19

Pseudodiploria strigosa	         1,32	      0,17	   7,90	   1,36	    9,91	65,09

Colpophyllia natans	         0,27	      1,06	   5,91	   0,99	    7,41	72,51

Porites astreoides	         0,68	      1,14	   5,81	   1,19	    7,29	79,80

Groups La Venada  &  Mero

Average dissimilarity = 98,03

	Group La Venada	Group Mero	       	       	        	     

Species	       Av.Abund	  Av.Abund	Av.Diss	Diss/SD	Contrib%	Cum.%

Acropora cervicornis	           4,54	      0,00	  51,81	   2,27	   52,85	52,85

Porites astreoides	           0,00	      1,14	  10,12	   1,41	   10,32	63,18

Colpophyllia natans	           0,00	      1,06	   7,34	   0,87	    7,49	70,66

Porites porites	           0,00	      0,55	   5,00	   0,87	    5,10	75,77

Groups San Agustin  &  Mero

Average dissimilarity = 100,00

	Group San Agustin	Group Mero	       	       	        	     

Species	         Av.Abund	  Av.Abund	Av.Diss	Diss/SD	Contrib%	Cum.%

Porites astreoides	             0,00	      1,14	  26,09	   1,92	   26,09	26,09

Colpophyllia natans	             0,00	      1,06	  14,78	   1,15	   14,78	40,86

Porites porites	             0,00	      0,55	  14,27	   0,90	   14,27	55,14

Montastrea cavernosa	             0,17	      0,00	  10,49	   0,38	   10,49	65,63

Orbicella faveolata	             0,00	      0,45	  10,16	   1,03	   10,16	75,79

Groups Blanca  &  Mero

Average dissimilarity = 81,35

	Group Blanca	Group Mero	       	       	        	     

Species	    Av.Abund	  Av.Abund	Av.Diss	Diss/SD	Contrib%	Cum.%

Colpophyllia natans	        3,84	      1,06	  19,11	   1,59	   23,49	23,49

Pseudodiploria strigosa	        2,94	      0,17	  17,68	   2,62	   21,74	45,23

Montastrea cavernosa	        1,70	      0,00	  10,74	   2,61	   13,20	58,43

Dichocoenia stokesii	        1,17	      0,00	   6,83	   1,47	    8,40	66,83

Porites astreoides	        0,62	      1,14	   5,02	   1,52	    6,16	72,99

Siderastrea siderea	        0,52	      0,62	   3,69	   1,16	    4,54	77,53

Groups Carabela  &  Mero

Average dissimilarity = 95,26

	Group Carabela	Group Mero	       	       	        	     

Species	      Av.Abund	  Av.Abund	Av.Diss	Diss/SD	Contrib%	Cum.%

Colpophyllia natans	          0,66	      1,06	  22,37	   0,84	   23,48	23,48

Porites astreoides	          0,00	      1,14	  21,91	   1,58	   23,00	46,48

Porites porites	          0,00	      0,55	  11,81	   0,83	   12,40	58,87

Montastrea cavernosa	          0,17	      0,00	   9,74	   0,37	   10,23	69,10

Orbicella faveolata	          0,00	      0,45	   8,47	   0,94	    8,90	78,00

Groups Punta Cruz  &  Mero

Average dissimilarity = 89,19

	Group Punta Cruz	Group Mero	       	       	        	     

Species	        Av.Abund	  Av.Abund	Av.Diss	Diss/SD	Contrib%	Cum.%

Montastrea cavernosa	            1,66	      0,00	  19,27	   1,40	   21,60	21,60

Dichocoenia stokesii	            1,29	      0,00	  14,29	   1,24	   16,02	37,62

Colpophyllia natans	            0,85	      1,06	  11,34	   1,15	   12,72	50,33

Porites astreoides	            0,00	      1,14	  10,25	   1,39	   11,49	61,82

Pseudodiploria strigosa	            1,07	      0,17	  10,13	   0,86	   11,36	73,18

Siderastrea siderea	            0,49	      0,62	   8,72	   0,72	    9,78	82,96

Groups Gabarra  &  Mero

Average dissimilarity = 83,48

	Group Gabarra	Group Mero	       	       	        	     

Species	     Av.Abund	  Av.Abund	Av.Diss	Diss/SD	Contrib%	Cum.%

Pseudodiploria strigosa	         1,57	      0,17	  24,83	   1,01	   29,74	29,74

Colpophyllia natans	         1,17	      1,06	  15,74	   1,19	   18,86	48,60

Porites astreoides	         0,17	      1,14	  12,37	   1,24	   14,82	63,43

Siderastrea siderea	         0,76	      0,62	   9,97	   0,94	   11,94	75,36

Groups Garrapata  &  Mero

Average dissimilarity = 79,19

	Group Garrapata	Group Mero	       	       	        	     

Species	       Av.Abund	  Av.Abund	Av.Diss	Diss/SD	Contrib%	Cum.%

Pseudodiploria strigosa	           3,23	      0,17	  20,33	   3,03	   25,67	25,67

Colpophyllia natans	           3,62	      1,06	  19,04	   1,59	   24,04	49,71

Montastrea cavernosa	           2,71	      0,00	  17,75	   3,67	   22,42	72,13

Porites astreoides	           0,62	      1,14	   5,21	   1,47	    6,57	78,70

Groups Boca Seca  &  Mero

Average dissimilarity = 75,96

	Group Boca Seca	Group Mero	       	       	        	     

Species	       Av.Abund	  Av.Abund	Av.Diss	Diss/SD	Contrib%	Cum.%

Orbicella faveolata	           0,90	      0,45	  13,66	   0,80	   17,98	17,98

Porites astreoides	           0,75	      1,14	  12,87	   1,18	   16,94	34,92

Colpophyllia natans	           0,49	      1,06	  11,74	   0,96	   15,46	50,38

Agaricia humilis	           0,52	      0,17	   9,08	   0,77	   11,95	62,33

Porites porites	           0,17	      0,55	   6,86	   0,88	    9,03	71,37

Siderastrea siderea	           0,00	      0,62	   5,54	   0,93	    7,29	78,65

Groups Bajo Caiman  &  Mero

Average dissimilarity = 76,51

	Group Bajo Caiman	Group Mero	       	       	        	     

Species	         Av.Abund	  Av.Abund	Av.Diss	Diss/SD	Contrib%	Cum.%

Orbicella faveolata	             1,12	      0,45	  16,90	   0,69	   22,10	22,10

Porites astreoides	             1,01	      1,14	  16,86	   0,94	   22,03	44,13

Colpophyllia natans	             0,45	      1,06	  13,19	   0,86	   17,24	61,37

Porites porites	             0,00	      0,55	   6,79	   0,80	    8,88	70,25

Orbicella annularis	             0,45	      0,00	   6,31	   0,79	    8,25	78,50

Groups Medio  &  Mero

Average dissimilarity = 89,81

	Group Medio	Group Mero	       	       	        	     

Species	   Av.Abund	  Av.Abund	Av.Diss	Diss/SD	Contrib%	Cum.%

Orbicella faveolata	       4,49	      0,45	  33,49	   1,93	   37,29	37,29

Agaricia sp.	       1,23	      0,00	   8,58	   1,28	    9,56	46,84

Porites astreoides	       0,00	      1,14	   7,63	   1,38	    8,50	55,34

Colpophyllia natans	       0,35	      1,06	   6,75	   1,01	    7,51	62,85

Madracis sp.	       1,01	      0,00	   5,79	   0,54	    6,44	69,29

Agaricia agaricites (danai)	       0,75	      0,00	   4,66	   0,80	    5,19	74,48

Agaricia tenuifolia	       0,58	      0,00	   4,50	   0,53	    5,01	79,49

Groups La Pared  &  Norte

Average dissimilarity = 96,70

	Group La Pared	Group Norte	       	       	        	     

Species	      Av.Abund	   Av.Abund	Av.Diss	Diss/SD	Contrib%	Cum.%

Orbicella faveolata	          0,00	       5,10	  22,20	   7,14	   22,95	22,95

Pseudodiploria strigosa	          2,33	       0,00	  10,15	   2,93	   10,50	33,45

Meandrina meandrites	          1,94	       0,00	   8,11	   1,55	    8,38	41,84

Siderastrea siderea	          1,37	       0,00	   5,97	   5,26	    6,18	48,01

Agaricia agaricites (danai)	          0,00	       1,35	   5,80	   1,29	    6,00	54,01

Montastrea cavernosa	          1,38	       0,00	   5,75	   1,55	    5,95	59,96

Colpophyllia natans	          0,00	       1,30	   5,49	   2,36	    5,67	65,64

Acropora palmata	          0,00	       1,19	   5,32	   0,94	    5,51	71,14

Orbicella sp.	          0,00	       1,16	   4,85	   0,91	    5,01	76,15

Groups Media Legua  &  Norte

Average dissimilarity = 94,37

	Group Media Legua	Group Norte	       	       	        	     

Species	         Av.Abund	   Av.Abund	Av.Diss	Diss/SD	Contrib%	Cum.%

Orbicella faveolata	             0,17	       5,10	  27,62	   6,21	   29,26	29,26

Pseudodiploria strigosa	             2,20	       0,00	  12,23	   4,11	   12,96	42,23

Agaricia agaricites (danai)	             0,00	       1,35	   7,43	   1,31	    7,88	50,10

Acropora palmata	             0,00	       1,19	   6,91	   0,94	    7,32	57,42

Orbicella sp.	             0,00	       1,16	   6,16	   0,92	    6,52	63,95

Colpophyllia natans	             0,17	       1,30	   6,05	   1,79	    6,41	70,35

Siderastrea siderea	             0,85	       0,00	   4,71	   1,43	    4,99	75,35

Groups Punta de Media Legua  &  Norte

Average dissimilarity = 98,28

	Group Punta de Media Legua	Group Norte	       	       	        	     

Species	                  Av.Abund	   Av.Abund	Av.Diss	Diss/SD	Contrib%	Cum.%

Orbicella faveolata	                      0,00	       5,10	  27,66	   3,85	   28,15	28,15

Pseudodiploria strigosa	                      1,63	       0,00	   7,88	   1,63	    8,02	36,17

Siderastrea siderea	                      1,59	       0,00	   7,56	   2,28	    7,69	43,86

Agaricia agaricites (danai)	                      0,00	       1,35	   7,20	   1,24	    7,32	51,18

Acropora palmata	                      0,00	       1,19	   6,69	   0,90	    6,81	57,98

Colpophyllia natans	                      0,17	       1,30	   6,08	   1,64	    6,19	64,17

Orbicella sp.	                      0,00	       1,16	   5,96	   0,89	    6,06	70,23

Montastrea cavernosa	                      1,14	       0,00	   5,64	   1,43	    5,74	75,97

Groups Petaquire  &  Norte

Average dissimilarity = 93,82

	Group Petaquire	Group Norte	       	       	        	     

Species	       Av.Abund	   Av.Abund	Av.Diss	Diss/SD	Contrib%	Cum.%

Orbicella faveolata	           0,23	       5,10	  27,16	   4,48	   28,95	28,95

Pseudodiploria strigosa	           2,75	       0,00	  14,97	   5,95	   15,95	44,90

Agaricia agaricites (danai)	           0,00	       1,35	   7,36	   1,28	    7,85	52,75

Acropora palmata	           0,00	       1,19	   6,84	   0,92	    7,29	60,04

Orbicella sp.	           0,00	       1,16	   6,10	   0,90	    6,50	66,54

Colpophyllia natans	           0,23	       1,30	   5,80	   1,57	    6,18	72,73

Siderastrea siderea	           0,92	       0,00	   4,97	   3,36	    5,30	78,02

Groups Playa Tiburon  &  Norte

Average dissimilarity = 93,91

	Group Playa Tiburon	Group Norte	       	       	        	     

Species	           Av.Abund	   Av.Abund	Av.Diss	Diss/SD	Contrib%	Cum.%

Orbicella faveolata	               0,00	       5,10	  24,36	   6,75	   25,94	25,94

Pseudodiploria strigosa	               2,38	       0,00	  11,27	   4,13	   12,00	37,94

Siderastrea siderea	               1,64	       0,00	   7,78	   2,46	    8,29	46,23

Diploria labyrinthiformis	               1,38	       0,00	   6,46	   2,77	    6,88	53,11

Agaricia agaricites (danai)	               0,00	       1,35	   6,36	   1,29	    6,77	59,88

Colpophyllia natans	               0,00	       1,30	   6,01	   2,39	    6,39	66,27

Acropora palmata	               0,00	       1,19	   5,86	   0,94	    6,24	72,51

Orbicella sp.	               0,00	       1,16	   5,29	   0,91	    5,64	78,15

Groups Punta Mono  &  Norte

Average dissimilarity = 99,64

	Group Punta Mono	Group Norte	       	       	        	     

Species	        Av.Abund	   Av.Abund	Av.Diss	Diss/SD	Contrib%	Cum.%

Orbicella faveolata	            0,00	       5,10	  24,93	   6,08	   25,02	25,02

Pseudodiploria strigosa	            2,96	       0,00	  14,12	   6,14	   14,17	39,20

Agaricia agaricites (danai)	            0,00	       1,35	   6,50	   1,29	    6,53	45,72

Siderastrea siderea	            1,34	       0,00	   6,46	   3,43	    6,49	52,21

Colpophyllia natans	            0,00	       1,30	   6,14	   2,37	    6,16	58,37

Acropora palmata	            0,00	       1,19	   6,00	   0,93	    6,02	64,39

Meandrina meandrites	            1,21	       0,00	   5,91	   1,59	    5,93	70,32

Orbicella sp.	            0,00	       1,16	   5,41	   0,91	    5,43	75,75

Groups Charagato  &  Norte

Average dissimilarity = 88,99

	Group Charagato	Group Norte	       	       	        	     

Species	       Av.Abund	   Av.Abund	Av.Diss	Diss/SD	Contrib%	Cum.%

Orbicella faveolata	           0,00	       5,10	  34,14	   4,63	   38,37	38,37

Agaricia agaricites (danai)	           0,00	       1,35	   8,84	   1,29	    9,94	48,30

Colpophyllia natans	           1,86	       1,30	   8,47	   1,68	    9,52	57,82

Acropora palmata	           0,00	       1,19	   8,32	   0,91	    9,35	67,17

Orbicella sp.	           0,00	       1,16	   7,26	   0,91	    8,16	75,33

Groups Punta Conejo  &  Norte

Average dissimilarity = 99,56

	Group Punta Conejo	Group Norte	       	       	        	     

Species	          Av.Abund	   Av.Abund	Av.Diss	Diss/SD	Contrib%	Cum.%

Orbicella faveolata	              0,00	       5,10	  33,96	   5,04	   34,11	34,11

Pseudodiploria strigosa	              1,99	       0,00	  12,03	   1,35	   12,08	46,19

Agaricia agaricites (danai)	              0,00	       1,35	   8,80	   1,30	    8,84	55,03

Acropora palmata	              0,00	       1,19	   8,27	   0,92	    8,30	63,33

Colpophyllia natans	              0,00	       1,30	   8,26	   2,49	    8,30	71,63

Orbicella sp.	              0,00	       1,16	   7,23	   0,91	    7,26	78,90

Groups La Muerta  &  Norte

Average dissimilarity = 92,49

	Group La Muerta	Group Norte	       	       	        	     

Species	       Av.Abund	   Av.Abund	Av.Diss	Diss/SD	Contrib%	Cum.%

Orbicella faveolata	           0,35	       5,10	  30,91	   3,21	   33,42	33,42

Pseudodiploria strigosa	           1,99	       0,00	  11,46	   1,62	   12,39	45,81

Agaricia agaricites (danai)	           0,00	       1,35	   8,48	   1,28	    9,16	54,97

Acropora palmata	           0,00	       1,19	   7,95	   0,91	    8,60	63,57

Colpophyllia natans	           0,62	       1,30	   7,93	   2,18	    8,58	72,15

Orbicella sp.	           0,00	       1,16	   6,97	   0,90	    7,54	79,69

Groups Cominoto  &  Norte

Average dissimilarity = 99,06

	Group Cominoto	Group Norte	       	       	        	     

Species	      Av.Abund	   Av.Abund	Av.Diss	Diss/SD	Contrib%	Cum.%

Orbicella faveolata	          0,00	       5,10	  31,67	   6,94	   31,97	31,97

Pseudodiploria strigosa	          2,96	       0,00	  18,36	   4,63	   18,54	50,51

Agaricia agaricites (danai)	          0,00	       1,35	   8,22	   1,32	    8,30	58,81

Colpophyllia natans	          0,00	       1,30	   7,73	   2,56	    7,81	66,61

Acropora palmata	          0,00	       1,19	   7,68	   0,94	    7,76	74,37

Orbicella sp.	          0,00	       1,16	   6,78	   0,92	    6,85	81,22

Groups La Pecha  &  Norte

Average dissimilarity = 100,00

	Group La Pecha	Group Norte	       	       	        	     

Species	      Av.Abund	   Av.Abund	Av.Diss	Diss/SD	Contrib%	Cum.%

Orbicella faveolata	          0,00	       5,10	  40,19	   5,83	   40,19	40,19

Agaricia agaricites (danai)	          0,00	       1,35	  10,37	   1,34	   10,37	50,56

Acropora palmata	          0,00	       1,19	   9,86	   0,93	    9,86	60,42

Colpophyllia natans	          0,00	       1,30	   9,70	   2,72	    9,70	70,12

Orbicella sp.	          0,00	       1,16	   8,46	   0,93	    8,46	78,57

Groups Puerto Real  &  Norte

Average dissimilarity = 96,85

	Group Puerto Real	Group Norte	       	       	        	     

Species	         Av.Abund	   Av.Abund	Av.Diss	Diss/SD	Contrib%	Cum.%

Orbicella faveolata	             0,00	       5,10	  29,94	   6,84	   30,91	30,91

Pseudodiploria strigosa	             2,60	       0,00	  14,97	   2,85	   15,46	46,37

Agaricia agaricites (danai)	             0,00	       1,35	   7,78	   1,31	    8,03	54,40

Colpophyllia natans	             0,00	       1,30	   7,33	   2,51	    7,56	61,97

Acropora palmata	             0,00	       1,19	   7,25	   0,94	    7,48	69,45

Orbicella sp.	             0,00	       1,16	   6,43	   0,92	    6,64	76,09

Groups Cayo Agua  &  Norte

Average dissimilarity = 59,62

	Group Cayo Agua	Group Norte	       	       	        	     

Species	       Av.Abund	   Av.Abund	Av.Diss	Diss/SD	Contrib%	Cum.%

Madracis mirabilis	           3,47	       0,00	  12,58	   4,26	   21,10	21,10

Orbicella annularis	           3,05	       0,87	   8,48	   1,95	   14,22	35,32

Orbicella faveolata	           3,24	       5,10	   6,75	   4,42	   11,32	46,64

Agaricia agaricites (danai)	           0,00	       1,35	   4,88	   1,29	    8,18	54,82

Acropora palmata	           0,17	       1,19	   4,43	   1,04	    7,44	62,26

Orbicella sp.	           0,68	       1,16	   4,14	   1,16	    6,95	69,20

Madracis decactis	           1,10	       0,00	   3,87	   0,96	    6,50	75,70

Groups Boca de Cote  &  Norte

Average dissimilarity = 67,72

	Group Boca de Cote	Group Norte	       	       	        	     

Species	          Av.Abund	   Av.Abund	Av.Diss	Diss/SD	Contrib%	Cum.%

Siderastrea siderea	              2,54	       0,00	   9,00	   4,84	   13,29	13,29

Orbicella faveolata	              3,10	       5,10	   7,19	   2,26	   10,62	23,91

Porites astreoides	              1,92	       0,17	   6,29	   3,13	    9,29	33,20

Montastrea cavernosa	              1,76	       0,00	   6,18	   2,33	    9,12	42,32

Diploria labyrinthiformis	              1,37	       0,00	   4,96	   2,77	    7,32	49,64

Agaricia agaricites (danai)	              0,00	       1,35	   4,76	   1,28	    7,03	56,67

Orbicella annularis	              1,35	       0,87	   4,47	   1,57	    6,60	63,27

Acropora palmata	              0,00	       1,19	   4,34	   0,94	    6,41	69,67

Orbicella sp.	              0,27	       1,16	   4,01	   1,00	    5,93	75,60

Groups Dos Mosquises Sur  &  Norte

Average dissimilarity = 59,48

	Group Dos Mosquises Sur	Group Norte	       	       	        	     

Species	               Av.Abund	   Av.Abund	Av.Diss	Diss/SD	Contrib%	Cum.%

Madracis mirabilis	                   2,55	       0,00	   9,29	   1,60	   15,61	15,61

Orbicella annularis	                   2,43	       0,87	   7,75	   1,48	   13,03	28,65

Orbicella faveolata	                   3,41	       5,10	   6,35	   2,24	   10,68	39,33

Agaricia agaricites (danai)	                   0,00	       1,35	   5,10	   1,28	    8,58	47,90

Colpophyllia natans	                   2,54	       1,30	   4,99	   1,61	    8,39	56,29

Acropora palmata	                   0,00	       1,19	   4,66	   0,94	    7,83	64,12

Orbicella sp.	                   0,00	       1,16	   4,28	   0,90	    7,19	71,31

Agaricia sp.	                   0,00	       0,85	   3,16	   1,39	    5,31	76,62

Groups Madrisqui  &  Norte

Average dissimilarity = 62,11

	Group Madrisqui	Group Norte	       	       	        	     

Species	       Av.Abund	   Av.Abund	Av.Diss	Diss/SD	Contrib%	Cum.%

Orbicella faveolata	           2,67	       5,10	  11,00	   3,26	   17,71	17,71

Colpophyllia natans	           1,96	       1,30	   5,92	   2,03	    9,53	27,24

Porites astreoides	           1,41	       0,17	   5,84	   1,58	    9,40	36,64

Agaricia agaricites (danai)	           0,35	       1,35	   5,58	   1,37	    8,98	45,62

Acropora palmata	           0,00	       1,19	   5,50	   0,94	    8,85	54,47

Orbicella sp.	           0,00	       1,16	   4,99	   0,91	    8,04	62,51

Agaricia sp.	           0,93	       0,85	   4,74	   1,30	    7,63	70,13

Orbicella annularis	           0,00	       0,87	   3,79	   0,76	    6,10	76,23

Groups Pelona de Rabusqui  &  Norte

Average dissimilarity = 37,81

	Group Pelona de Rabusqui	Group Norte	       	       	        	     

Species	                Av.Abund	   Av.Abund	Av.Diss	Diss/SD	Contrib%	Cum.%

Orbicella annularis	                    2,00	       0,87	   7,02	   1,48	   18,56	18,56

Agaricia agaricites (danai)	                    0,00	       1,35	   5,53	   1,28	   14,62	33,18

Orbicella sp.	                    0,74	       1,16	   5,31	   1,02	   14,05	47,23

Acropora palmata	                    0,00	       1,19	   5,06	   0,94	   13,39	60,62

Colpophyllia natans	                    1,87	       1,30	   4,00	   1,39	   10,58	71,21

Agaricia sp.	                    1,10	       0,85	   3,14	   1,17	    8,30	79,51

Groups Salinas  &  Norte

Average dissimilarity = 75,36

	Group Salinas	Group Norte	       	       	        	     

Species	     Av.Abund	   Av.Abund	Av.Diss	Diss/SD	Contrib%	Cum.%

Orbicella faveolata	         2,24	       5,10	  12,07	   2,75	   16,02	16,02

Siderastrea siderea	         2,20	       0,00	   9,26	   2,57	   12,29	28,31

Montastrea cavernosa	         1,66	       0,00	   6,62	   1,66	    8,78	37,10

Diploria labyrinthiformis	         1,40	       0,00	   5,68	   1,54	    7,54	44,64

Agaricia agaricites (danai)	         0,00	       1,35	   5,65	   1,28	    7,49	52,13

Pseudodiploria strigosa	         1,32	       0,00	   5,31	   1,52	    7,05	59,18

Acropora palmata	         0,00	       1,19	   5,18	   0,94	    6,87	66,05

Orbicella sp.	         0,00	       1,16	   4,72	   0,90	    6,26	72,32

Colpophyllia natans	         0,27	       1,30	   4,29	   1,63	    5,69	78,01

Groups La Venada  &  Norte

Average dissimilarity = 96,64

	Group La Venada	Group Norte	       	       	        	     

Species	       Av.Abund	   Av.Abund	Av.Diss	Diss/SD	Contrib%	Cum.%

Orbicella faveolata	           0,00	       5,10	  28,19	   6,96	   29,17	29,17

Acropora cervicornis	           4,54	       0,00	  25,00	   8,73	   25,87	55,03

Agaricia agaricites (danai)	           0,00	       1,35	   7,34	   1,31	    7,59	62,62

Colpophyllia natans	           0,00	       1,30	   6,91	   2,48	    7,15	69,78

Acropora palmata	           0,00	       1,19	   6,81	   0,94	    7,05	76,82

Groups San Agustin  &  Norte

Average dissimilarity = 100,00

	Group San Agustin	Group Norte	       	       	        	     

Species	         Av.Abund	   Av.Abund	Av.Diss	Diss/SD	Contrib%	Cum.%

Orbicella faveolata	             0,00	       5,10	  40,19	   5,83	   40,19	40,19

Agaricia agaricites (danai)	             0,00	       1,35	  10,37	   1,34	   10,37	50,56

Acropora palmata	             0,00	       1,19	   9,86	   0,93	    9,86	60,42

Colpophyllia natans	             0,00	       1,30	   9,70	   2,72	    9,70	70,12

Orbicella sp.	             0,00	       1,16	   8,46	   0,93	    8,46	78,57

Groups Blanca  &  Norte

Average dissimilarity = 88,86

	Group Blanca	Group Norte	       	       	        	     

Species	    Av.Abund	   Av.Abund	Av.Diss	Diss/SD	Contrib%	Cum.%

Orbicella faveolata	        0,00	       5,10	  20,54	   6,87	   23,12	23,12

Pseudodiploria strigosa	        2,94	       0,00	  11,84	   5,80	   13,32	36,44

Colpophyllia natans	        3,84	       1,30	  10,38	   3,13	   11,68	48,12

Montastrea cavernosa	        1,70	       0,00	   6,87	   3,97	    7,74	55,86

Agaricia agaricites (danai)	        0,00	       1,35	   5,37	   1,28	    6,05	61,91

Acropora palmata	        0,00	       1,19	   4,92	   0,94	    5,53	67,44

Dichocoenia stokesii	        1,17	       0,00	   4,50	   1,58	    5,07	72,51

Orbicella sp.	        0,00	       1,16	   4,50	   0,90	    5,06	77,58

Groups Carabela  &  Norte

Average dissimilarity = 95,91

	Group Carabela	Group Norte	       	       	        	     

Species	      Av.Abund	   Av.Abund	Av.Diss	Diss/SD	Contrib%	Cum.%

Orbicella faveolata	          0,00	       5,10	  38,39	   5,45	   40,03	40,03

Agaricia agaricites (danai)	          0,00	       1,35	   9,92	   1,33	   10,34	50,37

Colpophyllia natans	          0,66	       1,30	   9,53	   2,44	    9,94	60,31

Acropora palmata	          0,00	       1,19	   9,40	   0,92	    9,80	70,10

Orbicella sp.	          0,00	       1,16	   8,10	   0,92	    8,45	78,55

Groups Punta Cruz  &  Norte

Average dissimilarity = 90,63

	Group Punta Cruz	Group Norte	       	       	        	     

Species	        Av.Abund	   Av.Abund	Av.Diss	Diss/SD	Contrib%	Cum.%

Orbicella faveolata	            0,17	       5,10	  27,40	   5,60	   30,24	30,24

Montastrea cavernosa	            1,66	       0,00	   9,15	   2,18	   10,10	40,33

Agaricia agaricites (danai)	            0,00	       1,35	   7,37	   1,30	    8,13	48,47

Dichocoenia stokesii	            1,29	       0,00	   6,98	   1,61	    7,71	56,17

Acropora palmata	            0,00	       1,19	   6,85	   0,93	    7,55	63,72

Orbicella sp.	            0,00	       1,16	   6,11	   0,91	    6,74	70,46

Pseudodiploria strigosa	            1,07	       0,00	   5,40	   0,88	    5,96	76,42

Groups Gabarra  &  Norte

Average dissimilarity = 90,85

	Group Gabarra	Group Norte	       	       	        	     

Species	     Av.Abund	   Av.Abund	Av.Diss	Diss/SD	Contrib%	Cum.%

Orbicella faveolata	         0,00	       5,10	  31,53	   5,01	   34,71	34,71

Pseudodiploria strigosa	         1,57	       0,00	   9,32	   2,32	   10,26	44,97

Agaricia agaricites (danai)	         0,00	       1,35	   8,18	   1,29	    9,01	53,98

Acropora palmata	         0,00	       1,19	   7,65	   0,92	    8,42	62,40

Orbicella sp.	         0,00	       1,16	   6,75	   0,91	    7,43	69,83

Colpophyllia natans	         1,17	       1,30	   5,64	   1,46	    6,21	76,04

Groups Garrapata  &  Norte

Average dissimilarity = 85,61

	Group Garrapata	Group Norte	       	       	        	     

Species	       Av.Abund	   Av.Abund	Av.Diss	Diss/SD	Contrib%	Cum.%

Orbicella faveolata	           0,35	       5,10	  19,79	   5,81	   23,11	23,11

Pseudodiploria strigosa	           3,23	       0,00	  13,41	   7,58	   15,66	38,77

Montastrea cavernosa	           2,71	       0,00	  11,26	   7,60	   13,15	51,92

Colpophyllia natans	           3,62	       1,30	   9,81	   2,16	   11,46	63,38

Agaricia agaricites (danai)	           0,00	       1,35	   5,55	   1,30	    6,49	69,87

Acropora palmata	           0,00	       1,19	   5,08	   0,95	    5,94	75,81

Groups Boca Seca  &  Norte

Average dissimilarity = 76,57

	Group Boca Seca	Group Norte	       	       	        	     

Species	       Av.Abund	   Av.Abund	Av.Diss	Diss/SD	Contrib%	Cum.%

Orbicella faveolata	           0,90	       5,10	  26,56	   3,01	   34,68	34,68

Agaricia agaricites (danai)	           0,00	       1,35	   8,27	   1,30	   10,80	45,48

Acropora palmata	           0,00	       1,19	   7,74	   0,93	   10,10	55,58

Colpophyllia natans	           0,49	       1,30	   7,21	   2,17	    9,41	64,99

Orbicella sp.	           0,00	       1,16	   6,82	   0,91	    8,90	73,89

Orbicella annularis	           0,00	       0,87	   5,21	   0,77	    6,81	80,70

Groups Bajo Caiman  &  Norte

Average dissimilarity = 78,87

	Group Bajo Caiman	Group Norte	       	       	        	     

Species	         Av.Abund	   Av.Abund	Av.Diss	Diss/SD	Contrib%	Cum.%

Orbicella faveolata	             1,12	       5,10	  25,51	   4,65	   32,34	32,34

Agaricia agaricites (danai)	             0,00	       1,35	   8,46	   1,31	   10,72	43,06

Acropora palmata	             0,00	       1,19	   7,92	   0,93	   10,04	53,10

Colpophyllia natans	             0,45	       1,30	   7,19	   2,22	    9,12	62,22

Orbicella sp.	             0,00	       1,16	   6,96	   0,92	    8,83	71,05

Porites astreoides	             1,01	       0,17	   6,08	   0,95	    7,71	78,76

Groups Medio  &  Norte

Average dissimilarity = 43,04

	Group Medio	Group Norte	       	       	        	     

Species	   Av.Abund	   Av.Abund	Av.Diss	Diss/SD	Contrib%	Cum.%

Acropora palmata	       0,00	       1,19	   5,63	   0,93	   13,08	13,08

Agaricia agaricites (danai)	       0,75	       1,35	   5,49	   1,23	   12,76	25,84

Orbicella sp.	       0,00	       1,16	   5,10	   0,90	   11,85	37,70

Colpophyllia natans	       0,35	       1,30	   4,96	   1,84	   11,52	49,22

Agaricia sp.	       1,23	       0,85	   4,32	   1,35	   10,04	59,26

Orbicella annularis	       0,00	       0,87	   3,87	   0,76	    9,00	68,26

Madracis sp.	       1,01	       0,00	   3,86	   0,56	    8,96	77,22

Groups Mero  &  Norte

Average dissimilarity = 86,84

	Group Mero	Group Norte	       	       	        	     

Species	  Av.Abund	   Av.Abund	Av.Diss	Diss/SD	Contrib%	Cum.%

Orbicella faveolata	      0,45	       5,10	  28,61	   2,93	   32,94	32,94

Agaricia agaricites (danai)	      0,00	       1,35	   8,02	   1,24	    9,23	42,18

Acropora palmata	      0,00	       1,19	   7,51	   0,89	    8,64	50,82

Colpophyllia natans	      1,06	       1,30	   7,00	   1,62	    8,07	58,89

Orbicella sp.	      0,00	       1,16	   6,61	   0,89	    7,61	66,50

Porites astreoides	      1,14	       0,17	   5,77	   1,43	    6,64	73,14

Orbicella annularis	      0,00	       0,87	   5,05	   0,75	    5,82	78,96

Groups La Pared  &  Sombrero

Average dissimilarity = 80,08

	Group La Pared	Group Sombrero	       	       	        	     

Species	      Av.Abund	      Av.Abund	Av.Diss	Diss/SD	Contrib%	Cum.%

Colpophyllia natans	          0,00	          3,81	  15,51	   8,01	   19,37	19,37

Orbicella faveolata	          0,00	          3,55	  14,41	   7,31	   18,00	37,37

Meandrina meandrites	          1,94	          0,00	   7,62	   1,56	    9,51	46,88

Pseudodiploria strigosa	          2,33	          1,17	   5,55	   1,33	    6,93	53,81

Porites astreoides	          0,45	          1,72	   5,28	   2,12	    6,59	60,40

Siderastrea siderea	          1,37	          0,17	   4,88	   3,12	    6,09	66,49

Montastrea cavernosa	          1,38	          0,85	   4,18	   1,23	    5,21	71,71

Diploria labyrinthiformis	          0,72	          0,00	   2,98	   0,94	    3,72	75,43

Groups Media Legua  &  Sombrero

Average dissimilarity = 79,18

	Group Media Legua	Group Sombrero	       	       	        	     

Species	         Av.Abund	      Av.Abund	Av.Diss	Diss/SD	Contrib%	Cum.%

Colpophyllia natans	             0,17	          3,81	  18,70	   6,70	   23,62	23,62

Orbicella faveolata	             0,17	          3,55	  17,31	   6,12	   21,86	45,48

Porites astreoides	             0,17	          1,72	   8,05	   3,32	   10,17	55,65

Pseudodiploria strigosa	             2,20	          1,17	   6,30	   1,39	    7,96	63,61

Montastrea cavernosa	             0,45	          0,85	   4,38	   1,28	    5,53	69,14

Siderastrea siderea	             0,85	          0,17	   3,88	   1,36	    4,90	74,04

Agaricia agaricites (danai)	             0,00	          0,69	   3,48	   0,82	    4,40	78,43

Groups Punta de Media Legua  &  Sombrero

Average dissimilarity = 81,81

	Group Punta de Media Legua	Group Sombrero	       	       	        	     

Species	                  Av.Abund	      Av.Abund	Av.Diss	Diss/SD	Contrib%	Cum.%

Colpophyllia natans	                      0,17	          3,81	  18,28	   3,58	   22,35	22,35

Orbicella faveolata	                      0,00	          3,55	  17,60	   4,30	   21,51	43,86

Porites astreoides	                      0,27	          1,72	   7,55	   2,06	    9,23	53,09

Siderastrea siderea	                      1,59	          0,17	   6,14	   1,68	    7,51	60,60

Pseudodiploria strigosa	                      1,63	          1,17	   5,67	   1,39	    6,93	67,52

Montastrea cavernosa	                      1,14	          0,85	   4,62	   1,22	    5,64	73,17

Agaricia agaricites (danai)	                      0,00	          0,69	   3,37	   0,80	    4,12	77,28

Groups Petaquire  &  Sombrero

Average dissimilarity = 80,03

	Group Petaquire	Group Sombrero	       	       	        	     

Species	       Av.Abund	      Av.Abund	Av.Diss	Diss/SD	Contrib%	Cum.%

Colpophyllia natans	           0,23	          3,81	  18,34	   4,64	   22,91	22,91

Orbicella faveolata	           0,23	          3,55	  16,96	   4,37	   21,19	44,11

Porites astreoides	           0,00	          1,72	   8,79	   4,73	   10,99	55,10

Pseudodiploria strigosa	           2,75	          1,17	   8,27	   1,59	   10,33	65,43

Montastrea cavernosa	           0,23	          0,85	   4,35	   1,12	    5,44	70,86

Siderastrea siderea	           0,92	          0,17	   3,69	   1,74	    4,61	75,47

Groups Playa Tiburon  &  Sombrero

Average dissimilarity = 79,31

	Group Playa Tiburon	Group Sombrero	       	       	        	     

Species	           Av.Abund	      Av.Abund	Av.Diss	Diss/SD	Contrib%	Cum.%

Colpophyllia natans	               0,00	          3,81	  16,91	   7,80	   21,32	21,32

Orbicella faveolata	               0,00	          3,55	  15,71	   7,18	   19,81	41,13

Siderastrea siderea	               1,64	          0,17	   6,47	   2,01	    8,16	49,29

Pseudodiploria strigosa	               2,38	          1,17	   6,13	   1,53	    7,73	57,02

Porites astreoides	               0,40	          1,72	   6,11	   1,82	    7,70	64,73

Diploria labyrinthiformis	               1,38	          0,00	   6,02	   2,81	    7,59	72,32

Montastrea cavernosa	               0,93	          0,85	   4,02	   1,44	    5,07	77,39

Groups Punta Mono  &  Sombrero

Average dissimilarity = 85,52

	Group Punta Mono	Group Sombrero	       	       	        	     

Species	        Av.Abund	      Av.Abund	Av.Diss	Diss/SD	Contrib%	Cum.%

Colpophyllia natans	            0,00	          3,81	  17,27	   7,03	   20,19	20,19

Orbicella faveolata	            0,00	          3,55	  16,05	   6,57	   18,76	38,96

Pseudodiploria strigosa	            2,96	          1,17	   8,34	   1,86	    9,75	48,70

Porites astreoides	            0,17	          1,72	   7,10	   3,18	    8,30	57,01

Meandrina meandrites	            1,21	          0,00	   5,49	   1,60	    6,41	63,42

Siderastrea siderea	            1,34	          0,17	   5,21	   2,35	    6,10	69,52

Diploria labyrinthiformis	            1,02	          0,00	   4,55	   3,03	    5,32	74,84

Montastrea cavernosa	            0,27	          0,85	   3,87	   1,06	    4,53	79,37

Groups Charagato  &  Sombrero

Average dissimilarity = 77,05

	Group Charagato	Group Sombrero	       	       	        	     

Species	       Av.Abund	      Av.Abund	Av.Diss	Diss/SD	Contrib%	Cum.%

Orbicella faveolata	           0,00	          3,55	  21,31	   5,58	   27,66	27,66

Colpophyllia natans	           1,86	          3,81	  12,86	   1,28	   16,69	44,35

Porites astreoides	           0,00	          1,72	  10,42	   4,33	   13,52	57,87

Pseudodiploria strigosa	           0,40	          1,17	   6,45	   1,28	    8,38	66,25

Montastrea cavernosa	           0,00	          0,85	   5,18	   0,95	    6,72	72,96

Agaricia agaricites (danai)	           0,00	          0,69	   4,06	   0,81	    5,27	78,24

Groups Punta Conejo  &  Sombrero

Average dissimilarity = 88,64

	Group Punta Conejo	Group Sombrero	       	       	        	     

Species	          Av.Abund	      Av.Abund	Av.Diss	Diss/SD	Contrib%	Cum.%

Colpophyllia natans	              0,00	          3,81	  22,85	   6,40	   25,78	25,78

Orbicella faveolata	              0,00	          3,55	  21,23	   6,13	   23,95	49,73

Porites astreoides	              0,17	          1,72	   9,47	   2,96	   10,69	60,42

Pseudodiploria strigosa	              1,99	          1,17	   9,05	   1,41	   10,21	70,62

Montastrea cavernosa	              0,00	          0,85	   5,15	   0,96	    5,81	76,44

Groups La Muerta  &  Sombrero

Average dissimilarity = 77,36

	Group La Muerta	Group Sombrero	       	       	        	     

Species	       Av.Abund	      Av.Abund	Av.Diss	Diss/SD	Contrib%	Cum.%

Orbicella faveolata	           0,35	          3,55	  18,84	   3,06	   24,35	24,35

Colpophyllia natans	           0,62	          3,81	  18,83	   2,31	   24,35	48,70

Porites astreoides	           0,52	          1,72	   8,37	   1,84	   10,82	59,52

Pseudodiploria strigosa	           1,99	          1,17	   8,21	   1,55	   10,61	70,13

Montastrea cavernosa	           0,00	          0,85	   4,97	   0,95	    6,43	76,56

Groups Cominoto  &  Sombrero

Average dissimilarity = 81,18

	Group Cominoto	Group Sombrero	       	       	        	     

Species	      Av.Abund	      Av.Abund	Av.Diss	Diss/SD	Contrib%	Cum.%

Colpophyllia natans	          0,00	          3,81	  21,50	   9,26	   26,48	26,48

Orbicella faveolata	          0,00	          3,55	  19,97	   8,41	   24,60	51,08

Pseudodiploria strigosa	          2,96	          1,17	  10,50	   1,66	   12,93	64,01

Porites astreoides	          0,58	          1,72	   6,70	   1,68	    8,25	72,26

Montastrea cavernosa	          0,00	          0,85	   4,84	   0,97	    5,97	78,23

Groups La Pecha  &  Sombrero

Average dissimilarity = 98,32

	Group La Pecha	Group Sombrero	       	       	        	     

Species	      Av.Abund	      Av.Abund	Av.Diss	Diss/SD	Contrib%	Cum.%

Colpophyllia natans	          0,00	          3,81	  26,56	   8,66	   27,01	27,01

Orbicella faveolata	          0,00	          3,55	  24,66	   8,11	   25,08	52,10

Porites astreoides	          0,00	          1,72	  12,06	   5,16	   12,27	64,37

Pseudodiploria strigosa	          0,17	          1,17	   7,24	   1,31	    7,36	71,73

Montastrea cavernosa	          0,00	          0,85	   6,00	   0,97	    6,10	77,83

Groups Puerto Real  &  Sombrero

Average dissimilarity = 75,31

	Group Puerto Real	Group Sombrero	       	       	        	     

Species	         Av.Abund	      Av.Abund	Av.Diss	Diss/SD	Contrib%	Cum.%

Colpophyllia natans	             0,00	          3,81	  20,43	   8,74	   27,12	27,12

Orbicella faveolata	             0,00	          3,55	  18,98	   7,99	   25,20	52,32

Pseudodiploria strigosa	             2,60	          1,17	   8,60	   1,42	   11,41	63,74

Montastrea cavernosa	             0,00	          0,85	   4,60	   0,97	    6,11	69,84

Agaricia agaricites (danai)	             0,00	          0,69	   3,63	   0,82	    4,82	74,66

Porites astreoides	             1,10	          1,72	   3,38	   2,37	    4,49	79,15

Groups Cayo Agua  &  Sombrero

Average dissimilarity = 63,32

	Group Cayo Agua	Group Sombrero	       	       	        	     

Species	       Av.Abund	      Av.Abund	Av.Diss	Diss/SD	Contrib%	Cum.%

Madracis mirabilis	           3,47	          0,00	  11,92	   4,36	   18,82	18,82

Orbicella annularis	           3,05	          0,00	  10,46	   3,57	   16,51	35,33

Colpophyllia natans	           1,15	          3,81	   9,24	   2,64	   14,59	49,92

Porites astreoides	           0,69	          1,72	   4,11	   1,91	    6,50	56,42

Pseudodiploria strigosa	           0,00	          1,17	   3,94	   1,30	    6,23	62,64

Madracis decactis	           1,10	          0,17	   3,69	   1,12	    5,83	68,48

Montastrea cavernosa	           0,17	          0,85	   2,95	   1,13	    4,65	73,13

Orbicella sp.	           0,68	          0,17	   2,36	   1,04	    3,73	76,85

Groups Boca de Cote  &  Sombrero

Average dissimilarity = 58,04

	Group Boca de Cote	Group Sombrero	       	       	        	     

Species	          Av.Abund	      Av.Abund	Av.Diss	Diss/SD	Contrib%	Cum.%

Colpophyllia natans	              0,17	          3,81	  12,33	   5,76	   21,24	21,24

Siderastrea siderea	              2,54	          0,17	   7,95	   4,01	   13,69	34,93

Diploria labyrinthiformis	              1,37	          0,00	   4,69	   2,82	    8,09	43,02

Orbicella annularis	              1,35	          0,00	   4,39	   1,53	    7,57	50,59

Montastrea cavernosa	              1,76	          0,85	   3,94	   1,39	    6,79	57,37

Madracis mirabilis	              1,01	          0,00	   3,18	   0,73	    5,48	62,85

Pseudodiploria strigosa	              0,62	          1,17	   3,15	   1,14	    5,43	68,28

Orbicella faveolata	              3,10	          3,55	   2,54	   1,21	    4,38	72,65

Agaricia agaricites (danai)	              0,00	          0,69	   2,30	   0,81	    3,97	76,62

Groups Dos Mosquises Sur  &  Sombrero

Average dissimilarity = 54,63

	Group Dos Mosquises Sur	Group Sombrero	       	       	        	     

Species	               Av.Abund	      Av.Abund	Av.Diss	Diss/SD	Contrib%	Cum.%

Madracis mirabilis	                   2,55	          0,00	   8,79	   1,61	   16,09	16,09

Orbicella annularis	                   2,43	          0,00	   8,39	   1,49	   15,37	31,46

Porites astreoides	                   0,17	          1,72	   5,66	   3,25	   10,35	41,81

Colpophyllia natans	                   2,54	          3,81	   4,51	   2,73	    8,25	50,07

Pseudodiploria strigosa	                   0,45	          1,17	   3,97	   1,35	    7,27	57,33

Montastrea cavernosa	                   0,00	          0,85	   3,08	   0,96	    5,64	62,97

Agaricia agaricites (danai)	                   0,00	          0,69	   2,46	   0,81	    4,50	67,47

Orbicella faveolata	                   3,41	          3,55	   2,39	   1,29	    4,38	71,85

Agaricia sp.	                   0,00	          0,58	   2,04	   0,85	    3,74	75,59

Groups Madrisqui  &  Sombrero

Average dissimilarity = 45,55

	Group Madrisqui	Group Sombrero	       	       	        	     

Species	       Av.Abund	      Av.Abund	Av.Diss	Diss/SD	Contrib%	Cum.%

Colpophyllia natans	           1,96	          3,81	   8,00	   1,38	   17,56	17,56

Pseudodiploria strigosa	           0,00	          1,17	   4,76	   1,30	   10,45	28,01

Agaricia sp.	           0,93	          0,58	   4,18	   1,04	    9,17	37,18

Montastrea cavernosa	           0,52	          0,85	   4,00	   1,05	    8,78	45,97

Orbicella faveolata	           2,67	          3,55	   3,83	   1,39	    8,40	54,37

Agaricia agaricites (danai)	           0,35	          0,69	   3,24	   1,01	    7,10	61,47

Porites astreoides	           1,41	          1,72	   3,06	   1,05	    6,71	68,18

Eusmilia fastigata	           0,68	          0,27	   2,75	   1,03	    6,03	74,21

Stephanocoenia intersepta	           0,00	          0,58	   2,34	   0,56	    5,14	79,35

Groups Pelona de Rabusqui  &  Sombrero

Average dissimilarity = 52,89

	Group Pelona de Rabusqui	Group Sombrero	       	       	        	     

Species	                Av.Abund	      Av.Abund	Av.Diss	Diss/SD	Contrib%	Cum.%

Colpophyllia natans	                    1,87	          3,81	   7,67	   1,89	   14,50	14,50

Orbicella annularis	                    2,00	          0,00	   7,66	   1,52	   14,48	28,97

Porites astreoides	                    0,00	          1,72	   6,71	   5,25	   12,69	41,67

Orbicella faveolata	                    4,91	          3,55	   5,37	   2,78	   10,16	51,83

Pseudodiploria strigosa	                    0,17	          1,17	   4,14	   1,26	    7,83	59,65

Agaricia sp.	                    1,10	          0,58	   3,38	   1,21	    6,39	66,04

Montastrea cavernosa	                    0,00	          0,85	   3,33	   0,96	    6,29	72,33

Orbicella sp.	                    0,74	          0,17	   2,95	   0,70	    5,57	77,90

Groups Salinas  &  Sombrero

Average dissimilarity = 61,65

	Group Salinas	Group Sombrero	       	       	        	     

Species	     Av.Abund	      Av.Abund	Av.Diss	Diss/SD	Contrib%	Cum.%

Colpophyllia natans	         0,27	          3,81	  13,89	   6,71	   22,53	22,53

Siderastrea siderea	         2,20	          0,17	   8,00	   2,27	   12,97	35,50

Diploria labyrinthiformis	         1,40	          0,00	   5,34	   1,55	    8,67	44,17

Orbicella faveolata	         2,24	          3,55	   5,27	   1,38	    8,55	52,72

Montastrea cavernosa	         1,66	          0,85	   4,76	   1,34	    7,72	60,44

Porites astreoides	         0,68	          1,72	   4,34	   1,48	    7,03	67,48

Pseudodiploria strigosa	         1,32	          1,17	   4,02	   1,34	    6,52	74,00

Agaricia agaricites (danai)	         0,00	          0,69	   2,70	   0,81	    4,38	78,38

Groups La Venada  &  Sombrero

Average dissimilarity = 96,85

	Group La Venada	Group Sombrero	       	       	        	     

Species	       Av.Abund	      Av.Abund	Av.Diss	Diss/SD	Contrib%	Cum.%

Acropora cervicornis	           4,54	          0,00	  23,00	  12,57	   23,74	23,74

Colpophyllia natans	           0,00	          3,81	  19,34	   8,63	   19,97	43,71

Orbicella faveolata	           0,00	          3,55	  17,97	   7,87	   18,55	62,26

Porites astreoides	           0,00	          1,72	   8,77	   5,41	    9,06	71,32

Pseudodiploria strigosa	           0,27	          1,17	   5,14	   1,18	    5,30	76,62

Groups San Agustin  &  Sombrero

Average dissimilarity = 98,82

	Group San Agustin	Group Sombrero	       	       	        	     

Species	         Av.Abund	      Av.Abund	Av.Diss	Diss/SD	Contrib%	Cum.%

Colpophyllia natans	             0,00	          3,81	  26,56	   8,66	   26,88	26,88

Orbicella faveolata	             0,00	          3,55	  24,66	   8,11	   24,96	51,83

Porites astreoides	             0,00	          1,72	  12,06	   5,16	   12,21	64,04

Pseudodiploria strigosa	             0,00	          1,17	   7,75	   1,33	    7,85	71,89

Montastrea cavernosa	             0,17	          0,85	   5,98	   1,09	    6,05	77,94

Groups Blanca  &  Sombrero

Average dissimilarity = 51,24

	Group Blanca	Group Sombrero	       	       	        	     

Species	    Av.Abund	      Av.Abund	Av.Diss	Diss/SD	Contrib%	Cum.%

Orbicella faveolata	        0,00	          3,55	  13,41	   6,86	   26,17	26,17

Pseudodiploria strigosa	        2,94	          1,17	   6,87	   1,74	   13,40	39,56

Dichocoenia stokesii	        1,17	          0,00	   4,25	   1,59	    8,29	47,86

Porites astreoides	        0,62	          1,72	   4,24	   2,19	    8,27	56,13

Montastrea cavernosa	        1,70	          0,85	   3,70	   1,21	    7,22	63,34

Agaricia agaricites (danai)	        0,00	          0,69	   2,58	   0,81	    5,03	68,38

Stephanocoenia intersepta	        0,27	          0,58	   2,58	   0,74	    5,03	73,41

Agaricia sp.	        0,00	          0,58	   2,14	   0,85	    4,18	77,59

Groups Carabela  &  Sombrero

Average dissimilarity = 90,98

	Group Carabela	Group Sombrero	       	       	        	     

Species	      Av.Abund	      Av.Abund	Av.Diss	Diss/SD	Contrib%	Cum.%

Orbicella faveolata	          0,00	          3,55	  23,68	   7,16	   26,03	26,03

Colpophyllia natans	          0,66	          3,81	  21,58	   2,35	   23,72	49,75

Porites astreoides	          0,00	          1,72	  11,58	   4,91	   12,73	62,48

Pseudodiploria strigosa	          0,00	          1,17	   7,46	   1,32	    8,20	70,68

Montastrea cavernosa	          0,17	          0,85	   5,74	   1,10	    6,31	76,99

Groups Punta Cruz  &  Sombrero

Average dissimilarity = 77,53

	Group Punta Cruz	Group Sombrero	       	       	        	     

Species	        Av.Abund	      Av.Abund	Av.Diss	Diss/SD	Contrib%	Cum.%

Orbicella faveolata	            0,17	          3,55	  17,19	   5,54	   22,17	22,17

Colpophyllia natans	            0,85	          3,81	  15,13	   3,66	   19,52	41,69

Porites astreoides	            0,00	          1,72	   8,81	   5,18	   11,36	53,05

Dichocoenia stokesii	            1,29	          0,00	   6,43	   1,63	    8,30	61,34

Pseudodiploria strigosa	            1,07	          1,17	   6,03	   1,35	    7,77	69,11

Montastrea cavernosa	            1,66	          0,85	   5,80	   1,49	    7,49	76,60

Groups Gabarra  &  Sombrero

Average dissimilarity = 75,25

	Group Gabarra	Group Sombrero	       	       	        	     

Species	     Av.Abund	      Av.Abund	Av.Diss	Diss/SD	Contrib%	Cum.%

Orbicella faveolata	         0,00	          3,55	  19,86	   5,95	   26,39	26,39

Colpophyllia natans	         1,17	          3,81	  15,27	   2,11	   20,29	46,68

Porites astreoides	         0,17	          1,72	   8,82	   3,00	   11,72	58,40

Pseudodiploria strigosa	         1,57	          1,17	   5,41	   1,29	    7,19	65,59

Montastrea cavernosa	         0,00	          0,85	   4,82	   0,95	    6,40	71,99

Siderastrea siderea	         0,76	          0,17	   4,00	   1,05	    5,32	77,31

Groups Garrapata  &  Sombrero

Average dissimilarity = 49,13

	Group Garrapata	Group Sombrero	       	       	        	     

Species	       Av.Abund	      Av.Abund	Av.Diss	Diss/SD	Contrib%	Cum.%

Orbicella faveolata	           0,35	          3,55	  12,48	   4,33	   25,40	25,40

Pseudodiploria strigosa	           3,23	          1,17	   8,15	   1,98	   16,58	41,98

Montastrea cavernosa	           2,71	          0,85	   7,24	   2,03	   14,73	56,71

Porites astreoides	           0,62	          1,72	   4,31	   2,26	    8,78	65,49

Colpophyllia natans	           3,62	          3,81	   2,66	   1,28	    5,42	70,91

Agaricia agaricites (danai)	           0,00	          0,69	   2,66	   0,81	    5,41	76,33

Groups Boca Seca  &  Sombrero

Average dissimilarity = 71,55

	Group Boca Seca	Group Sombrero	       	       	        	     

Species	       Av.Abund	      Av.Abund	Av.Diss	Diss/SD	Contrib%	Cum.%

Colpophyllia natans	           0,49	          3,81	  19,05	   3,03	   26,63	26,63

Orbicella faveolata	           0,90	          3,55	  15,21	   2,25	   21,26	47,89

Porites astreoides	           0,75	          1,72	   5,90	   1,52	    8,24	56,13

Pseudodiploria strigosa	           0,45	          1,17	   5,31	   1,15	    7,43	63,56

Montastrea cavernosa	           0,00	          0,85	   4,86	   0,96	    6,80	70,36

Agaricia agaricites (danai)	           0,00	          0,69	   3,83	   0,82	    5,35	75,71

Groups Bajo Caiman  &  Sombrero

Average dissimilarity = 73,49

	Group Bajo Caiman	Group Sombrero	       	       	        	     

Species	         Av.Abund	      Av.Abund	Av.Diss	Diss/SD	Contrib%	Cum.%

Colpophyllia natans	             0,45	          3,81	  19,47	   3,52	   26,50	26,50

Orbicella faveolata	             1,12	          3,55	  14,04	   4,06	   19,10	45,60

Porites astreoides	             1,01	          1,72	   7,04	   1,65	    9,58	55,17

Pseudodiploria strigosa	             0,00	          1,17	   6,49	   1,31	    8,84	64,01

Montastrea cavernosa	             0,00	          0,85	   4,97	   0,96	    6,76	70,77

Agaricia agaricites (danai)	             0,00	          0,69	   3,91	   0,82	    5,32	76,09

Groups Medio  &  Sombrero

Average dissimilarity = 59,43

	Group Medio	Group Sombrero	       	       	        	     

Species	   Av.Abund	      Av.Abund	Av.Diss	Diss/SD	Contrib%	Cum.%

Colpophyllia natans	       0,35	          3,81	  14,82	   4,15	   24,94	24,94

Porites astreoides	       0,00	          1,72	   7,39	   4,88	   12,44	37,38

Orbicella faveolata	       4,49	          3,55	   4,53	   1,69	    7,62	44,99

Pseudodiploria strigosa	       0,17	          1,17	   4,51	   1,28	    7,59	52,58

Agaricia sp.	       1,23	          0,58	   4,36	   1,30	    7,33	59,91

Agaricia agaricites (danai)	       0,75	          0,69	   3,75	   1,06	    6,31	66,22

Montastrea cavernosa	       0,00	          0,85	   3,66	   0,96	    6,16	72,39

Madracis sp.	       1,01	          0,00	   3,64	   0,56	    6,13	78,52

Groups Mero  &  Sombrero

Average dissimilarity = 73,68

	Group Mero	Group Sombrero	       	       	        	     

Species	  Av.Abund	      Av.Abund	Av.Diss	Diss/SD	Contrib%	Cum.%

Orbicella faveolata	      0,45	          3,55	  17,32	   2,89	   23,51	23,51

Colpophyllia natans	      1,06	          3,81	  16,39	   1,68	   22,25	45,76

Pseudodiploria strigosa	      0,17	          1,17	   5,84	   1,20	    7,93	53,69

Porites astreoides	      1,14	          1,72	   4,76	   0,99	    6,46	60,15

Montastrea cavernosa	      0,00	          0,85	   4,72	   0,93	    6,40	66,55

Agaricia agaricites (danai)	      0,00	          0,69	   3,71	   0,80	    5,04	71,59

Agaricia sp.	      0,00	          0,58	   3,08	   0,83	    4,19	75,77

Groups Norte  &  Sombrero

Average dissimilarity = 55,46

	Group Norte	Group Sombrero	       	       	        	     

Species	   Av.Abund	      Av.Abund	Av.Diss	Diss/SD	Contrib%	Cum.%

Colpophyllia natans	       1,30	          3,81	   9,46	   3,08	   17,05	17,05

Porites astreoides	       0,17	          1,72	   5,82	   3,35	   10,50	27,55

Orbicella faveolata	       5,10	          3,55	   5,81	   2,97	   10,48	38,03

Acropora palmata	       1,19	          0,00	   4,53	   0,95	    8,18	46,20

Agaricia agaricites (danai)	       1,35	          0,69	   4,31	   1,26	    7,78	53,98

Pseudodiploria strigosa	       0,00	          1,17	   4,24	   1,30	    7,64	61,62

Orbicella sp.	       1,16	          0,17	   4,18	   1,01	    7,54	69,16

Montastrea cavernosa	       0,00	          0,85	   3,18	   0,96	    5,73	74,90

Orbicella annularis	       0,87	          0,00	   3,16	   0,76	    5,69	80,59

Groups La Pared  &  Sur

Average dissimilarity = 90,37

	Group La Pared	Group Sur	       	       	        	     

Species	      Av.Abund	 Av.Abund	Av.Diss	Diss/SD	Contrib%	Cum.%

Orbicella faveolata	          0,00	     4,60	  22,29	   6,36	   24,67	24,67

Colpophyllia natans	          0,00	     2,70	  12,85	   2,85	   14,22	38,89

Meandrina meandrites	          1,94	     0,00	   8,96	   1,55	    9,91	48,80

Pseudodiploria strigosa	          2,33	     0,62	   8,17	   2,05	    9,04	57,84

Siderastrea siderea	          1,37	     0,00	   6,64	   4,96	    7,34	65,18

Montastrea cavernosa	          1,38	     0,00	   6,36	   1,55	    7,03	72,22

Diploria labyrinthiformis	          0,72	     0,00	   3,55	   0,94	    3,93	76,14

Groups Media Legua  &  Sur

Average dissimilarity = 85,36

	Group Media Legua	Group Sur	       	       	        	     

Species	         Av.Abund	 Av.Abund	Av.Diss	Diss/SD	Contrib%	Cum.%

Orbicella faveolata	             0,17	     4,60	  28,56	   5,38	   33,46	33,46

Colpophyllia natans	             0,17	     2,70	  15,90	   2,54	   18,63	52,09

Pseudodiploria strigosa	             2,20	     0,62	   9,85	   2,70	   11,54	63,63

Siderastrea siderea	             0,85	     0,00	   5,40	   1,43	    6,33	69,96

Meandrina meandrites	             0,60	     0,00	   3,87	   0,55	    4,53	74,49

Agaricia tenuifolia	             0,00	     0,55	   3,31	   0,56	    3,87	78,36

Groups Punta de Media Legua  &  Sur

Average dissimilarity = 90,73

	Group Punta de Media Legua	Group Sur	       	       	        	     

Species	                  Av.Abund	 Av.Abund	Av.Diss	Diss/SD	Contrib%	Cum.%

Orbicella faveolata	                      0,00	     4,60	  28,78	   3,33	   31,72	31,72

Colpophyllia natans	                      0,17	     2,70	  15,72	   2,05	   17,32	49,04

Siderastrea siderea	                      1,59	     0,00	   8,53	   2,41	    9,40	58,45

Pseudodiploria strigosa	                      1,63	     0,62	   7,25	   2,36	    7,99	66,43

Montastrea cavernosa	                      1,14	     0,00	   6,39	   1,41	    7,05	73,48

Diploria labyrinthiformis	                      0,83	     0,00	   3,82	   0,77	    4,21	77,69

Groups Petaquire  &  Sur

Average dissimilarity = 85,43

	Group Petaquire	Group Sur	       	       	        	     

Species	       Av.Abund	 Av.Abund	Av.Diss	Diss/SD	Contrib%	Cum.%

Orbicella faveolata	           0,23	     4,60	  28,11	   3,88	   32,90	32,90

Colpophyllia natans	           0,23	     2,70	  15,56	   2,22	   18,22	51,12

Pseudodiploria strigosa	           2,75	     0,62	  12,99	   4,29	   15,21	66,33

Siderastrea siderea	           0,92	     0,00	   5,69	   3,40	    6,66	72,98

Agaricia tenuifolia	           0,00	     0,55	   3,27	   0,55	    3,83	76,81

Groups Playa Tiburon  &  Sur

Average dissimilarity = 89,17

	Group Playa Tiburon	Group Sur	       	       	        	     

Species	           Av.Abund	 Av.Abund	Av.Diss	Diss/SD	Contrib%	Cum.%

Orbicella faveolata	               0,00	     4,60	  24,75	   5,93	   27,75	27,75

Colpophyllia natans	               0,00	     2,70	  14,24	   2,83	   15,97	43,72

Pseudodiploria strigosa	               2,38	     0,62	   9,18	   2,78	   10,30	54,01

Siderastrea siderea	               1,64	     0,00	   8,74	   2,45	    9,80	63,81

Diploria labyrinthiformis	               1,38	     0,00	   7,24	   2,77	    8,12	71,93

Montastrea cavernosa	               0,93	     0,00	   4,94	   1,31	    5,54	77,48

Groups Punta Mono  &  Sur

Average dissimilarity = 91,69

	Group Punta Mono	Group Sur	       	       	        	     

Species	        Av.Abund	 Av.Abund	Av.Diss	Diss/SD	Contrib%	Cum.%

Orbicella faveolata	            0,00	     4,60	  25,42	   5,31	   27,72	27,72

Colpophyllia natans	            0,00	     2,70	  14,62	   2,76	   15,94	43,66

Pseudodiploria strigosa	            2,96	     0,62	  12,30	   4,20	   13,42	57,08

Siderastrea siderea	            1,34	     0,00	   7,27	   3,41	    7,93	65,02

Meandrina meandrites	            1,21	     0,00	   6,66	   1,59	    7,27	72,28

Diploria labyrinthiformis	            1,02	     0,00	   5,50	   3,01	    6,00	78,28

Groups Charagato  &  Sur

Average dissimilarity = 76,32

	Group Charagato	Group Sur	       	       	        	     

Species	       Av.Abund	 Av.Abund	Av.Diss	Diss/SD	Contrib%	Cum.%

Orbicella faveolata	           0,00	     4,60	  36,70	   3,85	   48,09	48,09

Colpophyllia natans	           1,86	     2,70	  12,17	   1,14	   15,95	64,04

Pseudodiploria strigosa	           0,40	     0,62	   5,69	   1,64	    7,46	71,50

Agaricia tenuifolia	           0,00	     0,55	   4,00	   0,55	    5,25	76,75

Groups Punta Conejo  &  Sur

Average dissimilarity = 91,13

	Group Punta Conejo	Group Sur	       	       	        	     

Species	          Av.Abund	 Av.Abund	Av.Diss	Diss/SD	Contrib%	Cum.%

Orbicella faveolata	              0,00	     4,60	  36,42	   4,20	   39,96	39,96

Colpophyllia natans	              0,00	     2,70	  20,74	   2,68	   22,76	62,72

Pseudodiploria strigosa	              1,99	     0,62	  11,84	   1,53	   13,00	75,72

Groups La Muerta  &  Sur

Average dissimilarity = 81,38

	Group La Muerta	Group Sur	       	       	        	     

Species	       Av.Abund	 Av.Abund	Av.Diss	Diss/SD	Contrib%	Cum.%

Orbicella faveolata	           0,35	     4,60	  32,90	   2,75	   40,43	40,43

Colpophyllia natans	           0,62	     2,70	  16,97	   1,67	   20,85	61,28

Pseudodiploria strigosa	           1,99	     0,62	  11,52	   2,64	   14,16	75,44

Groups Cominoto  &  Sur

Average dissimilarity = 88,33

	Group Cominoto	Group Sur	       	       	        	     

Species	      Av.Abund	 Av.Abund	Av.Diss	Diss/SD	Contrib%	Cum.%

Orbicella faveolata	          0,00	     4,60	  33,42	   5,89	   37,83	37,83

Colpophyllia natans	          0,00	     2,70	  19,10	   2,93	   21,62	59,45

Pseudodiploria strigosa	          2,96	     0,62	  16,69	   3,71	   18,89	78,34

Groups La Pecha  &  Sur

Average dissimilarity = 97,46

	Group La Pecha	Group Sur	       	       	        	     

Species	      Av.Abund	 Av.Abund	Av.Diss	Diss/SD	Contrib%	Cum.%

Orbicella faveolata	          0,00	     4,60	  44,53	   4,73	   45,69	45,69

Colpophyllia natans	          0,00	     2,70	  25,21	   2,89	   25,86	71,55

Pseudodiploria strigosa	          0,17	     0,62	   5,45	   1,24	    5,59	77,14

Groups Puerto Real  &  Sur

Average dissimilarity = 85,51

	Group Puerto Real	Group Sur	       	       	        	     

Species	         Av.Abund	 Av.Abund	Av.Diss	Diss/SD	Contrib%	Cum.%

Orbicella faveolata	             0,00	     4,60	  31,30	   5,85	   36,60	36,60

Colpophyllia natans	             0,00	     2,70	  17,92	   2,90	   20,95	57,56

Pseudodiploria strigosa	             2,60	     0,62	  12,83	   2,05	   15,01	72,56

Porites astreoides	             1,10	     0,35	   5,35	   1,76	    6,26	78,82

Groups Cayo Agua  &  Sur

Average dissimilarity = 59,76

	Group Cayo Agua	Group Sur	       	       	        	     

Species	       Av.Abund	 Av.Abund	Av.Diss	Diss/SD	Contrib%	Cum.%

Madracis mirabilis	           3,47	     0,00	  13,72	   4,22	   22,95	22,95

Orbicella annularis	           3,05	     0,58	  10,29	   2,11	   17,22	40,17

Colpophyllia natans	           1,15	     2,70	   6,66	   1,49	   11,14	51,31

Orbicella faveolata	           3,24	     4,60	   5,40	   3,48	    9,04	60,35

Madracis decactis	           1,10	     0,00	   4,21	   0,96	    7,05	67,40

Porites astreoides	           0,69	     0,35	   2,85	   0,90	    4,77	72,17

Orbicella sp.	           0,68	     0,00	   2,72	   0,91	    4,56	76,72

Groups Boca de Cote  &  Sur

Average dissimilarity = 64,42

	Group Boca de Cote	Group Sur	       	       	        	     

Species	          Av.Abund	 Av.Abund	Av.Diss	Diss/SD	Contrib%	Cum.%

Siderastrea siderea	              2,54	     0,00	   9,80	   4,73	   15,21	15,21

Colpophyllia natans	              0,17	     2,70	   9,74	   2,47	   15,12	30,33

Montastrea cavernosa	              1,76	     0,00	   6,72	   2,32	   10,43	40,75

Porites astreoides	              1,92	     0,35	   6,22	   2,57	    9,65	50,40

Orbicella faveolata	              3,10	     4,60	   5,93	   1,76	    9,21	59,61

Diploria labyrinthiformis	              1,37	     0,00	   5,40	   2,72	    8,39	68,00

Orbicella annularis	              1,35	     0,58	   4,80	   1,40	    7,45	75,44

Groups Dos Mosquises Sur  &  Sur

Average dissimilarity = 47,34

	Group Dos Mosquises Sur	Group Sur	       	       	        	     

Species	               Av.Abund	 Av.Abund	Av.Diss	Diss/SD	Contrib%	Cum.%

Madracis mirabilis	                   2,55	     0,00	  10,13	   1,60	   21,39	21,39

Orbicella annularis	                   2,43	     0,58	   8,82	   1,38	   18,64	40,03

Orbicella faveolata	                   3,41	     4,60	   4,96	   1,66	   10,47	50,50

Colpophyllia natans	                   2,54	     2,70	   3,62	   1,52	    7,65	58,15

Pseudodiploria strigosa	                   0,45	     0,62	   3,21	   1,65	    6,78	64,93

Agaricia tenuifolia	                   0,17	     0,55	   2,58	   0,72	    5,44	70,37

Siderastrea siderea	                   0,52	     0,00	   2,29	   0,82	    4,83	75,21

Groups Madrisqui  &  Sur

Average dissimilarity = 53,90

	Group Madrisqui	Group Sur	       	       	        	     

Species	       Av.Abund	 Av.Abund	Av.Diss	Diss/SD	Contrib%	Cum.%

Orbicella faveolata	           2,67	     4,60	   9,82	   2,70	   18,21	18,21

Colpophyllia natans	           1,96	     2,70	   6,67	   1,16	   12,37	30,59

Porites astreoides	           1,41	     0,35	   6,11	   1,62	   11,33	41,92

Agaricia sp.	           0,93	     0,17	   4,25	   0,79	    7,88	49,80

Pseudodiploria strigosa	           0,00	     0,62	   3,23	   1,49	    5,99	55,79

Eusmilia fastigata	           0,68	     0,00	   3,20	   0,95	    5,93	61,72

Montastrea cavernosa	           0,52	     0,00	   2,85	   0,56	    5,28	67,00

Agaricia tenuifolia	           0,00	     0,55	   2,62	   0,56	    4,85	71,86

Orbicella annularis	           0,00	     0,58	   2,54	   0,56	    4,71	76,57

Groups Pelona de Rabusqui  &  Sur

Average dissimilarity = 35,85

	Group Pelona de Rabusqui	Group Sur	       	       	        	     

Species	                Av.Abund	 Av.Abund	Av.Diss	Diss/SD	Contrib%	Cum.%

Orbicella annularis	                    2,00	     0,58	   7,96	   1,30	   22,21	22,21

Colpophyllia natans	                    1,87	     2,70	   5,66	   1,30	   15,77	37,99

Agaricia sp.	                    1,10	     0,17	   4,35	   1,15	   12,12	50,11

Orbicella sp.	                    0,74	     0,00	   2,97	   0,56	    8,28	58,38

Pseudodiploria strigosa	                    0,17	     0,62	   2,56	   1,24	    7,15	65,54

Agaricia tenuifolia	                    0,00	     0,55	   2,40	   0,56	    6,71	72,24

Agaricia humilis	                    0,27	     0,35	   2,05	   1,13	    5,72	77,97

Groups Salinas  &  Sur

Average dissimilarity = 67,40

	Group Salinas	Group Sur	       	       	        	     

Species	     Av.Abund	 Av.Abund	Av.Diss	Diss/SD	Contrib%	Cum.%

Orbicella faveolata	         2,24	     4,60	  11,07	   2,30	   16,42	16,42

Colpophyllia natans	         0,27	     2,70	  10,99	   2,36	   16,31	32,73

Siderastrea siderea	         2,20	     0,00	  10,26	   2,53	   15,23	47,96

Montastrea cavernosa	         1,66	     0,00	   7,29	   1,66	   10,81	58,77

Diploria labyrinthiformis	         1,40	     0,00	   6,27	   1,52	    9,30	68,07

Pseudodiploria strigosa	         1,32	     0,62	   4,59	   1,92	    6,81	74,87

Orbicella annularis	         0,35	     0,58	   3,14	   0,77	    4,66	79,54

Groups La Venada  &  Sur

Average dissimilarity = 96,67

	Group La Venada	Group Sur	       	       	        	     

Species	       Av.Abund	 Av.Abund	Av.Diss	Diss/SD	Contrib%	Cum.%

Orbicella faveolata	           0,00	     4,60	  29,20	   6,02	   30,20	30,20

Acropora cervicornis	           4,54	     0,00	  28,64	   7,37	   29,62	59,83

Colpophyllia natans	           0,00	     2,70	  16,74	   2,90	   17,32	77,14

Groups San Agustin  &  Sur

Average dissimilarity = 100,00

	Group San Agustin	Group Sur	       	       	        	     

Species	         Av.Abund	 Av.Abund	Av.Diss	Diss/SD	Contrib%	Cum.%

Orbicella faveolata	             0,00	     4,60	  44,53	   4,73	   44,53	44,53

Colpophyllia natans	             0,00	     2,70	  25,21	   2,89	   25,21	69,74

Pseudodiploria strigosa	             0,00	     0,62	   6,40	   1,49	    6,40	76,14

Groups Blanca  &  Sur

Average dissimilarity = 67,39

	Group Blanca	Group Sur	       	       	        	     

Species	    Av.Abund	 Av.Abund	Av.Diss	Diss/SD	Contrib%	Cum.%

Orbicella faveolata	        0,00	     4,60	  20,46	   6,21	   30,36	30,36

Pseudodiploria strigosa	        2,94	     0,62	  10,19	   4,39	   15,12	45,49

Montastrea cavernosa	        1,70	     0,00	   7,58	   3,80	   11,25	56,73

Colpophyllia natans	        3,84	     2,70	   5,28	   1,13	    7,83	64,56

Dichocoenia stokesii	        1,17	     0,00	   4,94	   1,58	    7,33	71,90

Agaricia tenuifolia	        0,00	     0,55	   2,33	   0,56	    3,46	75,36

Groups Carabela  &  Sur

Average dissimilarity = 91,45

	Group Carabela	Group Sur	       	       	        	     

Species	      Av.Abund	 Av.Abund	Av.Diss	Diss/SD	Contrib%	Cum.%

Orbicella faveolata	          0,00	     4,60	  42,16	   4,45	   46,10	46,10

Colpophyllia natans	          0,66	     2,70	  20,46	   1,80	   22,37	68,47

Pseudodiploria strigosa	          0,00	     0,62	   6,04	   1,47	    6,61	75,08

Groups Punta Cruz  &  Sur

Average dissimilarity = 83,66

	Group Punta Cruz	Group Sur	       	       	        	     

Species	        Av.Abund	 Av.Abund	Av.Diss	Diss/SD	Contrib%	Cum.%

Orbicella faveolata	            0,17	     4,60	  28,33	   4,86	   33,86	33,86

Colpophyllia natans	            0,85	     2,70	  11,73	   1,67	   14,02	47,88

Montastrea cavernosa	            1,66	     0,00	  10,49	   2,13	   12,54	60,41

Dichocoenia stokesii	            1,29	     0,00	   7,98	   1,59	    9,54	69,95

Pseudodiploria strigosa	            1,07	     0,62	   6,48	   1,50	    7,75	77,70

Groups Gabarra  &  Sur

Average dissimilarity = 75,37

	Group Gabarra	Group Sur	       	       	        	     

Species	     Av.Abund	 Av.Abund	Av.Diss	Diss/SD	Contrib%	Cum.%

Orbicella faveolata	         0,00	     4,60	  33,37	   4,17	   44,27	44,27

Colpophyllia natans	         1,17	     2,70	  12,54	   1,33	   16,64	60,92

Pseudodiploria strigosa	         1,57	     0,62	   6,59	   1,39	    8,74	69,65

Siderastrea siderea	         0,76	     0,00	   4,91	   0,90	    6,52	76,17

Groups Garrapata  &  Sur

Average dissimilarity = 65,29

	Group Garrapata	Group Sur	       	       	        	     

Species	       Av.Abund	 Av.Abund	Av.Diss	Diss/SD	Contrib%	Cum.%

Orbicella faveolata	           0,35	     4,60	  19,61	   5,22	   30,03	30,03

Montastrea cavernosa	           2,71	     0,00	  12,44	   7,16	   19,05	49,08

Pseudodiploria strigosa	           3,23	     0,62	  11,85	   5,66	   18,15	67,23

Colpophyllia natans	           3,62	     2,70	   5,82	   1,30	    8,92	76,15

Groups Boca Seca  &  Sur

Average dissimilarity = 70,15

	Group Boca Seca	Group Sur	       	       	        	     

Species	       Av.Abund	 Av.Abund	Av.Diss	Diss/SD	Contrib%	Cum.%

Orbicella faveolata	           0,90	     4,60	  27,57	   2,63	   39,30	39,30

Colpophyllia natans	           0,49	     2,70	  16,61	   1,88	   23,67	62,97

Porites astreoides	           0,75	     0,35	   4,14	   1,05	    5,90	68,88

Pseudodiploria strigosa	           0,45	     0,62	   3,82	   1,14	    5,44	74,32

Agaricia tenuifolia	           0,00	     0,55	   3,72	   0,55	    5,30	79,61

Groups Bajo Caiman  &  Sur

Average dissimilarity = 73,73

	Group Bajo Caiman	Group Sur	       	       	        	     

Species	         Av.Abund	 Av.Abund	Av.Diss	Diss/SD	Contrib%	Cum.%

Orbicella faveolata	             1,12	     4,60	  26,32	   3,95	   35,69	35,69

Colpophyllia natans	             0,45	     2,70	  16,89	   1,97	   22,90	58,60

Porites astreoides	             1,01	     0,35	   7,15	   1,05	    9,70	68,30

Orbicella annularis	             0,45	     0,58	   5,37	   1,04	    7,28	75,58

Groups Medio  &  Sur

Average dissimilarity = 44,67

	Group Medio	Group Sur	       	       	        	     

Species	   Av.Abund	 Av.Abund	Av.Diss	Diss/SD	Contrib%	Cum.%

Colpophyllia natans	       0,35	     2,70	  11,87	   1,99	   26,58	26,58

Agaricia sp.	       1,23	     0,17	   5,41	   1,22	   12,12	38,70

Agaricia tenuifolia	       0,58	     0,55	   4,30	   0,75	    9,62	48,33

Madracis sp.	       1,01	     0,00	   4,23	   0,56	    9,46	57,79

Agaricia agaricites (danai)	       0,75	     0,00	   3,31	   0,81	    7,40	65,19

Pseudodiploria strigosa	       0,17	     0,62	   2,83	   1,23	    6,33	71,52

Orbicella annularis	       0,00	     0,58	   2,60	   0,55	    5,81	77,33

Groups Mero  &  Sur

Average dissimilarity = 80,12

	Group Mero	Group Sur	       	       	        	     

Species	  Av.Abund	 Av.Abund	Av.Diss	Diss/SD	Contrib%	Cum.%

Orbicella faveolata	      0,45	     4,60	  30,19	   2,50	   37,68	37,68

Colpophyllia natans	      1,06	     2,70	  14,85	   1,36	   18,53	56,21

Porites astreoides	      1,14	     0,35	   6,26	   1,44	    7,81	64,02

Pseudodiploria strigosa	      0,17	     0,62	   4,20	   1,13	    5,24	69,26

Agaricia tenuifolia	      0,00	     0,55	   3,61	   0,54	    4,50	73,76

Porites porites	      0,55	     0,00	   3,39	   0,91	    4,23	78,00

Groups Norte  &  Sur

Average dissimilarity = 43,41

	Group Norte	Group Sur	       	       	        	     

Species	   Av.Abund	 Av.Abund	Av.Diss	Diss/SD	Contrib%	Cum.%

Colpophyllia natans	       1,30	     2,70	   6,58	   1,52	   15,17	15,17

Agaricia agaricites (danai)	       1,35	     0,00	   5,79	   1,29	   13,33	28,50

Acropora palmata	       1,19	     0,00	   5,31	   0,94	   12,24	40,74

Orbicella sp.	       1,16	     0,00	   4,84	   0,91	   11,14	51,88

Orbicella annularis	       0,87	     0,58	   4,47	   0,95	   10,31	62,19

Agaricia sp.	       0,85	     0,17	   3,27	   1,30	    7,52	69,71

Pseudodiploria strigosa	       0,00	     0,62	   2,79	   1,50	    6,42	76,13

Groups Sombrero  &  Sur

Average dissimilarity = 40,79

	Group Sombrero	Group Sur	       	       	        	     

Species	      Av.Abund	 Av.Abund	Av.Diss	Diss/SD	Contrib%	Cum.%

Porites astreoides	          1,72	     0,35	   5,70	   2,61	   13,98	13,98

Colpophyllia natans	          3,81	     2,70	   4,86	   1,17	   11,92	25,90

Orbicella faveolata	          3,55	     4,60	   4,36	   2,18	   10,68	36,58

Montastrea cavernosa	          0,85	     0,00	   3,48	   0,96	    8,52	45,10

Pseudodiploria strigosa	          1,17	     0,62	   3,40	   1,25	    8,34	53,44

Stephanocoenia intersepta	          0,58	     0,27	   2,79	   0,75	    6,84	60,28

Agaricia agaricites (danai)	          0,69	     0,00	   2,76	   0,81	    6,78	67,06

Agaricia sp.	          0,58	     0,17	   2,30	   0,92	    5,65	72,71

Agaricia tenuifolia	          0,00	     0,55	   2,15	   0,56	    5,27	77,97

Groups La Pared  &  Cienaga este

Average dissimilarity = 85,52

	Group La Pared	Group Cienaga este	       	       	        	     

Species	      Av.Abund	          Av.Abund	Av.Diss	Diss/SD	Contrib%	Cum.%

Orbicella faveolata	          0,00	              3,63	  19,53	   5,80	   22,84	22,84

Pseudodiploria strigosa	          2,33	              0,17	  11,53	   2,63	   13,48	36,32

Meandrina meandrites	          1,94	              0,17	   9,52	   1,63	   11,14	47,46

Orbicella annularis	          0,00	              1,34	   6,90	   0,96	    8,07	55,53

Montastrea cavernosa	          1,38	              0,40	   6,27	   1,38	    7,33	62,86

Porites astreoides	          0,45	              1,53	   6,19	   1,36	    7,24	70,10

Siderastrea siderea	          1,37	              0,27	   5,95	   1,97	    6,96	77,06

Groups Media Legua  &  Cienaga este

Average dissimilarity = 86,79

	Group Media Legua	Group Cienaga este	       	       	        	     

Species	         Av.Abund	          Av.Abund	Av.Diss	Diss/SD	Contrib%	Cum.%

Orbicella faveolata	             0,17	              3,63	  25,66	   4,89	   29,57	29,57

Pseudodiploria strigosa	             2,20	              0,17	  14,70	   3,83	   16,94	46,51

Porites astreoides	             0,17	              1,53	  10,18	   1,69	   11,73	58,24

Orbicella annularis	             0,00	              1,34	   9,34	   0,96	   10,76	69,01

Siderastrea siderea	             0,85	              0,27	   5,59	   1,32	    6,44	75,45

Groups Punta de Media Legua  &  Cienaga este

Average dissimilarity = 88,05

	Group Punta de Media Legua	Group Cienaga este	       	       	        	     

Species	                  Av.Abund	          Av.Abund	Av.Diss	Diss/SD	Contrib%	Cum.%

Orbicella faveolata	                      0,00	              3,63	  26,23	   3,01	   29,80	29,80

Porites astreoides	                      0,27	              1,53	   9,94	   1,41	   11,29	41,08

Pseudodiploria strigosa	                      1,63	              0,17	   9,59	   1,85	   10,89	51,97

Orbicella annularis	                      0,00	              1,34	   9,07	   0,91	   10,31	62,27

Siderastrea siderea	                      1,59	              0,27	   8,64	   2,21	    9,81	72,09

Montastrea cavernosa	                      1,14	              0,40	   6,86	   1,27	    7,79	79,88

Groups Petaquire  &  Cienaga este

Average dissimilarity = 89,71

	Group Petaquire	Group Cienaga este	       	       	        	     

Species	       Av.Abund	          Av.Abund	Av.Diss	Diss/SD	Contrib%	Cum.%

Orbicella faveolata	           0,23	              3,63	  25,26	   3,42	   28,15	28,15

Pseudodiploria strigosa	           2,75	              0,17	  18,26	   6,58	   20,35	48,51

Porites astreoides	           0,00	              1,53	  11,24	   1,97	   12,53	61,04

Orbicella annularis	           0,00	              1,34	   9,26	   0,94	   10,32	71,36

Siderastrea siderea	           0,92	              0,27	   5,61	   2,33	    6,25	77,61

Groups Playa Tiburon  &  Cienaga este

Average dissimilarity = 86,78

	Group Playa Tiburon	Group Cienaga este	       	       	        	     

Species	           Av.Abund	          Av.Abund	Av.Diss	Diss/SD	Contrib%	Cum.%

Orbicella faveolata	               0,00	              3,63	  21,95	   5,47	   25,29	25,29

Pseudodiploria strigosa	               2,38	              0,17	  13,07	   3,79	   15,06	40,35

Siderastrea siderea	               1,64	              0,27	   8,22	   1,65	    9,47	49,82

Diploria labyrinthiformis	               1,38	              0,00	   8,13	   2,86	    9,36	59,18

Porites astreoides	               0,40	              1,53	   7,94	   1,59	    9,16	68,34

Orbicella annularis	               0,00	              1,34	   7,71	   0,96	    8,88	77,22

Groups Punta Mono  &  Cienaga este

Average dissimilarity = 89,44

	Group Punta Mono	Group Cienaga este	       	       	        	     

Species	        Av.Abund	          Av.Abund	Av.Diss	Diss/SD	Contrib%	Cum.%

Orbicella faveolata	            0,00	              3,63	  22,63	   4,86	   25,30	25,30

Pseudodiploria strigosa	            2,96	              0,17	  16,67	   6,73	   18,64	43,95

Porites astreoides	            0,17	              1,53	   8,56	   1,64	    9,58	53,52

Orbicella annularis	            0,00	              1,34	   7,93	   0,95	    8,87	62,39

Meandrina meandrites	            1,21	              0,17	   6,94	   1,62	    7,76	70,16

Siderastrea siderea	            1,34	              0,27	   6,55	   1,68	    7,32	77,48

Groups Charagato  &  Cienaga este

Average dissimilarity = 98,43

	Group Charagato	Group Cienaga este	       	       	        	     

Species	       Av.Abund	          Av.Abund	Av.Diss	Diss/SD	Contrib%	Cum.%

Orbicella faveolata	           0,00	              3,63	  34,68	   3,52	   35,23	35,23

Colpophyllia natans	           1,86	              0,00	  15,22	   1,49	   15,46	50,69

Porites astreoides	           0,00	              1,53	  14,60	   1,91	   14,83	65,52

Orbicella annularis	           0,00	              1,34	  11,75	   0,94	   11,94	77,46

Groups Punta Conejo  &  Cienaga este

Average dissimilarity = 93,29

	Group Punta Conejo	Group Cienaga este	       	       	        	     

Species	          Av.Abund	          Av.Abund	Av.Diss	Diss/SD	Contrib%	Cum.%

Orbicella faveolata	              0,00	              3,63	  34,30	   3,89	   36,77	36,77

Pseudodiploria strigosa	              1,99	              0,17	  15,62	   1,47	   16,75	53,51

Porites astreoides	              0,17	              1,53	  13,14	   1,60	   14,08	67,60

Orbicella annularis	              0,00	              1,34	  11,65	   0,94	   12,48	80,08

Groups La Muerta  &  Cienaga este

Average dissimilarity = 88,42

	Group La Muerta	Group Cienaga este	       	       	        	     

Species	       Av.Abund	          Av.Abund	Av.Diss	Diss/SD	Contrib%	Cum.%

Orbicella faveolata	           0,35	              3,63	  30,48	   2,38	   34,48	34,48

Pseudodiploria strigosa	           1,99	              0,17	  14,82	   1,90	   16,76	51,23

Porites astreoides	           0,52	              1,53	  12,62	   1,56	   14,27	65,51

Orbicella annularis	           0,00	              1,34	  11,15	   0,92	   12,61	78,11

Groups Cominoto  &  Cienaga este

Average dissimilarity = 88,40

	Group Cominoto	Group Cienaga este	       	       	        	     

Species	      Av.Abund	          Av.Abund	Av.Diss	Diss/SD	Contrib%	Cum.%

Orbicella faveolata	          0,00	              3,63	  30,88	   5,78	   34,94	34,94

Pseudodiploria strigosa	          2,96	              0,17	  23,42	   4,89	   26,50	61,43

Orbicella annularis	          0,00	              1,34	  10,61	   0,96	   12,00	73,43

Porites astreoides	          0,58	              1,53	   9,57	   1,42	   10,82	84,26

Groups La Pecha  &  Cienaga este

Average dissimilarity = 98,83

	Group La Pecha	Group Cienaga este	       	       	        	     

Species	      Av.Abund	          Av.Abund	Av.Diss	Diss/SD	Contrib%	Cum.%

Orbicella faveolata	          0,00	              3,63	  43,52	   4,69	   44,03	44,03

Porites astreoides	          0,00	              1,53	  18,31	   2,11	   18,52	62,56

Orbicella annularis	          0,00	              1,34	  14,45	   0,97	   14,62	77,18

Groups Puerto Real  &  Cienaga este

Average dissimilarity = 79,50

	Group Puerto Real	Group Cienaga este	       	       	        	     

Species	         Av.Abund	          Av.Abund	Av.Diss	Diss/SD	Contrib%	Cum.%

Orbicella faveolata	             0,00	              3,63	  28,64	   5,64	   36,02	36,02

Pseudodiploria strigosa	             2,60	              0,17	  18,42	   2,78	   23,16	59,19

Orbicella annularis	             0,00	              1,34	   9,89	   0,96	   12,44	71,63

Porites astreoides	             1,10	              1,53	   4,89	   1,16	    6,15	77,78

Groups Cayo Agua  &  Cienaga este

Average dissimilarity = 53,39

	Group Cayo Agua	Group Cienaga este	       	       	        	     

Species	       Av.Abund	          Av.Abund	Av.Diss	Diss/SD	Contrib%	Cum.%

Madracis mirabilis	           3,47	              0,27	  13,82	   3,29	   25,88	25,88

Orbicella annularis	           3,05	              1,34	   8,69	   1,51	   16,27	42,15

Porites astreoides	           0,69	              1,53	   5,08	   1,64	    9,51	51,65

Colpophyllia natans	           1,15	              0,00	   4,91	   1,27	    9,20	60,85

Madracis decactis	           1,10	              0,00	   4,58	   0,97	    8,57	69,43

Orbicella sp.	           0,68	              0,40	   3,22	   0,99	    6,03	75,46

Groups Boca de Cote  &  Cienaga este

Average dissimilarity = 50,27

	Group Boca de Cote	Group Cienaga este	       	       	        	     

Species	          Av.Abund	          Av.Abund	Av.Diss	Diss/SD	Contrib%	Cum.%

Siderastrea siderea	              2,54	              0,27	   9,53	   3,10	   18,95	18,95

Montastrea cavernosa	              1,76	              0,40	   6,13	   1,71	   12,20	31,15

Diploria labyrinthiformis	              1,37	              0,00	   5,89	   2,72	   11,72	42,86

Orbicella annularis	              1,35	              1,34	   5,65	   1,43	   11,24	54,10

Madracis mirabilis	              1,01	              0,27	   4,15	   0,86	    8,26	62,36

Orbicella faveolata	              3,10	              3,63	   3,27	   1,18	    6,51	68,86

Porites astreoides	              1,92	              1,53	   3,16	   1,65	    6,29	75,15

Groups Dos Mosquises Sur  &  Cienaga este

Average dissimilarity = 57,77

	Group Dos Mosquises Sur	Group Cienaga este	       	       	        	     

Species	               Av.Abund	          Av.Abund	Av.Diss	Diss/SD	Contrib%	Cum.%

Colpophyllia natans	                   2,54	              0,00	  11,77	   4,08	   20,37	20,37

Madracis mirabilis	                   2,55	              0,27	  10,53	   1,68	   18,23	38,60

Orbicella annularis	                   2,43	              1,34	   8,66	   1,33	   15,00	53,60

Porites astreoides	                   0,17	              1,53	   6,32	   1,67	   10,95	64,54

Orbicella faveolata	                   3,41	              3,63	   3,14	   1,52	    5,44	69,99

Siderastrea siderea	                   0,52	              0,27	   2,71	   0,95	    4,68	74,67

Pseudodiploria strigosa	                   0,45	              0,17	   2,45	   0,77	    4,24	78,91

Groups Madrisqui  &  Cienaga este

Average dissimilarity = 57,68

	Group Madrisqui	Group Cienaga este	       	       	        	     

Species	       Av.Abund	          Av.Abund	Av.Diss	Diss/SD	Contrib%	Cum.%

Colpophyllia natans	           1,96	              0,00	  10,50	   1,67	   18,21	18,21

Orbicella annularis	           0,00	              1,34	   7,16	   0,96	   12,41	30,62

Orbicella faveolata	           2,67	              3,63	   5,64	   1,53	    9,78	40,40

Porites astreoides	           1,41	              1,53	   5,15	   1,35	    8,94	49,33

Agaricia sp.	           0,93	              0,00	   4,64	   0,74	    8,04	57,37

Montastrea cavernosa	           0,52	              0,40	   4,20	   0,77	    7,28	64,66

Eusmilia fastigata	           0,68	              0,00	   3,54	   0,96	    6,15	70,80

Other (Scleractinian massive)	           0,27	              0,17	   2,16	   0,79	    3,75	74,56

Orbicella sp.	           0,00	              0,40	   2,11	   0,56	    3,66	78,22

Groups Pelona de Rabusqui  &  Cienaga este

Average dissimilarity = 52,80

	Group Pelona de Rabusqui	Group Cienaga este	       	       	        	     

Species	                Av.Abund	          Av.Abund	Av.Diss	Diss/SD	Contrib%	Cum.%

Colpophyllia natans	                    1,87	              0,00	   9,25	   2,28	   17,51	17,51

Orbicella annularis	                    2,00	              1,34	   7,99	   1,26	   15,14	32,65

Porites astreoides	                    0,00	              1,53	   7,75	   2,04	   14,68	47,33

Orbicella faveolata	                    4,91	              3,63	   6,53	   3,28	   12,37	59,70

Agaricia sp.	                    1,10	              0,00	   5,59	   1,46	   10,59	70,29

Orbicella sp.	                    0,74	              0,40	   4,32	   0,79	    8,18	78,47

Groups Salinas  &  Cienaga este

Average dissimilarity = 61,95

	Group Salinas	Group Cienaga este	       	       	        	     

Species	     Av.Abund	          Av.Abund	Av.Diss	Diss/SD	Contrib%	Cum.%

Siderastrea siderea	         2,20	              0,27	   9,99	   1,94	   16,13	16,13

Orbicella faveolata	         2,24	              3,63	   7,34	   1,45	   11,85	27,97

Montastrea cavernosa	         1,66	              0,40	   7,17	   1,51	   11,58	39,55

Diploria labyrinthiformis	         1,40	              0,00	   6,92	   1,52	   11,17	50,72

Orbicella annularis	         0,35	              1,34	   6,74	   1,05	   10,89	61,61

Pseudodiploria strigosa	         1,32	              0,17	   6,05	   1,65	    9,77	71,37

Porites astreoides	         0,68	              1,53	   5,57	   1,35	    9,00	80,37

Groups La Venada  &  Cienaga este

Average dissimilarity = 99,38

	Group La Venada	Group Cienaga este	       	       	        	     

Species	       Av.Abund	          Av.Abund	Av.Diss	Diss/SD	Contrib%	Cum.%

Acropora cervicornis	           4,54	              0,00	  32,90	   9,02	   33,11	33,11

Orbicella faveolata	           0,00	              3,63	  26,45	   5,74	   26,61	59,72

Porites astreoides	           0,00	              1,53	  11,14	   2,09	   11,21	70,92

Orbicella annularis	           0,00	              1,34	   9,19	   0,96	    9,24	80,17

Groups San Agustin  &  Cienaga este

Average dissimilarity = 99,06

	Group San Agustin	Group Cienaga este	       	       	        	     

Species	         Av.Abund	          Av.Abund	Av.Diss	Diss/SD	Contrib%	Cum.%

Orbicella faveolata	             0,00	              3,63	  43,52	   4,69	   43,93	43,93

Porites astreoides	             0,00	              1,53	  18,31	   2,11	   18,48	62,41

Orbicella annularis	             0,00	              1,34	  14,45	   0,97	   14,59	77,00

Groups Blanca  &  Cienaga este

Average dissimilarity = 86,37

	Group Blanca	Group Cienaga este	       	       	        	     

Species	    Av.Abund	          Av.Abund	Av.Diss	Diss/SD	Contrib%	Cum.%

Colpophyllia natans	        3,84	              0,00	  18,80	   7,25	   21,77	21,77

Orbicella faveolata	        0,00	              3,63	  17,78	   5,65	   20,58	42,35

Pseudodiploria strigosa	        2,94	              0,17	  13,47	   5,27	   15,59	57,94

Montastrea cavernosa	        1,70	              0,40	   6,56	   1,70	    7,60	65,54

Orbicella annularis	        0,00	              1,34	   6,31	   0,95	    7,31	72,84

Dichocoenia stokesii	        1,17	              0,00	   5,42	   1,59	    6,28	79,12

Groups Carabela  &  Cienaga este

Average dissimilarity = 99,06

	Group Carabela	Group Cienaga este	       	       	        	     

Species	      Av.Abund	          Av.Abund	Av.Diss	Diss/SD	Contrib%	Cum.%

Orbicella faveolata	          0,00	              3,63	  40,78	   4,26	   41,16	41,16

Porites astreoides	          0,00	              1,53	  17,16	   2,05	   17,32	58,48

Orbicella annularis	          0,00	              1,34	  13,62	   0,96	   13,75	72,24

Colpophyllia natans	          0,66	              0,00	   6,05	   0,56	    6,11	78,34

Groups Punta Cruz  &  Cienaga este

Average dissimilarity = 90,56

	Group Punta Cruz	Group Cienaga este	       	       	        	     

Species	        Av.Abund	          Av.Abund	Av.Diss	Diss/SD	Contrib%	Cum.%

Orbicella faveolata	            0,17	              3,63	  25,45	   4,38	   28,10	28,10

Porites astreoides	            0,00	              1,53	  11,22	   2,06	   12,39	40,50

Montastrea cavernosa	            1,66	              0,40	  10,26	   1,68	   11,33	51,82

Orbicella annularis	            0,00	              1,34	   9,25	   0,96	   10,21	62,03

Dichocoenia stokesii	            1,29	              0,00	   9,15	   1,61	   10,10	72,14

Pseudodiploria strigosa	            1,07	              0,17	   7,09	   1,03	    7,82	79,96

Groups Gabarra  &  Cienaga este

Average dissimilarity = 92,09

	Group Gabarra	Group Cienaga este	       	       	        	     

Species	     Av.Abund	          Av.Abund	Av.Diss	Diss/SD	Contrib%	Cum.%

Orbicella faveolata	         0,00	              3,63	  30,97	   3,78	   33,63	33,63

Porites astreoides	         0,17	              1,53	  11,79	   1,58	   12,80	46,43

Pseudodiploria strigosa	         1,57	              0,17	  10,92	   1,94	   11,86	58,28

Orbicella annularis	         0,00	              1,34	  10,61	   0,94	   11,52	69,80

Colpophyllia natans	         1,17	              0,00	   8,86	   1,29	    9,62	79,41

Groups Garrapata  &  Cienaga este

Average dissimilarity = 82,69

	Group Garrapata	Group Cienaga este	       	       	        	     

Species	       Av.Abund	          Av.Abund	Av.Diss	Diss/SD	Contrib%	Cum.%

Colpophyllia natans	           3,62	              0,00	  18,39	   4,42	   22,24	22,24

Orbicella faveolata	           0,35	              3,63	  16,70	   4,23	   20,19	42,44

Pseudodiploria strigosa	           3,23	              0,17	  15,41	   7,71	   18,63	61,07

Montastrea cavernosa	           2,71	              0,40	  11,73	   2,88	   14,18	75,26

Groups Boca Seca  &  Cienaga este

Average dissimilarity = 73,51

	Group Boca Seca	Group Cienaga este	       	       	        	     

Species	       Av.Abund	          Av.Abund	Av.Diss	Diss/SD	Contrib%	Cum.%

Orbicella faveolata	           0,90	              3,63	  24,12	   2,13	   32,82	32,82

Orbicella annularis	           0,00	              1,34	  10,72	   0,95	   14,59	47,41

Porites astreoides	           0,75	              1,53	   8,46	   1,18	   11,51	58,92

Agaricia humilis	           0,52	              0,00	   4,71	   1,60	    6,41	65,33

Colpophyllia natans	           0,49	              0,00	   3,68	   0,56	    5,00	70,33

Pseudodiploria strigosa	           0,45	              0,17	   3,56	   1,02	    4,84	75,17

Groups Bajo Caiman  &  Cienaga este

Average dissimilarity = 65,90

	Group Bajo Caiman	Group Cienaga este	       	       	        	     

Species	         Av.Abund	          Av.Abund	Av.Diss	Diss/SD	Contrib%	Cum.%

Orbicella faveolata	             1,12	              3,63	  22,46	   3,31	   34,08	34,08

Orbicella annularis	             0,45	              1,34	  11,26	   1,23	   17,09	51,17

Porites astreoides	             1,01	              1,53	  10,65	   1,35	   16,16	67,33

Colpophyllia natans	             0,45	              0,00	   3,91	   0,56	    5,94	73,27

Montastrea cavernosa	             0,00	              0,40	   3,48	   0,55	    5,28	78,55

Groups Medio  &  Cienaga este

Average dissimilarity = 57,97

	Group Medio	Group Cienaga este	       	       	        	     

Species	   Av.Abund	          Av.Abund	Av.Diss	Diss/SD	Contrib%	Cum.%

Porites astreoides	       0,00	              1,53	   8,82	   2,00	   15,22	15,22

Orbicella annularis	       0,00	              1,34	   7,37	   0,95	   12,72	27,94

Agaricia sp.	       1,23	              0,00	   6,45	   1,31	   11,12	39,06

Orbicella faveolata	       4,49	              3,63	   5,66	   1,86	    9,76	48,82

Madracis sp.	       1,01	              0,00	   4,63	   0,56	    7,99	56,80

Agaricia agaricites (danai)	       0,75	              0,00	   3,64	   0,81	    6,28	63,09

Agaricia tenuifolia	       0,58	              0,00	   3,31	   0,56	    5,71	68,80

Agaricia humilis	       0,52	              0,00	   2,99	   1,58	    5,16	73,96

Montastrea cavernosa	       0,00	              0,40	   2,29	   0,55	    3,94	77,91

Groups Mero  &  Cienaga este

Average dissimilarity = 78,40

	Group Mero	Group Cienaga este	       	       	        	     

Species	  Av.Abund	          Av.Abund	Av.Diss	Diss/SD	Contrib%	Cum.%

Orbicella faveolata	      0,45	              3,63	  27,60	   2,16	   35,21	35,21

Orbicella annularis	      0,00	              1,34	  10,44	   0,90	   13,32	48,52

Porites astreoides	      1,14	              1,53	   8,16	   0,98	   10,40	58,93

Colpophyllia natans	      1,06	              0,00	   6,12	   0,86	    7,80	66,73

Siderastrea siderea	      0,62	              0,27	   4,34	   0,97	    5,53	72,26

Porites porites	      0,55	              0,00	   3,91	   0,90	    4,99	77,25

Groups Norte  &  Cienaga este

Average dissimilarity = 58,31

	Group Norte	Group Cienaga este	       	       	        	     

Species	   Av.Abund	          Av.Abund	Av.Diss	Diss/SD	Contrib%	Cum.%

Orbicella faveolata	       5,10	              3,63	   7,02	   3,50	   12,04	12,04

Porites astreoides	       0,17	              1,53	   6,55	   1,68	   11,24	23,28

Orbicella annularis	       0,87	              1,34	   6,47	   1,14	   11,09	34,37

Agaricia agaricites (danai)	       1,35	              0,00	   6,36	   1,30	   10,90	45,27

Colpophyllia natans	       1,30	              0,00	   6,01	   2,42	   10,30	55,57

Acropora palmata	       1,19	              0,00	   5,86	   0,94	   10,05	65,62

Orbicella sp.	       1,16	              0,40	   5,34	   1,00	    9,16	74,78

Agaricia sp.	       0,85	              0,00	   3,92	   1,39	    6,72	81,49

Groups Sombrero  &  Cienaga este

Average dissimilarity = 54,31

	Group Sombrero	Group Cienaga este	       	       	        	     

Species	      Av.Abund	          Av.Abund	Av.Diss	Diss/SD	Contrib%	Cum.%

Colpophyllia natans	          3,81	              0,00	  16,92	   8,99	   31,15	31,15

Orbicella annularis	          0,00	              1,34	   5,75	   0,96	   10,59	41,74

Pseudodiploria strigosa	          1,17	              0,17	   4,65	   1,32	    8,56	50,31

Montastrea cavernosa	          0,85	              0,40	   3,78	   0,99	    6,97	57,27

Agaricia agaricites (danai)	          0,69	              0,00	   3,02	   0,81	    5,55	62,82

Porites astreoides	          1,72	              1,53	   2,88	   1,64	    5,29	68,12

Agaricia sp.	          0,58	              0,00	   2,51	   0,85	    4,62	72,74

Stephanocoenia intersepta	          0,58	              0,00	   2,48	   0,56	    4,56	77,30

Groups Sur  &  Cienaga este

Average dissimilarity = 53,02

	Group Sur	Group Cienaga este	       	       	        	     

Species	 Av.Abund	          Av.Abund	Av.Diss	Diss/SD	Contrib%	Cum.%

Colpophyllia natans	     2,70	              0,00	  14,23	   2,91	   26,84	26,84

Orbicella annularis	     0,58	              1,34	   6,98	   1,01	   13,17	40,01

Porites astreoides	     0,35	              1,53	   6,51	   1,41	   12,28	52,30

Orbicella faveolata	     4,60	              3,63	   5,25	   2,55	    9,90	62,20

Pseudodiploria strigosa	     0,62	              0,17	   2,89	   1,29	    5,45	67,65

Agaricia tenuifolia	     0,55	              0,00	   2,79	   0,56	    5,27	72,92

Montastrea cavernosa	     0,00	              0,40	   2,13	   0,56	    4,02	76,94

Groups La Pared  &  Cienaga interno

Average dissimilarity = 71,28

	Group La Pared	Group Cienaga interno	       	       	        	     

Species	      Av.Abund	             Av.Abund	Av.Diss	Diss/SD	Contrib%	Cum.%

Orbicella faveolata	          0,00	                 3,25	  14,26	   8,25	   20,01	20,01

Colpophyllia natans	          0,00	                 3,09	  13,69	   7,56	   19,20	39,21

Meandrina meandrites	          1,94	                 0,00	   8,26	   1,55	   11,59	50,80

Pseudodiploria strigosa	          2,33	                 1,25	   6,20	   1,28	    8,70	59,51

Porites astreoides	          0,45	                 1,28	   4,78	   1,27	    6,71	66,21

Siderastrea siderea	          1,37	                 0,40	   4,65	   2,04	    6,52	72,73

Montastrea cavernosa	          1,38	                 1,94	   3,98	   1,06	    5,58	78,32

Groups Media Legua  &  Cienaga interno

Average dissimilarity = 74,02

	Group Media Legua	Group Cienaga interno	       	       	        	     

Species	         Av.Abund	             Av.Abund	Av.Diss	Diss/SD	Contrib%	Cum.%

Orbicella faveolata	             0,17	                 3,25	  17,42	   6,69	   23,54	23,54

Colpophyllia natans	             0,17	                 3,09	  16,73	   5,64	   22,60	46,14

Montastrea cavernosa	             0,45	                 1,94	   8,39	   2,15	   11,34	57,48

Pseudodiploria strigosa	             2,20	                 1,25	   7,34	   1,31	    9,92	67,40

Porites astreoides	             0,17	                 1,28	   6,50	   1,22	    8,78	76,18

Groups Punta de Media Legua  &  Cienaga interno

Average dissimilarity = 73,56

	Group Punta de Media Legua	Group Cienaga interno	       	       	        	     

Species	                  Av.Abund	             Av.Abund	Av.Diss	Diss/SD	Contrib%	Cum.%

Orbicella faveolata	                      0,00	                 3,25	  17,82	   4,24	   24,23	24,23

Colpophyllia natans	                      0,17	                 3,09	  16,44	   3,10	   22,35	46,58

Siderastrea siderea	                      1,59	                 0,40	   6,95	   2,29	    9,45	56,03

Pseudodiploria strigosa	                      1,63	                 1,25	   6,67	   1,33	    9,07	65,11

Porites astreoides	                      0,27	                 1,28	   6,36	   1,16	    8,65	73,76

Montastrea cavernosa	                      1,14	                 1,94	   5,66	   1,13	    7,70	81,45

Groups Petaquire  &  Cienaga interno

Average dissimilarity = 75,29

	Group Petaquire	Group Cienaga interno	       	       	        	     

Species	       Av.Abund	             Av.Abund	Av.Diss	Diss/SD	Contrib%	Cum.%

Orbicella faveolata	           0,23	                 3,25	  17,07	   4,33	   22,67	22,67

Colpophyllia natans	           0,23	                 3,09	  16,38	   3,93	   21,76	44,43

Montastrea cavernosa	           0,23	                 1,94	   9,49	   2,80	   12,61	57,04

Pseudodiploria strigosa	           2,75	                 1,25	   9,34	   1,45	   12,41	69,45

Porites astreoides	           0,00	                 1,28	   6,81	   1,20	    9,05	78,50

Groups Playa Tiburon  &  Cienaga interno

Average dissimilarity = 71,06

	Group Playa Tiburon	Group Cienaga interno	       	       	        	     

Species	           Av.Abund	             Av.Abund	Av.Diss	Diss/SD	Contrib%	Cum.%

Orbicella faveolata	               0,00	                 3,25	  15,67	   8,21	   22,05	22,05

Colpophyllia natans	               0,00	                 3,09	  15,05	   7,13	   21,18	43,23

Pseudodiploria strigosa	               2,38	                 1,25	   6,76	   1,34	    9,51	52,74

Siderastrea siderea	               1,64	                 0,40	   6,38	   1,82	    8,97	61,71

Porites astreoides	               0,40	                 1,28	   5,62	   1,24	    7,91	69,62

Diploria labyrinthiformis	               1,38	                 0,27	   5,38	   2,05	    7,58	77,20

Groups Punta Mono  &  Cienaga interno

Average dissimilarity = 78,20

	Group Punta Mono	Group Cienaga interno	       	       	        	     

Species	        Av.Abund	             Av.Abund	Av.Diss	Diss/SD	Contrib%	Cum.%

Orbicella faveolata	            0,00	                 3,25	  16,04	   7,22	   20,51	20,51

Colpophyllia natans	            0,00	                 3,09	  15,41	   6,33	   19,71	40,22

Pseudodiploria strigosa	            2,96	                 1,25	   9,03	   1,58	   11,55	51,77

Montastrea cavernosa	            0,27	                 1,94	   8,22	   2,33	   10,52	62,29

Meandrina meandrites	            1,21	                 0,00	   6,04	   1,59	    7,73	70,02

Porites astreoides	            0,17	                 1,28	   5,70	   1,20	    7,29	77,30

Groups Charagato  &  Cienaga interno

Average dissimilarity = 75,18

	Group Charagato	Group Cienaga interno	       	       	        	     

Species	       Av.Abund	             Av.Abund	Av.Diss	Diss/SD	Contrib%	Cum.%

Orbicella faveolata	           0,00	                 3,25	  22,06	   5,85	   29,34	29,34

Montastrea cavernosa	           0,00	                 1,94	  12,95	   4,33	   17,22	46,57

Colpophyllia natans	           1,86	                 3,09	  11,34	   1,19	   15,08	61,65

Porites astreoides	           0,00	                 1,28	   8,13	   1,21	   10,81	72,46

Pseudodiploria strigosa	           0,40	                 1,25	   7,65	   1,15	   10,18	82,64

Groups Punta Conejo  &  Cienaga interno

Average dissimilarity = 87,92

	Group Punta Conejo	Group Cienaga interno	       	       	        	     

Species	          Av.Abund	             Av.Abund	Av.Diss	Diss/SD	Contrib%	Cum.%

Orbicella faveolata	              0,00	                 3,25	  21,94	   6,70	   24,95	24,95

Colpophyllia natans	              0,00	                 3,09	  21,17	   5,12	   24,07	49,02

Montastrea cavernosa	              0,00	                 1,94	  12,88	   4,60	   14,65	63,68

Pseudodiploria strigosa	              1,99	                 1,25	  10,75	   1,42	   12,23	75,90

Groups La Muerta  &  Cienaga interno

Average dissimilarity = 76,29

	Group La Muerta	Group Cienaga interno	       	       	        	     

Species	       Av.Abund	             Av.Abund	Av.Diss	Diss/SD	Contrib%	Cum.%

Orbicella faveolata	           0,35	                 3,25	  19,29	   2,89	   25,28	25,28

Colpophyllia natans	           0,62	                 3,09	  16,76	   1,84	   21,96	47,25

Montastrea cavernosa	           0,00	                 1,94	  12,40	   3,97	   16,26	63,50

Pseudodiploria strigosa	           1,99	                 1,25	   9,43	   1,37	   12,36	75,86

Groups Cominoto  &  Cienaga interno

Average dissimilarity = 81,02

	Group Cominoto	Group Cienaga interno	       	       	        	     

Species	      Av.Abund	             Av.Abund	Av.Diss	Diss/SD	Contrib%	Cum.%

Orbicella faveolata	          0,00	                 3,25	  20,43	  12,38	   25,22	25,22

Colpophyllia natans	          0,00	                 3,09	  19,69	   7,45	   24,30	49,51

Montastrea cavernosa	          0,00	                 1,94	  12,02	   5,32	   14,83	64,35

Pseudodiploria strigosa	          2,96	                 1,25	  11,76	   1,41	   14,51	78,86

Groups La Pecha  &  Cienaga interno

Average dissimilarity = 98,15

	Group La Pecha	Group Cienaga interno	       	       	        	     

Species	      Av.Abund	             Av.Abund	Av.Diss	Diss/SD	Contrib%	Cum.%

Orbicella faveolata	          0,00	                 3,25	  26,04	  11,83	   26,53	26,53

Colpophyllia natans	          0,00	                 3,09	  25,19	   5,93	   25,67	52,19

Montastrea cavernosa	          0,00	                 1,94	  15,23	   6,13	   15,52	67,71

Porites astreoides	          0,00	                 1,28	   9,48	   1,24	    9,65	77,37

Groups Puerto Real  &  Cienaga interno

Average dissimilarity = 78,29

	Group Puerto Real	Group Cienaga interno	       	       	        	     

Species	         Av.Abund	             Av.Abund	Av.Diss	Diss/SD	Contrib%	Cum.%

Orbicella faveolata	             0,00	                 3,25	  19,29	  10,66	   24,64	24,64

Colpophyllia natans	             0,00	                 3,09	  18,58	   7,32	   23,73	48,37

Montastrea cavernosa	             0,00	                 1,94	  11,36	   5,06	   14,51	62,89

Pseudodiploria strigosa	             2,60	                 1,25	   9,83	   1,35	   12,55	75,44

Groups Cayo Agua  &  Cienaga interno

Average dissimilarity = 64,57

	Group Cayo Agua	Group Cienaga interno	       	       	        	     

Species	       Av.Abund	             Av.Abund	Av.Diss	Diss/SD	Contrib%	Cum.%

Madracis mirabilis	           3,47	                 0,00	  12,79	   4,22	   19,81	19,81

Orbicella annularis	           3,05	                 0,00	  11,22	   3,49	   17,38	37,18

Colpophyllia natans	           1,15	                 3,09	   7,24	   2,00	   11,21	48,40

Montastrea cavernosa	           0,17	                 1,94	   6,40	   3,37	    9,91	58,30

Pseudodiploria strigosa	           0,00	                 1,25	   4,47	   1,20	    6,92	65,22

Porites astreoides	           0,69	                 1,28	   4,17	   1,26	    6,45	71,67

Madracis decactis	           1,10	                 0,00	   3,93	   0,96	    6,09	77,77

Groups Boca de Cote  &  Cienaga interno

Average dissimilarity = 51,39

	Group Boca de Cote	Group Cienaga interno	       	       	        	     

Species	          Av.Abund	             Av.Abund	Av.Diss	Diss/SD	Contrib%	Cum.%

Colpophyllia natans	              0,17	                 3,09	  10,60	   5,24	   20,63	20,63

Siderastrea siderea	              2,54	                 0,40	   7,52	   2,58	   14,64	35,27

Orbicella annularis	              1,35	                 0,00	   4,70	   1,52	    9,14	44,41

Diploria labyrinthiformis	              1,37	                 0,27	   4,11	   2,05	    8,00	52,42

Porites astreoides	              1,92	                 1,28	   3,90	   1,35	    7,60	60,01

Pseudodiploria strigosa	              0,62	                 1,25	   3,87	   1,24	    7,53	67,54

Madracis mirabilis	              1,01	                 0,00	   3,39	   0,73	    6,60	74,14

Montastrea cavernosa	              1,76	                 1,94	   2,81	   1,45	    5,47	79,61

Groups Dos Mosquises Sur  &  Cienaga interno

Average dissimilarity = 52,55

	Group Dos Mosquises Sur	Group Cienaga interno	       	       	        	     

Species	               Av.Abund	             Av.Abund	Av.Diss	Diss/SD	Contrib%	Cum.%

Madracis mirabilis	                   2,55	                 0,00	   9,44	   1,60	   17,96	17,96

Orbicella annularis	                   2,43	                 0,00	   9,01	   1,48	   17,15	35,11

Montastrea cavernosa	                   0,00	                 1,94	   7,41	   4,26	   14,10	49,21

Pseudodiploria strigosa	                   0,45	                 1,25	   4,50	   1,22	    8,56	57,76

Porites astreoides	                   0,17	                 1,28	   4,49	   1,20	    8,55	66,32

Orbicella faveolata	                   3,41	                 3,25	   2,74	   1,17	    5,21	71,53

Siderastrea siderea	                   0,52	                 0,40	   2,67	   0,98	    5,09	76,62

Groups Madrisqui  &  Cienaga interno

Average dissimilarity = 46,71

	Group Madrisqui	Group Cienaga interno	       	       	        	     

Species	       Av.Abund	             Av.Abund	Av.Diss	Diss/SD	Contrib%	Cum.%

Montastrea cavernosa	           0,52	                 1,94	   6,82	   1,93	   14,61	14,61

Colpophyllia natans	           1,96	                 3,09	   5,51	   0,91	   11,80	26,40

Pseudodiploria strigosa	           0,00	                 1,25	   5,46	   1,19	   11,68	38,09

Porites astreoides	           1,41	                 1,28	   5,09	   1,34	   10,91	48,99

Agaricia sp.	           0,93	                 0,00	   3,88	   0,74	    8,31	57,31

Orbicella faveolata	           2,67	                 3,25	   3,33	   1,37	    7,13	64,44

Eusmilia fastigata	           0,68	                 0,00	   2,94	   0,95	    6,29	70,73

Siderastrea siderea	           0,00	                 0,40	   2,11	   0,56	    4,52	75,25

Groups Pelona de Rabusqui  &  Cienaga interno

Average dissimilarity = 55,43

	Group Pelona de Rabusqui	Group Cienaga interno	       	       	        	     

Species	                Av.Abund	             Av.Abund	Av.Diss	Diss/SD	Contrib%	Cum.%

Orbicella annularis	                    2,00	                 0,00	   8,29	   1,51	   14,96	14,96

Montastrea cavernosa	                    0,00	                 1,94	   8,03	   4,25	   14,49	29,46

Orbicella faveolata	                    4,91	                 3,25	   7,20	   2,50	   12,99	42,45

Colpophyllia natans	                    1,87	                 3,09	   5,28	   1,27	    9,53	51,97

Porites astreoides	                    0,00	                 1,28	   5,15	   1,22	    9,29	61,27

Pseudodiploria strigosa	                    0,17	                 1,25	   4,76	   1,16	    8,59	69,86

Agaricia sp.	                    1,10	                 0,00	   4,66	   1,46	    8,40	78,26

Groups Salinas  &  Cienaga interno

Average dissimilarity = 51,58

	Group Salinas	Group Cienaga interno	       	       	        	     

Species	     Av.Abund	             Av.Abund	Av.Diss	Diss/SD	Contrib%	Cum.%

Colpophyllia natans	         0,27	                 3,09	  11,96	   5,82	   23,19	23,19

Siderastrea siderea	         2,20	                 0,40	   7,76	   1,96	   15,05	38,24

Diploria labyrinthiformis	         1,40	                 0,27	   5,23	   1,60	   10,13	48,37

Orbicella faveolata	         2,24	                 3,25	   4,91	   1,38	    9,52	57,89

Pseudodiploria strigosa	         1,32	                 1,25	   4,70	   1,29	    9,10	66,99

Porites astreoides	         0,68	                 1,28	   4,68	   1,34	    9,06	76,06

Groups La Venada  &  Cienaga interno

Average dissimilarity = 97,34

	Group La Venada	Group Cienaga interno	       	       	        	     

Species	       Av.Abund	             Av.Abund	Av.Diss	Diss/SD	Contrib%	Cum.%

Acropora cervicornis	           4,54	                 0,00	  25,65	   7,74	   26,35	26,35

Orbicella faveolata	           0,00	                 3,25	  18,15	  10,13	   18,65	45,00

Colpophyllia natans	           0,00	                 3,09	  17,47	   7,47	   17,95	62,95

Montastrea cavernosa	           0,00	                 1,94	  10,70	   4,90	   10,99	73,94

Porites astreoides	           0,00	                 1,28	   6,79	   1,23	    6,98	80,92

Groups San Agustin  &  Cienaga interno

Average dissimilarity = 97,29

	Group San Agustin	Group Cienaga interno	       	       	        	     

Species	         Av.Abund	             Av.Abund	Av.Diss	Diss/SD	Contrib%	Cum.%

Orbicella faveolata	             0,00	                 3,25	  26,04	  11,83	   26,76	26,76

Colpophyllia natans	             0,00	                 3,09	  25,19	   5,93	   25,89	52,65

Montastrea cavernosa	             0,17	                 1,94	  13,88	   3,59	   14,26	66,92

Porites astreoides	             0,00	                 1,28	   9,48	   1,24	    9,74	76,66

Groups Blanca  &  Cienaga interno

Average dissimilarity = 47,05

	Group Blanca	Group Cienaga interno	       	       	        	     

Species	    Av.Abund	             Av.Abund	Av.Diss	Diss/SD	Contrib%	Cum.%

Orbicella faveolata	        0,00	                 3,25	  13,19	   7,47	   28,03	28,03

Pseudodiploria strigosa	        2,94	                 1,25	   7,29	   1,50	   15,50	43,53

Dichocoenia stokesii	        1,17	                 0,00	   4,58	   1,58	    9,74	53,27

Porites astreoides	        0,62	                 1,28	   4,10	   1,33	    8,71	61,98

Colpophyllia natans	        3,84	                 3,09	   3,10	   2,33	    6,59	68,57

Siderastrea siderea	        0,52	                 0,40	   2,71	   0,96	    5,75	74,33

Montastrea cavernosa	        1,70	                 1,94	   2,29	   1,64	    4,86	79,19

Groups Carabela  &  Cienaga interno

Average dissimilarity = 88,36

	Group Carabela	Group Cienaga interno	       	       	        	     

Species	      Av.Abund	             Av.Abund	Av.Diss	Diss/SD	Contrib%	Cum.%

Orbicella faveolata	          0,00	                 3,25	  24,85	   8,81	   28,13	28,13

Colpophyllia natans	          0,66	                 3,09	  19,56	   1,86	   22,14	50,27

Montastrea cavernosa	          0,17	                 1,94	  13,20	   3,53	   14,94	65,20

Porites astreoides	          0,00	                 1,28	   9,08	   1,23	   10,28	75,48

Groups Punta Cruz  &  Cienaga interno

Average dissimilarity = 65,85

	Group Punta Cruz	Group Cienaga interno	       	       	        	     

Species	        Av.Abund	             Av.Abund	Av.Diss	Diss/SD	Contrib%	Cum.%

Orbicella faveolata	            0,17	                 3,25	  17,30	   5,82	   26,27	26,27

Colpophyllia natans	            0,85	                 3,09	  12,78	   2,90	   19,40	45,67

Dichocoenia stokesii	            1,29	                 0,00	   7,16	   1,60	   10,88	56,54

Pseudodiploria strigosa	            1,07	                 1,25	   7,00	   1,26	   10,63	67,17

Porites astreoides	            0,00	                 1,28	   6,82	   1,23	   10,36	77,53

Groups Gabarra  &  Cienaga interno

Average dissimilarity = 72,47

	Group Gabarra	Group Cienaga interno	       	       	        	     

Species	     Av.Abund	             Av.Abund	Av.Diss	Diss/SD	Contrib%	Cum.%

Orbicella faveolata	         0,00	                 3,25	  20,35	   6,32	   28,07	28,07

Colpophyllia natans	         1,17	                 3,09	  12,77	   1,60	   17,61	45,69

Montastrea cavernosa	         0,00	                 1,94	  11,97	   4,37	   16,51	62,20

Porites astreoides	         0,17	                 1,28	   7,15	   1,20	    9,86	72,06

Pseudodiploria strigosa	         1,57	                 1,25	   6,86	   1,37	    9,47	81,53

Groups Garrapata  &  Cienaga interno

Average dissimilarity = 41,18

	Group Garrapata	Group Cienaga interno	       	       	        	     

Species	       Av.Abund	             Av.Abund	Av.Diss	Diss/SD	Contrib%	Cum.%

Orbicella faveolata	           0,35	                 3,25	  12,16	   4,14	   29,54	29,54

Pseudodiploria strigosa	           3,23	                 1,25	   8,59	   1,62	   20,85	50,39

Porites astreoides	           0,62	                 1,28	   4,24	   1,37	   10,30	60,69

Colpophyllia natans	           3,62	                 3,09	   3,93	   3,24	    9,55	70,25

Montastrea cavernosa	           2,71	                 1,94	   3,45	   1,22	    8,38	78,63

Groups Boca Seca  &  Cienaga interno

Average dissimilarity = 72,74

	Group Boca Seca	Group Cienaga interno	       	       	        	     

Species	       Av.Abund	             Av.Abund	Av.Diss	Diss/SD	Contrib%	Cum.%

Colpophyllia natans	           0,49	                 3,09	  16,97	   2,44	   23,33	23,33

Orbicella faveolata	           0,90	                 3,25	  15,10	   2,05	   20,76	44,09

Montastrea cavernosa	           0,00	                 1,94	  12,09	   4,66	   16,62	60,71

Pseudodiploria strigosa	           0,45	                 1,25	   6,70	   1,19	    9,21	69,92

Porites astreoides	           0,75	                 1,28	   6,43	   1,28	    8,84	78,76

Groups Bajo Caiman  &  Cienaga interno

Average dissimilarity = 73,38

	Group Bajo Caiman	Group Cienaga interno	       	       	        	     

Species	         Av.Abund	             Av.Abund	Av.Diss	Diss/SD	Contrib%	Cum.%

Colpophyllia natans	             0,45	                 3,09	  17,36	   2,78	   23,66	23,66

Orbicella faveolata	             1,12	                 3,25	  13,73	   4,00	   18,71	42,37

Montastrea cavernosa	             0,00	                 1,94	  12,37	   4,86	   16,86	59,23

Porites astreoides	             1,01	                 1,28	   7,85	   1,22	   10,69	69,92

Pseudodiploria strigosa	             0,00	                 1,25	   7,64	   1,19	   10,42	80,34

Groups Medio  &  Cienaga interno

Average dissimilarity = 63,99

	Group Medio	Group Cienaga interno	       	       	        	     

Species	   Av.Abund	             Av.Abund	Av.Diss	Diss/SD	Contrib%	Cum.%

Colpophyllia natans	       0,35	                 3,09	  12,84	   3,48	   20,07	20,07

Montastrea cavernosa	       0,00	                 1,94	   8,91	   4,16	   13,92	33,99

Orbicella faveolata	       4,49	                 3,25	   6,28	   1,56	    9,81	43,80

Porites astreoides	       0,00	                 1,28	   5,69	   1,22	    8,89	52,69

Agaricia sp.	       1,23	                 0,00	   5,33	   1,27	    8,33	61,02

Pseudodiploria strigosa	       0,17	                 1,25	   5,23	   1,17	    8,18	69,20

Madracis sp.	       1,01	                 0,00	   3,92	   0,56	    6,13	75,34

Groups Mero  &  Cienaga interno

Average dissimilarity = 74,77

	Group Mero	Group Cienaga interno	       	       	        	     

Species	  Av.Abund	             Av.Abund	Av.Diss	Diss/SD	Contrib%	Cum.%

Orbicella faveolata	      0,45	                 3,25	  17,59	   2,70	   23,53	23,53

Colpophyllia natans	      1,06	                 3,09	  14,53	   1,44	   19,43	42,96

Montastrea cavernosa	      0,00	                 1,94	  11,73	   3,40	   15,69	58,64

Pseudodiploria strigosa	      0,17	                 1,25	   6,92	   1,10	    9,26	67,91

Porites astreoides	      1,14	                 1,28	   6,60	   1,24	    8,83	76,73

Groups Norte  &  Cienaga interno

Average dissimilarity = 61,73

	Group Norte	Group Cienaga interno	       	       	        	     

Species	   Av.Abund	             Av.Abund	Av.Diss	Diss/SD	Contrib%	Cum.%

Montastrea cavernosa	       0,00	                 1,94	   7,66	   4,38	   12,41	12,41

Orbicella faveolata	       5,10	                 3,25	   7,58	   2,69	   12,28	24,68

Colpophyllia natans	       1,30	                 3,09	   7,32	   2,41	   11,86	36,55

Agaricia agaricites (danai)	       1,35	                 0,00	   5,37	   1,29	    8,69	45,24

Acropora palmata	       1,19	                 0,00	   4,91	   0,94	    7,95	53,19

Pseudodiploria strigosa	       0,00	                 1,25	   4,82	   1,19	    7,81	61,00

Porites astreoides	       0,17	                 1,28	   4,64	   1,21	    7,51	68,51

Orbicella sp.	       1,16	                 0,00	   4,49	   0,91	    7,28	75,79

Groups Sombrero  &  Cienaga interno

Average dissimilarity = 34,20

	Group Sombrero	Group Cienaga interno	       	       	        	     

Species	      Av.Abund	             Av.Abund	Av.Diss	Diss/SD	Contrib%	Cum.%

Montastrea cavernosa	          0,85	                 1,94	   4,62	   1,60	   13,51	13,51

Pseudodiploria strigosa	          1,17	                 1,25	   4,16	   1,35	   12,18	25,69

Porites astreoides	          1,72	                 1,28	   3,78	   1,48	   11,05	36,74

Colpophyllia natans	          3,81	                 3,09	   2,73	   1,67	    7,99	44,73

Agaricia agaricites (danai)	          0,69	                 0,00	   2,58	   0,81	    7,53	52,26

Agaricia sp.	          0,58	                 0,00	   2,14	   0,85	    6,26	58,53

Stephanocoenia intersepta	          0,58	                 0,00	   2,12	   0,56	    6,20	64,72

Orbicella faveolata	          3,55	                 3,25	   2,02	   1,16	    5,91	70,63

Siderastrea siderea	          0,17	                 0,40	   1,99	   0,74	    5,83	76,46

Groups Sur  &  Cienaga interno

Average dissimilarity = 42,65

	Group Sur	Group Cienaga interno	       	       	        	     

Species	 Av.Abund	             Av.Abund	Av.Diss	Diss/SD	Contrib%	Cum.%

Montastrea cavernosa	     0,00	                 1,94	   8,42	   4,40	   19,74	19,74

Orbicella faveolata	     4,60	                 3,25	   6,19	   2,12	   14,51	34,25

Porites astreoides	     0,35	                 1,28	   4,77	   1,20	   11,19	45,44

Pseudodiploria strigosa	     0,62	                 1,25	   4,29	   1,33	   10,06	55,50

Colpophyllia natans	     2,70	                 3,09	   3,85	   1,29	    9,03	64,53

Agaricia tenuifolia	     0,55	                 0,17	   2,70	   0,71	    6,34	70,86

Orbicella annularis	     0,58	                 0,00	   2,28	   0,56	    5,34	76,21

Groups Cienaga este  &  Cienaga interno

Average dissimilarity = 55,09

	Group Cienaga este	Group Cienaga interno	       	       	        	     

Species	          Av.Abund	             Av.Abund	Av.Diss	Diss/SD	Contrib%	Cum.%

Colpophyllia natans	              0,00	                 3,09	  15,05	   8,30	   27,32	27,32

Montastrea cavernosa	              0,40	                 1,94	   7,67	   2,27	   13,93	41,25

Orbicella annularis	              1,34	                 0,00	   6,29	   0,96	   11,43	52,67

Pseudodiploria strigosa	              0,17	                 1,25	   5,41	   1,21	    9,83	62,50

Porites astreoides	              1,53	                 1,28	   5,18	   1,36	    9,41	71,91

Siderastrea siderea	              0,27	                 0,40	   2,82	   0,76	    5,13	77,03

Groups La Pared  &  Cienaga oeste

Average dissimilarity = 75,01

	Group La Pared	Group Cienaga oeste	       	       	        	     

Species	      Av.Abund	           Av.Abund	Av.Diss	Diss/SD	Contrib%	Cum.%

Colpophyllia natans	          0,00	               3,00	  14,69	   2,95	   19,59	19,59

Orbicella faveolata	          0,00	               2,25	  10,03	   2,09	   13,37	32,96

Meandrina meandrites	          1,94	               0,00	   9,00	   1,47	   12,00	44,96

Pseudodiploria strigosa	          2,33	               0,90	   7,86	   1,39	   10,47	55,44

Montastrea cavernosa	          1,38	               1,22	   5,01	   1,11	    6,68	62,11

Siderastrea siderea	          1,37	               0,97	   4,90	   1,70	    6,53	68,65

Dendrogyra cylindrus	          0,00	               0,89	   3,58	   0,94	    4,78	73,42

Diploria labyrinthiformis	          0,72	               0,00	   3,58	   0,92	    4,77	78,19

Groups Media Legua  &  Cienaga oeste

Average dissimilarity = 75,27

	Group Media Legua	Group Cienaga oeste	       	       	        	     

Species	         Av.Abund	           Av.Abund	Av.Diss	Diss/SD	Contrib%	Cum.%

Colpophyllia natans	             0,17	               3,00	  18,80	   2,33	   24,98	24,98

Orbicella faveolata	             0,17	               2,25	  12,05	   1,89	   16,01	40,99

Pseudodiploria strigosa	             2,20	               0,90	   9,66	   1,33	   12,84	53,83

Montastrea cavernosa	             0,45	               1,22	   6,43	   1,15	    8,54	62,37

Siderastrea siderea	             0,85	               0,97	   6,28	   1,48	    8,35	70,71

Porites astreoides	             0,17	               1,02	   5,24	   1,87	    6,96	77,68

Groups Punta de Media Legua  &  Cienaga oeste

Average dissimilarity = 75,86

	Group Punta de Media Legua	Group Cienaga oeste	       	       	        	     

Species	                  Av.Abund	           Av.Abund	Av.Diss	Diss/SD	Contrib%	Cum.%

Colpophyllia natans	                      0,17	               3,00	  18,67	   1,77	   24,62	24,62

Orbicella faveolata	                      0,00	               2,25	  12,81	   1,97	   16,89	41,51

Siderastrea siderea	                      1,59	               0,97	   7,50	   1,66	    9,88	51,39

Pseudodiploria strigosa	                      1,63	               0,90	   7,35	   1,38	    9,69	61,08

Montastrea cavernosa	                      1,14	               1,22	   6,10	   0,98	    8,04	69,12

Porites astreoides	                      0,27	               1,02	   5,41	   1,79	    7,13	76,24

Groups Petaquire  &  Cienaga oeste

Average dissimilarity = 77,55

	Group Petaquire	Group Cienaga oeste	       	       	        	     

Species	       Av.Abund	           Av.Abund	Av.Diss	Diss/SD	Contrib%	Cum.%

Colpophyllia natans	           0,23	               3,00	  18,48	   2,01	   23,83	23,83

Pseudodiploria strigosa	           2,75	               0,90	  12,65	   1,56	   16,32	40,14

Orbicella faveolata	           0,23	               2,25	  11,77	   1,71	   15,17	55,32

Montastrea cavernosa	           0,23	               1,22	   6,78	   1,15	    8,75	64,06

Siderastrea siderea	           0,92	               0,97	   6,25	   2,37	    8,06	72,13

Porites astreoides	           0,00	               1,02	   6,22	   3,29	    8,03	80,15

Groups Playa Tiburon  &  Cienaga oeste

Average dissimilarity = 75,39

	Group Playa Tiburon	Group Cienaga oeste	       	       	        	     

Species	           Av.Abund	           Av.Abund	Av.Diss	Diss/SD	Contrib%	Cum.%

Colpophyllia natans	               0,00	               3,00	  16,40	   2,74	   21,76	21,76

Orbicella faveolata	               0,00	               2,25	  11,08	   2,13	   14,70	36,46

Pseudodiploria strigosa	               2,38	               0,90	   8,60	   1,38	   11,40	47,86

Diploria labyrinthiformis	               1,38	               0,00	   7,32	   2,40	    9,71	57,57

Siderastrea siderea	               1,64	               0,97	   6,55	   1,44	    8,69	66,25

Montastrea cavernosa	               0,93	               1,22	   5,18	   1,19	    6,87	73,12

Porites astreoides	               0,40	               1,02	   4,83	   2,34	    6,40	79,53

Groups Punta Mono  &  Cienaga oeste

Average dissimilarity = 80,99

	Group Punta Mono	Group Cienaga oeste	       	       	        	     

Species	        Av.Abund	           Av.Abund	Av.Diss	Diss/SD	Contrib%	Cum.%

Colpophyllia natans	            0,00	               3,00	  16,89	   2,59	   20,85	20,85

Pseudodiploria strigosa	            2,96	               0,90	  11,77	   1,76	   14,53	35,39

Orbicella faveolata	            0,00	               2,25	  11,37	   2,12	   14,03	49,42

Meandrina meandrites	            1,21	               0,00	   6,76	   1,49	    8,34	57,76

Montastrea cavernosa	            0,27	               1,22	   5,82	   1,11	    7,19	64,95

Siderastrea siderea	            1,34	               0,97	   5,76	   1,69	    7,12	72,06

Diploria labyrinthiformis	            1,02	               0,00	   5,57	   2,55	    6,88	78,94

Groups Charagato  &  Cienaga oeste

Average dissimilarity = 71,53

	Group Charagato	Group Cienaga oeste	       	       	        	     

Species	       Av.Abund	           Av.Abund	Av.Diss	Diss/SD	Contrib%	Cum.%

Orbicella faveolata	           0,00	               2,25	  16,25	   2,18	   22,72	22,72

Colpophyllia natans	           1,86	               3,00	  14,21	   0,90	   19,87	42,59

Montastrea cavernosa	           0,00	               1,22	   8,91	   1,09	   12,46	55,04

Porites astreoides	           0,00	               1,02	   7,82	   3,06	   10,94	65,98

Siderastrea siderea	           0,27	               0,97	   7,03	   0,97	    9,83	75,82

Groups Punta Conejo  &  Cienaga oeste

Average dissimilarity = 88,75

	Group Punta Conejo	Group Cienaga oeste	       	       	        	     

Species	          Av.Abund	           Av.Abund	Av.Diss	Diss/SD	Contrib%	Cum.%

Colpophyllia natans	              0,00	               3,00	  25,22	   1,92	   28,42	28,42

Orbicella faveolata	              0,00	               2,25	  16,11	   2,23	   18,15	46,57

Pseudodiploria strigosa	              1,99	               0,90	  12,43	   1,21	   14,00	60,58

Montastrea cavernosa	              0,00	               1,22	   8,85	   1,10	    9,97	70,55

Siderastrea siderea	              0,17	               0,97	   6,94	   0,99	    7,82	78,37

Groups La Muerta  &  Cienaga oeste

Average dissimilarity = 77,80

	Group La Muerta	Group Cienaga oeste	       	       	        	     

Species	       Av.Abund	           Av.Abund	Av.Diss	Diss/SD	Contrib%	Cum.%

Colpophyllia natans	           0,62	               3,00	  20,29	   1,28	   26,07	26,07

Orbicella faveolata	           0,35	               2,25	  13,98	   1,66	   17,97	44,04

Pseudodiploria strigosa	           1,99	               0,90	  11,50	   1,41	   14,78	58,83

Montastrea cavernosa	           0,00	               1,22	   8,50	   1,08	   10,92	69,75

Porites astreoides	           0,52	               1,02	   7,67	   2,59	    9,86	79,61

Groups Cominoto  &  Cienaga oeste

Average dissimilarity = 83,02

	Group Cominoto	Group Cienaga oeste	       	       	        	     

Species	      Av.Abund	           Av.Abund	Av.Diss	Diss/SD	Contrib%	Cum.%

Colpophyllia natans	          0,00	               3,00	  22,73	   2,31	   27,38	27,38

Pseudodiploria strigosa	          2,96	               0,90	  16,69	   1,54	   20,11	47,49

Orbicella faveolata	          0,00	               2,25	  14,78	   2,33	   17,81	65,30

Montastrea cavernosa	          0,00	               1,22	   8,17	   1,15	    9,84	75,13

Groups La Pecha  &  Cienaga oeste

Average dissimilarity = 97,97

	Group La Pecha	Group Cienaga oeste	       	       	        	     

Species	      Av.Abund	           Av.Abund	Av.Diss	Diss/SD	Contrib%	Cum.%

Colpophyllia natans	          0,00	               3,00	  31,97	   1,79	   32,63	32,63

Orbicella faveolata	          0,00	               2,25	  19,72	   2,44	   20,13	52,77

Montastrea cavernosa	          0,00	               1,22	  10,69	   1,09	   10,91	63,68

Porites astreoides	          0,00	               1,02	   9,58	   3,60	    9,78	73,46

Siderastrea siderea	          0,00	               0,97	   8,20	   0,89	    8,37	81,82

Groups Puerto Real  &  Cienaga oeste

Average dissimilarity = 78,14

	Group Puerto Real	Group Cienaga oeste	       	       	        	     

Species	         Av.Abund	           Av.Abund	Av.Diss	Diss/SD	Contrib%	Cum.%

Colpophyllia natans	             0,00	               3,00	  21,14	   2,40	   27,05	27,05

Orbicella faveolata	             0,00	               2,25	  13,87	   2,28	   17,76	44,81

Pseudodiploria strigosa	             2,60	               0,90	  12,80	   1,32	   16,38	61,18

Montastrea cavernosa	             0,00	               1,22	   7,68	   1,15	    9,83	71,01

Siderastrea siderea	             0,00	               0,97	   5,92	   0,92	    7,58	78,59

Groups Cayo Agua  &  Cienaga oeste

Average dissimilarity = 69,79

	Group Cayo Agua	Group Cienaga oeste	       	       	        	     

Species	       Av.Abund	           Av.Abund	Av.Diss	Diss/SD	Contrib%	Cum.%

Madracis mirabilis	           3,47	               0,00	  13,73	   3,55	   19,67	19,67

Orbicella annularis	           3,05	               0,00	  12,04	   3,08	   17,25	36,92

Colpophyllia natans	           1,15	               3,00	   7,60	   1,65	   10,89	47,81

Orbicella faveolata	           3,24	               2,25	   5,64	   1,17	    8,08	55,89

Montastrea cavernosa	           0,17	               1,22	   4,34	   1,28	    6,23	62,11

Madracis decactis	           1,10	               0,27	   4,24	   1,04	    6,07	68,18

Siderastrea siderea	           0,00	               0,97	   3,60	   0,95	    5,16	73,34

Porites astreoides	           0,69	               1,02	   3,35	   1,61	    4,80	78,14

Groups Boca de Cote  &  Cienaga oeste

Average dissimilarity = 59,48

	Group Boca de Cote	Group Cienaga oeste	       	       	        	     

Species	          Av.Abund	           Av.Abund	Av.Diss	Diss/SD	Contrib%	Cum.%

Colpophyllia natans	              0,17	               3,00	  11,09	   2,93	   18,65	18,65

Siderastrea siderea	              2,54	               0,97	   6,43	   1,37	   10,82	29,46

Orbicella faveolata	              3,10	               2,25	   5,64	   1,28	    9,49	38,95

Diploria labyrinthiformis	              1,37	               0,00	   5,41	   2,49	    9,09	48,04

Orbicella annularis	              1,35	               0,00	   5,02	   1,48	    8,44	56,48

Montastrea cavernosa	              1,76	               1,22	   4,23	   1,25	    7,11	63,58

Porites astreoides	              1,92	               1,02	   3,75	   1,60	    6,31	69,90

Madracis mirabilis	              1,01	               0,00	   3,61	   0,72	    6,07	75,97

Groups Dos Mosquises Sur  &  Cienaga oeste

Average dissimilarity = 55,96

	Group Dos Mosquises Sur	Group Cienaga oeste	       	       	        	     

Species	               Av.Abund	           Av.Abund	Av.Diss	Diss/SD	Contrib%	Cum.%

Madracis mirabilis	                   2,55	               0,00	  10,14	   1,54	   18,11	18,11

Orbicella annularis	                   2,43	               0,00	   9,68	   1,42	   17,30	35,42

Orbicella faveolata	                   3,41	               2,25	   6,91	   1,17	   12,34	47,76

Montastrea cavernosa	                   0,00	               1,22	   4,85	   1,22	    8,67	56,42

Siderastrea siderea	                   0,52	               0,97	   3,96	   1,25	    7,07	63,49

Pseudodiploria strigosa	                   0,45	               0,90	   3,55	   1,31	    6,34	69,83

Dendrogyra cylindrus	                   0,49	               0,89	   3,55	   0,97	    6,34	76,17

Groups Madrisqui  &  Cienaga oeste

Average dissimilarity = 53,79

	Group Madrisqui	Group Cienaga oeste	       	       	        	     

Species	       Av.Abund	           Av.Abund	Av.Diss	Diss/SD	Contrib%	Cum.%

Colpophyllia natans	           1,96	               3,00	   6,51	   0,92	   12,11	12,11

Orbicella faveolata	           2,67	               2,25	   6,47	   1,53	   12,02	24,13

Montastrea cavernosa	           0,52	               1,22	   5,73	   1,18	   10,65	34,78

Porites astreoides	           1,41	               1,02	   4,70	   1,79	    8,75	43,52

Siderastrea siderea	           0,00	               0,97	   4,46	   0,94	    8,30	51,82

Agaricia sp.	           0,93	               0,00	   4,22	   0,72	    7,85	59,67

Pseudodiploria strigosa	           0,00	               0,90	   3,95	   1,42	    7,34	67,01

Dendrogyra cylindrus	           0,00	               0,89	   3,69	   0,94	    6,86	73,87

Eusmilia fastigata	           0,68	               0,00	   3,22	   0,92	    5,98	79,84

Groups Pelona de Rabusqui  &  Cienaga oeste

Average dissimilarity = 62,11

	Group Pelona de Rabusqui	Group Cienaga oeste	       	       	        	     

Species	                Av.Abund	           Av.Abund	Av.Diss	Diss/SD	Contrib%	Cum.%

Orbicella faveolata	                    4,91	               2,25	  13,13	   1,60	   21,14	21,14

Orbicella annularis	                    2,00	               0,00	   9,03	   1,44	   14,53	35,67

Colpophyllia natans	                    1,87	               3,00	   5,95	   1,18	    9,59	45,25

Montastrea cavernosa	                    0,00	               1,22	   5,28	   1,21	    8,51	53,76

Agaricia sp.	                    1,10	               0,00	   5,08	   1,39	    8,18	61,94

Porites astreoides	                    0,00	               1,02	   4,49	   3,28	    7,22	69,16

Siderastrea siderea	                    0,27	               0,97	   4,21	   1,10	    6,78	75,94

Groups Salinas  &  Cienaga oeste

Average dissimilarity = 58,27

	Group Salinas	Group Cienaga oeste	       	       	        	     

Species	     Av.Abund	           Av.Abund	Av.Diss	Diss/SD	Contrib%	Cum.%

Colpophyllia natans	         0,27	               3,00	  12,75	   2,75	   21,87	21,87

Siderastrea siderea	         2,20	               0,97	   7,18	   1,30	   12,33	34,20

Diploria labyrinthiformis	         1,40	               0,00	   6,30	   1,45	   10,81	45,01

Orbicella faveolata	         2,24	               2,25	   6,01	   1,29	   10,31	55,32

Montastrea cavernosa	         1,66	               1,22	   5,44	   1,17	    9,34	64,66

Pseudodiploria strigosa	         1,32	               0,90	   4,54	   1,28	    7,79	72,45

Dendrogyra cylindrus	         0,00	               0,89	   3,49	   0,93	    6,00	78,44

Groups La Venada  &  Cienaga oeste

Average dissimilarity = 97,33

	Group La Venada	Group Cienaga oeste	       	       	        	     

Species	       Av.Abund	           Av.Abund	Av.Diss	Diss/SD	Contrib%	Cum.%

Acropora cervicornis	           4,54	               0,00	  29,26	   3,56	   30,07	30,07

Colpophyllia natans	           0,00	               3,00	  19,59	   2,52	   20,12	50,19

Orbicella faveolata	           0,00	               2,25	  12,98	   2,24	   13,33	63,53

Montastrea cavernosa	           0,00	               1,22	   7,19	   1,17	    7,39	70,91

Porites astreoides	           0,00	               1,02	   6,18	   3,67	    6,35	77,26

Groups San Agustin  &  Cienaga oeste

Average dissimilarity = 97,97

	Group San Agustin	Group Cienaga oeste	       	       	        	     

Species	         Av.Abund	           Av.Abund	Av.Diss	Diss/SD	Contrib%	Cum.%

Colpophyllia natans	             0,00	               3,00	  31,97	   1,79	   32,63	32,63

Orbicella faveolata	             0,00	               2,25	  19,72	   2,44	   20,13	52,77

Montastrea cavernosa	             0,17	               1,22	  10,36	   1,12	   10,57	63,34

Porites astreoides	             0,00	               1,02	   9,58	   3,60	    9,78	73,12

Siderastrea siderea	             0,00	               0,97	   8,20	   0,89	    8,37	81,48

Groups Blanca  &  Cienaga oeste

Average dissimilarity = 50,54

	Group Blanca	Group Cienaga oeste	       	       	        	     

Species	    Av.Abund	           Av.Abund	Av.Diss	Diss/SD	Contrib%	Cum.%

Pseudodiploria strigosa	        2,94	               0,90	   9,56	   1,87	   18,91	18,91

Orbicella faveolata	        0,00	               2,25	   9,25	   2,05	   18,30	37,21

Dichocoenia stokesii	        1,17	               0,00	   4,96	   1,51	    9,81	47,02

Siderastrea siderea	        0,52	               0,97	   4,13	   1,20	    8,17	55,19

Montastrea cavernosa	        1,70	               1,22	   4,11	   1,08	    8,13	63,32

Colpophyllia natans	        3,84	               3,00	   3,68	   1,38	    7,28	70,60

Dendrogyra cylindrus	        0,00	               0,89	   3,33	   0,94	    6,60	77,19

Groups Carabela  &  Cienaga oeste

Average dissimilarity = 87,81

	Group Carabela	Group Cienaga oeste	       	       	        	     

Species	      Av.Abund	           Av.Abund	Av.Diss	Diss/SD	Contrib%	Cum.%

Colpophyllia natans	          0,66	               3,00	  25,11	   1,29	   28,59	28,59

Orbicella faveolata	          0,00	               2,25	  18,66	   2,36	   21,25	49,84

Montastrea cavernosa	          0,17	               1,22	   9,82	   1,13	   11,18	61,03

Porites astreoides	          0,00	               1,02	   9,04	   3,43	   10,29	71,32

Siderastrea siderea	          0,00	               0,97	   7,79	   0,89	    8,87	80,19

Groups Punta Cruz  &  Cienaga oeste

Average dissimilarity = 68,58

	Group Punta Cruz	Group Cienaga oeste	       	       	        	     

Species	        Av.Abund	           Av.Abund	Av.Diss	Diss/SD	Contrib%	Cum.%

Colpophyllia natans	            0,85	               3,00	  14,28	   1,74	   20,82	20,82

Orbicella faveolata	            0,17	               2,25	  11,98	   1,87	   17,47	38,29

Dichocoenia stokesii	            1,29	               0,00	   8,15	   1,45	   11,88	50,17

Montastrea cavernosa	            1,66	               1,22	   6,89	   1,02	   10,04	60,22

Pseudodiploria strigosa	            1,07	               0,90	   6,80	   1,23	    9,91	70,13

Porites astreoides	            0,00	               1,02	   6,22	   3,55	    9,07	79,20

Groups Gabarra  &  Cienaga oeste

Average dissimilarity = 68,43

	Group Gabarra	Group Cienaga oeste	       	       	        	     

Species	     Av.Abund	           Av.Abund	Av.Diss	Diss/SD	Contrib%	Cum.%

Colpophyllia natans	         1,17	               3,00	  14,98	   1,10	   21,88	21,88

Orbicella faveolata	         0,00	               2,25	  14,79	   2,17	   21,61	43,49

Montastrea cavernosa	         0,00	               1,22	   8,15	   1,11	   11,91	55,40

Pseudodiploria strigosa	         1,57	               0,90	   7,43	   1,11	   10,86	66,26

Siderastrea siderea	         0,76	               0,97	   6,87	   1,02	   10,04	76,31

Groups Garrapata  &  Cienaga oeste

Average dissimilarity = 45,83

	Group Garrapata	Group Cienaga oeste	       	       	        	     

Species	       Av.Abund	           Av.Abund	Av.Diss	Diss/SD	Contrib%	Cum.%

Pseudodiploria strigosa	           3,23	               0,90	  11,22	   2,09	   24,48	24,48

Orbicella faveolata	           0,35	               2,25	   8,39	   1,74	   18,31	42,78

Montastrea cavernosa	           2,71	               1,22	   7,25	   1,29	   15,82	58,60

Colpophyllia natans	           3,62	               3,00	   4,51	   1,68	    9,84	68,44

Siderastrea siderea	           0,52	               0,97	   4,31	   1,75	    9,42	77,86

Groups Boca Seca  &  Cienaga oeste

Average dissimilarity = 70,43

	Group Boca Seca	Group Cienaga oeste	       	       	        	     

Species	       Av.Abund	           Av.Abund	Av.Diss	Diss/SD	Contrib%	Cum.%

Colpophyllia natans	           0,49	               3,00	  19,79	   1,55	   28,10	28,10

Orbicella faveolata	           0,90	               2,25	  11,78	   1,75	   16,72	44,83

Montastrea cavernosa	           0,00	               1,22	   8,23	   1,12	   11,69	56,52

Siderastrea siderea	           0,00	               0,97	   6,34	   0,91	    9,01	65,53

Dendrogyra cylindrus	           0,00	               0,89	   4,98	   0,93	    7,07	72,60

Pseudodiploria strigosa	           0,45	               0,90	   4,89	   1,24	    6,95	79,54

Groups Bajo Caiman  &  Cienaga oeste

Average dissimilarity = 71,23

	Group Bajo Caiman	Group Cienaga oeste	       	       	        	     

Species	         Av.Abund	           Av.Abund	Av.Diss	Diss/SD	Contrib%	Cum.%

Colpophyllia natans	             0,45	               3,00	  20,30	   1,63	   28,50	28,50

Orbicella faveolata	             1,12	               2,25	   8,66	   1,46	   12,16	40,66

Montastrea cavernosa	             0,00	               1,22	   8,44	   1,12	   11,86	52,52

Porites astreoides	             1,01	               1,02	   7,99	   1,64	   11,22	63,74

Siderastrea siderea	             0,00	               0,97	   6,50	   0,91	    9,13	72,86

Pseudodiploria strigosa	             0,00	               0,90	   5,63	   1,42	    7,91	80,77

Groups Medio  &  Cienaga oeste

Average dissimilarity = 72,49

	Group Medio	Group Cienaga oeste	       	       	        	     

Species	   Av.Abund	           Av.Abund	Av.Diss	Diss/SD	Contrib%	Cum.%

Colpophyllia natans	       0,35	               3,00	  13,89	   2,15	   19,17	19,17

Orbicella faveolata	       4,49	               2,25	  13,04	   1,33	   17,98	37,15

Montastrea cavernosa	       0,00	               1,22	   5,90	   1,18	    8,14	45,29

Agaricia sp.	       1,23	               0,00	   5,85	   1,24	    8,06	53,35

Porites astreoides	       0,00	               1,02	   5,03	   3,24	    6,94	60,29

Siderastrea siderea	       0,00	               0,97	   4,57	   0,93	    6,31	66,60

Madracis sp.	       1,01	               0,00	   4,23	   0,55	    5,84	72,44

Dendrogyra cylindrus	       0,00	               0,89	   3,77	   0,93	    5,20	77,64

Groups Mero  &  Cienaga oeste

Average dissimilarity = 70,52

	Group Mero	Group Cienaga oeste	       	       	        	     

Species	  Av.Abund	           Av.Abund	Av.Diss	Diss/SD	Contrib%	Cum.%

Colpophyllia natans	      1,06	               3,00	  17,73	   1,08	   25,14	25,14

Orbicella faveolata	      0,45	               2,25	  12,15	   1,44	   17,23	42,37

Montastrea cavernosa	      0,00	               1,22	   8,01	   1,05	   11,35	53,72

Siderastrea siderea	      0,62	               0,97	   6,51	   1,04	    9,23	62,95

Porites astreoides	      1,14	               1,02	   5,55	   1,34	    7,87	70,82

Pseudodiploria strigosa	      0,17	               0,90	   5,05	   1,24	    7,16	77,98

Groups Norte  &  Cienaga oeste

Average dissimilarity = 68,22

	Group Norte	Group Cienaga oeste	       	       	        	     

Species	   Av.Abund	           Av.Abund	Av.Diss	Diss/SD	Contrib%	Cum.%

Orbicella faveolata	       5,10	               2,25	  13,16	   1,70	   19,30	19,30

Colpophyllia natans	       1,30	               3,00	   7,70	   1,73	   11,28	30,58

Agaricia agaricites (danai)	       1,35	               0,00	   5,80	   1,25	    8,51	39,09

Acropora palmata	       1,19	               0,00	   5,33	   0,92	    7,82	46,91

Montastrea cavernosa	       0,00	               1,22	   5,02	   1,22	    7,36	54,27

Orbicella sp.	       1,16	               0,00	   4,85	   0,89	    7,10	61,37

Siderastrea siderea	       0,00	               0,97	   3,91	   0,94	    5,72	67,09

Orbicella annularis	       0,87	               0,00	   3,68	   0,75	    5,39	72,48

Agaricia sp.	       0,85	               0,00	   3,58	   1,34	    5,25	77,73

Groups Sombrero  &  Cienaga oeste

Average dissimilarity = 41,61

	Group Sombrero	Group Cienaga oeste	       	       	        	     

Species	      Av.Abund	           Av.Abund	Av.Diss	Diss/SD	Contrib%	Cum.%

Orbicella faveolata	          3,55	               2,25	   6,47	   1,18	   15,54	15,54

Montastrea cavernosa	          0,85	               1,22	   4,20	   1,22	   10,10	25,64

Siderastrea siderea	          0,17	               0,97	   3,72	   1,10	    8,95	34,58

Pseudodiploria strigosa	          1,17	               0,90	   3,64	   1,15	    8,74	43,33

Colpophyllia natans	          3,81	               3,00	   3,25	   1,22	    7,82	51,14

Porites astreoides	          1,72	               1,02	   3,13	   1,49	    7,53	58,67

Dendrogyra cylindrus	          0,00	               0,89	   3,10	   0,94	    7,45	66,12

Agaricia agaricites (danai)	          0,69	               0,00	   2,77	   0,80	    6,65	72,77

Agaricia sp.	          0,58	               0,00	   2,30	   0,83	    5,53	78,30

Groups Sur  &  Cienaga oeste

Average dissimilarity = 49,04

	Group Sur	Group Cienaga oeste	       	       	        	     

Species	 Av.Abund	           Av.Abund	Av.Diss	Diss/SD	Contrib%	Cum.%

Orbicella faveolata	     4,60	               2,25	  12,38	   1,47	   25,25	25,25

Montastrea cavernosa	     0,00	               1,22	   5,55	   1,20	   11,32	36,57

Colpophyllia natans	     2,70	               3,00	   4,48	   1,11	    9,13	45,70

Siderastrea siderea	     0,00	               0,97	   4,31	   0,94	    8,79	54,49

Dendrogyra cylindrus	     0,00	               0,89	   3,58	   0,94	    7,29	61,78

Porites astreoides	     0,35	               1,02	   3,18	   1,29	    6,48	68,26

Pseudodiploria strigosa	     0,62	               0,90	   2,95	   1,29	    6,01	74,27

Agaricia tenuifolia	     0,55	               0,00	   2,54	   0,55	    5,17	79,44

Groups Cienaga este  &  Cienaga oeste

Average dissimilarity = 63,65

	Group Cienaga este	Group Cienaga oeste	       	       	        	     

Species	          Av.Abund	           Av.Abund	Av.Diss	Diss/SD	Contrib%	Cum.%

Colpophyllia natans	              0,00	               3,00	  16,38	   2,85	   25,74	25,74

Orbicella faveolata	              3,63	               2,25	   9,08	   1,12	   14,26	40,00

Orbicella annularis	              1,34	               0,00	   6,95	   0,93	   10,92	50,92

Montastrea cavernosa	              0,40	               1,22	   5,82	   1,17	    9,14	60,06

Siderastrea siderea	              0,27	               0,97	   4,89	   1,08	    7,69	67,75

Porites astreoides	              1,53	               1,02	   4,04	   1,04	    6,34	74,09

Dendrogyra cylindrus	              0,00	               0,89	   3,91	   0,94	    6,14	80,23

Groups Cienaga interno  &  Cienaga oeste

Average dissimilarity = 36,50

	Group Cienaga interno	Group Cienaga oeste	       	       	        	     

Species	             Av.Abund	           Av.Abund	Av.Diss	Diss/SD	Contrib%	Cum.%

Orbicella faveolata	                 3,25	               2,25	   6,37	   1,24	   17,46	17,46

Montastrea cavernosa	                 1,94	               1,22	   4,64	   1,12	   12,70	30,16

Pseudodiploria strigosa	                 1,25	               0,90	   4,51	   1,21	   12,35	42,51

Siderastrea siderea	                 0,40	               0,97	   4,16	   1,02	   11,41	53,91

Porites astreoides	                 1,28	               1,02	   4,14	   1,43	   11,33	65,25

Dendrogyra cylindrus	                 0,00	               0,89	   3,33	   0,94	    9,12	74,37

Colpophyllia natans	                 3,09	               3,00	   2,10	   1,15	    5,76	80,13

Groups La Pared  &  Guabinitas

Average dissimilarity = 65,79

	Group La Pared	Group Guabinitas	       	       	        	     

Species	      Av.Abund	        Av.Abund	Av.Diss	Diss/SD	Contrib%	Cum.%

Meandrina meandrites	          1,94	            0,00	  12,00	   1,52	   18,24	18,24

Siderastrea siderea	          1,37	            1,47	   9,59	   4,96	   14,57	32,81

Pseudodiploria strigosa	          2,33	            0,97	   9,13	   1,89	   13,87	46,68

Montastrea cavernosa	          1,38	            1,07	   6,38	   1,20	    9,70	56,38

Diploria labyrinthiformis	          0,72	            0,00	   4,89	   0,95	    7,43	63,81

Other (Scleractinian massive)	          0,62	            0,00	   4,40	   0,90	    6,68	70,49

Porites astreoides	          0,45	            0,72	   3,60	   1,19	    5,47	75,97

Groups Media Legua  &  Guabinitas

Average dissimilarity = 62,43

	Group Media Legua	Group Guabinitas	       	       	        	     

Species	         Av.Abund	        Av.Abund	Av.Diss	Diss/SD	Contrib%	Cum.%

Siderastrea siderea	             0,85	            1,47	  13,64	   1,87	   21,84	21,84

Pseudodiploria strigosa	             2,20	            0,97	  11,74	   2,22	   18,80	40,64

Montastrea cavernosa	             0,45	            1,07	   8,75	   1,28	   14,02	54,66

Porites astreoides	             0,17	            0,72	   6,03	   1,40	    9,66	64,32

Meandrina meandrites	             0,60	            0,00	   6,02	   0,55	    9,65	73,96

Orbicella faveolata	             0,17	            0,52	   4,64	   1,04	    7,44	81,40

Groups Punta de Media Legua  &  Guabinitas

Average dissimilarity = 61,92

	Group Punta de Media Legua	Group Guabinitas	       	       	        	     

Species	                  Av.Abund	        Av.Abund	Av.Diss	Diss/SD	Contrib%	Cum.%

Siderastrea siderea	                      1,59	            1,47	  14,04	   1,74	   22,68	22,68

Pseudodiploria strigosa	                      1,63	            0,97	   9,54	   2,12	   15,40	38,08

Montastrea cavernosa	                      1,14	            1,07	   8,95	   1,05	   14,45	52,54

Porites astreoides	                      0,27	            0,72	   6,23	   1,18	   10,06	62,60

Diploria labyrinthiformis	                      0,83	            0,00	   5,12	   0,77	    8,26	70,86

Orbicella faveolata	                      0,00	            0,52	   4,50	   0,87	    7,27	78,12

Groups Petaquire  &  Guabinitas

Average dissimilarity = 66,56

	Group Petaquire	Group Guabinitas	       	       	        	     

Species	       Av.Abund	        Av.Abund	Av.Diss	Diss/SD	Contrib%	Cum.%

Pseudodiploria strigosa	           2,75	            0,97	  16,53	   4,22	   24,83	24,83

Siderastrea siderea	           0,92	            1,47	  13,60	   2,42	   20,43	45,26

Montastrea cavernosa	           0,23	            1,07	   9,17	   1,15	   13,78	59,05

Porites astreoides	           0,00	            0,72	   6,67	   1,49	   10,02	69,07

Orbicella faveolata	           0,23	            0,52	   4,62	   1,00	    6,94	76,01

Groups Playa Tiburon  &  Guabinitas

Average dissimilarity = 61,72

	Group Playa Tiburon	Group Guabinitas	       	       	        	     

Species	           Av.Abund	        Av.Abund	Av.Diss	Diss/SD	Contrib%	Cum.%

Siderastrea siderea	               1,64	            1,47	  11,39	   2,06	   18,46	18,46

Pseudodiploria strigosa	               2,38	            0,97	  10,34	   2,35	   16,76	35,22

Diploria labyrinthiformis	               1,38	            0,00	  10,22	   2,78	   16,56	51,78

Montastrea cavernosa	               0,93	            1,07	   6,46	   1,33	   10,47	62,25

Porites astreoides	               0,40	            0,72	   5,61	   1,59	    9,08	71,34

Meandrina meandrites	               0,75	            0,00	   5,22	   0,79	    8,47	79,80

Groups Punta Mono  &  Guabinitas

Average dissimilarity = 70,68

	Group Punta Mono	Group Guabinitas	       	       	        	     

Species	        Av.Abund	        Av.Abund	Av.Diss	Diss/SD	Contrib%	Cum.%

Pseudodiploria strigosa	            2,96	            0,97	  14,72	   4,05	   20,83	20,83

Siderastrea siderea	            1,34	            1,47	  11,46	   3,13	   16,21	37,05

Meandrina meandrites	            1,21	            0,00	   9,66	   1,60	   13,67	50,72

Diploria labyrinthiformis	            1,02	            0,00	   7,88	   3,07	   11,15	61,87

Montastrea cavernosa	            0,27	            1,07	   7,64	   1,24	   10,80	72,67

Porites astreoides	            0,17	            0,72	   4,87	   1,36	    6,89	79,57

Groups Charagato  &  Guabinitas

Average dissimilarity = 89,46

	Group Charagato	Group Guabinitas	       	       	        	     

Species	       Av.Abund	        Av.Abund	Av.Diss	Diss/SD	Contrib%	Cum.%

Colpophyllia natans	           1,86	            0,17	  21,08	   1,64	   23,56	23,56

Siderastrea siderea	           0,27	            1,47	  17,22	   0,98	   19,25	42,81

Montastrea cavernosa	           0,00	            1,07	  14,77	   1,07	   16,51	59,32

Pseudodiploria strigosa	           0,40	            0,97	  14,55	   1,36	   16,26	75,58

Groups Punta Conejo  &  Guabinitas

Average dissimilarity = 76,70

	Group Punta Conejo	Group Guabinitas	       	       	        	     

Species	          Av.Abund	        Av.Abund	Av.Diss	Diss/SD	Contrib%	Cum.%

Pseudodiploria strigosa	              1,99	            0,97	  19,06	   1,74	   24,85	24,85

Siderastrea siderea	              0,17	            1,47	  16,88	   1,00	   22,00	46,86

Montastrea cavernosa	              0,00	            1,07	  14,38	   1,12	   18,74	65,60

Porites astreoides	              0,17	            0,72	   8,64	   1,28	   11,26	76,86

Groups La Muerta  &  Guabinitas

Average dissimilarity = 77,05

	Group La Muerta	Group Guabinitas	       	       	        	     

Species	       Av.Abund	        Av.Abund	Av.Diss	Diss/SD	Contrib%	Cum.%

Pseudodiploria strigosa	           1,99	            0,97	  18,66	   2,46	   24,22	24,22

Siderastrea siderea	           0,00	            1,47	  15,85	   0,89	   20,58	44,80

Montastrea cavernosa	           0,00	            1,07	  13,75	   1,03	   17,85	62,65

Porites astreoides	           0,52	            0,72	  10,54	   1,54	   13,69	76,33

Groups Cominoto  &  Guabinitas

Average dissimilarity = 68,57

	Group Cominoto	Group Guabinitas	       	       	        	     

Species	      Av.Abund	        Av.Abund	Av.Diss	Diss/SD	Contrib%	Cum.%

Pseudodiploria strigosa	          2,96	            0,97	  23,62	   3,21	   34,44	34,44

Siderastrea siderea	          0,00	            1,47	  14,56	   0,96	   21,23	55,67

Montastrea cavernosa	          0,00	            1,07	  12,12	   1,26	   17,67	73,35

Porites astreoides	          0,58	            0,72	   7,93	   1,36	   11,56	84,91

Groups La Pecha  &  Guabinitas

Average dissimilarity = 93,43

	Group La Pecha	Group Guabinitas	       	       	        	     

Species	      Av.Abund	        Av.Abund	Av.Diss	Diss/SD	Contrib%	Cum.%

Siderastrea siderea	          0,00	            1,47	  22,20	   0,95	   23,76	23,76

Montastrea cavernosa	          0,00	            1,07	  20,61	   1,13	   22,06	45,82

Pseudodiploria strigosa	          0,17	            0,97	  19,00	   1,35	   20,33	66,16

Porites astreoides	          0,00	            0,72	  13,27	   1,56	   14,20	80,36

Groups Puerto Real  &  Guabinitas

Average dissimilarity = 63,48

	Group Puerto Real	Group Guabinitas	       	       	        	     

Species	         Av.Abund	        Av.Abund	Av.Diss	Diss/SD	Contrib%	Cum.%

Pseudodiploria strigosa	             2,60	            0,97	  16,39	   1,74	   25,82	25,82

Siderastrea siderea	             0,00	            1,47	  13,34	   0,96	   21,01	46,84

Montastrea cavernosa	             0,00	            1,07	  10,95	   1,26	   17,26	64,09

Orbicella faveolata	             0,00	            0,52	   4,90	   0,95	    7,72	71,81

Porites astreoides	             1,10	            0,72	   4,72	   0,81	    7,44	79,25

Groups Cayo Agua  &  Guabinitas

Average dissimilarity = 88,61

	Group Cayo Agua	Group Guabinitas	       	       	        	     

Species	       Av.Abund	        Av.Abund	Av.Diss	Diss/SD	Contrib%	Cum.%

Madracis mirabilis	           3,47	            0,00	  17,52	   4,15	   19,77	19,77

Orbicella annularis	           3,05	            0,00	  15,36	   3,46	   17,33	37,10

Orbicella faveolata	           3,24	            0,52	  14,13	   3,06	   15,94	53,05

Siderastrea siderea	           0,00	            1,47	   6,87	   0,96	    7,75	60,80

Madracis decactis	           1,10	            0,00	   5,33	   0,96	    6,02	66,82

Colpophyllia natans	           1,15	            0,17	   5,31	   1,27	    5,99	72,81

Pseudodiploria strigosa	           0,00	            0,97	   5,03	   2,56	    5,68	78,49

Groups Boca de Cote  &  Guabinitas

Average dissimilarity = 62,60

	Group Boca de Cote	Group Guabinitas	       	       	        	     

Species	          Av.Abund	        Av.Abund	Av.Diss	Diss/SD	Contrib%	Cum.%

Orbicella faveolata	              3,10	            0,52	  12,80	   2,44	   20,45	20,45

Siderastrea siderea	              2,54	            1,47	   8,02	   1,33	   12,81	33,25

Diploria labyrinthiformis	              1,37	            0,00	   6,91	   2,59	   11,03	44,29

Orbicella annularis	              1,35	            0,00	   6,31	   1,52	   10,08	54,37

Porites astreoides	              1,92	            0,72	   6,09	   1,79	    9,73	64,11

Montastrea cavernosa	              1,76	            1,07	   5,00	   1,23	    7,99	72,09

Madracis mirabilis	              1,01	            0,00	   4,51	   0,74	    7,20	79,29

Groups Dos Mosquises Sur  &  Guabinitas

Average dissimilarity = 86,15

	Group Dos Mosquises Sur	Group Guabinitas	       	       	        	     

Species	               Av.Abund	        Av.Abund	Av.Diss	Diss/SD	Contrib%	Cum.%

Orbicella faveolata	                   3,41	            0,52	  16,22	   2,24	   18,83	18,83

Colpophyllia natans	                   2,54	            0,17	  12,98	   3,32	   15,07	33,90

Madracis mirabilis	                   2,55	            0,00	  12,96	   1,59	   15,04	48,94

Orbicella annularis	                   2,43	            0,00	  12,39	   1,46	   14,38	63,31

Siderastrea siderea	                   0,52	            1,47	   7,57	   1,35	    8,79	72,10

Montastrea cavernosa	                   0,00	            1,07	   5,63	   1,31	    6,53	78,63

Groups Madrisqui  &  Guabinitas

Average dissimilarity = 80,59

	Group Madrisqui	Group Guabinitas	       	       	        	     

Species	       Av.Abund	        Av.Abund	Av.Diss	Diss/SD	Contrib%	Cum.%

Orbicella faveolata	           2,67	            0,52	  15,05	   2,39	   18,68	18,68

Colpophyllia natans	           1,96	            0,17	  12,32	   1,80	   15,28	33,96

Siderastrea siderea	           0,00	            1,47	   9,05	   0,96	   11,23	45,19

Porites astreoides	           1,41	            0,72	   7,18	   1,61	    8,91	54,10

Montastrea cavernosa	           0,52	            1,07	   7,17	   1,26	    8,89	62,99

Pseudodiploria strigosa	           0,00	            0,97	   6,90	   2,33	    8,56	71,55

Agaricia sp.	           0,93	            0,00	   5,59	   0,74	    6,94	78,49

Groups Pelona de Rabusqui  &  Guabinitas

Average dissimilarity = 87,93

	Group Pelona de Rabusqui	Group Guabinitas	       	       	        	     

Species	                Av.Abund	        Av.Abund	Av.Diss	Diss/SD	Contrib%	Cum.%

Orbicella faveolata	                    4,91	            0,52	  27,36	   3,56	   31,11	31,11

Orbicella annularis	                    2,00	            0,00	  11,98	   1,50	   13,63	44,74

Colpophyllia natans	                    1,87	            0,17	   9,98	   1,90	   11,34	56,08

Siderastrea siderea	                    0,27	            1,47	   8,29	   1,12	    9,43	65,51

Agaricia sp.	                    1,10	            0,00	   6,79	   1,44	    7,72	73,23

Montastrea cavernosa	                    0,00	            1,07	   6,32	   1,30	    7,19	80,42

Groups Salinas  &  Guabinitas

Average dissimilarity = 58,53

	Group Salinas	Group Guabinitas	       	       	        	     

Species	     Av.Abund	        Av.Abund	Av.Diss	Diss/SD	Contrib%	Cum.%

Orbicella faveolata	         2,24	            0,52	  11,60	   1,42	   19,82	19,82

Siderastrea siderea	         2,20	            1,47	  10,23	   1,41	   17,48	37,30

Diploria labyrinthiformis	         1,40	            0,00	   8,35	   1,47	   14,27	51,56

Montastrea cavernosa	         1,66	            1,07	   7,07	   1,30	   12,08	63,64

Pseudodiploria strigosa	         1,32	            0,97	   5,30	   1,63	    9,05	72,69

Porites astreoides	         0,68	            0,72	   4,14	   1,32	    7,07	79,76

Groups La Venada  &  Guabinitas

Average dissimilarity = 95,41

	Group La Venada	Group Guabinitas	       	       	        	     

Species	       Av.Abund	        Av.Abund	Av.Diss	Diss/SD	Contrib%	Cum.%

Acropora cervicornis	           4,54	            0,00	  44,20	   5,12	   46,33	46,33

Siderastrea siderea	           0,00	            1,47	  12,17	   0,96	   12,76	59,08

Montastrea cavernosa	           0,00	            1,07	   9,86	   1,28	   10,34	69,42

Pseudodiploria strigosa	           0,27	            0,97	   8,30	   1,51	    8,70	78,12

Groups San Agustin  &  Guabinitas

Average dissimilarity = 95,51

	Group San Agustin	Group Guabinitas	       	       	        	     

Species	         Av.Abund	        Av.Abund	Av.Diss	Diss/SD	Contrib%	Cum.%

Pseudodiploria strigosa	             0,00	            0,97	  22,28	   1,76	   23,33	23,33

Siderastrea siderea	             0,00	            1,47	  22,20	   0,95	   23,24	46,57

Montastrea cavernosa	             0,17	            1,07	  19,41	   1,12	   20,32	66,90

Porites astreoides	             0,00	            0,72	  13,27	   1,56	   13,89	80,78

Groups Blanca  &  Guabinitas

Average dissimilarity = 67,71

	Group Blanca	Group Guabinitas	       	       	        	     

Species	    Av.Abund	        Av.Abund	Av.Diss	Diss/SD	Contrib%	Cum.%

Colpophyllia natans	        3,84	            0,17	  21,52	   5,51	   31,79	31,79

Pseudodiploria strigosa	        2,94	            0,97	  11,48	   3,91	   16,96	48,74

Siderastrea siderea	        0,52	            1,47	   8,03	   1,21	   11,86	60,60

Dichocoenia stokesii	        1,17	            0,00	   6,46	   1,57	    9,54	70,14

Montastrea cavernosa	        1,70	            1,07	   5,10	   1,15	    7,54	77,68

Groups Carabela  &  Guabinitas

Average dissimilarity = 94,09

	Group Carabela	Group Guabinitas	       	       	        	     

Species	      Av.Abund	        Av.Abund	Av.Diss	Diss/SD	Contrib%	Cum.%

Siderastrea siderea	          0,00	            1,47	  20,50	   0,94	   21,79	21,79

Pseudodiploria strigosa	          0,00	            0,97	  19,97	   1,68	   21,23	43,02

Montastrea cavernosa	          0,17	            1,07	  17,47	   1,11	   18,56	61,58

Porites astreoides	          0,00	            0,72	  12,07	   1,51	   12,82	74,41

Colpophyllia natans	          0,66	            0,17	  11,83	   0,79	   12,57	86,98

Groups Punta Cruz  &  Guabinitas

Average dissimilarity = 66,31

	Group Punta Cruz	Group Guabinitas	       	       	        	     

Species	        Av.Abund	        Av.Abund	Av.Diss	Diss/SD	Contrib%	Cum.%

Siderastrea siderea	            0,49	            1,47	  13,15	   1,12	   19,83	19,83

Dichocoenia stokesii	            1,29	            0,00	  12,22	   1,53	   18,43	38,26

Pseudodiploria strigosa	            1,07	            0,97	  10,32	   1,75	   15,56	53,82

Montastrea cavernosa	            1,66	            1,07	  10,02	   1,19	   15,11	68,93

Colpophyllia natans	            0,85	            0,17	   7,42	   1,20	   11,19	80,12

Groups Gabarra  &  Guabinitas

Average dissimilarity = 66,08

	Group Gabarra	Group Guabinitas	       	       	        	     

Species	     Av.Abund	        Av.Abund	Av.Diss	Diss/SD	Contrib%	Cum.%

Siderastrea siderea	         0,76	            1,47	  15,84	   1,18	   23,97	23,97

Montastrea cavernosa	         0,00	            1,07	  12,45	   1,11	   18,85	42,82

Colpophyllia natans	         1,17	            0,17	  11,55	   1,31	   17,47	60,29

Pseudodiploria strigosa	         1,57	            0,97	   8,82	   1,41	   13,35	73,65

Porites astreoides	         0,17	            0,72	   7,45	   1,25	   11,28	84,93

Groups Garrapata  &  Guabinitas

Average dissimilarity = 63,25

	Group Garrapata	Group Guabinitas	       	       	        	     

Species	       Av.Abund	        Av.Abund	Av.Diss	Diss/SD	Contrib%	Cum.%

Colpophyllia natans	           3,62	            0,17	  21,09	   3,90	   33,35	33,35

Pseudodiploria strigosa	           3,23	            0,97	  13,70	   5,50	   21,66	55,01

Montastrea cavernosa	           2,71	            1,07	  10,32	   1,76	   16,31	71,32

Siderastrea siderea	           0,52	            1,47	   8,44	   1,52	   13,34	84,66

Groups Boca Seca  &  Guabinitas

Average dissimilarity = 72,16

	Group Boca Seca	Group Guabinitas	       	       	        	     

Species	       Av.Abund	        Av.Abund	Av.Diss	Diss/SD	Contrib%	Cum.%

Siderastrea siderea	           0,00	            1,47	  14,86	   0,94	   20,60	20,60

Montastrea cavernosa	           0,00	            1,07	  12,51	   1,18	   17,33	37,93

Orbicella faveolata	           0,90	            0,52	  10,57	   1,19	   14,65	52,57

Pseudodiploria strigosa	           0,45	            0,97	   9,21	   1,04	   12,76	65,33

Porites astreoides	           0,75	            0,72	   7,17	   1,22	    9,94	75,27

Groups Bajo Caiman  &  Guabinitas

Average dissimilarity = 81,24

	Group Bajo Caiman	Group Guabinitas	       	       	        	     

Species	         Av.Abund	        Av.Abund	Av.Diss	Diss/SD	Contrib%	Cum.%

Siderastrea siderea	             0,00	            1,47	  15,40	   0,94	   18,95	18,95

Pseudodiploria strigosa	             0,00	            0,97	  13,38	   1,81	   16,47	35,42

Montastrea cavernosa	             0,00	            1,07	  13,04	   1,18	   16,05	51,47

Porites astreoides	             1,01	            0,72	  12,58	   1,31	   15,49	66,95

Orbicella faveolata	             1,12	            0,52	  10,14	   1,45	   12,49	79,44

Groups Medio  &  Guabinitas

Average dissimilarity = 87,95

	Group Medio	Group Guabinitas	       	       	        	     

Species	   Av.Abund	        Av.Abund	Av.Diss	Diss/SD	Contrib%	Cum.%

Orbicella faveolata	       4,49	            0,52	  29,60	   2,65	   33,65	33,65

Siderastrea siderea	       0,00	            1,47	   9,38	   0,94	   10,67	44,32

Agaricia sp.	       1,23	            0,00	   7,93	   1,34	    9,01	53,33

Montastrea cavernosa	       0,00	            1,07	   7,40	   1,26	    8,41	61,74

Pseudodiploria strigosa	       0,17	            0,97	   5,98	   1,50	    6,80	68,54

Madracis sp.	       1,01	            0,00	   5,49	   0,56	    6,24	74,78

Porites astreoides	       0,00	            0,72	   4,93	   1,53	    5,61	80,39

Groups Mero  &  Guabinitas

Average dissimilarity = 77,76

	Group Mero	Group Guabinitas	       	       	        	     

Species	  Av.Abund	        Av.Abund	Av.Diss	Diss/SD	Contrib%	Cum.%

Siderastrea siderea	      0,62	            1,47	  15,29	   1,00	   19,66	19,66

Montastrea cavernosa	      0,00	            1,07	  12,69	   0,98	   16,32	35,98

Pseudodiploria strigosa	      0,17	            0,97	  12,03	   1,09	   15,48	51,46

Porites astreoides	      1,14	            0,72	   9,38	   1,18	   12,06	63,52

Colpophyllia natans	      1,06	            0,17	   8,84	   1,05	   11,37	74,89

Orbicella faveolata	      0,45	            0,52	   6,17	   1,01	    7,93	82,82

Groups Norte  &  Guabinitas

Average dissimilarity = 88,94

	Group Norte	Group Guabinitas	       	       	        	     

Species	   Av.Abund	        Av.Abund	Av.Diss	Diss/SD	Contrib%	Cum.%

Orbicella faveolata	       5,10	            0,52	  26,43	   4,01	   29,71	29,71

Siderastrea siderea	       0,00	            1,47	   7,62	   0,96	    8,56	38,28

Agaricia agaricites (danai)	       1,35	            0,00	   7,58	   1,30	    8,52	46,80

Acropora palmata	       1,19	            0,00	   7,05	   0,93	    7,93	54,74

Orbicella sp.	       1,16	            0,00	   6,27	   0,91	    7,05	61,79

Colpophyllia natans	       1,30	            0,17	   6,06	   1,79	    6,82	68,60

Montastrea cavernosa	       0,00	            1,07	   5,89	   1,32	    6,62	75,22

Groups Sombrero  &  Guabinitas

Average dissimilarity = 72,04

	Group Sombrero	Group Guabinitas	       	       	        	     

Species	      Av.Abund	        Av.Abund	Av.Diss	Diss/SD	Contrib%	Cum.%

Colpophyllia natans	          3,81	            0,17	  18,95	   6,92	   26,30	26,30

Orbicella faveolata	          3,55	            0,52	  16,00	   3,47	   22,21	48,51

Siderastrea siderea	          0,17	            1,47	   7,12	   1,08	    9,88	58,39

Porites astreoides	          1,72	            0,72	   5,41	   1,69	    7,52	65,90

Montastrea cavernosa	          0,85	            1,07	   4,67	   1,15	    6,48	72,38

Pseudodiploria strigosa	          1,17	            0,97	   3,93	   1,29	    5,46	77,84

Groups Sur  &  Guabinitas

Average dissimilarity = 79,01

	Group Sur	Group Guabinitas	       	       	        	     

Species	 Av.Abund	        Av.Abund	Av.Diss	Diss/SD	Contrib%	Cum.%

Orbicella faveolata	     4,60	            0,52	  27,29	   3,53	   34,54	34,54

Colpophyllia natans	     2,70	            0,17	  16,15	   2,55	   20,44	54,99

Siderastrea siderea	     0,00	            1,47	   8,65	   0,96	   10,95	65,93

Montastrea cavernosa	     0,00	            1,07	   6,76	   1,30	    8,55	74,48

Porites astreoides	     0,35	            0,72	   3,58	   1,25	    4,53	79,02

Groups Cienaga este  &  Guabinitas

Average dissimilarity = 74,86

	Group Cienaga este	Group Guabinitas	       	       	        	     

Species	          Av.Abund	        Av.Abund	Av.Diss	Diss/SD	Contrib%	Cum.%

Orbicella faveolata	              3,63	            0,52	  24,18	   3,03	   32,31	32,31

Siderastrea siderea	              0,27	            1,47	  10,06	   1,12	   13,44	45,75

Orbicella annularis	              1,34	            0,00	   9,60	   0,95	   12,82	58,57

Montastrea cavernosa	              0,40	            1,07	   7,29	   1,24	    9,74	68,31

Porites astreoides	              1,53	            0,72	   7,13	   1,15	    9,53	77,84

Groups Cienaga interno  &  Guabinitas

Average dissimilarity = 65,69

	Group Cienaga interno	Group Guabinitas	       	       	        	     

Species	             Av.Abund	        Av.Abund	Av.Diss	Diss/SD	Contrib%	Cum.%

Colpophyllia natans	                 3,09	            0,17	  16,98	   5,86	   25,85	25,85

Orbicella faveolata	                 3,25	            0,52	  15,99	   3,33	   24,34	50,19

Siderastrea siderea	                 0,40	            1,47	   8,10	   1,19	   12,32	62,52

Montastrea cavernosa	                 1,94	            1,07	   6,18	   1,42	    9,41	71,93

Porites astreoides	                 1,28	            0,72	   5,87	   1,39	    8,93	80,86

Groups Cienaga oeste  &  Guabinitas

Average dissimilarity = 62,90

	Group Cienaga oeste	Group Guabinitas	       	       	        	     

Species	           Av.Abund	        Av.Abund	Av.Diss	Diss/SD	Contrib%	Cum.%

Colpophyllia natans	               3,00	            0,17	  19,20	   2,28	   30,53	30,53

Orbicella faveolata	               2,25	            0,52	  10,82	   1,55	   17,20	47,73

Siderastrea siderea	               0,97	            1,47	   9,33	   1,15	   14,84	62,57

Montastrea cavernosa	               1,22	            1,07	   6,78	   1,13	   10,78	73,35

Dendrogyra cylindrus	               0,89	            0,00	   4,60	   0,93	    7,31	80,66
